# National, Regional, and Global Trends in Infertility Prevalence Since 1990: A Systematic Analysis of 277 Health Surveys

**DOI:** 10.1371/journal.pmed.1001356

**Published:** 2012-12-18

**Authors:** Maya N. Mascarenhas, Seth R. Flaxman, Ties Boerma, Sheryl Vanderpoel, Gretchen A. Stevens

**Affiliations:** 1 Department of Epidemiology and Biostatistics, University of California, San Francisco, California, United States of America; 2 Machine Learning Department, School of Computer Science, Carnegie Mellon University, Pittsburgh, Pennsylvania, United States of America; 3 School of Public Policy and Management, H. John Heinz III College, Carnegie Mellon University, Pittsburgh, Pennsylvania, United States of America; 4 Department of Health Statistics and Information Systems, World Health Organization, Geneva, Switzerland; 5 Department of Reproductive Health and Research, World Health Organization, Geneva, Switzerland; University of Bern, Switzerland

## Abstract

**Background:**

Global, regional, and national estimates of prevalence of and tends in infertility are needed to target prevention and treatment efforts. By applying a consistent algorithm to demographic and reproductive surveys available from developed and developing countries, we estimate infertility prevalence and trends, 1990 to 2010, by country and region.

**Methods and Findings:**

We accessed and analyzed household survey data from 277 demographic and reproductive health surveys using a consistent algorithm to calculate infertility. We used a demographic infertility measure with live birth as the outcome and a 5-y exposure period based on union status, contraceptive use, and desire for a child. We corrected for biases arising from the use of incomplete information on past union status and contraceptive use. We used a Bayesian hierarchical model to estimate prevalence of and trends in infertility in 190 countries and territories. In 2010, among women 20–44 y of age who were exposed to the risk of pregnancy, 1.9% (95% uncertainty interval 1.7%, 2.2%) were unable to attain a live birth (primary infertility). Out of women who had had at least one live birth and were exposed to the risk of pregnancy, 10.5% (9.5%, 11.7%) were unable to have another child (secondary infertility). Infertility prevalence was highest in South Asia, Sub-Saharan Africa, North Africa/Middle East, and Central/Eastern Europe and Central Asia. Levels of infertility in 2010 were similar to those in 1990 in most world regions, apart from declines in primary and secondary infertility in Sub-Saharan Africa and primary infertility in South Asia (posterior probability [pp] ≥0.99). Although there were no statistically significant changes in the prevalence of infertility in most regions amongst women who were exposed to the risk of pregnancy, reduced child-seeking behavior resulted in a reduction of primary infertility among all women from 1.6% to 1.5% (pp = 0.90) and a reduction of secondary infertility among all women from 3.9% to 3.0% (pp>0.99) from 1990 to 2010. Due to population growth, however, the absolute number of couples affected by infertility increased from 42.0 million (39.6 million, 44.8 million) in 1990 to 48.5 million (45.0 million, 52.6 million) in 2010. Limitations of the study include gaps in survey data for some countries and the use of proxies to determine exposure to pregnancy.

**Conclusions:**

We analyzed demographic and reproductive household survey data to reveal global patterns and trends in infertility. Independent from population growth and worldwide declines in the preferred number of children, we found little evidence of changes in infertility over two decades, apart from in the regions of Sub-Saharan Africa and South Asia. Further research is needed to identify the etiological causes of these patterns and trends.

*Please see later in the article for the Editors' Summary*

## Abstract 2

Gretchen Stevens and colleagues use information from demographic reproductive health surveys to estimate the global, regional, and country levels, patterns, and trends in infertility between 1990 and 2010.

## Editors' Summary

**Background:**

Reproductive health is a priority global health area: the target for Millennium Development Goal 5B is to provide universal access to reproductive health by 2015. The indicators for monitoring progress in reaching this target are contraceptive prevalence rate, adolescent birth rate, antenatal care coverage, and the unmet need for family planning. Infertility, the inability to conceive after a prolonged period (the length of time varies in different definitions) of unprotected intercourse, is a critical but much neglected aspect of reproductive health. The inability to have children affects couples worldwide and causes emotional and psychological distress in both men and women. Many factors—including physiological, genetic, environmental, and social— contribute to infertility. According to the World Health Organization, infertility resulting from sexually transmitted diseases or reproductive tract infections is particularly problematic in Africa and Latin America.

**Why Was This Study Done?:**

The researchers used a uniform measure of infertility that incorporated live birth as the outcome of interest (as this information is more commonly reported than pregnancies), a five-year “exposure period,” that is, a five-year period of being in an intimate relationship, not using contraceptives, and wanting a child (as the researchers calculated that this period was necessary to accommodate the time it takes to become pregnant and have a child, and to allow for incomplete information on frequency of unprotected intercourse). The researchers used a statistical model (Bayesian hierarchical model) to generate estimates for levels and trends of infertility in 190 countries over the time period 1990 to 2010 using information collected from national demographic and reproductive health surveys. The most data was available for South Asia and Sub-Saharan Africa.

**What Did the Researchers Do and Find?:**

The researchers found that in 2010, 1.9% of women aged 20–44 years who wanted to have children were unable to have their first live birth (primary infertility), and 10.5% of women with a previous live birth were unable to have an additional live birth (secondary infertility). The researchers found that the levels of infertility were similar in 1990 and 2010, with only a slight overall decrease in primary infertility (0.1%, but with a more pronounced drop in Sub-Saharan Africa and South Asia) and a modest overall increase in secondary infertility (0.4%). Age affected infertility rates: the prevalence of primary infertility was higher among women aged 20–24 years than among older women. The age pattern was reversed and even more pronounced for secondary infertility. And primary infertility rates among women wanting children also varied by region, from 1.5% in Latin America and the Caribbean in 2010, to 2.6% in North Africa and the Middle East. With a few exceptions, global and country patterns of secondary infertility were similar to those of primary infertility.

**What Do These Findings Mean?:**

These findings suggest that in 2010, an estimated 48.5 million couples worldwide were unable to have a child after five years. However, these findings also suggest that global levels of primary and secondary infertility hardly changed between 1990 and 2010. It is important to note that an infertility measure based on ability to become pregnant (rather than having a live birth—the outcome used in this study) may show different levels of infertility, and using an exposure period shorter than the five years used in this study would produce higher rates of infertility. However, because of the lack of widespread data collection on time to pregnancy, the methods used and results shown in this study provide useful insights into global, regional, and country patterns and trends in infertility.

**Additional Information:**

Please access these websites via the online version of this summary at http://dx.doi.org/10.1371/journal.pmed.1001356.

- The World Health Organization has information on reproductive health
- Wikipedia defines infertility and gives some useful information (note that Wikipedia is a free online encyclopedia that anyone can edit)
- Patient friendly information on infertility can be found at PubMed Health and NHS Choices

## Introduction

The global health community has had great success in improving maternal and child health in the past decade, partly through a focus on reproductive health [1],[2]. Infertility is a critical component of reproductive health, and has often been neglected in these efforts [3]. The inability to have children affects men and women across the globe. Infertility can lead to distress and depression, as well as discrimination and ostracism [3],[4]. An accurate profile of the prevalence, distribution, and trends of infertility is an important first step towards shaping evidence-based interventions and policies to reduce the burden of this neglected disability globally.

Few comparative analyses of global infertility have been conducted, and none, to our knowledge, have applied a consistent algorithm to demographic and reproductive health survey data from both developing and developed countries, nor used these data to estimate regional and global trends in infertility prevalence. Boivin et al. estimated global infertility by summarizing prevalence data from seven studies: five from developed countries and two from developing countries [5]. A Demographic and Health Surveys (DHS) report also estimated infertility for developing countries using survey data from 47 national DHS surveys [6]. The report's estimate of infertility and analysis of trends did not apply to developed countries, nor to China. Ericksen and Brunette [7] and Larsen [8] applied consistent definitions of infertility in their analyses of household survey data, but considered only Sub-Saharan African countries.

The main challenges in generating global estimates of infertility are the scarcity of population-based studies and the inconsistent definitions used in the few high-quality studies available [9],[10]. In population-based studies of infertility, there has been little consistency in how prevalence is calculated [9],[11]. An explicit detailing of the numerator and denominator of each definition is needed to make clear what is being measured. The authors of a recent literature review concluded that it is not possible to synthesize infertility prevalence data in the published literature because of the incomparable definitions used [9].

An alternative to synthesizing data found in the literature is to apply a consistent definition to regularly collected demographic and reproductive health survey data. In this paper, we used a consistent algorithm to measure infertility using household survey data. Our measure is a demographic definition that uses live birth as the outcome and a 5-y exposure period based on union status, use of contraceptives, and desire for a child [6]–[8],[12]. There are challenges associated with inferring prevalence from household survey data. Few household surveys ask how long the respondent has tried to get pregnant, and none include a comprehensive medical history and clinical examination. Instead, these surveys may collect information on births, couple status, fertility preferences, and contraceptive use. In a previous analysis we performed sensitivity analyses around each of these components to identify important biases that may arise when information is incomplete [13]. We found that a 5-y exposure period is needed to accommodate the time it takes to become pregnant and give birth, and helps prevent unreported temporary separations, periods of postpartum sexual abstinence, or lactational amenorrhea from unduly affecting the infertility measure. Births, rather than pregnancies, are the preferred outcome, as information on live births is collected more often and reported more accurately: neither pregnancies in the first trimester nor voluntary terminations are reliably reported in household surveys [14]–[16]. Lastly, we argued previously that the intent to have a child serves as a proxy for regular, unprotected sexual intercourse, and may correct for underreporting of contraceptive use [13],[17].

Clinical and epidemiologic infertility definitions are also used to monitor infertility; however, they are not appropriate when making population-based estimates of infertility using household surveys. The clinical definition of infertility used by the World Health Organization (WHO) is “a disease of the reproductive system defined by the failure to achieve a clinical pregnancy after 12 months or more of regular unprotected sexual intercourse” [18], while the WHO's epidemiologic definition is “women of reproductive age at risk of becoming pregnant who report unsuccessfully trying for a pregnancy for more than two years” [19]. Clinical definitions are designed for early detection and treatment of infertility [18]–[20]. A definition and assessment of infertility based on medical histories and diagnostic tests is appropriate for clinical settings, where the aim is to understand causes and provide treatment as soon as it is indicated. However, measuring patterns and trends in infertility at the population level necessitates a measure that may be elicited using a standard set of survey questions [17]. The WHO's epidemiologic definition is more closely aligned with clinical practice than demographic definitions are, and may be measured using survey data. However, few household surveys determine whether a couple is trying to become pregnant, and the majority do not collect information on past pregnancies, only on previous live births.

In this study, we analyzed data from a range of reproductive and demographic surveys to estimate infertility prevalence. We applied consistent definitions of primary infertility (inability to have any live birth) and secondary infertility (inability to have an additional live birth). We developed a Bayesian hierarchical model to generate estimates for levels and trends of infertility and their uncertainties by country for the time period 1990 to 2010.

## Methods

### Study Design

We estimated prevalence of primary and secondary infertility, their trends between 1990 and 2010, and their uncertainties, in 190 countries and territories. We used survey data consisting of interviews with the female partner. Although infertility occurs in couples and may have a male or a female cause, estimates are indexed on the woman in each couple. We made estimates for women aged 20–44 y, excluding infertility during the beginning (15–19 y) and end (45–49 y) of the reproductive period, when fewer couples are seeking a child and estimates of prevalence are less stable. We additionally estimated the proportion of women in each region who were exposed to the risk of pregnancy, i.e., those who were in a union, were not using contraceptives, and had a child or wished to have one, either her first (primary infertility) or an additional (secondary infertility) child. We grouped the countries into the seven regions (High Income, Central/Eastern Europe and Central Asia, East Asia/Pacific, Latin America/Caribbean, North Africa/Middle East, Sub-Saharan Africa, and South Asia) and 21 nested subregions of the Institute for Health Metrics and Evaluation Global Burden of Disease 2010 study (Table A in Text S1).

Our analysis included four steps: (1) identification and extraction of data, (2) adjustment of extracted data for known biases as needed, (3) application of a statistical model to estimate infertility prevalence and exposure proportion trends by country and age of the female partner, and (4) calculation of the number of couples currently affected by infertility. We calculated the estimates' uncertainty, taking into account both sampling error and uncertainty from each step of statistical modeling.

### Data Sources

We included data from demographic and reproductive health surveys that we could obtain at the (anonymized) individual level, and hence to which we could apply a consistent definition of infertility. We identified data sources from national demographic studies in a recent systematic literature review of infertility prevalence [9], as well as data that were known to the authors of the present study. To be included, each survey had to collect women's age, current couple status, current contraceptive use, time since first and last births, time since first union, and desire to have a child. Data available only as summary statistics were excluded.

We obtained data from the following survey programs: DHS, Reproductive Health Surveys, the World Fertility Survey, the Pan Arab Project for Family Health and Pan Arab Project for Child Development, the European Multicenter Study on Infertility and Subfecundity, the Fertility and Family Survey, the United States National Survey of Family Growth, and the China In-Depth Fertility Sample Surveys (Table 1; Table A and Figure A in Text S1). We included surveys prior to 1990 to capture heterogeneity in levels of infertility in countries that did not have more recent surveys. For each data source, we recorded information on survey population and sampling strategy. For each female survey respondent, we extracted data on union (marriage or cohabitation), birth history, contraceptive use status and history (if available), and the woman's desire for a child or an additional child. We used stated desire for a child to exclude women who take unreported actions to prevent pregnancies or births, including unreported periods of abstinence or contraceptive use, or voluntary terminations [13]. We included women who were undecided about having additional children and women who declared they were unable to become pregnant in the same category as women who stated they wanted another child, because this group is less likely to be preventing pregnancies or births in ways that are not captured by other survey questions. We refer to these women as women who desire a child. We excluded ten Fertility and Family Surveys and three Reproductive Health Surveys because at least one response was missing for more than 15% of respondents.

**Table 1:** Surveys included in the analysis.

Survey	Region or Country	Survey Sample	Years	Number of Countries (Number of Surveys)
China In-Depth Fertility Sample Surveys	China	Eight provinces in China	1985, 1987	1 (7)
DHS	Developing countries	National	1985–2011	75 (193)
European Multicenter Study on Infertility and Subfecundity	Western Europe	Subnational regions	1992	5
Fertility and Family Survey	Europe	National	1989–1997	12
National Survey of Family and Growth	United States	National	1988, 1995, 2002, 2007	1 (4)
Pan Arab Project for Family Health	Middle East	National	2002–2004	6
Pan Arab Project for Child Development	Middle East	National	1990–1997	10 (13)
Reproductive Health Surveys	Latin America and Eastern Europe	National	1989–2008	7 (11)
World Fertility Survey	Developing countries	National	1974–1981	33

### Prevalence and Exposure Definitions

Mascarenhas et al. evaluated potential bias from using standard demographic or reproductive health surveys to estimate infertility prevalence and recommended the following standard algorithms [13], which we employed (see Figures B and C in Text S1):

Primary infertility is defined as the absence of a live birth for women who desire a child and have been in a union for at least five years, during which they have not used any contraceptives. The prevalence of primary infertility is calculated as the number of women in an infertile union divided by the number of women in both infertile and fertile unions, where women in a fertile union have successfully had at least one live birth and have been in the union for at least five years at the time of the survey.
Secondary infertility is defined as the absence of a live birth for women who desire a child and have been in a union for at least five years since their last live birth, during which they did not use any contraceptives. The prevalence of secondary infertility is calculated as the number of women in an infertile union divided by the combined number of women in infertile and fertile unions. Women in a fertile union have successfully had at least one live birth in the past five years and, at the time of the survey, have been in a union for at least five years following their first birth.

We also calculated the proportion of women of reproductive age (20–44 y) who are exposed to the risk of pregnancy in order to calculate the overall percent of women who are affected by unwanted infertility. Women are exposed if they are fertile, infertile, or their fertility status is not determined at the time of the survey. Specifically:

Exposure to primary infertility is defined as the number of women who are currently in a union, are not using any contraceptives, and desire a child, as well as the women who are currently in a union and have given birth to at least one child. The proportion exposed is calculated as the number of women exposed over the total number of women surveyed (Figure B in Text S1).
Exposure to secondary infertility is defined as the number of women who have had at least one live birth, are currently in a union, are not using any contraceptives, and desire another child, as well as the women who are currently in a union and have given birth to an additional child in the last 5 y. The proportion exposed is calculated as the number of women exposed over the total number of women surveyed (Figure C in Text S1).

A small proportion of DHS surveys in high-fertility countries interview only women who have been in a union. We used exposure data from these surveys for women over age 30 y, as virtually all women in these countries have been in a union by age 30 y.

We applied the above definitions to all of the survey data, generating four indicators for each survey: prevalence of primary and secondary infertility and exposure to primary and secondary infertility. We calculated the effective sample size for each indicator to reflect the subset of survey responses used to calculate primary and secondary infertility and to account for sampling uncertainty (Text A and Table B in Text S1). We did not calculate secondary infertility using survey data from China or make estimates of secondary infertility for China, because survey-based estimates of secondary infertility are difficult to interpret in a setting where government regulations strongly affect decisions around limiting family size.

### Correction of Infertility Prevalence for Incomplete Information on Contraceptive Use and Couple Status

Many household surveys ascertain current contraceptive use, but do not collect information on past contraceptive use over a defined exposure period. Using current contraceptive use as a proxy for use over the past 5 y overestimates infertility prevalence, particularly for secondary infertility among younger couples [13],[21]. Likewise, data on time since first union are available more often than data on the length of the current union. Assuming that exposure is continuous from the time of first, rather than current, union can also lead to biases [13]. We developed regressions to correct infertility estimates generated from surveys that did not provide a measure of continuous contraceptive use and couple status over the exposure period, using data from a subset of DHS surveys that provided complete information (Table B in Text S1). The dependent variable in these regressions was the natural log of the less-biased estimate of prevalence, and the independent variables were the biased estimate, age, and, for secondary infertility, prevalence of contraceptive use (further details in Text B in Text S1). The uncertainty of the estimated prevalences included the statistical sampling uncertainty as well as the uncertainty associated with the correction for incomplete information on contraceptive use and union duration.

### Statistical Analysis

Despite the large number of surveys used in this analysis, data were not available for many country-years of interest. In addition, some of the surveys that we used were not nationally representative. As a result, we developed a statistical model to generate estimates for every country and year, including those for which no data were identified. We estimated four indicators: the prevalence of primary infertility, the prevalence of secondary infertility, and the proportion of couples exposed to each type of infertility (see definitions section above). We made these estimates for 190 countries, the years 1990–2010, and each age group. We used a Bayesian hierarchical model to makes estimates for each country-year-age grouping, informed by the unit, if available, and by data from other units. Text C in Text S1 describes the model in detail, the main features of which are summarized below.

We fit a hierarchical model in which our estimates for countries were nested within subregional, regional, and global levels. Because the model is hierarchical, estimates for each country are informed by data from the country itself, if available, and by data from other countries, especially countries in the same region. A hierarchical model shares information to a greater degree when data are sparse, uncertain, or inconsistent, and to a lesser degree in data-rich countries and regions. We also modeled hierarchical linear time trends. Specifically, region-specific time trends were nested in a global trend. We used a time-varying covariate to inform our estimates, namely, maternal education (average years of schooling for women of reproductive age) [22]. Subnational studies are less informative than national studies, thus we included separate variance components for subnational and national data sources. These variance components were estimated as part of the model fitting process, allowing national data to have greater influence on estimates than subnational data.

Age of the female partner is a major determinant of fertility. We made estimates by 5-y age group for the ages 20–44 y, using indicator variables for each age category. This allowed us to generate a fully flexible age pattern. While the increase in infertility with female age is biologically determined, the age at which women wish to have a child is also culturally determined. Thus, we allowed for different age patterns of exposure to primary fertility in the High Income region, as defined in Table A in Text S1, versus in other regions.

We estimated the following sources of uncertainty (see Texts A–C in Text S1 for details): sampling uncertainty in the data sources, uncertainty associated with the conversion from prevalence estimates using incomplete information on contraceptive use and couple status, uncertainty from study design factors for national surveys, additional uncertainty for non-national data sources, and uncertainty from the use of a model to estimate prevalence of primary and secondary infertility by country, year, and age group where data were not available.

We fit the Bayesian model using Markov chain Monte Carlo methods to obtain 1,600 samples from the posterior distribution of the model parameters, reflecting the uncertainty from each step of the analysis; these parameter values were in turn used to calculate the posterior distribution of each indicator. We calculated trends by subtracting the estimate for 1990 from the estimate for 2010 for each draw. We calculated central estimates as the mean of the draws, and uncertainty intervals as the 2.5th–97.5th percentiles of these draws. We also reported the posterior probability (pp) that an estimated increase or decrease corresponds to a truly increasing or decreasing trend. pp's are not *p*-values; they are probabilities: if the pp of an increase is 0.5 then an increase and a decrease are both equally likely, while a high pp of an increase indicates high certainty that an increase occurred. We considered a trend to be statistically significant if its pp was greater than 0.975. Survey analyses were carried out using Stata 10.1, and Markov chain Monte Carlo analysis was carried out in Python using the PyMC package [23].

We evaluated the predictive validity of our models' central estimates and their uncertainty intervals by performing cross-validation. We ran each model five times, each time withholding data from a random sample of 20% of countries. We then compared the model predictions to the known-but-withheld data. For each model, we calculated the root mean square error, median relative error, and the percent of withheld data that fell within the model's 95% uncertainty interval.

We report four results: prevalence of primary and secondary infertility among child-seeking women, i.e., among women who are exposed to the risk of pregnancy, and the percent of primary and secondary infertility among all women of reproductive age, calculated as the product of the prevalence of infertility among child-seeking women and the proportion who are exposed to the risk of pregnancy. We also calculated the number of couples affected by infertility using population data from the United Nations Population Division's “World Population Prospects: 2010 revision” [24]. We also report two additional indicators, percent of women exposed to the risk of primary and secondary infertility, in Figures H, I, M, and N in Text S1. All estimates were made by country and age; we calculated all-age, regional, and global estimates by weighting country- and age-specific estimates by the population of women in the relevant age group.

## Results

We identified 277 demographic and reproductive health surveys, including seven multi-country programs and two country-specific surveys, that included questions on infertility and for which we could obtain the individual-level questionnaire responses (Table 1; Table B and Figure A in Text S1). National data were available for 101 countries, and regional data were obtained for a further three countries. At least two surveys were available for 69 countries. The South Asia and Sub-Saharan Africa regions had the greatest data availability, with at least one survey available for 67% of countries and an average of more than two surveys per country. There were fewer data available for the High Income and Central/Eastern Europe and Central Asia regions: we did not identify any data for 38 of 59 countries in these regions (64%), and we identified two or more data sources for only nine of these countries.

Predictive validity statistics are shown in Table C in Text S1. Root mean square prediction errors for countries for which data were left out were 1.3% for primary prevalence, 6.1% for secondary prevalence, and 9.1%–13.1% for exposure to primary and secondary fertility (see Figures D–I in Text S1 for graphical presentation of model fit). The models' 95% uncertainty intervals contained 93%–96% of left-out data points.

In 2010, 1.9% of child-seeking women aged 20–44 y were unable to have a first live birth (primary infertility; 95% uncertainty interval 1.7%, 2.2%), and 10.5% of child-seeking women with a prior live birth were unable to have an additional live birth (secondary infertility; 9.5%, 11.7%). Levels of infertility were similar in 1990 and 2010, decreasing 0.1 (−0.1, 0.3) percentage points for primary infertility (from 2.0% [1.9%, 2.2%] in 1990; pp = 0.84) and increasing 0.4 (−0.8, 1.6) percentage points for secondary infertility (from 10.2% [9.3%, 11.1%] in 1990; pp of increase = 0.71).

**Figure 1:**
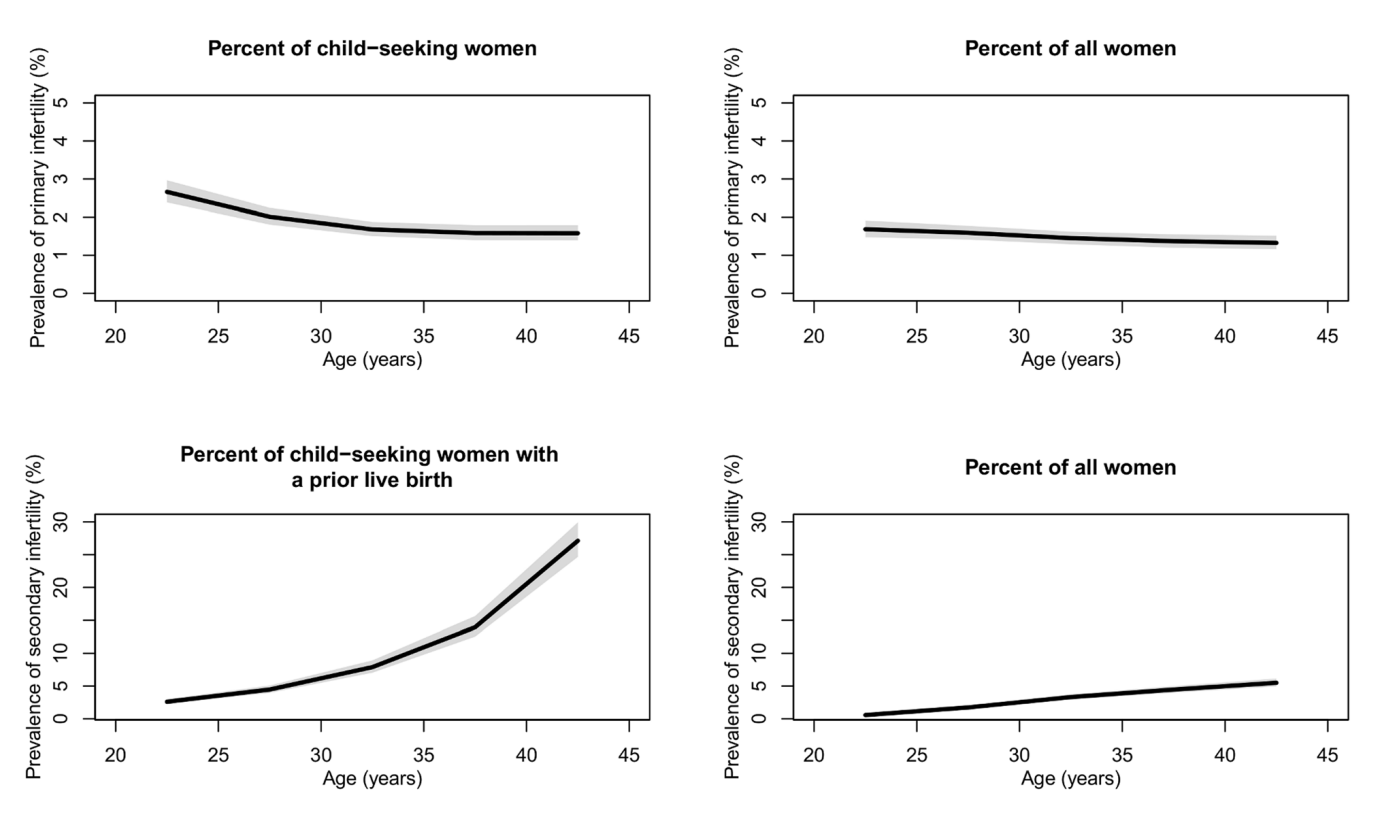
Global prevalence of primary and secondary infertility in 2010, by the female partner's age. Infertility is calculated as the percent of women who seek a child and as the percent of all women of reproductive age. The solid line represents the posterior mean, and the shaded area the 95% uncertainty interval.

Figure 1 presents the prevalence of primary and secondary infertility by age (see Figure J in Text S1 for age pattern of exposure to primary and secondary infertility). The prevalence of primary infertility was higher among women aged 20–24 y (2.7% [2.4%, 3.0%] in 2010) compared to women aged 25–29 y (2.0% [1.8%, 2.2%]) and women aged 30–44 y (ranging from 1.6% to 1.7% in 2010). Prevalence of secondary infertility increased sharply with age, from 2.6% (2.3%, 3.0%) in women aged 20–24 y to 27.1% (24.7%, 29.9%) in women aged 40–44 y. Both age patterns are less pronounced when calculated as a percent of all women (Figure 1).

### Patterns and Trends in Infertility among Child-Seeking Women

**Figure 2:**
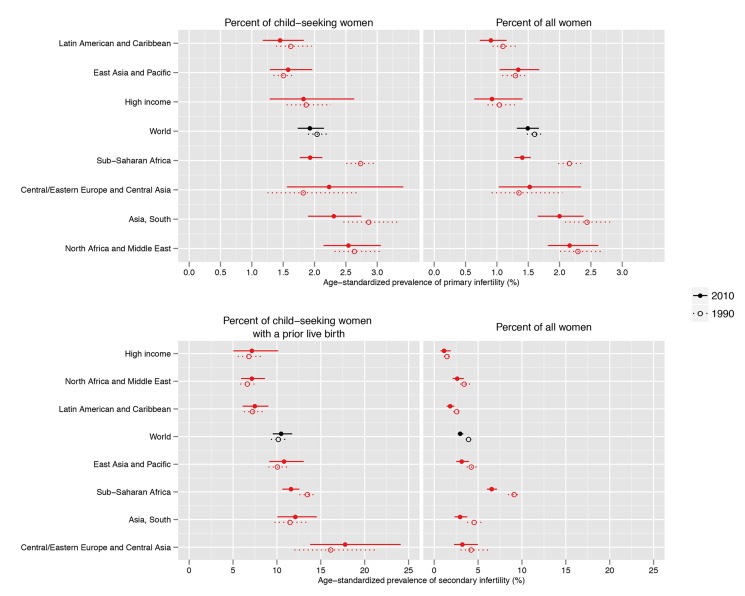
Prevalence of primary infertility and secondary infertility, presented as the percent of women who seek a child, and as the percent of all women of reproductive age, in 1990 and 2010. Infertility prevalence is indexed on the female partner; age-standardized prevalence among women aged 20–44 y is shown here. Horizontal lines indicate the 95% uncertainty interval.

Primary infertility prevalence among child-seeking women varied by region in 2010, from 1.5% (1.2%, 1.8%) in the Latin America/Caribbean region, to 2.6% (2.1%, 3.1%) in the North Africa/Middle East region (Figure 2; Dataset S1). Twenty-year trends in infertility prevalence were not statistically significant in most regions, with low-certainty increases in prevalence in Central/Eastern Europe and Central Asia (0.4 [−0.4, 1.6] percentage points; pp = 0.79) and in the East Asia/Pacific region (0.1 percentage points [−0.2, 0.4]; pp = 0.71), and non-significant declines in the High Income, North Africa/Middle East, and Latin America/Caribbean regions (ranging from 0.0 to 0.2 percentage points; pp 0.56–0.93). In South Asia, the prevalence of primary infertility declined 0.6 (0.1, 1.0) percentage points (pp = 0.99); however, this decline was attenuated, declining 0.3 (−0.3, 1.0) percentage points (pp = 0.88), if World Fertility Surveys data from 1974–1981 were excluded from the model (results not shown). The decline in primary infertility was greatest in Sub-Saharan Africa, which experienced a substantial decline in primary infertility, from 2.7% (2.5%, 3.0%) in 1990 to 1.9% (1.8%, 2.1%) in 2010, a decline of 0.8 (0.5, 1.1) percentage points over the 20-y period (pp>0.99). This resulted in a reordering of the regions by primary infertility prevalence: in 1990, Sub-Saharan Africa and South Asia had the two highest prevalences of primary infertility, and in 2010, they were 4th and 2nd highest of seven regions, respectively.

**Figure 3:**
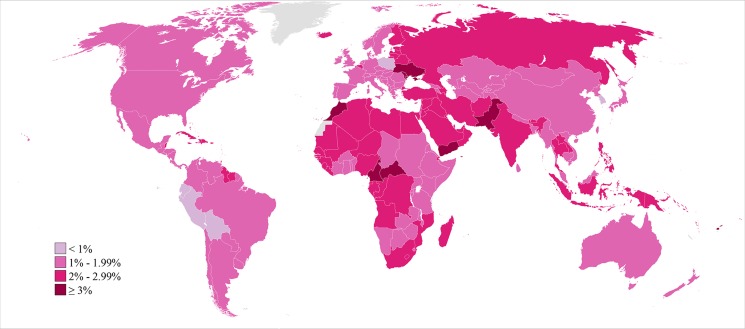
Prevalence of primary infertility among women who seek a child, in 2010. Infertility prevalence is indexed on the female partner; age-standardized prevalence among women aged 20–44 y is shown here.

The prevalence of primary infertility varied within these regions (Figure 3; Dataset S2; Figure K in Text S1). Within the Sub-Saharan Africa region, the prevalence was lowest in East Africa and Southern Africa. Kenya, Zimbabwe, and Rwanda all had low prevalences of primary infertility in Sub-Saharan Africa in 2010 (1.0%–1.1%). In contrast, some countries, mostly in central Sub-Saharan Africa, had very high prevalences: Equatorial Guinea, Mozambique, Angola, Gabon, Cameroon, and the Central African Republic all had prevalences of 2.5% or greater. Primary infertility prevalence also varied within the Latin America/Caribbean region: some Caribbean countries had prevalences of 2.5% or greater in 2010: Jamaica, Suriname, Haiti, and Trinidad and Tobago. In contrast, all countries in Central Latin America and Andean Latin America had prevalences of 1.6% or less.

In 2010, the lowest estimated prevalences of primary infertility occurred in middle-income countries in Latin America (Peru, Bolivia, Ecuador, and El Salvador; 0.8%–1.0%) and in Poland, Kenya, and the Republic of Korea (0.9%–1.0%). At the other extreme, 13 countries in Eastern Europe, North Africa/Middle East, Oceania, and Sub-Saharan Africa had prevalences of 3.0% or greater.

**Figure 4:**
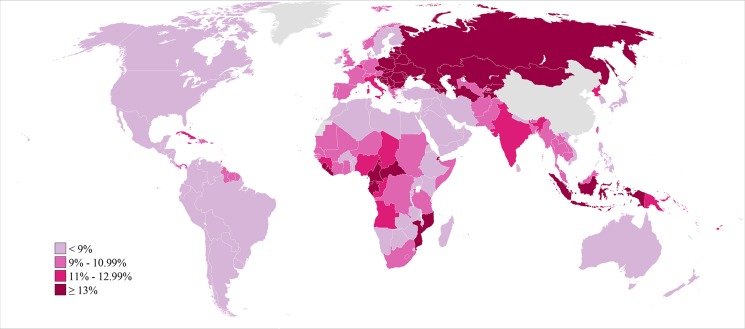
Prevalence of secondary infertility among women who have had a live birth and seek another, in 2010. Infertility prevalence is indexed on the female partner; age-standardized prevalence among women aged 20–44 y is shown here.

Global and country patterns of secondary infertility were similar to those of primary infertility, with two notable exceptions: first, the prevalence of primary infertility was high in some countries in the North Africa/Middle East region, notably Morocco and Yemen, with prevalences greater than 3%, but prevalence of secondary infertility was low in those same countries (Figures 2–4; Dataset S1). Second, the prevalence of primary infertility observed in the Central/Eastern Europe and Central Asia region was low-to-intermediate relative to that of other regions, though this region had the highest prevalence of secondary infertility.

**Figure 5:**
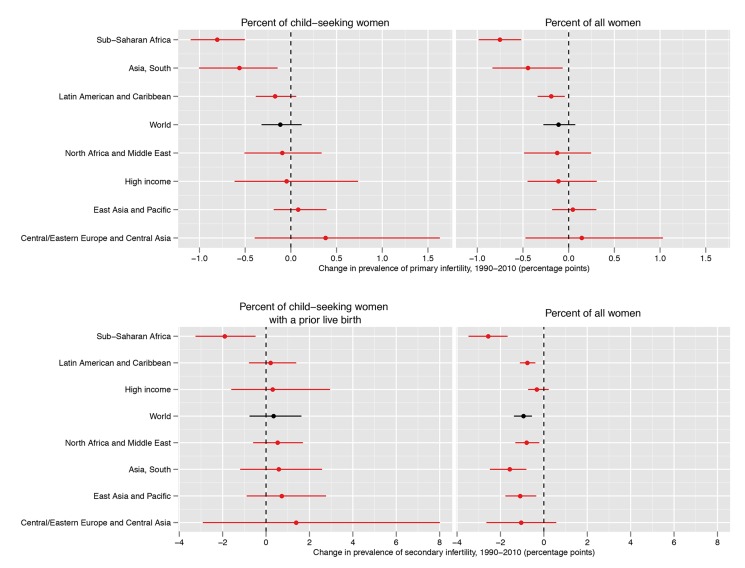
Absolute change in prevalence of primary and secondary infertility, measured as the percent of women who seek a child and as the percent of all women of reproductive age, between 1990 and 2010. Infertility prevalence is indexed on the female partner; change in age-standardized prevalence among women aged 20–44 y is shown here. Horizontal lines indicate the 95% uncertainty interval.

The prevalence of secondary infertility ranged from 7.2% (5.0%, 10.2%) in the High Income region and 7.2% (5.9%, 8.6%) in the North Africa/Middle East region to 18.0% (13.8%, 24.1%) in the Central/Eastern Europe and Central Asia region. Most regions experienced non-significant increases in the prevalence of secondary infertility between 1990 and 2010 (pps = 0.64–0.81; Figure 5), with the exception of Sub-Saharan Africa, where the prevalence of secondary infertility declined from 13.5% (12.5%, 14.5%) in 1990 to 11.6% (10.6%, 12.6%; pp>0.99) in 2010.

Like primary infertility, the prevalence of secondary infertility varied by country within each region, particularly in Sub-Saharan Africa (Figure 4; Dataset S2; Figure L in Text S1). In 2010, eight countries in five regions had a prevalence of secondary infertility below 6%: Rwanda, Jordan, Peru, United States of America, Bolivia, Egypt, Tunisia, and Viet Nam. At the other extreme, 19 countries in Central/Eastern Europe and Central Asia and four in Sub-Saharan Africa had prevalences greater than 16%.

### Patterns and Trends in Infertility among All Women of Reproductive Age

Mirroring worldwide declines in fertility, the proportion of women with one or more children who are at risk of pregnancy has decreased since 1990 in every world region (Figure N in Text S1). This has resulted in a decrease in the percent of women of reproductive age who are affected by secondary infertility in every world region (Figure 5; Figures O and P in Text S1). Worldwide, the age-standardized percent of women aged 20–44 y affected by secondary infertility has decreased from 3.9% (3.6%, 4.3%) to 3.0% (2.7%, 3.3%); the pp of this decline being real is ≥0.9 in the High Income and Central/Eastern Europe and Central Asia regions, and ≥0.99 globally and in all other world regions. The proportion of women who want a first child has decreased less over time, meaning that the proportion of women who are affected by primary infertility has changed little, from 1.6% (1.5%, 1.7%) in 1990 to 1.5% (1.3%, 1.7%) in 2010 (pp = 0.90).

Worldwide, 48.5 million (45.0 million, 52.6 million) couples are unable to have a child, of which 19.2 million (17.0 million, 21.5 million) couples are unable to have a first child, and 29.3 million (26.3 million, 32.6 million) couples are unable to have an additional child (the latter figure excludes China). 14.4 million (12.2 million, 16.8 million) of these couples live in South Asia, and a further 10.0 million (9.3 million, 10.8 million) live in Sub-Saharan Africa. The number of couples suffering from infertility has increased since 1990, when 42.0 million (39.6 million, 44.8 million) couples were unable to have a child. Though the number of infertile couples has increased globally and in most regions, it has decreased from 4.2 million in 1990 to 3.6 million in 2010 in the High Income region, and from 4.4 million in 1990 to 3.8 million in 2010 in the Central/Eastern Europe and Central Asia region. Although there were no significant changes in the prevalence of infertility amongst child-seeking women, reduced child-seeking behavior coupled with a lack of population growth resulted in a decrease in the absolute number of infertile couples in these regions.

## Discussion

In 2010, an estimated 48.5 million (45.0 million, 52.6 million) couples worldwide were infertile. Between 1990 and 2010, levels of primary and secondary infertility changed little in most world regions. The exceptions were Sub-Saharan Africa and South Asia (for primary infertility only), where infertility prevalence decreased during the 20-y period. Reduced child-seeking behavior (i.e., reduced exposure to pregnancy due to changing fertility preferences) means that even where infertility prevalence among those exposed to the risk of pregnancy did not change, a decreasing proportion of couples were affected by infertility because fewer attempted to have a child. However, the absolute number of infertile couples increased due to population growth.

Our estimate of the global number of couples affected by infertility is lower than that of Boivin et al. [5] or Rutstein and Shah [6]. Boiven et al. estimated 72.4 million women were currently infertile in 2006 [5]. They used the median prevalence reported by seven published infertility studies that used a 12- or 24-mo definition of infertility; our estimates differ because we used a larger dataset and a different algorithm to calculate infertility [5],[10]. Rutstein and Shah presented a variety of infertility measures using DHS data from the late 1990s, demonstrating the importance of choices in defining infertility [6]. They estimated that 186 million ever-married women in developing countries (excluding China) were infertile in 2002; this larger number is a result of definitional differences: they included women who may not have been exposed to the risk of pregnancy and women aged 15–20 y and 45–49 y, age groups that have higher prevalences of infertility than women aged 20–44 y.

The strengths of this study were the application of consistent algorithms to calculate primary and secondary infertility from 277 survey datasets, most of which were nationally representative; our use of a Bayesian hierarchical model to estimate infertility prevalence and trends; and our systematic quantification of uncertainty. We identified where survey data did not collect information on past contraceptive use or marital status, and corrected for biases that arose when information on contraceptive use or marriage was incomplete. We used definitions of primary and secondary infertility that allowed us to disentangle trends in ability to have a child from trends in fertility preferences [25]. Specifically, women who were not in a union, had used any contraceptive in the previous 5 y, or did not wish to have a child were excluded from both the numerator and the denominator when calculating the prevalence of infertility. This allowed us to calculate trends in infertility that were independent from worldwide declines in the preferred number of children and independent of population growth in that time period.

The major limitations of our study are gaps in data for certain countries, the use of proxies to assess exposure to pregnancy, potential reporting inaccuracies, and the inability of our definition to capture all instances of infertility. Despite extensive data seeking, data gaps remained, especially in high-income countries and in Central and Eastern Europe. The use of demographic and reproductive health surveys to infer infertility prevalence requires several assumptions. First, we assume that women who are in a union, wish to have a child, and are not using contraceptives are engaged in regular, unprotected sexual intercourse. We also rely on women's reported couple status, births, contraceptive use, and desire for a child. These assumptions may be violated, as women may not report accurately on sensitive topics, such as past voluntary abortions [26],[27]. Women might also report non-biological children as their own. Furthermore, the reporting of the date of marriage and date of last birth may not be accurate in some settings [7]. Several studies have found that, in China, reporting of births in household surveys may be suppressed or the timing of births may be misreported because of policy considerations, which could affect our infertility estimates [28]–[30]. Finally, infertile women may state that they do not want a child, as a coping mechanism [17],[31]. Our correction of incomplete contraceptive and marriage information, use of birth as the outcome, and use of a 5-y infertility definition reduced the susceptibility of our estimates to these biases [13]. Some types of infertility are not measured using our algorithm [32]. The algorithm cannot capture any infertile men whose female partners conceive and give birth to a child with another man, nor primary infertility in men who have had multiple partners. It is not possible to capture infertile couples trying to have a child but using condoms intermittently for sexually transmitted infection (STI) prevention [21]. Lastly, our 5-y definition excludes from the prevalence estimation men and women who do not maintain a union for 5 y. Our prevalence estimate of infertility, however, is applied to all couples in a union, independent of the length, to calculate absolute numbers of couples affected. To the extent that infertile unions are more likely to dissolve than fertile unions, we expect our estimate to be biased downwards because we only measure infertility in unions that last for 5 y [33].

There are several important implications of the algorithm we use to measure infertility. We measure current infertility using a 5-y exposure with birth as an outcome. An infertility measure based on ability to become pregnant may have different patterns, trends, and levels than those presented in this paper. Infertility prevalences measured using a shorter exposure period would have a similar geographic and temporal pattern, but would be approximately twice as high as our estimates (see Figure Q in Text S1; [13]) The shorter exposure period identifies couples affected by temporary separations or periods of abstinence or lactational amenorrhea, infertility that resolves at between 2 and 5 y, and infertile unions that dissolve after 2 y but before 5 y without a birth. Our algorithm does not capture childlessness experienced by couples who are no longer of reproductive age or infertility experienced by women aged less than 20 y. Infertility that is identified and successfully treated within a 5-y period is not captured by this definition. Finally, men and women who use contraceptives, choose to be childless, or are not in a union, may indeed be infertile. However, these individuals are not included in our estimate of the number of infertile unions. We aimed to calculate the number of couples currently affected by infertility, and these individuals are not currently attempting to have a child, or, in the case of those not in a union, it is not possible to determine whether they are attempting to have a child.

Multiple factors—infectious, environmental, genetic, and even dietary in origin—can contribute to infertility [34]. These factors may affect the female, the male, or both partners in a union, resulting in an inability to become pregnant or carry a child to term. Current evidence, mostly from clinical studies with few exceptions [35], indicates that differences in the incidence and prevalence of infectious diseases, leading to fallopian tube blockage in women, are the main reason for changes over time and differences between populations [36]–[39]. Some have hypothesized that sperm quality is declining [40], but the evidence is not conclusive [41].

Increasing age at childbearing could also increase the prevalence of infertility, as the ability to become pregnant and deliver a live birth reduces with age in all populations. Globally, the mean age at childbearing has remained the same (about 28 y) since the 1970s, although this masks regional and temporal heterogeneity in trends [42]. In low- and middle-income countries, age at first birth has increased, although first birth still occurs at young ages: in 40 countries with one DHS survey in the 1990s and another survey during 2000–2011, the overall median of the median age at first birth among women aged 25–49 y increased from 19.8 to 20.3 y [42]. While the age at first birth has increased, the average number of children has decreased, and thus, the mean age at childbearing has not changed in these countries [42]. On the other hand, mean age at first birth and mean age at childbearing have increased in all developed countries since the 1990s [42],[43]. This does not appear to have affected primary infertility levels in those countries. However, it may have contributed to the modest increase in secondary infertility that we estimated.

The geographic pattern of infertility prevalence we found is consistent with previous estimates of infertility in Sub-Saharan Africa, specifically high prevalence in some West, Central, and Southern African countries, and low prevalence in most East African countries [7],[8],[44]. This pattern has mainly been attributed to the consequences of untreated reproductive tract infections, including both STIs such as *Neisseria gonorrhoeae* and *Chlamydia trachomatis*, and, to a lesser extent, infections from unsafe abortions or obstetric practices [34],[36],[45]. The improved trends for the region as a whole may be due to reduced prevalence of STIs, possibly associated with changes in sexual behavior and STI treatment in response to the HIV epidemic. There are, however, no reliable data on regional trends in the prevalence of STIs. WHO estimated that the prevalence of *C. trachomatis* infections among adult females in 2005 was 4%–6% in all regions of the world, except the WHO Eastern Mediterranean and South East Asia regions, where prevalence was below 2% [46]. *N. gonorrhoeae* was considerably more prevalent in the WHO African region than all other regions among adult women and men. If the prevalence of maternal syphilis has decreased since 1990, it may have reduced the risk of stillbirths and therefore increased the ability to have a live birth, which is our definition of fertility [47]–[50]. Infection is also associated with reduced fertility. Infertile women, especially those with primary infertility, are more likely to acquire HIV infection because of greater marital instability [51], and HIV is also associated with reduced fertility in the later stages of infection [52]. However, the population effect of the HIV epidemic on fertility is likely small: despite the epidemic, infertility declined in all Sub-Saharan African subregions.

Post-abortion complications are also an important factor contributing to infertility. The risk is higher for unsafe practices than for safe abortion procedures. The relatively high levels of secondary infertility in the Central/Eastern Europe and Central Asia region may be associated with the higher incidence of abortion. In these regions, the abortion rate declined between 1995 and 2003, but stayed at levels higher than the global average [16]. Both induced abortions and higher levels of STIs/HIV may play a role in explaining the elevated levels of secondary infertility in the Caribbean. Declines in unsafe abortion rates in Sub-Saharan Africa between 1995 and 2003 may have contributed to declines in infertility rates [16].

Among women who have had a pregnancy or birth, pregnancy complications may cause infections of the reproductive tract that result in infertility. Maternal mortality ratios—an indicator of obstetric risk—are estimated to have declined slightly in Sub-Saharan Africa and more substantially Southern Asia since 1990, and it is possible that injuries/infections caused or aggravated by childbirth declined together with decreases in maternal mortality [2].

Including questions on how long women have tried to become pregnant in national or international survey programs would allow for the use of a definition that is more closely aligned with clinical practice than the algorithm used in this study. This may lead to more reliable estimation of levels and trends in infertility than current methods, which in turn would inform policy and program requirements to address this neglected area of reproductive health. However, in the absence of widespread data collection on time to pregnancy, the methods used and results presented here provide valuable insights into global, regional, and country patterns and trends in infertility.

## Supporting Information

Dataset S1 Prevalence of primary and secondary infertility by region and globally, 1990 and 2010.
(XLSX)

Dataset S2 Prevalence of primary and secondary infertility by country, 1990 and 2010.
(XLSX)

Text S1 Additional methods and results.
(PDF)

## Abbreviations

DHS: Demographic and Health Surveys
pp: posterior probability
STI: sexually transmitted infection
WHO: World Health Organization
