# Supplementary material for: National, Regional, and Global Trends in Infertility Prevalence Since 1990: A Systematic Analysis of 277 Health Surveys
Source: PLoS Med. 2012 Dec 18;9(12):e1001356. doi: 10.1371/journal.pmed.1001356 (PMC3525527; doi:10.1371/journal.pmed.1001356)
Supplement: Text S1 — Additional methods and results. (PDF) [file pmed.1001356.s003.pdf]

Text A. Accounting for complex survey design

All of the surveys in our analysis used complex survey designs. Specifically, in designing a representative survey, the target populations were usually divided into strata based on geographical regions within the country and whether place of residence was rural or urban; within each stratum, a number of clusters were randomly selected. Clusters may be villages, administrative units, or census units. Households or participants were then randomly sampled within each cluster. Because total population may differ among strata and clusters, individuals or households in smaller units have a higher probability of being selected than those in larger units. To account for the differences in probability of being sampled, each observation is assigned a sample weight. These weights are calculated to make the survey data representative of the total population.

An implication of the sampling method is that the so-called effective sample size of the survey (ESS) is different from its actual sample size. This occurs primarily because individuals are clustered: though still nationally representative, cluster-based sampling is different than performing a true random sample of the entire population. To accurately incorporate this information into our model, we estimated ESS using the *estat effects* command of the Stata version 10.1 *svy* suite of commands (StataCorp, 2009). This command generates the design effect (DEFF), which is the ratio between the (usually smaller) effective sample size and real sample size, *e.g.* a survey with 1000 subjects with a DEFF of 2.0 has an ESS of 500.

The DEFF may differ for different indicators (primary *vs.* secondary infertility). We calculated an ESS n for each survey-indicator pair, and used n and the calculated prevalence as the inputs into the Bayesian hierarchical model described in Text C.

Text B. Methods to account for bias due to incomplete information on marriage status and contraceptive use

Many household surveys ascertain current contraceptive use, but do not collect information on past contraceptive use over a defined exposure period. Likewise, data on time since first union are available more often than data on couple status during a defined exposure period. Failure to account for changing contraceptive use over time can lead to biases in secondary infertility estimates of more than 100% for some age groups, and assuming that exposure is continuous from the time of first union can lead to smaller biases, generally under 5%[1]. We used data from surveys that collected data on continuous contraceptive use and couple status to develop regression equations to predict unbiased estimates of infertility (taking into account continuous contraceptive use and union status) from biased estimates (calculated assuming that current contraceptive use reflects past contraceptive use, and that the respondant has been in a union continuously since her first union).

Two linear regressions were developed to estimate unbiased primary and secondary infertility from biased primary and secondary infertility estimates, respectively. This analysis pooled household surveys which collected continuous contraceptive use and union status, a total of 53 Demographic and Health Surveys (DHS; see Table B). For primary infertility, the dependent variable was the natural log of the unbiased estimate of infertility, and the independent variables were the natural log of the biased estimate, and a an indicator variable for women under age 30, among whom current contraception use as a proxy for past use leads to a larger bias[1]. For secondary infertility, the dependent variable was the natural log of the unbiased estimate of infertility prevalence; independent variables were the natural log of the biased estimate, age, square of age, and the prevalence of contraceptive use in the survey sample. Regressions were restricted to surveys in which 30 or more individuals were identified as infertile to avoid using uncertain data points to fit the regression. We calculated uncertainty in the predicted prevalences using the standard error predicted using the $stdf$ option in Stata 10.1; this option provides a standard error for a single prediction or forecast[2].

Predictive variables in ordinary least squares linear regression to predict primary infertility prevalence. Dependent variable is the natural log of the unbiased prevalence estimate. N = 75 and the adjusted R^2 of the regression is 0.928.

**Predictor**	**Coefficient**
Constant	-0.23 (-0.47, -0.01)
Natural log of biased prevalence estimate (calculated using current contraceptive use as a proxy for past use, and time since first union as a proxy for time in a union)	0.95 (0.89, 1.02)
Indicator variable equal to 1 for ages < 30 and equal to 0 for ages ≥ 30	-0.042 (-0.080, -0.003)

Predictive variables in ordinary least squares linear regression to predict secondary infertility prevalence. Dependent variable is the natural log of the unbiased prevalence estimate. N = 197 and the adjusted R^2 of the regression is 0.972.

**Predictor**	**Coefficient**
Constant	-2.8 (-3.4, -2.1)
Natural log of biased prevalence estimate (calculated using current contraceptive use as a proxy for past use, and time since first union as a proxy for time in a union)	1.03 (0.97, 1.09)
Age (years)	0.14 (0.10, 0.18)
Square of age (years)	-0.0017 (-0.0024, -0.0010)
Prevalence of contraceptive use in the survey sample	-0.54 (-0.73, -0.35)

Text C. Bayesian hierarchical model

We fit four Bayesian hierarchical logistic regressions to separately estimate the prevalence of and exposure to primary and secondary infertility for couples in each country-age group, indexed by the age of the female partner. We selected uninformative priors for all parameters fit in this model. For observation h, we define y_h to be the number of subjects with infertility (as defined in the main text) out of a total effective sample size n_h. Our model includes a 6-level hierarchy:

- 1,653 observations[1] are denoted o and indexed by h.
- 277 studies are denoted st and indexed by i.
- 104 countries are denoted c and indexed by j.
- 21 subregions are denoted sb and indexed by k.
- 8 regions are denoted r and indexed by l.
- Global-level effects are denoted g.

Our logistic regression is as follows, with covariates X defined below. p_h is the prevalence of infertility for a given observation h:

$$\begin{aligned} y_h &\sim \text{Binomial}(n_h, p_h), \\ p_h &= \text{logit}^{-1}\left(a^g + a^r_{l[h]} + a^{sb}_{k[h]} + a^c_{j[h]} + a^{st}_{i[h]} + a^o_h + b^g t_{i[h]} + b^r_{l[h]} t_{i[h]} + X_h \beta\right), \end{aligned}$$

[1] Chinese data were used only for primary infertility, and some DHS data were not used for the exposure models because the surveys only included ever-married women (see main text and Table B).

We calculate y_h based on the analyses described in Text A and Text B, in order to reflect uncertainty from each survey's complex design and the uncertainty from the adjustments we make to prevalence. In Text B we described our methods to estimate unbiased primary and secondary infertility from biased primary and secondary infertility. This gave us unbiased estimates and standard errors surrounding these estimates. To propagate these errors, we adjusted the variance for observations from bias-corrected studies in our Bayesian model as follows: rather than using the y_h's as the data in our likelihood function at each iteration of our sampler, we first multipled each y_h by a draw from a log-normal distribution with mean 1 and standard deviation equal to the standard error calculated for the bias-correction.

The hierarchical a's have normally distributed priors:

$$\begin{aligned}
a^g &\sim \mathcal{N}(0, 1000), \\
a^r_l &\sim \mathcal{N}\left(0, \sigma^2_r\right), \\
a^{sb}_k &\sim \mathcal{N}\left(0, \sigma^2_{sb}\right), \\
a^c_j &\sim \mathcal{N}\left(0, \sigma^2_c\right), \\
a^{st}_i &\sim \mathcal{N}\left(0, \sigma^2_{st,i}\right), \\
a^o_h &\sim \mathcal{N}\left(0, \sigma^2_o\right).
\end{aligned}$$

where the global, regional, subregional, and country standard deviations were given uniform priors $\mathcal{U}(0, 100)$.

Study-specific random effects allow for additional variability in studies not explained by sampling uncertainty. The variance of the study-specific random effects depends on whether the study is national or subnational, so:

$$\sigma_{st,i} = \begin{cases} \sigma^{\text{national}} & \text{if study } i \text{ is nationally representative} \\ \sigma^{\text{subnational}} & \text{otherwise.} \end{cases}$$

The a^o_h error terms, one per observation, allow for an overdispersed binomial distribution in our data. The resulting model is called the Binomial-normal model[3]. Since this parameter is constrained by a common normal distribution with variance σ^2_o, the degree of overdispersion that is necessary is informed by the data, and shrinks to zero if the data are binomially distributed.

Year of study denoted t was included as a hierarchically modeled covariate. A global slope b^g had prior $\mathcal{N}(0, 1000)$ and additionally separate region-specific slopes $b^r_{l[h]}$ had normal priors with mean zero and a shared standard deviation with a uniform prior $\mathcal{U}(0, 100)$. Before fitting the model, year values were normalized to have mean 0 and standard deviation 1.

Additional covariates representing characteristics of the data at the observation, study, and country levels were included. The observation-specific characteristics differed by indicator modeled. For all models, the observation-specific covariates included indicators for each age category, denoted by indicator variables $z^0, z^1, \ldots, z^4$ (corresponding to membership in the age groups 20-24, 25-29, 30-34, 35-39, 40-44). To model the exposure to primary infertility, we additionally included interactions between indicators for each age category and high income status, denoted by indicator variables $v^0, v^1, \ldots, v^4$ (corresponding to membership in a high income region and in the age groups 20-24, 25-29, 30-34, 35-39, 40-44). The age coefficients shared a normal prior with mean 0 and uniform $\mathcal{U}(0, 20)$ standard deviation. The study-level covariate is whether the study is from the World Fertility Survey series $\text{wfs}_{i[h]}$ (an indicator variable), with prior $\mathcal{N}(0, 1000)$. The country-level covariate is mean years of maternal education $\text{mat}_{j[h]}$, with prior $\mathcal{N}(0, 1000)$. Mean years of education was normalized to have mean 0 and standard deviation 1.

For primary and secondary prevalence of infertility and exposure to secondary infertility, our covariates are:

$$X_h\beta = \beta_0 z^0_h + \beta_1 z^1_h + \beta_2 z^2_h + \beta_3 z^3_h + \beta_4 z^4_h + \beta_5 \text{wfs}_{i[h]} + \beta_6 \text{mat}_{j[h]}$$

For exposure to primary infertility, our covariates are:

$$X_h\beta = \beta_0 z_h^0 + \beta_1 z_h^1 + \beta_2 z_h^2 + \beta_3 z_h^3 + \beta_4 z_h^4 + \beta_5 v_h^0 + \beta_6 v_h^1 + \beta_7 v_h^2 + \beta_8 v_h^3 + \beta_9 v_h^4 + \beta_{10}\mathrm{wfs}_{i[h]} + \beta_{11}\mathrm{mat}_{j[h]}$$

Other than age (described above) the covariates were all assigned uninformative uniform priors.

To fit the parameters of our Bayesian hierarchical model to our dataset, we used the PyMC package in python[4], which provides an implementation of Markov chain Monte Carlo (MCMC) sampling. We specified our model as described above. For each of the four indicators (primary or secondary, prevalence or exposure), we had the following sampling scheme: we ran four chains in parallel. After a burn-in of 5 million iterations, we ran each chain for an additional 5 million iterations, and then thinned our chains by a factor of 12,500, thus obtaining 400 posterior draws from each chain for a total of 1,600 draws for each model.

To assess mixing and convergence, we inspected graphical summaries, including plots of traces and autocorrelation plots, and also summary plots comparing parameter estimates from different chains for the same model. The sampling scheme described above was arrived at through tuning based on trace and autocorrelation plots, which showed us how well the MCMC sampler was exploring the parameter space—we used a large number of iterations and thinned our chains because we had high autocorrelation initially. We also calculated the Gelman-Rubin R-hat statistic[5], which allowed us to monitor convergence by ensuring that the parallel chains eventually converged to the same distribution for each parameter.

To predict primary and secondary infertility and exposure for each country-year-age unit, we include global, regional, and country effects, covariate effects, and age effects. Study effects (a^{st}) and error terms (a_h^o) are not included when making predictions, and the World Fertility Survey covariate $\mathrm{wfs}_{i[h]}$ is set to 0. For predictions we use the following equation, where the values of the parameters from the MCMC sampling at each iteration give a separate posterior prediction at that iteration:

$$p_h = \mathrm{logit}^{-1}\left(a^g + a_{l[h]}^r + a_{k[h]}^{sb} + a_{j[h]}^c + b^g t_{i[h]} + b_{l[h]}^r t_{i[h]} + X_h\beta\right)$$

References

1. M.N. Mascarenhas, H. Cheung, C.D. Mathers, and G.A. Stevens. Measuring infertility in populations: constructing a standard definition for use with demographic and reproductive health surveys. *Population health metrics*, x(x):x–x, 2012.

2. C.F. Baum. *An introduction to modern econometrics using Stata*. Stata Corp, 2006.

3. A. Gelman and J. Hill. *Data analysis using regression and multilevel/hierarchical models*. Cambridge University Press, 2007.

4. A. Patil, D. Huard, and C.J. Fonnesbeck. Pymc: Bayesian stochastic modelling in python. *Journal of statistical software*, 35(4):1, 2010.

5. A. Gelman and D.B. Rubin. Inference from iterative simulation using multiple sequences. *Statistical science*, 7(4):457–472, 1992.

**Table A.** Countries and territories in analysis regions and subregions. Countries not in the high-income region are considered to be low- or middle-income countries.	
**High Income region**	
Asia-Pacific, high income	Brunei Darussalam, Japan, Republic of Korea, Singapore
Australasia	Australia, New Zealand
North America, high income	Canada, United States of America
Western Europe	Andorra, Austria, Belgium, Cyprus, Denmark, Finland, France, Germany, Greece, Iceland, Ireland, Israel, Italy, Luxembourg, Malta, Netherlands, Norway, Portugal, Spain, Sweden, Switzerland, United Kingdom
**Central/Eastern Europe and Central Asia region**	
Central Asia	Armenia, Azerbaijan, Georgia, Kazakhstan, Kyrgyzstan, Mongolia, Tajikistan, Turkmenistan, Uzbekistan
Central Europe	Albania, Bosnia and Herzegovina, Bulgaria, Croatia, Czech Republic, Hungary, Macedonia (Former Yugoslav Republic of), Montenegro, Poland, Romania, Serbia, Slovakia, Slovenia
Eastern Europe	Belarus, Estonia, Latvia, Lithuania, Moldova, Russian Federation, Ukraine
**East Asia/Pacific region**	
East Asia	China, Hong Kong SAR (China), Macau SAR (China), Democratic People's Republic of Korea, Taiwan
Southeast Asia	Cambodia, Indonesia, Lao People's Democratic Republic, Malaysia, Maldives, Myanmar, Philippines, Sri Lanka, Thailand, Timor-Leste, Viet Nam
Oceania	Fiji, Kiribati, Marshall Islands, Micronesia (Federated States of), Papua New Guinea, Samoa, Solomon Islands, Tonga, Vanuatu
**Latin America/Caribbean region**	
Andean Latin America	Bolivia, Ecuador, Peru
Central Latin America	Colombia, Costa Rica, El Salvador, Guatemala, Honduras, Mexico, Nicaragua, Panama, Venezuela (Bolivarian Republic of)
Southern Latin America	Argentina, Chile, Uruguay
Tropical Latin America	Brazil, Paraguay
Caribbean	Antigua and Barbuda, Bahamas, Barbados, Belize, Cuba, Dominica, Dominican Republic, Grenada, Guyana, Haiti, Jamaica, Puerto Rico, Saint Lucia, Saint Vincent and the Grenadines, Suriname, Trinidad and Tobago
**North Africa/Middle East region**	
North Africa and Middle East	Algeria, Bahrain, Egypt, Iran (Islamic Republic of), Iraq, Jordan, Kuwait, Lebanon, Libyan Arab Jamahiriya, Morocco, Occupied Palestinian Territory, Oman, Qatar, Saudi Arabia, Syrian Arab Republic, Tunisia, Turkey, United Arab Emirates, Yemen
**South Asia region**	
South Asia	Afghanistan, Bangladesh, Bhutan, India, Nepal, Pakistan
**Sub-Saharan Africa region**	
Central Africa	Angola, Central African Republic, Congo, Democratic Republic of the Congo, Equatorial Guinea, Gabon
East Africa	Burundi, Comoros, Djibouti, Eritrea, Ethiopia, Kenya, Madagascar, Malawi, Mauritius, Mozambique, Rwanda, Somalia, Sudan, Uganda, United Republic of Tanzania, Zambia
Southern Africa	Botswana, Lesotho, Namibia, South Africa, Swaziland, Zimbabwe
West Africa	Benin, Burkina Faso, Cameroon, Cape Verde, Chad, Côte d'Ivoire, Gambia, Ghana, Guinea, Guinea-Bissau, Liberia, Mali, Mauritania, Niger, Nigeria, Senegal, Sierra Leone, São Tomé and Príncipe, Togo

Table B. Data sources and their characteristics. Data are nationally representative unless otherwise noted.

Country	Year	Survey	Notes	Sample Size (Primary)*	Sample Size (Secondary)*	Survey Size**
Albania	2002	Reproductive Health Survey	1	3102	1026	4671
Albania	2009	Demographic and Health Survey	2	3362	967	5005
Algeria	1992	Pan Arab Project for Child Development (PAPCHILD)	2, 3	3236	2344	4222
Algeria	2003	Pan Arab Population and Family Health Project (PAPFAM)	2, 3	4581	3088	6087
Armenia	2000	Demographic and Health Survey	1	2959	704	4456
Armenia	2005	Demographic and Health Survey	1	2628	578	4430
Azerbaijan	2006	Demographic and Health Survey	2	3401	936	5917
Bangladesh	1975	World Fertility Survey	2, 3	3845	2948	4641
Bangladesh	1993	Demographic and Health Survey	1, 3	6508	3572	7581
Bangladesh	1996	Demographic and Health Survey	1, 3	5987	3075	7060
Bangladesh	1999	Demographic and Health Survey	1, 3	6709	3244	8077
Bangladesh	2004	Demographic and Health Survey	1, 3	7250	3401	8662
Bangladesh	2007	Demographic and Health Survey	2, 3	7151	3253	8584
Belgium	1991	Fertility and Family Survey	2	1603	454	3234
Belize	1991	Reproductive Health Survey	2	1297	733	2160
Benin	1981	World Fertility Survey	2	2132	1743	3187
Benin	1996	Demographic and Health Survey	2	2931	2453	3982
Benin	2001	Demographic and Health Survey	2	3112	2544	4555
Benin	2006	Demographic and Health Survey	2	9497	7763	13335
Bolivia	1989	Demographic and Health Survey	2	3459	2412	5566
Bolivia	1994	Demographic and Health Survey	1	3567	2328	6068
Bolivia	1998	Demographic and Health Survey	2	4593	2869	7746
Bolivia	2003	Demographic and Health Survey	2	7285	4299	12314
Bolivia	2008	Demographic and Health Survey	2	6775	3639	11834
Brazil	1986	Demographic and Health Survey	2	2608	1373	4582
Brazil	1991	Demographic and Health Survey	1	2151	1039	4277
Brazil	1996	Demographic and Health Survey	1	4834	1708	8928
Burkina Faso	1992	Demographic and Health Survey	2	3520	2885	4507
Burkina Faso	1998	Demographic and Health Survey	2	3516	2948	4493
Burkina Faso	2003	Demographic and Health Survey	2	6650	5501	8623
Burundi	1987	Demographic and Health Survey	2	1819	1574	2953
Cambodia	2000	Demographic and Health Survey	2	6807	4963	10365
Cambodia	2005	Demographic and Health Survey	2	7076	4206	11486
Cambodia	2010	Demographic and Health Survey	2	7461	3964	12779
Cameroon	1978	World Fertility Survey	2	3836	3044	5808
Cameroon	1991	Demographic and Health Survey	2	1890	1541	2704
Cameroon	1998	Demographic and Health Survey	2	2342	1807	3871
Cameroon	2004	Demographic and Health Survey	2	4612	3477	7243
Central African Republic	1994	Demographic and Health Survey	2	2829	2278	4171
Chad	1996	Demographic and Health Survey	2	4010	3398	5197
Chad	2004	Demographic and Health Survey	2	3138	2674	4089

Country	Year	Survey	Notes	Sample Size (Primary)*	Sample Size (Secondary)*	Survey Size**
China (Gansu)	1987	In-depth Fertility Sample Surveys	2, 4, 5	3765	--	4951
China (Guangdong)	1987	In-depth Fertility Sample Surveys	2, 4, 5	4279	--	5821
China (Guizhou)	1987	In-depth Fertility Sample Surveys	2, 4, 5	4270	--	5520
China (Hebei)	1985	In-depth Fertility Sample Surveys	2, 4, 5	3306	--	4470
China (Liaoning)	1987	In-depth Fertility Sample Surveys	2, 4, 5	4396	--	5848
China (Shaanxi)	1985	In-depth Fertility Sample Surveys	2, 4, 5	2610	--	3524
China (Shanghai)	1985	In-depth Fertility Sample Surveys	2, 4, 5	2258	--	3635
Colombia	1976	World Fertility Survey	2	1731	1123	3621
Colombia	1986	Demographic and Health Survey	2	1881	986	3731
Colombia	1990	Demographic and Health Survey	1	2853	1220	6250
Colombia	1995	Demographic and Health Survey	1	3707	1569	8011
Colombia	2000	Demographic and Health Survey	1	3635	1364	8244
Colombia	2005	Demographic and Health Survey	1	11704	4304	26924
Colombia	2010	Demographic and Health Survey	2	17888	6753	34441
Comoros	1996	Demographic and Health Survey	2	1157	880	1997
Congo	2005	Demographic and Health Survey	2	2611	2018	5072
Costa Rica	1976	World Fertility Survey	2	1692	948	3473
Czech Republic	1997	Fertility and Family Survey	2	913	259	1489
Côte d'Ivoire	1980	World Fertility Survey	2	2661	2276	4145
Côte d'Ivoire	1994	Demographic and Health Survey	2	3614	3049	5619
Côte d'Ivoire	1998	Demographic and Health Survey	2	1112	902	2075
Democratic Republic of the Congo	2002	Demographic and Health Survey	1	8750	3471	16438
Democratic Republic of the Congo	2007	Demographic and Health Survey	2	4419	3610	7226
Denmark	1992	European Studies of Infertility and Subfecundity	1, 5	512	105	1028
Djibouti	2003	Pan Arab Population and Family Health Project (PAPFAM)	2, 3	1708	1402	2425
Dominican Republic	1975	World Fertility Survey	2	1037	753	2103
Dominican Republic	1986	Demographic and Health Survey	2	2927	1665	5119
Dominican Republic	1991	Demographic and Health Survey	1	2473	1145	5110
Dominican Republic	1996	Demographic and Health Survey	1	3097	1359	5954
Dominican Republic	1999	Demographic and Health Survey	1	458	179	903
Dominican Republic	2007	Demographic and Health Survey	2	11179	4476	18739
Ecuador	1987	Demographic and Health Survey	2	2069	1273	3381

Country	Year	Survey	Notes	Sample Size (Primary)*	Sample Size (Secondary)*	Survey Size**
Ecuador	1989	Reproductive Health Survey	2	2949	1662	5812
Ecuador	1999	Reproductive Health Survey	2	6788	3484	10947
Ecuador	2004	Reproductive Health Survey	2	4940	2349	8213
Egypt	1980	World Fertility Survey	2, 3	5227	3753	7234
Egypt	1988	Demographic and Health Survey	2, 3	5666	3613	7464
Egypt	1991	Pan Arab Project for Child Development (PAPCHILD)	2, 3	5864	3790	7576
Egypt	1992	Demographic and Health Survey	2, 3	6459	3944	8321
Egypt	1995	Demographic and Health Survey	1, 3	9220	5281	12100
Egypt	2000	Demographic and Health Survey	1, 3	9307	4980	12702
Egypt	2005	Demographic and Health Survey	1, 3	11548	5877	15990
Egypt	2008	Demographic and Health Survey	1, 3	9627	4611	13635
El Salvador	1985	Demographic and Health Survey	2	2080	1144	3545
El Salvador	1998	Reproductive Health Survey	2	5712	2984	9455
El Salvador	2002	Reproductive Health Survey	2	3983	2000	8034
El Salvador	2008	Reproductive Health Survey	2	4290	1909	9050
Ethiopia	2000	Demographic and Health Survey	2	6627	5051	10548
Ethiopia	2005	Demographic and Health Survey	1	5860	4507	9738
Ethiopia	2011	Demographic and Health Survey	2	7274	5425	11579
Fiji	1974	World Fertility Survey	2, 3	3104	1985	4263
Finland	1989	Fertility and Family Survey	2	3384	926	6816
Gabon	2000	Demographic and Health Survey	2	2263	1733	4219
Germany	1992	European Studies of Infertility and Subfecundity	1, 5	921	233	1485
Ghana	1988	Demographic and Health Survey	2	2224	1855	3273
Ghana	1993	Demographic and Health Survey	2	2293	1829	3423
Ghana	1998	Demographic and Health Survey	2	2209	1696	3516
Ghana	2003	Demographic and Health Survey	2	2564	1971	4077
Ghana	2008	Demographic and Health Survey	2	2041	1541	3444
Guatemala	1987	Demographic and Health Survey	2	2655	1978	3978
Guatemala	1995	Demographic and Health Survey	1	5575	4013	8411
Guinea	1999	Demographic and Health Survey	2	3950	3326	4852
Guinea	2005	Demographic and Health Survey	2	4503	3751	5509
Guyana	1975	World Fertility Survey	2	1682	1038	3314
Haiti	1977	World Fertility Survey	2	839	662	2332
Haiti	1994	Demographic and Health Survey	2	2042	1493	3662
Haiti	2000	Demographic and Health Survey	2	3791	2729	6758
Haiti	2005	Demographic and Health Survey	2	4083	2634	7053
Honduras	1996	Reproductive Health Survey	2	3633	2441	5600
Honduras	2001	Reproductive Health Survey	2	4094	2442	6349
Honduras	2005	Demographic and Health Survey	2	8198	4642	13790
India	1992	Demographic and Health Survey	2, 3	59062	27172	73184
India	1998	Demographic and Health Survey	2, 3	58944	24941	74715
India	2005	Demographic and Health Survey	1	62785	21778	90623
Indonesia	1976	World Fertility Survey	2, 3	5226	3849	7381

Country	Year	Survey	Notes	Sample Size (Primary)*	Sample Size (Secondary)*	Survey Size**
Indonesia	1987	Demographic and Health Survey	2, 3	7777	4195	9860
Indonesia	1991	Demographic and Health Survey	1, 3	14682	7761	19133
Indonesia	1994	Demographic and Health Survey	1, 3	18359	9522	23834
Indonesia	1997	Demographic and Health Survey	1, 3	18442	8935	24249
Indonesia	2002	Demographic and Health Survey	1, 3	18565	8322	24464
Indonesia	2007	Demographic and Health Survey	1, 3	20859	9836	27575
Italy	1992	European Studies of Infertility and Subfecundity	1, 5	1667	312	2724
Italy	1995	Fertility and Family Survey	2	1710	417	4109
Jamaica	1975	World Fertility Survey	2	925	608	2296
Jordan	1990	Demographic and Health Survey	2, 3	4062	3110	5363
Jordan	1997	Demographic and Health Survey	1, 3	3579	2446	4785
Jordan	2002	Demographic and Health Survey	2, 3	4004	2779	5223
Jordan	2007	Demographic and Health Survey	2, 3	7103	4705	9409
Jordan	2009	Demographic and Health Survey	2, 3	6353	4213	8693
Kazakhstan	1995	Demographic and Health Survey	2	1668	606	2741
Kazakhstan	1999	Demographic and Health Survey	1	2034	571	3489
Kenya	1977	World Fertility Survey	2	3679	3269	5645
Kenya	1989	Demographic and Health Survey	2	3371	2673	5242
Kenya	1993	Demographic and Health Survey	2	3142	2385	5312
Kenya	1998	Demographic and Health Survey	1	3200	2125	5505
Kenya	2003	Demographic and Health Survey	1	3142	2134	5865
Kenya	2008	Demographic and Health Survey	2	3368	2416	6007
Kyrgyzstan	1997	Demographic and Health Survey	2	1856	907	2789
Lebanon	1996	Pan Arab Project for Child Development (PAPCHILD)	2, 3	2018	1058	2579
Lebanon	2004	Pan Arab Population and Family Health Project (PAPFAM)	2, 3	1955	877	2441
Lesotho	1977	World Fertility Survey	2, 3	1974	1587	2944
Lesotho	2004	Demographic and Health Survey	2	2305	1462	4743
Lesotho	2009	Demographic and Health Survey	2	2416	1396	5138
Liberia	1986	Demographic and Health Survey	2	2457	2052	3664
Liberia	2006	Demographic and Health Survey	2	3220	2596	5148
Libyan Arab Jamahiriya	1995	Pan Arab Project for Child Development (PAPCHILD)	2, 3	3036	2404	3938
Lithuania	1995	Fertility and Family Survey	2	1134	367	2293
Madagascar	1992	Demographic and Health Survey	2	2508	1915	4498
Madagascar	1997	Demographic and Health Survey	2	2948	2251	5008
Madagascar	2003	Demographic and Health Survey	2	3392	2280	5761
Madagascar	2008	Demographic and Health Survey	2	7935	5363	11917
Malawi	1992	Demographic and Health Survey	2	2422	2019	3430
Malawi	2000	Demographic and Health Survey	2	6068	4684	9392
Malawi	2004	Demographic and Health Survey	1	5088	3883	8521
Malaysia	1974	World Fertility Survey	2, 3	3723	2650	5139
Maldives	2009	Demographic and Health Survey	2	3996	2045	6266

Country	Year	Survey	Notes	Sample Size (Primary)*	Sample Size (Secondary)*	Survey Size**
Mali	1987	Demographic and Health Survey	2	2109	1688	2407
Mali	1995	Demographic and Health Survey	2	5940	4922	7043
Mali	2001	Demographic and Health Survey	2	7661	6321	9347
Mali	2006	Demographic and Health Survey	2	8518	6975	10325
Mauritania	1981	World Fertility Survey	2, 3	1827	1492	2725
Mauritania	1990	Pan Arab Project for Child Development (PAPCHILD)	2, 3	2989	2454	4275
Mexico	1976	World Fertility Survey	2	3562	2794	6150
Mexico	1987	Demographic and Health Survey	2	3724	2199	6391
Moldova	1997	Reproductive Health Survey	1	3177	796	4685
Morocco	1980	World Fertility Survey	2	2375	1864	3878
Morocco	1987	Demographic and Health Survey	2, 3	3814	2677	4927
Morocco	1992	Demographic and Health Survey	1	3510	2369	6443
Morocco	1997	Pan Arab Project for Child Development (PAPCHILD)	2, 3	3116	1927	4190
Morocco	2003	Demographic and Health Survey	2	5832	3317	11760
Mozambique	1997	Demographic and Health Survey	2	4142	3319	6249
Mozambique	2003	Demographic and Health Survey	2	5724	4592	8860
Namibia	1992	Demographic and Health Survey	2	1414	1102	3781
Namibia	2000	Demographic and Health Survey	2	1698	1041	4878
Namibia	2006	Demographic and Health Survey	2	2124	1302	6886
Nepal	1976	World Fertility Survey	2, 3	3884	2717	4732
Nepal	1996	Demographic and Health Survey	2, 3	5604	3331	6648
Nepal	2001	Demographic and Health Survey	2, 3	5863	3152	6960
Nepal	2006	Demographic and Health Survey	2	5799	2606	7489
Nepal	2011	Demographic and Health Survey	2	6674	2491	8934
Nicaragua	1997	Demographic and Health Survey	1	5364	2952	9338
Nicaragua	2001	Demographic and Health Survey	2	5369	2665	8886
Niger	1992	Demographic and Health Survey	2	3914	3258	4703
Niger	1998	Demographic and Health Survey	2	4333	3653	5296
Niger	2006	Demographic and Health Survey	2	5349	4539	6713
Nigeria	1990	Demographic and Health Survey	2	4781	3865	6501
Nigeria	1999	Demographic and Health Survey	2	3914	3007	5863
Nigeria	2003	Demographic and Health Survey	2	3425	2760	5253
Nigeria	2008	Demographic and Health Survey	2	15988	13023	23889
Pakistan	1975	World Fertility Survey	2, 3	3149	2422	3856
Pakistan	1990	Demographic and Health Survey	2, 3	4532	3333	5584
Pakistan	2006	Demographic and Health Survey	2, 3	6387	4016	8217
Panama	1975	World Fertility Survey	2	1723	1060	3297
Paraguay	1979	World Fertility Survey	2	1532	1057	3160
Paraguay	1990	Demographic and Health Survey	1	2355	1546	4072
Peru	1977	World Fertility Survey	2, 3	3195	2453	4570
Peru	1986	Demographic and Health Survey	2	1988	1241	3506
Peru	1992	Demographic and Health Survey	1	6132	3510	11220
Peru	1996	Demographic and Health Survey	1	11960	6818	20678

Country	Year	Survey	Notes	Sample Size (Primary)*	Sample Size (Secondary)*	Survey Size**
Peru	2000	Demographic and Health Survey	1	11101	5525	19673
Philippines	1978	World Fertility Survey	2, 3	5873	4327	7727
Philippines	1993	Demographic and Health Survey	1	6263	3821	10746
Philippines	1998	Demographic and Health Survey	1	5713	3388	9819
Philippines	2003	Demographic and Health Survey	1	5646	3009	9644
Philippines	2008	Demographic and Health Survey	2	5511	2996	9383
Poland	1991	Fertility and Family Survey	2	2033	585	3430
Poland	1992	European Studies of Infertility and Subfecundity	1, 5	337	64	442
Portugal	1979	World Fertility Survey	2, 3	2931	1030	4071
Republic of Korea	1974	World Fertility Survey	2, 3	3232	2084	4627
Republic of Moldova	2005	Demographic and Health Survey	1	2976	628	4887
Rwanda	1992	Demographic and Health Survey	2	2546	2151	4699
Rwanda	2000	Demographic and Health Survey	2	3265	2610	6967
Rwanda	2005	Demographic and Health Survey	2	3717	3131	7821
Rwanda	2010	Demographic and Health Survey	2	4435	3337	9606
Sao Tome and Principe	2008	Demographic and Health Survey	2	1258	995	1863
Senegal	1978	World Fertility Survey	2	1967	1724	2882
Senegal	1986	Demographic and Health Survey	2	2385	1980	3169
Senegal	1992	Demographic and Health Survey	2	3292	2746	4513
Senegal	1997	Demographic and Health Survey	2	4231	3577	6105
Senegal	2005	Demographic and Health Survey	2	6644	5253	9933
Sierra Leone	2008	Demographic and Health Survey	2	3909	2978	5591
Slovenia	1995	Fertility and Family Survey	2	1681	383	2498
South Africa	1998	Demographic and Health Survey	2	3240	1719	8396
Spain	1992	European Studies of Infertility and Subfecundity	1, 5	562	107	857
Spain	1995	Fertility and Family Survey	2	1745	482	3311
Sri Lanka	1987	Demographic and Health Survey	2, 3	3733	1759	5092
Sudan	1978	World Fertility Survey	2, 3	2080	1728	2653
Sudan	1990	Demographic and Health Survey	2, 3	3740	3040	4940
Sudan	1993	Pan Arab Project for Child Development (PAPCHILD)	2, 3	2876	2358	3751
Swaziland	2006	Demographic and Health Survey	2	1207	767	3339
Syrian Arab Republic	1978	World Fertility Survey	2, 3	2755	2143	3543
Syrian Arab Republic	1993	Pan Arab Project for Child Development (PAPCHILD)	2, 3	3033	2080	3810
Syrian Arab Republic	2002	Pan Arab Population and Family Health Project (PAPFAM)	2, 3	4715	2991	5807
Thailand	1987	Demographic and Health Survey	2, 3	4159	1620	5686
Timor-Leste	2009	Demographic and Health Survey	2	5462	4270	8758

Country	Year	Survey	Notes	Sample Size (Primary)*	Sample Size (Secondary)*	Survey Size**
Togo	1988	Demographic and Health Survey	2	1701	1444	2393
Togo	1998	Demographic and Health Survey	2	4303	3539	6159
Trinidad and Tobago	1977	World Fertility Survey	2	1470	738	3336
Trinidad and Tobago	1987	Demographic and Health Survey	2	1508	772	2844
Tunisia	1988	Demographic and Health Survey	2, 3	2762	1762	3678
Tunisia	1994	Pan Arab Project for Child Development (PAPCHILD)	2, 3	2921	1833	3763
Tunisia	2002	Pan Arab Population and Family Health Project (PAPFAM)	2, 3	2443	1321	3199
Turkey	1978	World Fertility Survey	2, 3	2860	1754	3578
Turkey	1993	Demographic and Health Survey	1, 3	4308	1593	5514
Turkey	1998	Demographic and Health Survey	1	4023	1541	6101
Turkey	2003	Demographic and Health Survey	1, 3	2832	1032	6809
Uganda	1988	Demographic and Health Survey	2	2009	1714	3244
Uganda	1995	Demographic and Health Survey	2	3177	2538	5095
Uganda	2000	Demographic and Health Survey	2	3187	2497	5153
Uganda	2006	Demographic and Health Survey	2	3821	3132	5981
Ukraine	1999	Reproductive Health Survey	1	3627	589	6045
Ukraine	2007	Demographic and Health Survey	2	2718	614	4929
United Republic of Tanzania	1992	Demographic and Health Survey	2	3920	3300	6310
United Republic of Tanzania	1996	Demographic and Health Survey	2	3643	2990	5810
United Republic of Tanzania	1999	Demographic and Health Survey	2	1771	1413	2807
United Republic of Tanzania	2004	Demographic and Health Survey	1	4174	3240	7281
United Republic of Tanzania	2010	Demographic and Health Survey	2	4379	3519	7094
United States of America	1988	National Survey of Family and Growth	1	2680	729	7199
United States of America	1995	National Survey of Family and Growth	1	3487	924	9421
United States of America	2002	National Survey of Family and Growth	1	2047	666	6488
United States of America	2007	National Survey of Family and Growth	1	1770	609	6006
Uzbekistan	1996	Demographic and Health Survey	2	2056	983	3148
Venezuela (Bolivarian Republic of)	1977	World Fertility Survey	2	1469	928	3130
Viet Nam	1997	Demographic and Health Survey	1, 3	3767	1408	4937
Viet Nam	2002	Demographic and Health Survey	1, 3	3794	1011	4777

Country	Year	Survey	Notes	Sample Size (Primary)*	Sample Size (Secondary)*	Survey Size**
Yemen	1991	Pan Arab Project for Child Development (PAPCHILD)	2, 3	3925	3145	4733
Yemen	2003	Pan Arab Population and Family Health Project (PAPFAM)	2, 3	7580	5644	9334
Zambia	1992	Demographic and Health Survey	2	2992	2528	4706
Zambia	1996	Demographic and Health Survey	2	3272	2730	5523
Zambia	2001	Demographic and Health Survey	2	3187	2573	5365
Zambia	2007	Demographic and Health Survey	2	3032	2460	5086
Zimbabwe	1988	Demographic and Health Survey	2	1798	1499	2890
Zimbabwe	1994	Demographic and Health Survey	1	2335	1697	4226
Zimbabwe	1999	Demographic and Health Survey	1	2109	1391	4057
Zimbabwe	2005	Demographic and Health Survey	1	3164	1963	6158
Zimbabwe	2010	Demographic and Health Survey	2	3494	2379	6559

* Sample size refers to the proportion of women who were included in the calculations of primary or secondary infertility (i.e., the denominator in th eprevalence calculation)

** Survey size refers to the total number of women aged 15-44 surveyed.

Notes: 1 Information on past contraceptive use and past couple status were available

2 Data on current use of contraception and couple status were available, but data on past use and status were incomplete

3 Survey excluded never-married women

4 Data on secondary infertility from China were not used

5 Survey is representative of a subnational area

**Primary prevalence**	
Median relative error	0.345 (0.029)
Root mean square error	0.013 (0.001)
Fraction of data falling within 95% credible interval	0.926 (0.041)
**Secondary prevalence**	
Median relative error	0.326 (0.035)
Root mean square error	0.061 (0.007)
Fraction of data falling within 95% credible interval	0.943 (0.017)
**Primary exposure**	
Median relative error	0.061 (0.012)
Root mean square error	0.091 (0.013)
Fraction of data falling within 95% credible interval	0.961 (0.038)
**Secondary exposure**	
Median relative error	0.167 (0.057)
Root mean square error	0.131 (0.045)
Fraction of data falling within 95% credible interval	0.943 (0.033)

Table C: Crossvalidation statistics with standard errors: 10-fold crossvalidation was performed with replacement, where each sample left out 20% of the countries in the dataset (crossvalidation is described in the main text).

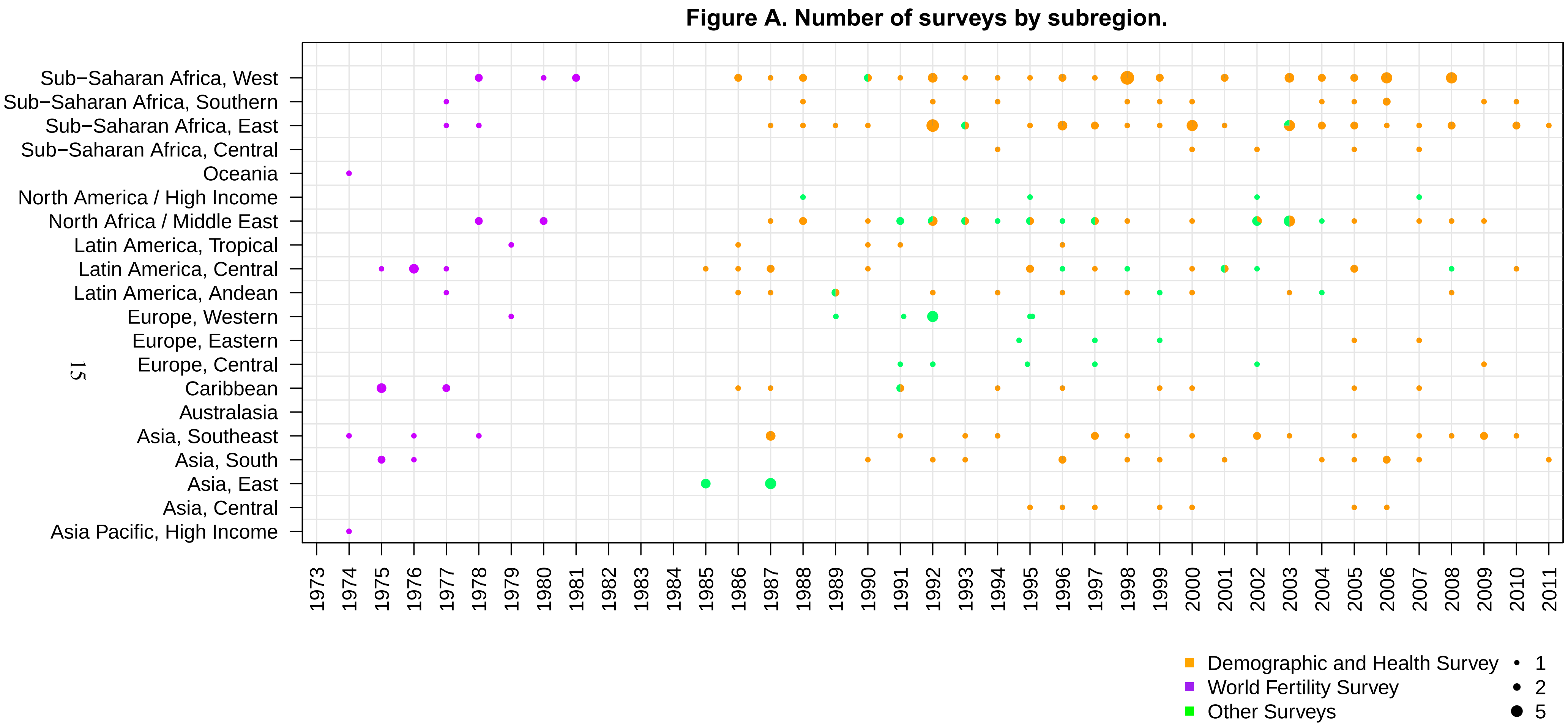
Figure A. Number of surveys by subregion.
Sub−Saharan Africa, West
Sub−Saharan Africa, Southern
Sub−Saharan Africa, East
Sub−Saharan Africa, Central
Oceania
North America / High Income
North Africa / Middle East
Latin America, Tropical
Latin America, Central
Latin America, Andean
Europe, Western
Europe, Eastern
Europe, Central
Caribbean
Australasia
Asia, Southeast
Asia, South
Asia, East
Asia, Central
Asia Pacific, High Income
1973
1974
1975
1976
1977
1978
1979
1980
1981
1982
1983
1984
1985
1986
1987
1988
1989
1990
1991
1992
1993
1994
1995
1996
1997
1998
1999
2000
2001
2002
2003
2004
2005
2006
2007
2008
2009
2010
2011
Demographic and Health Survey
World Fertility Survey
Other Surveys
1
2
5

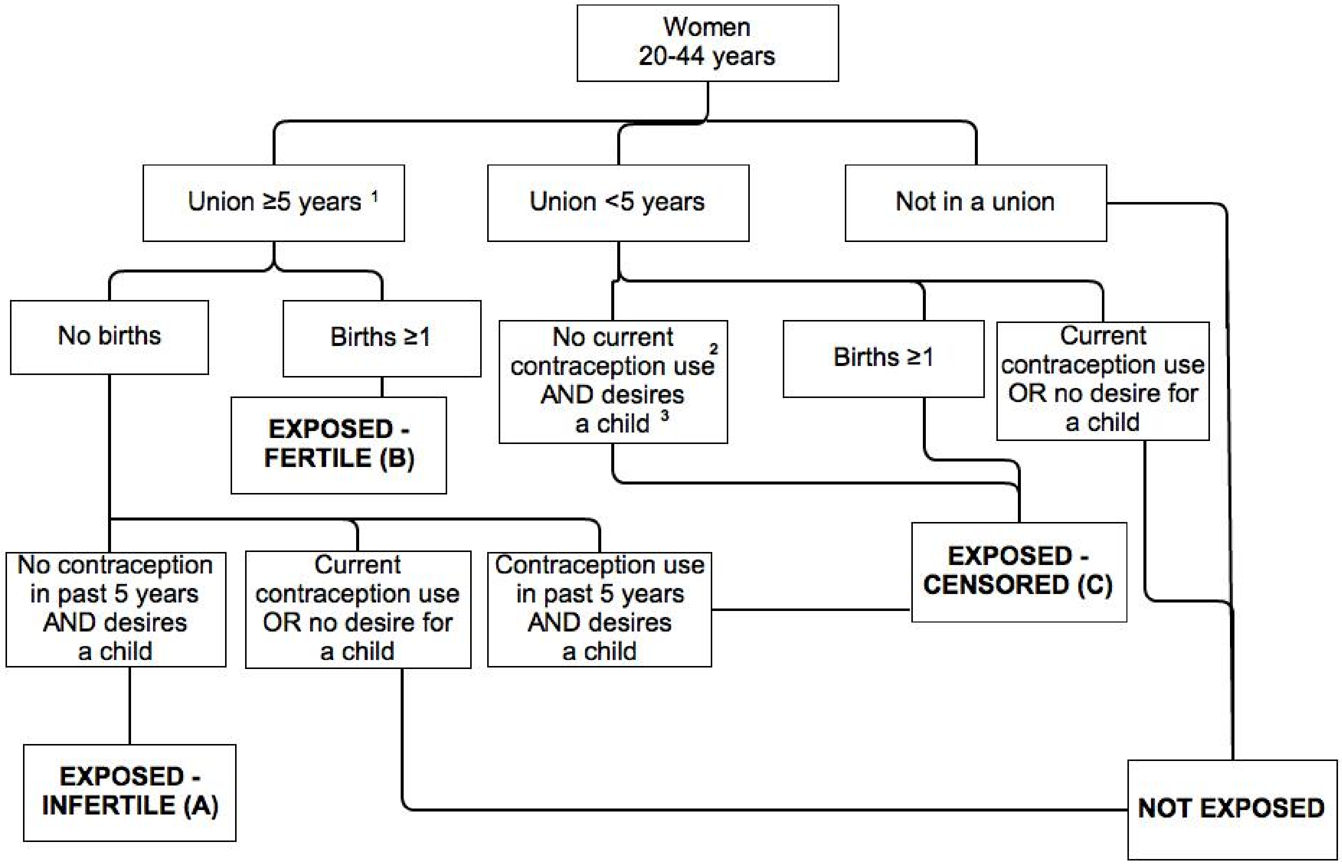

1- Union is defined as marriage or cohabitation
2- Contraception use includes any method used to prevent birth
3- Wants a child, undecided, or declared infecund

Figure B. Primary infertility, women aged 20-44. Primary infertility prevalence is calculated as the number of infertile women (A) divided by the number of women who are both infertile and fertile (A and B). Exposure to primary infertility is calculated as the sum of women who are fertile, infertile and censored (A, B, and C) over all women.

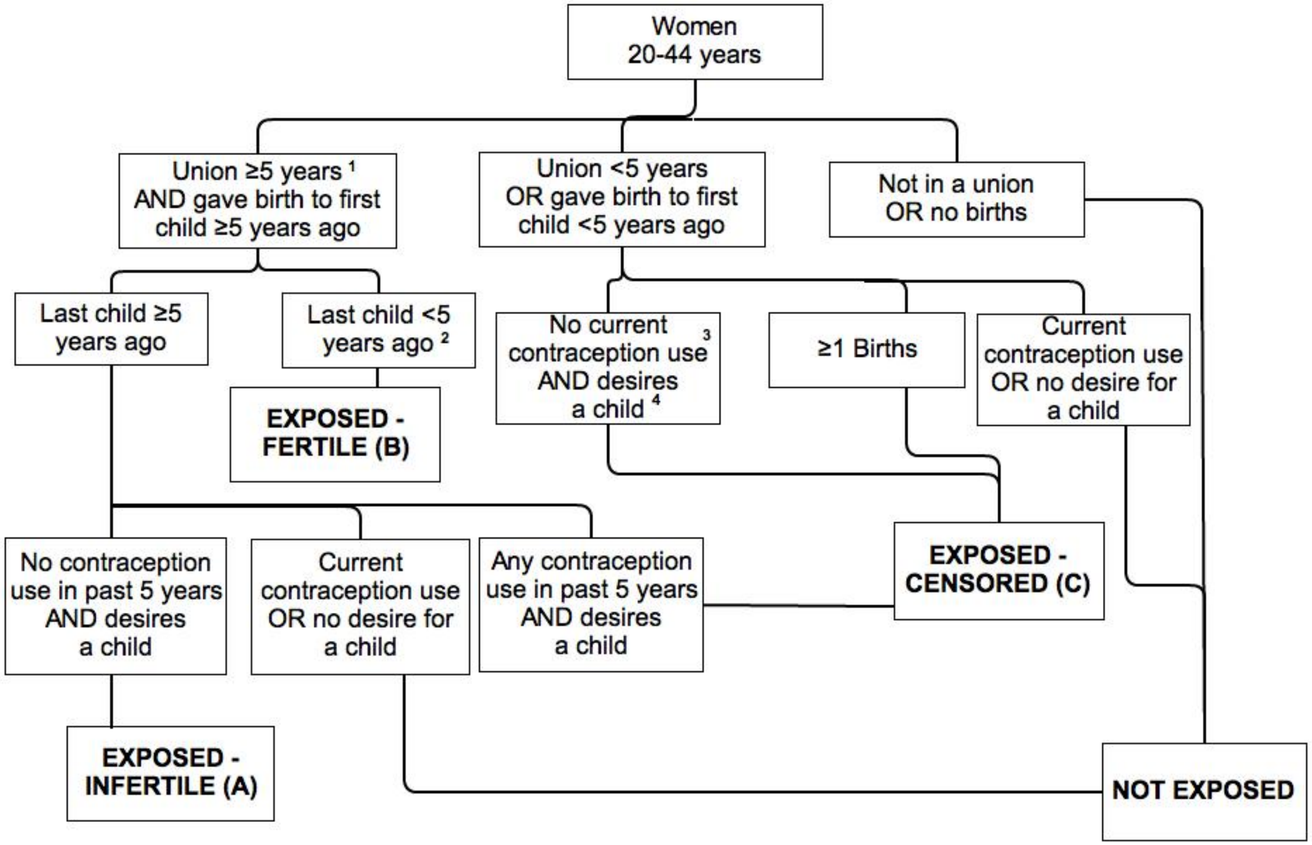

1- Union is defined as marriage or cohabitation
2- Last child refers to the most recent birth
3- Contraception use includes any method used to prevent pregnancy
4- Wants a child, undecided, or declared infecund

Figure C. Secondary infertility, women aged 20-44 years. Secondary infertility prevalence is calculated as the number of infertile women (A) divided by the number of women who are both infertile and fertile (A and B). Exposure to secondary infertility is calculated as the sum of women who are fertile, infertile and censored (A, B, and C) over all women.

Figure D: Prevalence of primary infertility by age group, shown for each country-year of data on following pages. Legend for plots is given below.

- Nationally representative (error bars represent standard errors)
- Subnational
- World Fertility Survey
- Posterior means (model predictions)
- 95% credible interval

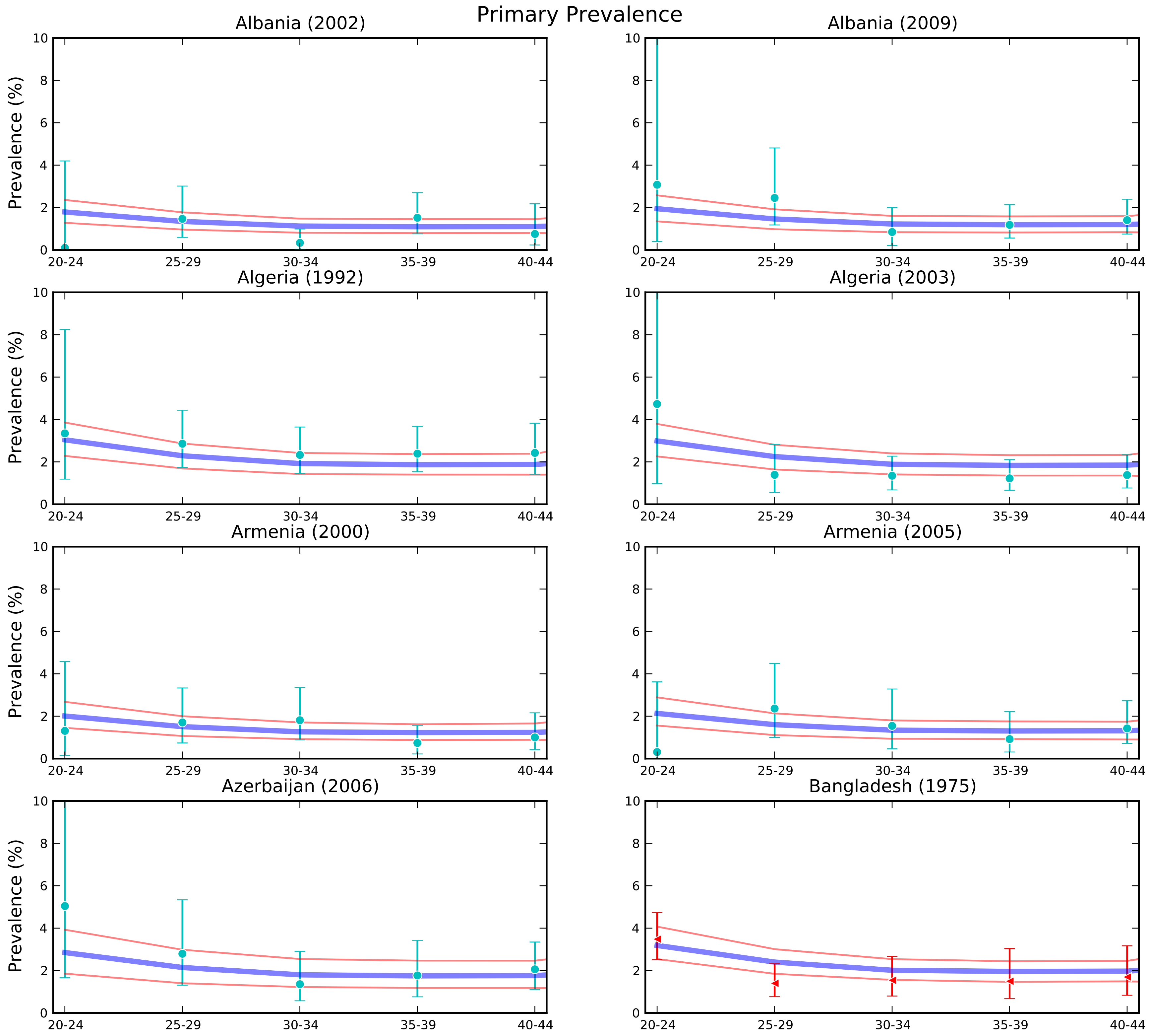
Primary Prevalence
Albania (2002)
Albania (2009)
Algeria (1992)
Algeria (2003)
Armenia (2000)
Armenia (2005)
Azerbaijan (2006)
Bangladesh (1975)
Prevalence (%)
0
2
4
6
8
10
20-24
25-29
30-34
35-39
40-44

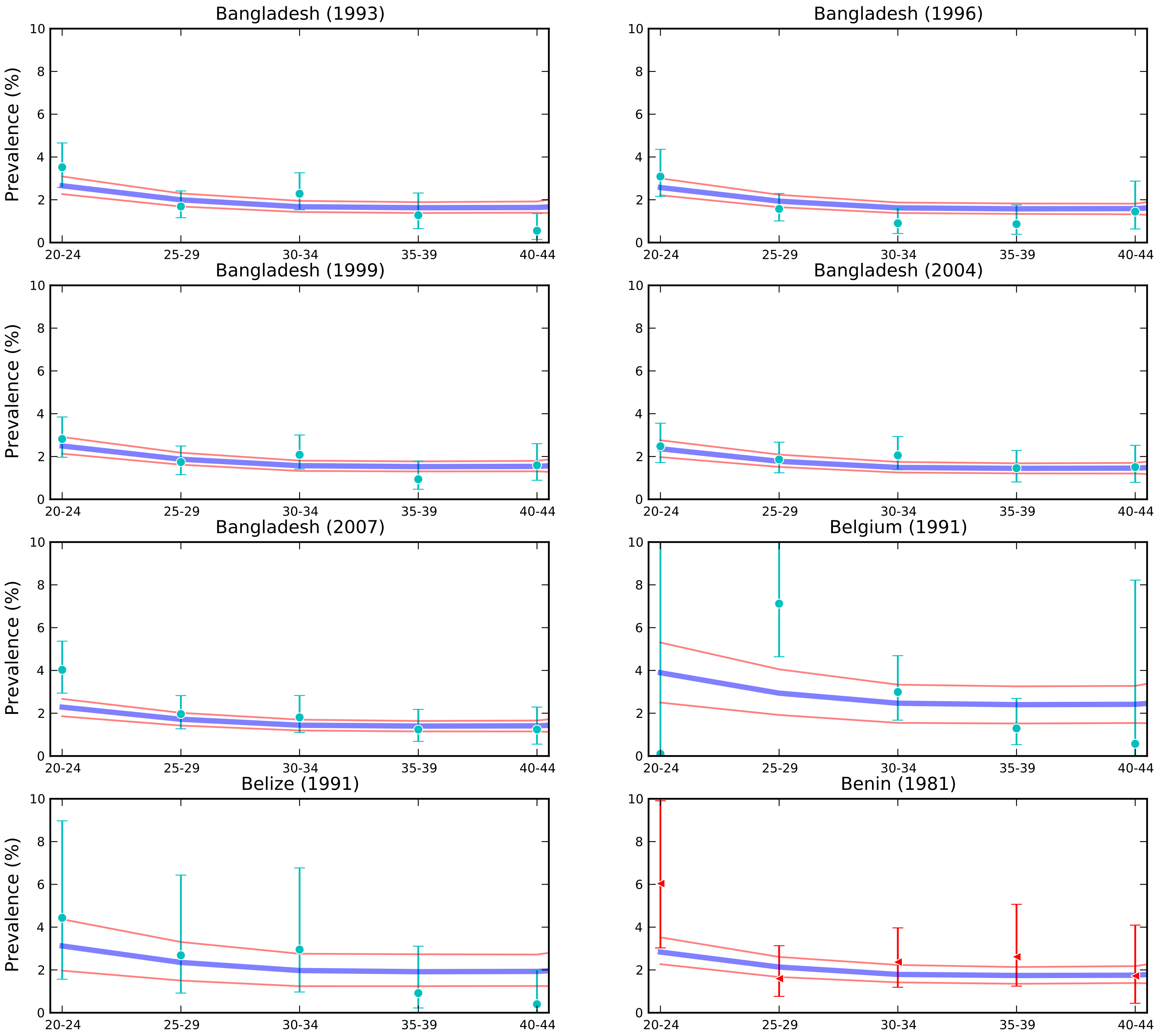
Bangladesh (1993)
Bangladesh (1996)
Bangladesh (1999)
Bangladesh (2004)
Bangladesh (2007)
Belgium (1991)
Belize (1991)
Benin (1981)
Prevalence (%)
0
2
4
6
8
10
20-24
25-29
30-34
35-39
40-44

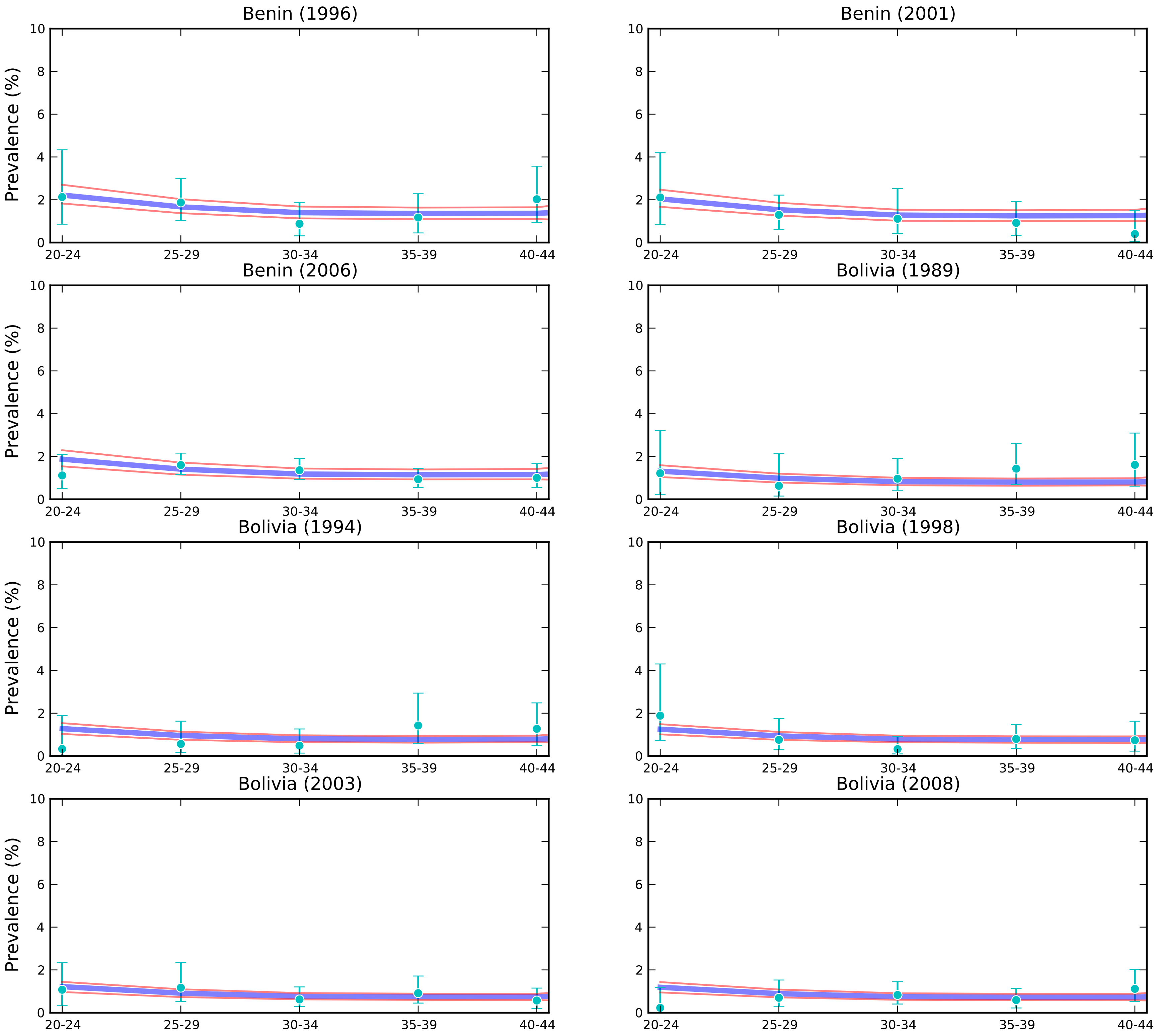
Benin (1996)
Benin (2001)
Benin (2006)
Bolivia (1989)
Bolivia (1994)
Bolivia (1998)
Bolivia (2003)
Bolivia (2008)
Prevalence (%)
0
2
4
6
8
10
20-24
25-29
30-34
35-39
40-44

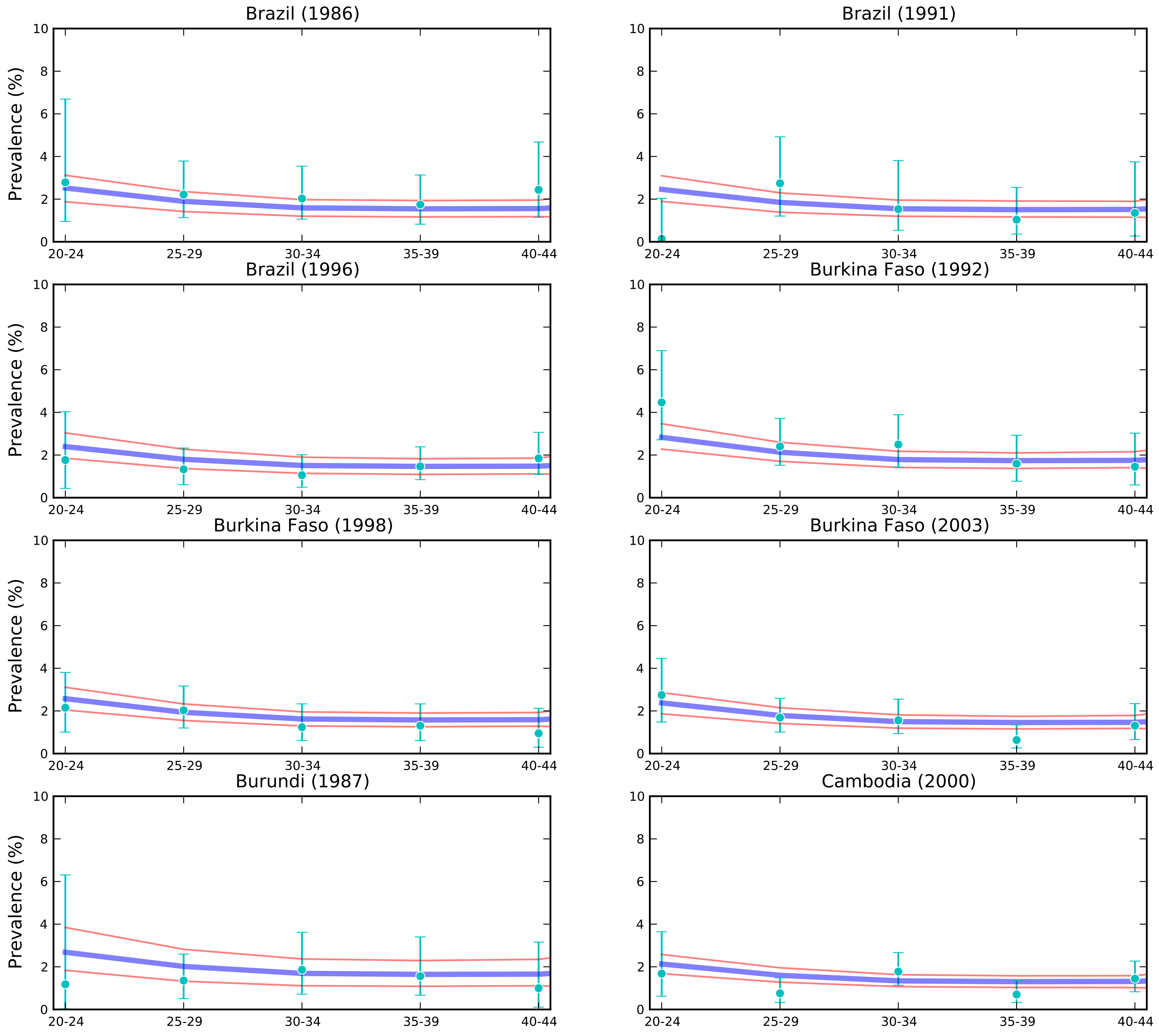
Brazil (1986)
Brazil (1991)
Brazil (1996)
Burkina Faso (1992)
Burkina Faso (1998)
Burkina Faso (2003)
Burundi (1987)
Cambodia (2000)
Prevalence (%)
0
2
4
6
8
10
20-24
25-29
30-34
35-39
40-44

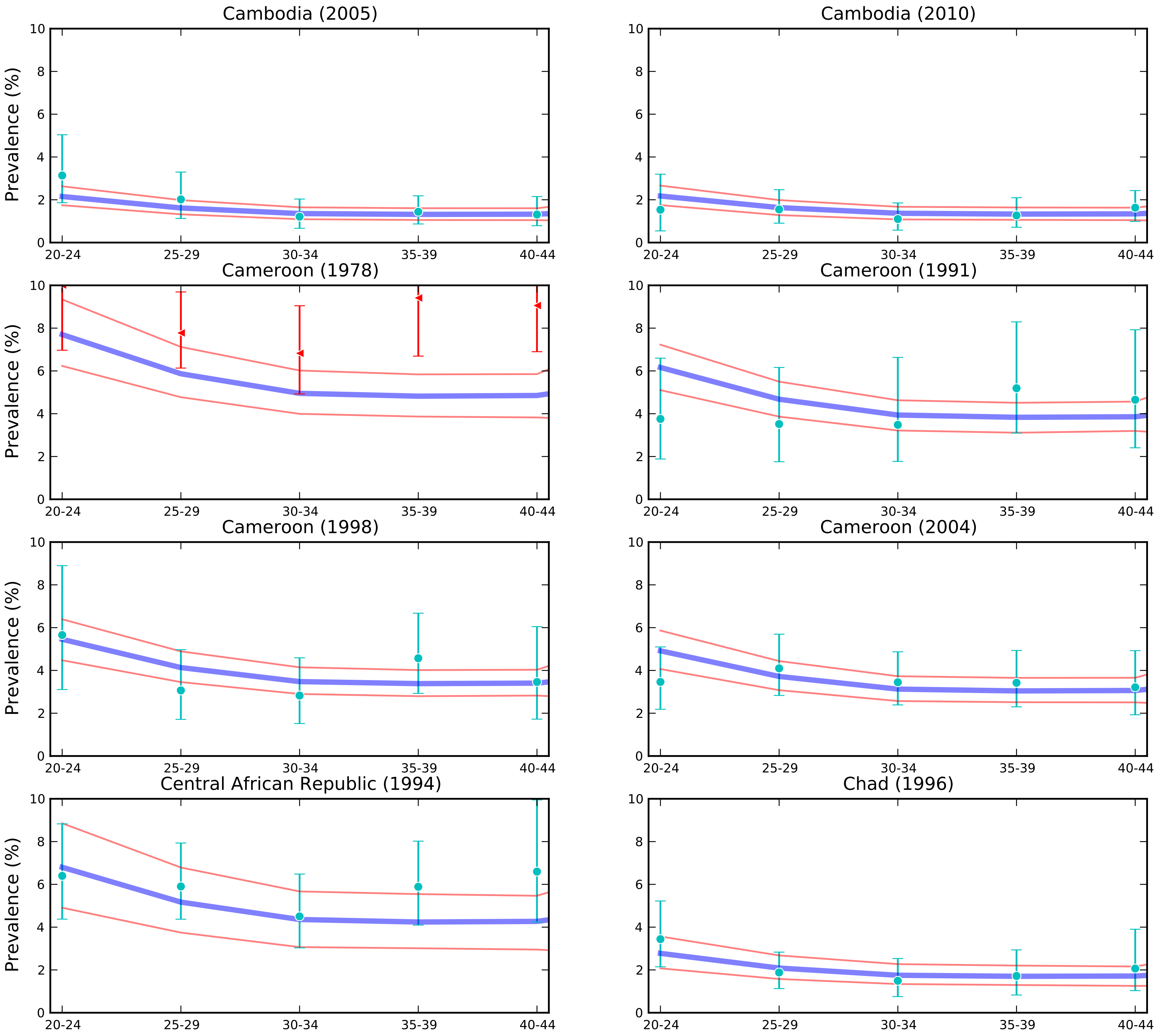
Cambodia (2005)
Cambodia (2010)
Cameroon (1978)
Cameroon (1991)
Cameroon (1998)
Cameroon (2004)
Central African Republic (1994)
Chad (1996)
Prevalence (%)
0
2
4
6
8
10
20-24
25-29
30-34
35-39
40-44

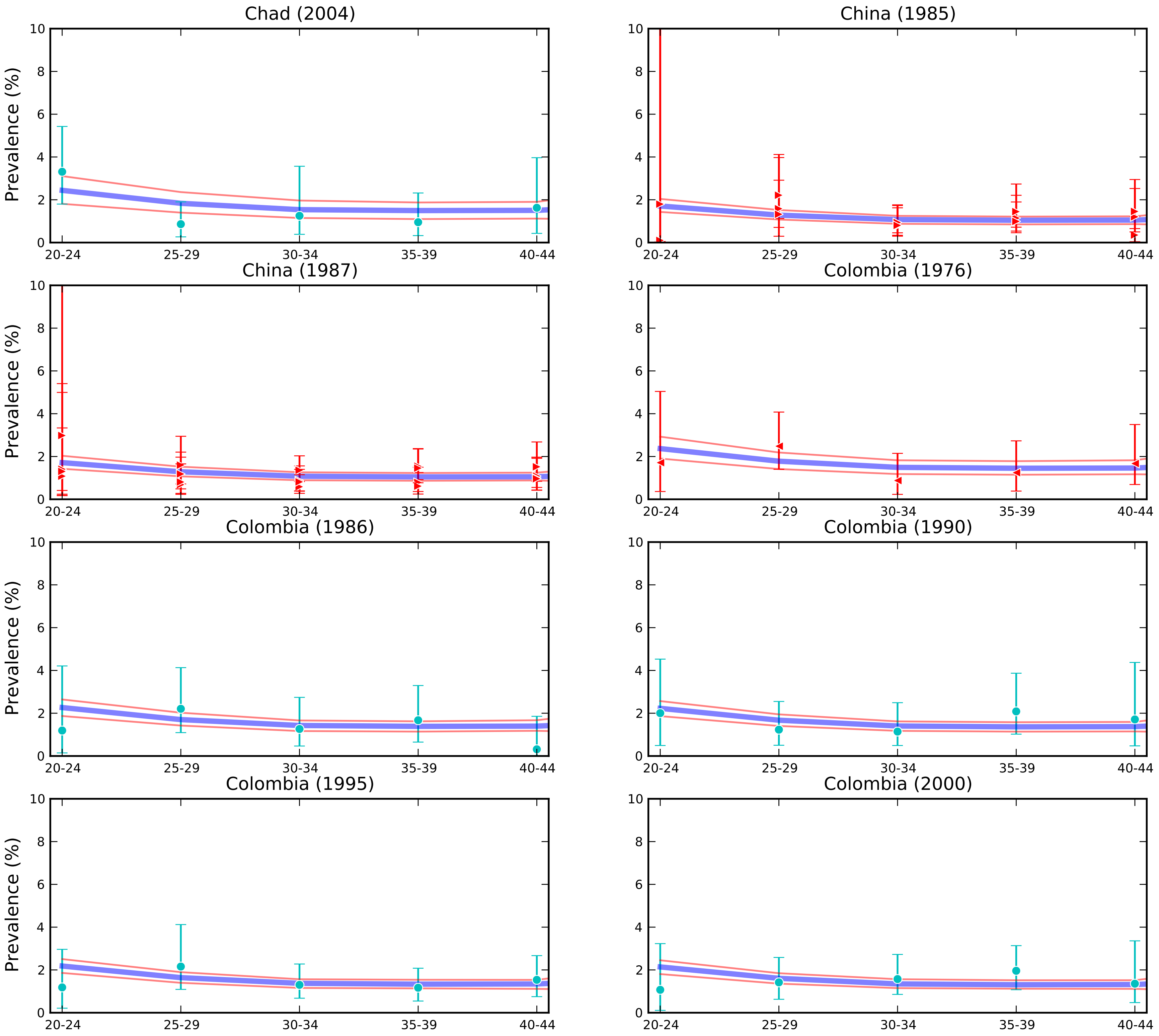
Chad (2004)
China (1985)
China (1987)
Colombia (1976)
Colombia (1986)
Colombia (1990)
Colombia (1995)
Colombia (2000)
Prevalence (%)
0
2
4
6
8
10
20-24
25-29
30-34
35-39
40-44

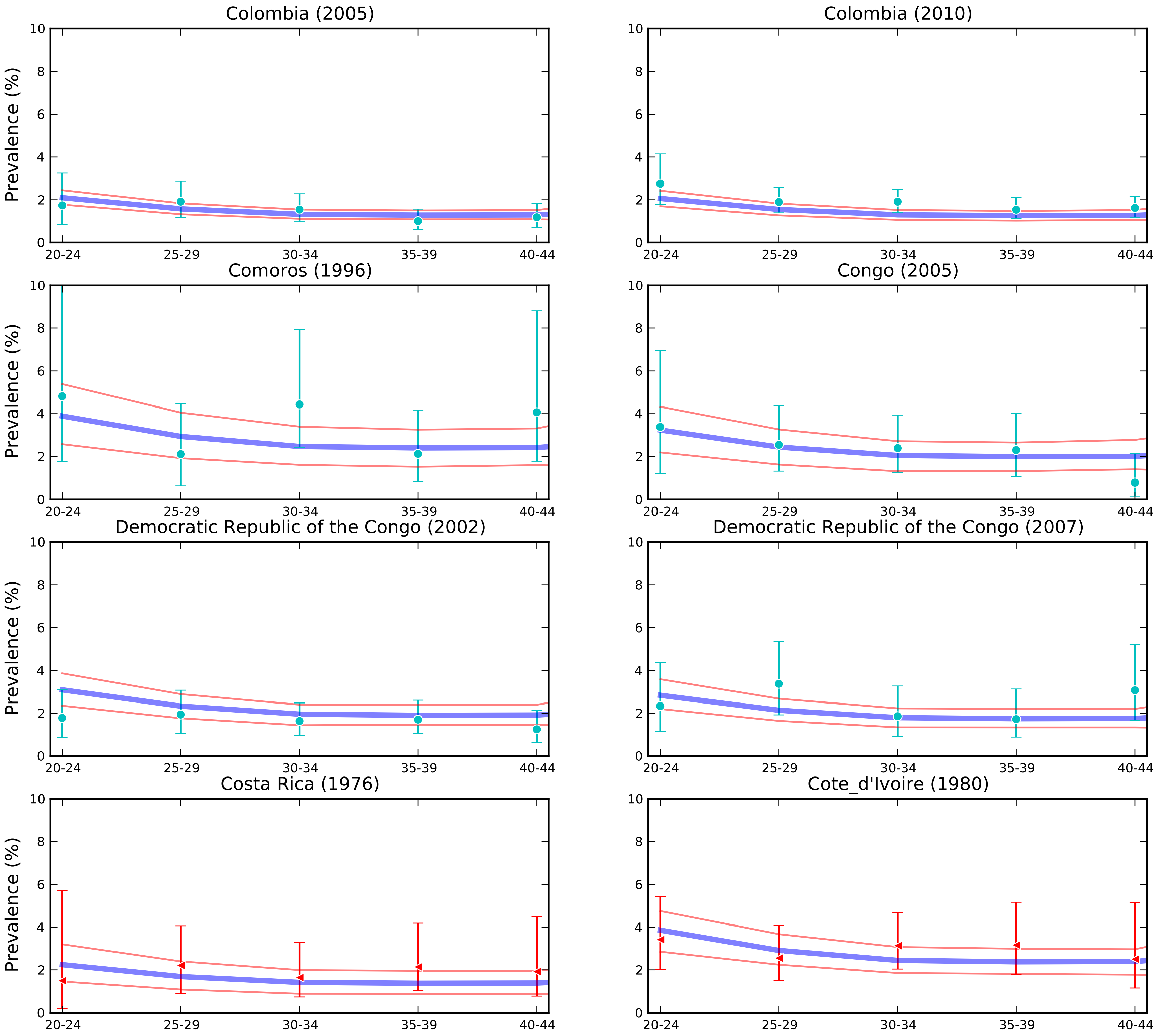

Colombia (2005)
Colombia (2010)
Comoros (1996)
Congo (2005)
Democratic Republic of the Congo (2002)
Democratic Republic of the Congo (2007)
Costa Rica (1976)
Cote_d'Ivoire (1980)
Prevalence (%)
0
2
4
6
8
10
20-24
25-29
30-34
35-39
40-44

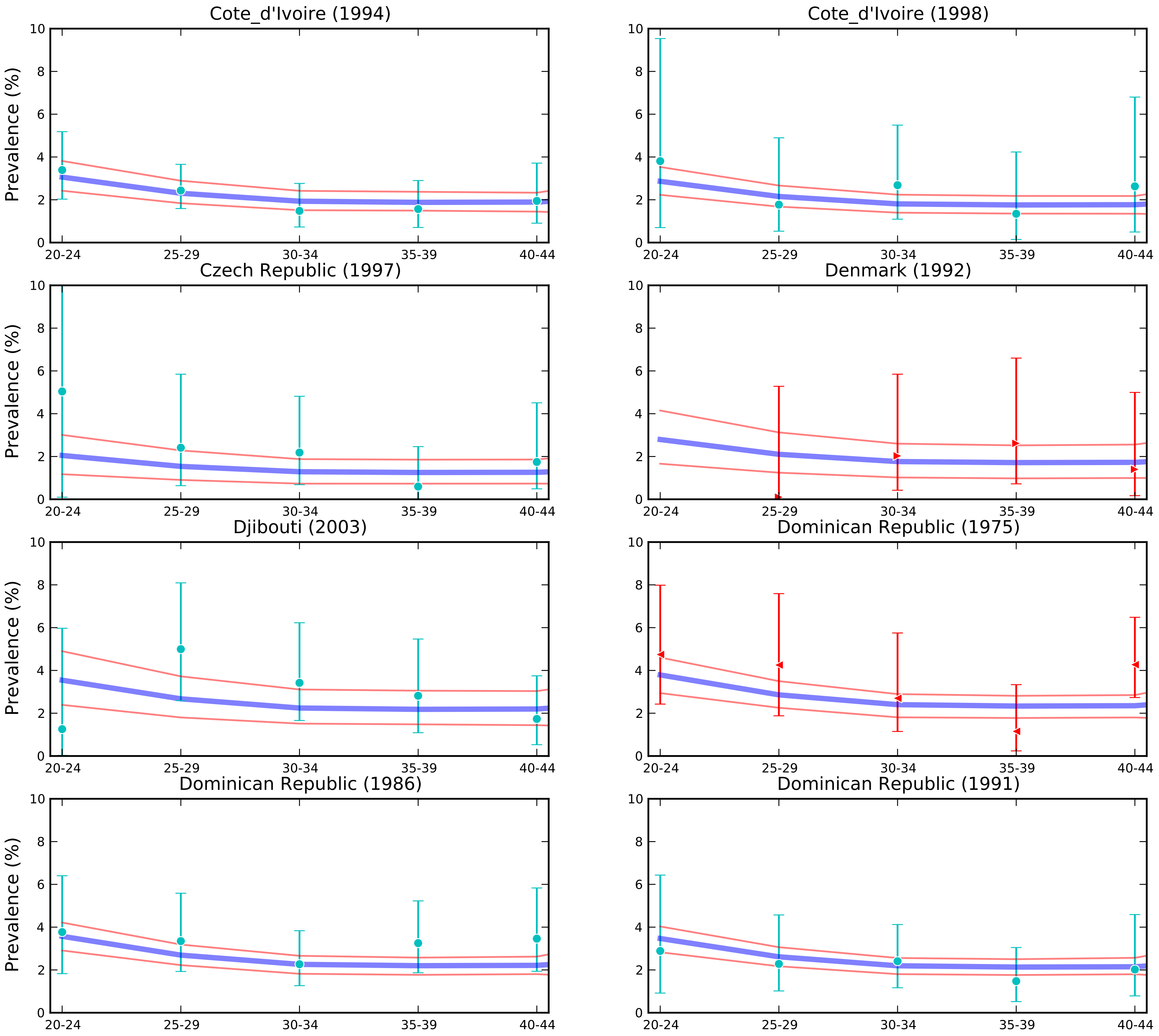
Cote_d'Ivoire (1994)
Cote_d'Ivoire (1998)
Czech Republic (1997)
Denmark (1992)
Djibouti (2003)
Dominican Republic (1975)
Dominican Republic (1986)
Dominican Republic (1991)
Prevalence (%)
0
2
4
6
8
10
20-24
25-29
30-34
35-39
40-44

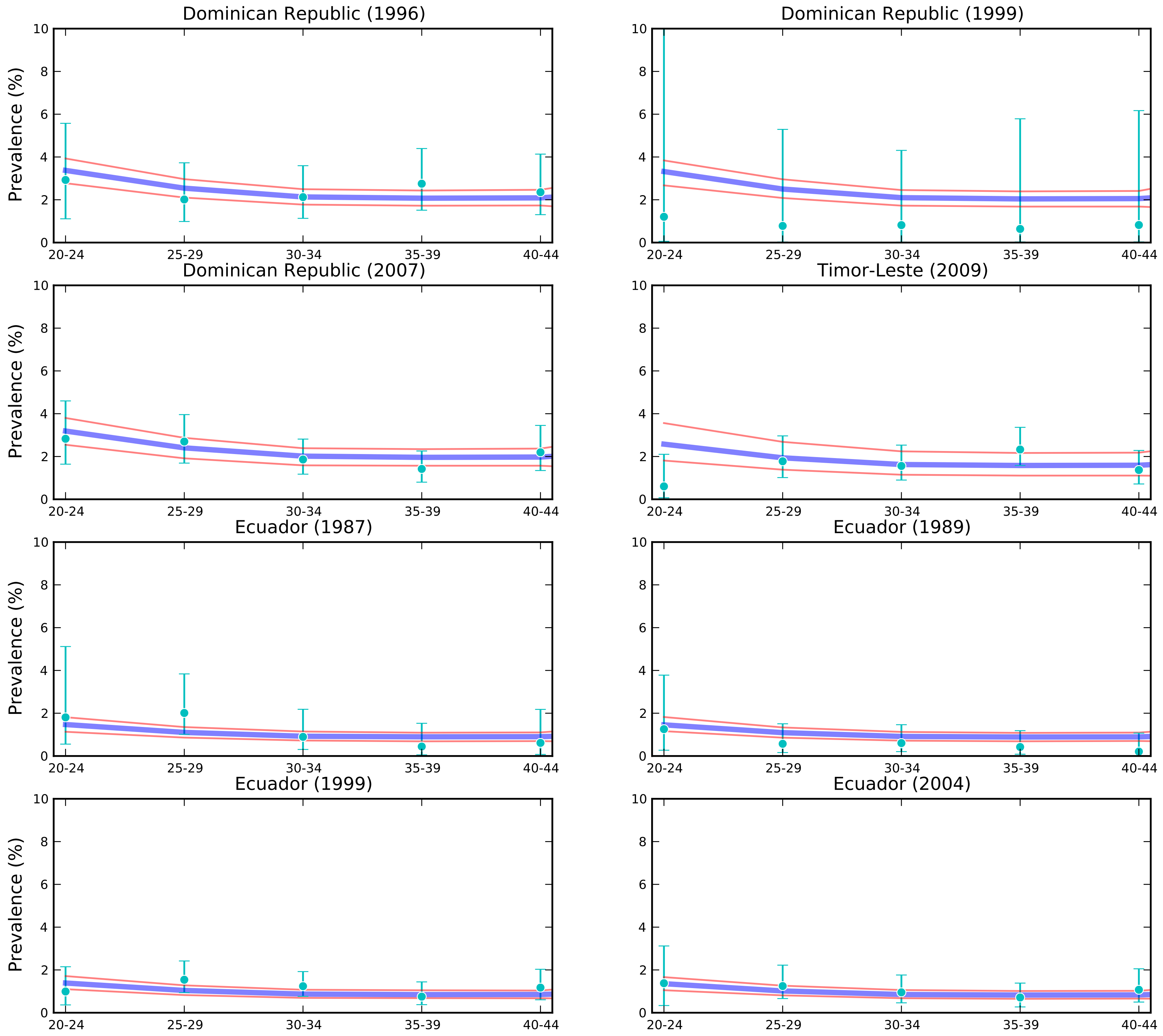
Dominican Republic (1996)
Dominican Republic (1999)
Dominican Republic (2007)
Timor-Leste (2009)
Ecuador (1987)
Ecuador (1989)
Ecuador (1999)
Ecuador (2004)
Prevalence (%)
0
2
4
6
8
10
20-24
25-29
30-34
35-39
40-44

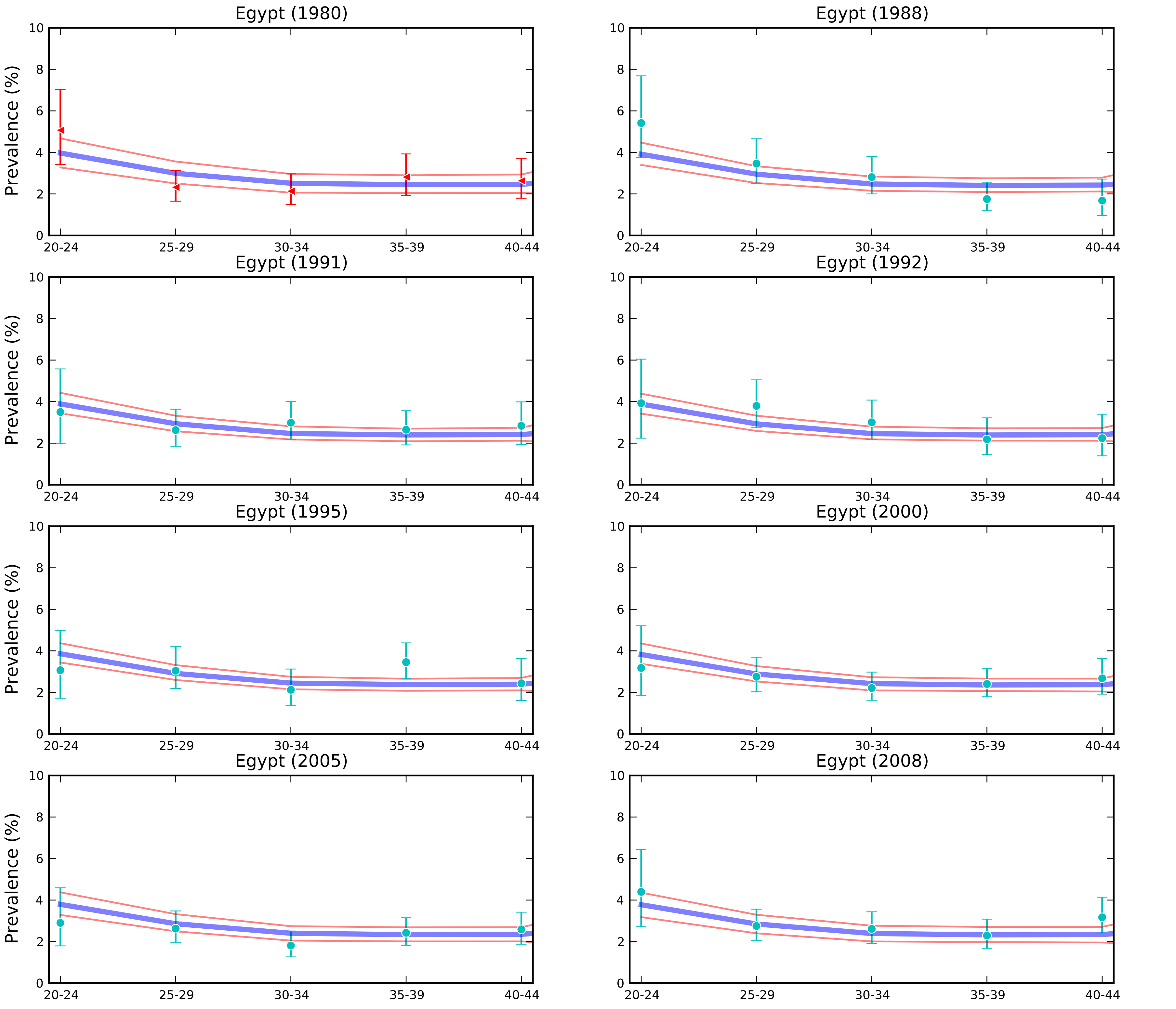
Egypt (1980)
Egypt (1988)
Egypt (1991)
Egypt (1992)
Egypt (1995)
Egypt (2000)
Egypt (2005)
Egypt (2008)
Prevalence (%)
0
2
4
6
8
10
20-24
25-29
30-34
35-39
40-44

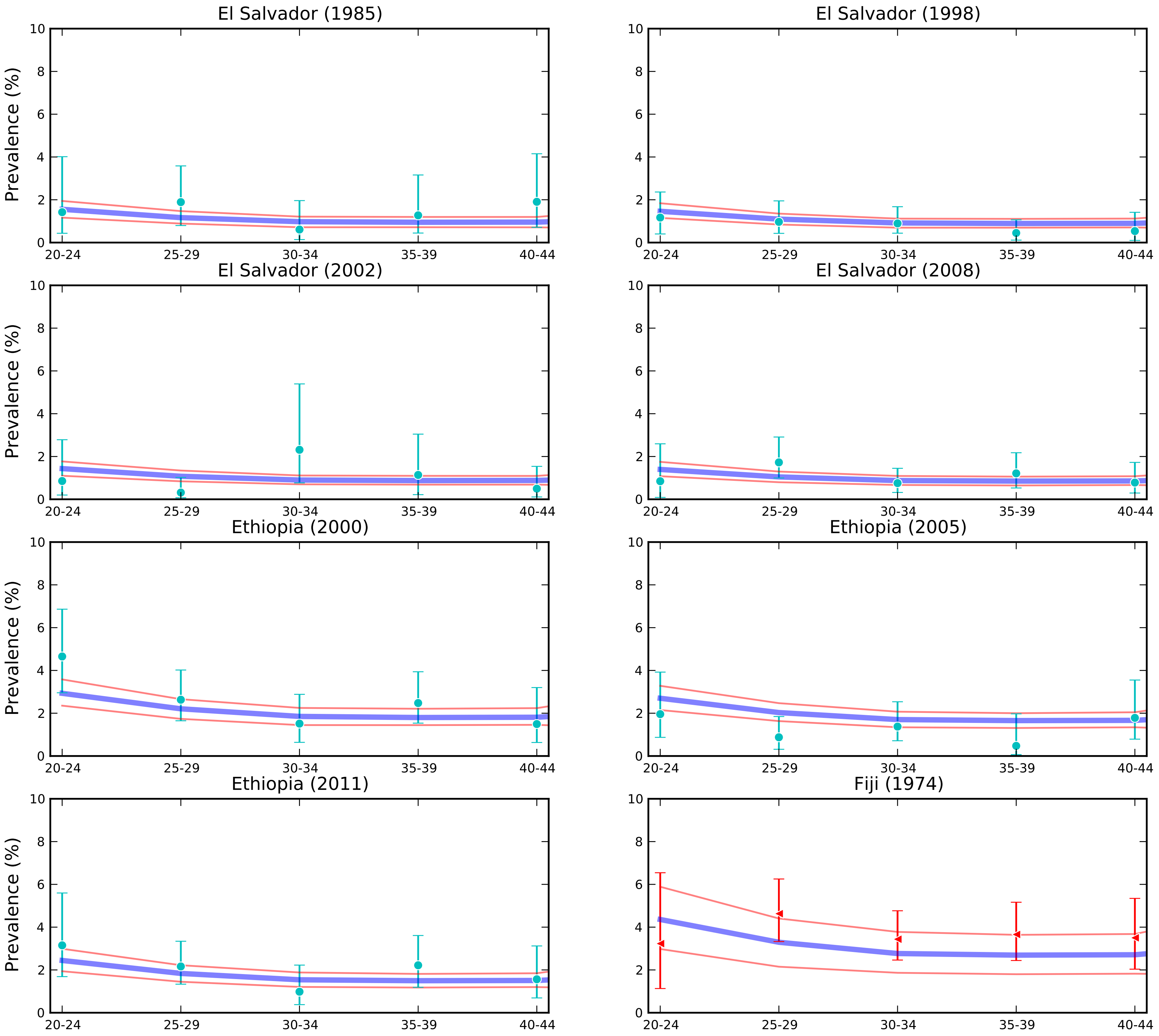
El Salvador (1985)
El Salvador (1998)
El Salvador (2002)
El Salvador (2008)
Ethiopia (2000)
Ethiopia (2005)
Ethiopia (2011)
Fiji (1974)
Prevalence (%)
0
2
4
6
8
10
20-24
25-29
30-34
35-39
40-44

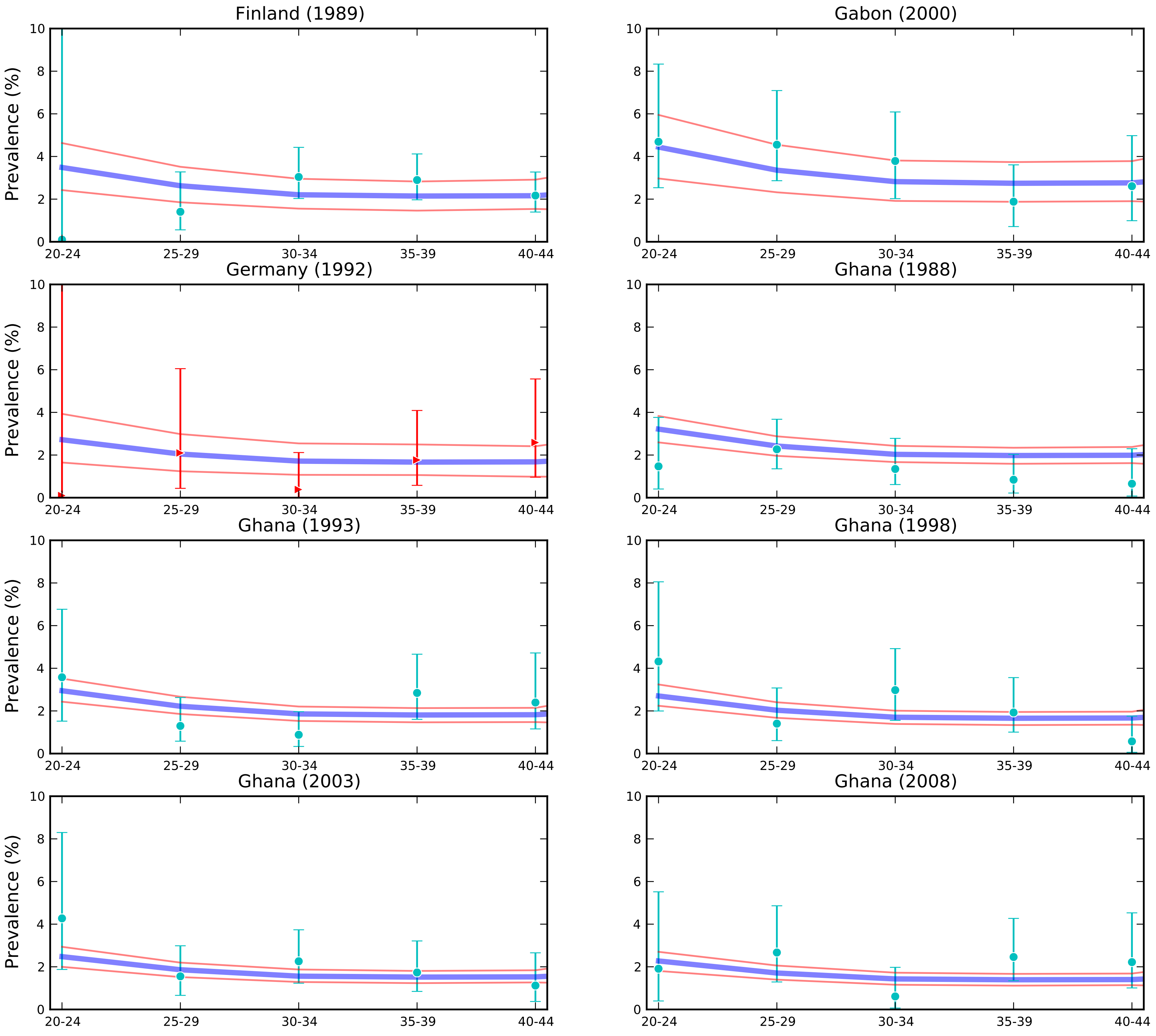
Finland (1989)
Gabon (2000)
Germany (1992)
Ghana (1988)
Ghana (1993)
Ghana (1998)
Ghana (2003)
Ghana (2008)
Prevalence (%)
0
2
4
6
8
10
20-24
25-29
30-34
35-39
40-44

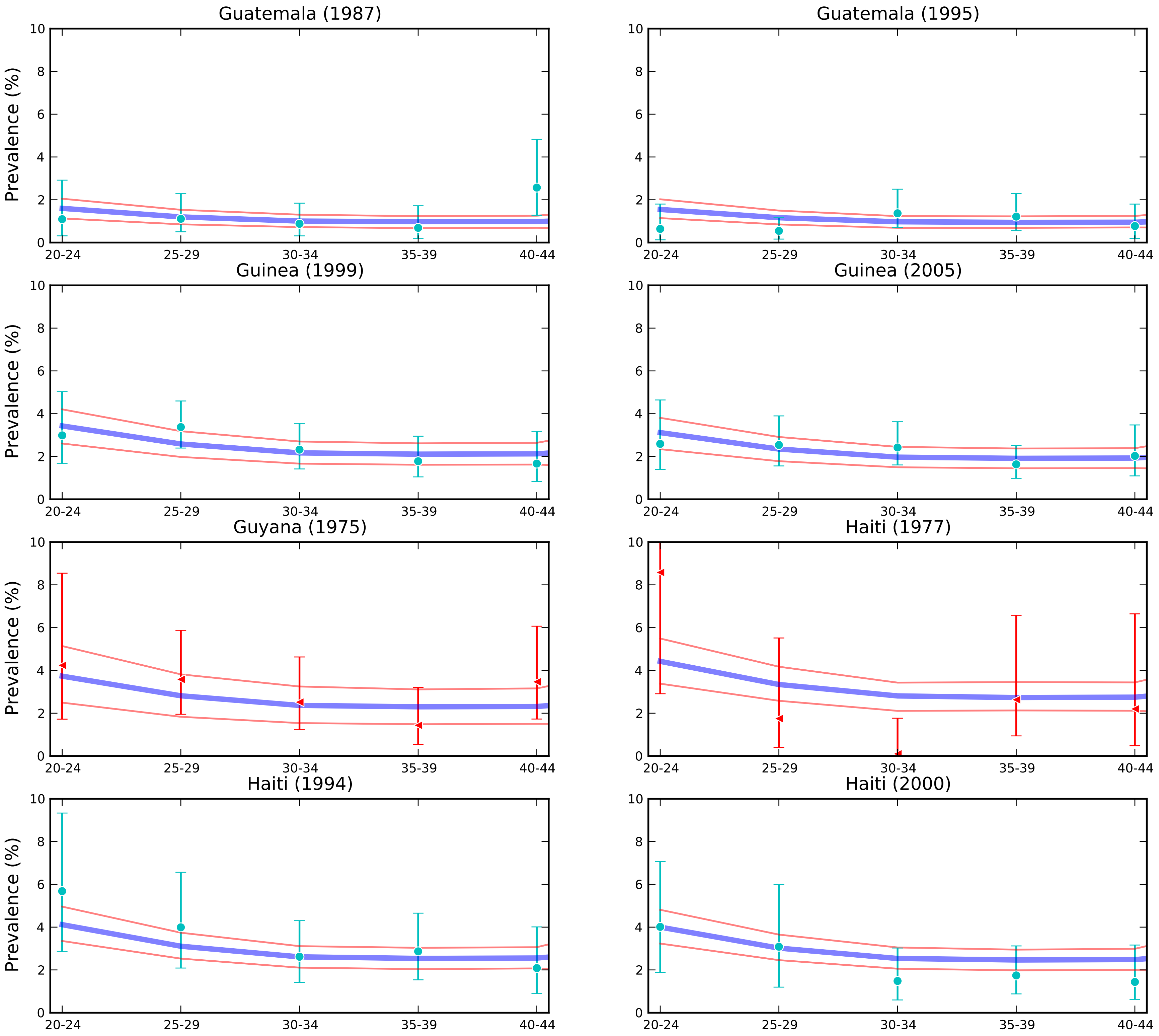
Guatemala (1987)
Guatemala (1995)
Guinea (1999)
Guinea (2005)
Guyana (1975)
Haiti (1977)
Haiti (1994)
Haiti (2000)
Prevalence (%)
0
2
4
6
8
10
20-24
25-29
30-34
35-39
40-44

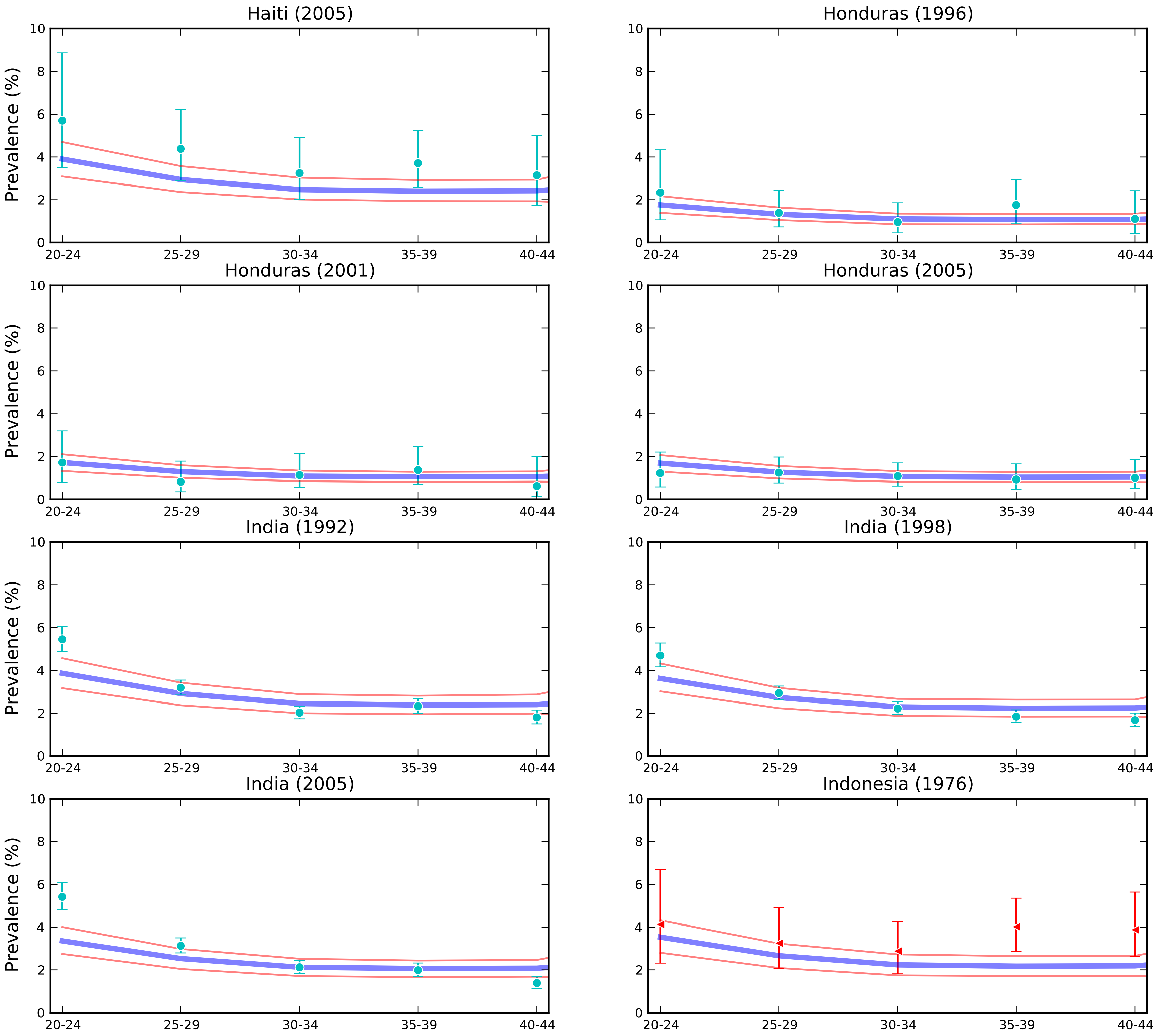

Haiti (2005)
Honduras (1996)
Honduras (2001)
Honduras (2005)
India (1992)
India (1998)
India (2005)
Indonesia (1976)
Prevalence (%)
0
2
4
6
8
10
20-24
25-29
30-34
35-39
40-44

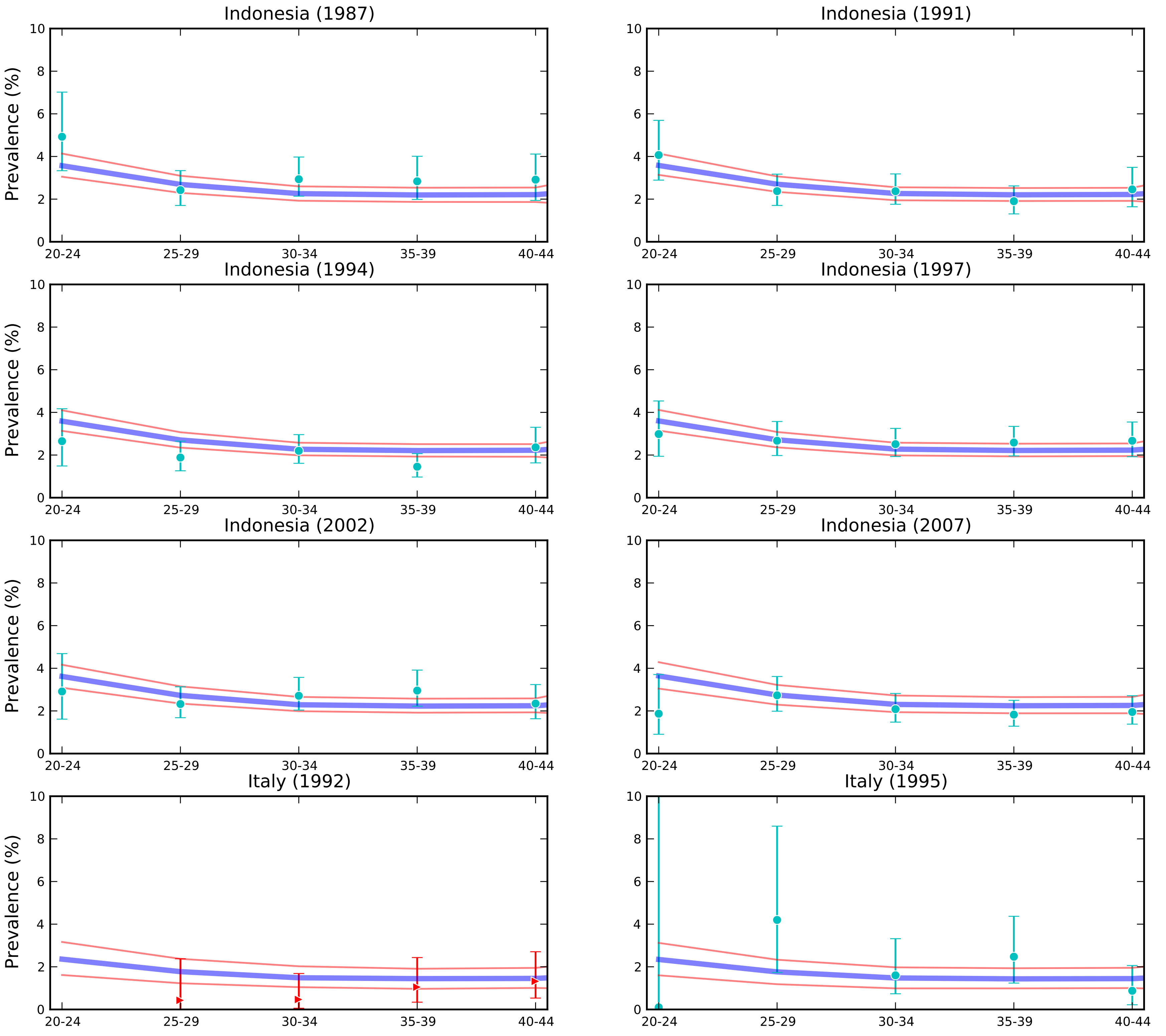
Indonesia (1987)
Indonesia (1991)
Indonesia (1994)
Indonesia (1997)
Indonesia (2002)
Indonesia (2007)
Italy (1992)
Italy (1995)
Prevalence (%)
0
2
4
6
8
10
20-24
25-29
30-34
35-39
40-44

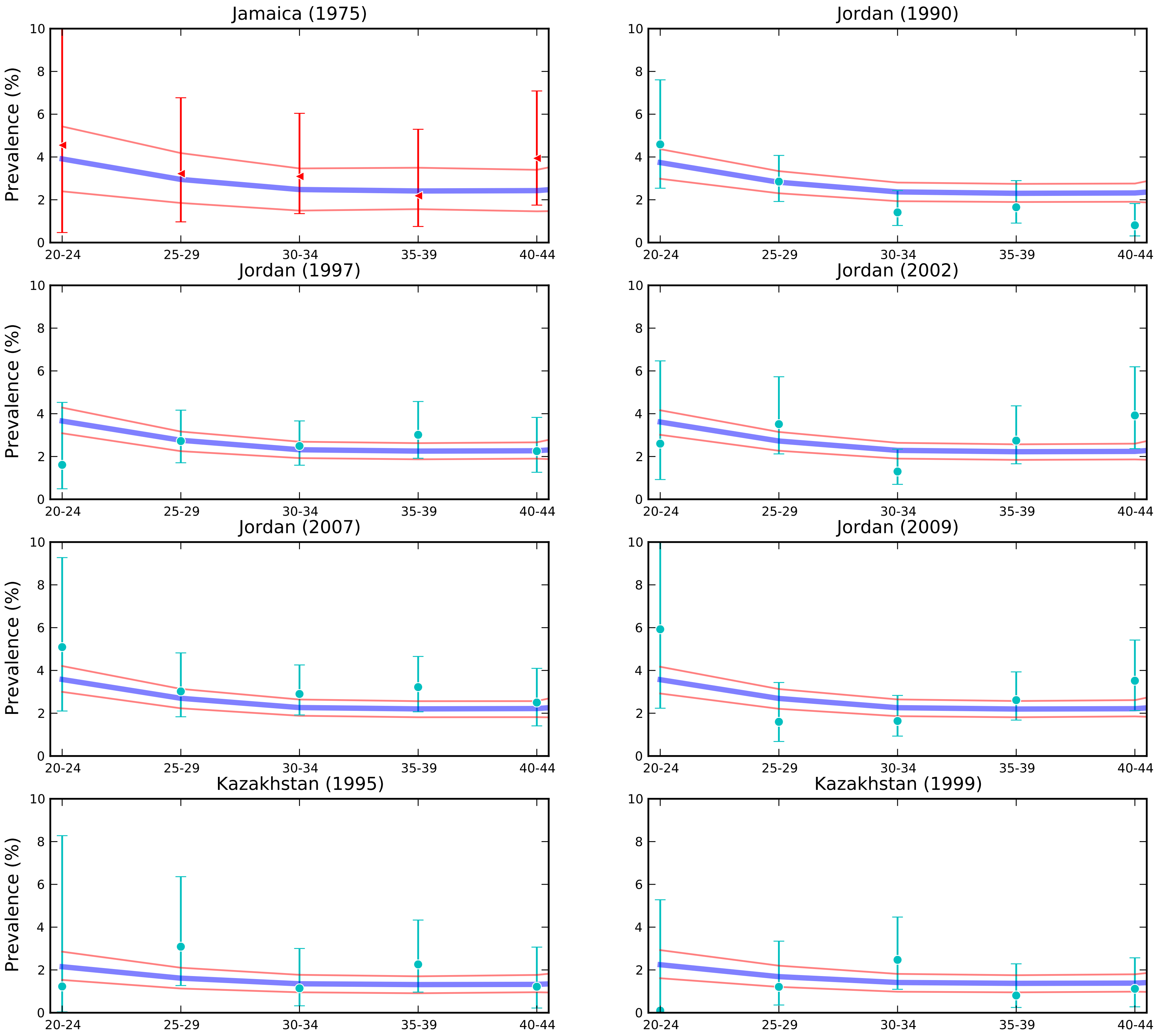
Jamaica (1975)
Jordan (1990)
Jordan (1997)
Jordan (2002)
Jordan (2007)
Jordan (2009)
Kazakhstan (1995)
Kazakhstan (1999)
Prevalence (%)
0
2
4
6
8
10
20-24
25-29
30-34
35-39
40-44

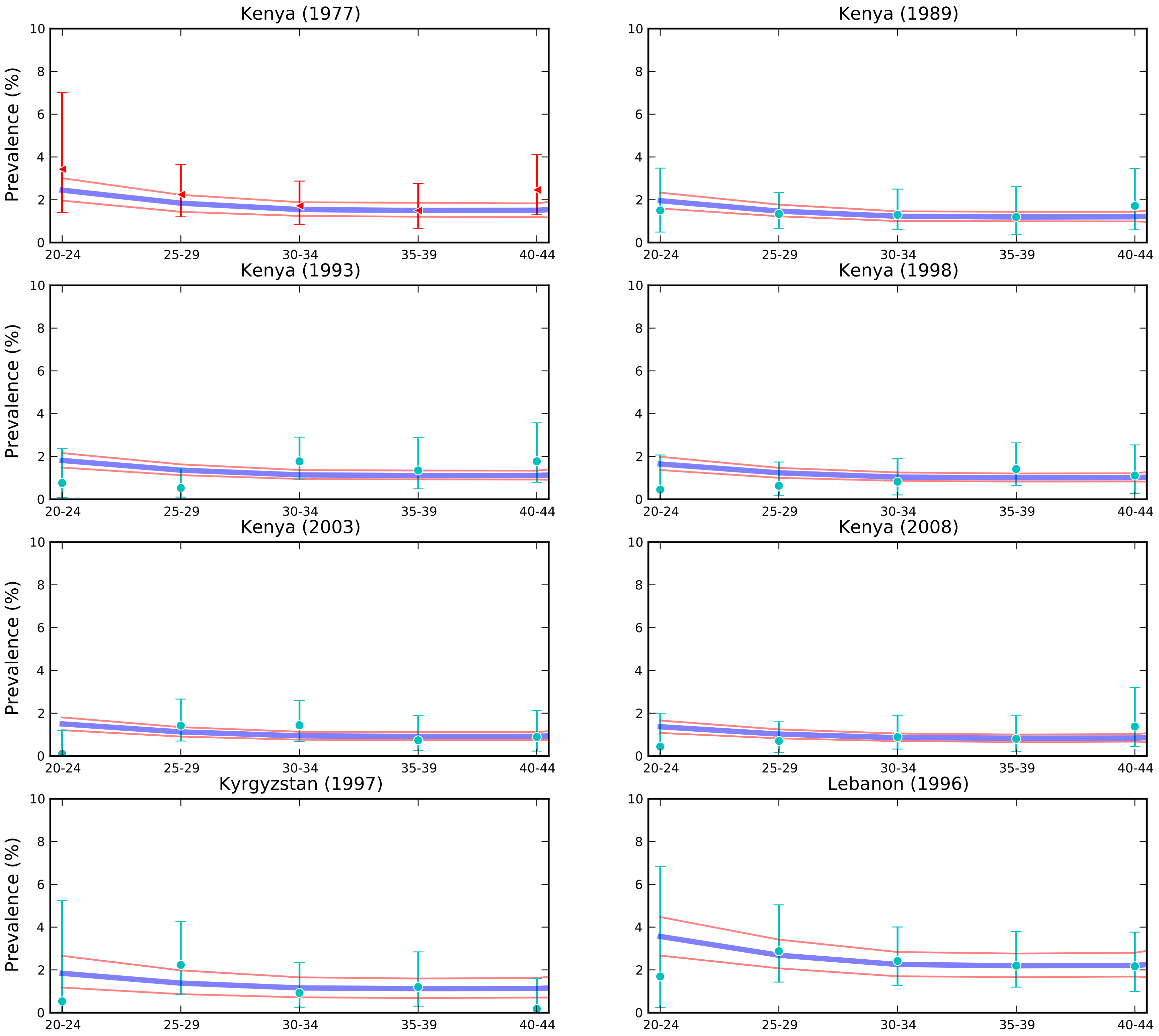
Kenya (1977)
Kenya (1989)
Kenya (1993)
Kenya (1998)
Kenya (2003)
Kenya (2008)
Kyrgyzstan (1997)
Lebanon (1996)
Prevalence (%)
0
2
4
6
8
10
20-24
25-29
30-34
35-39
40-44

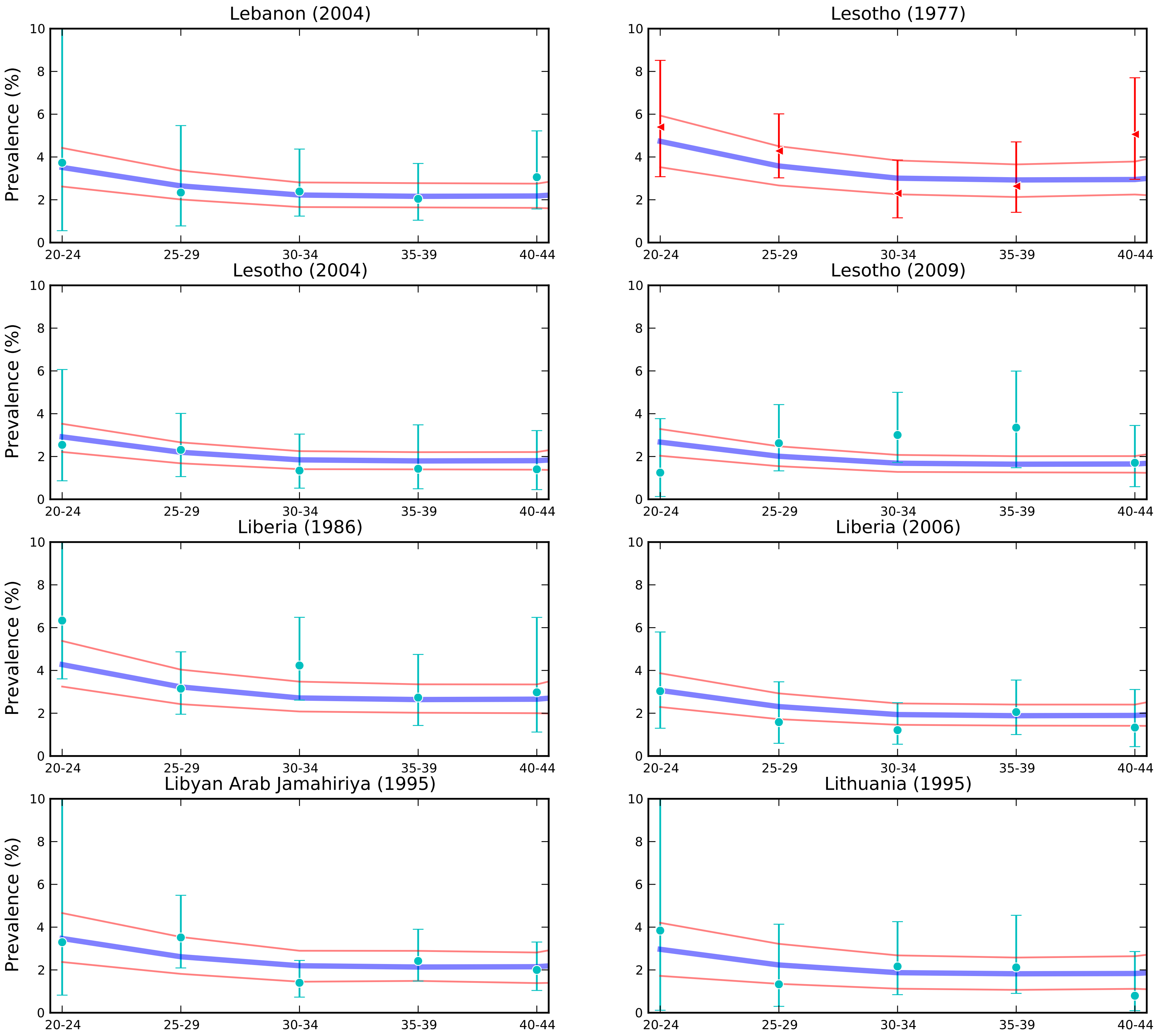
Lebanon (2004)
Lesotho (1977)
Lesotho (2004)
Lesotho (2009)
Liberia (1986)
Liberia (2006)
Libyan Arab Jamahiriya (1995)
Lithuania (1995)
Prevalence (%)
0
2
4
6
8
10
20-24
25-29
30-34
35-39
40-44

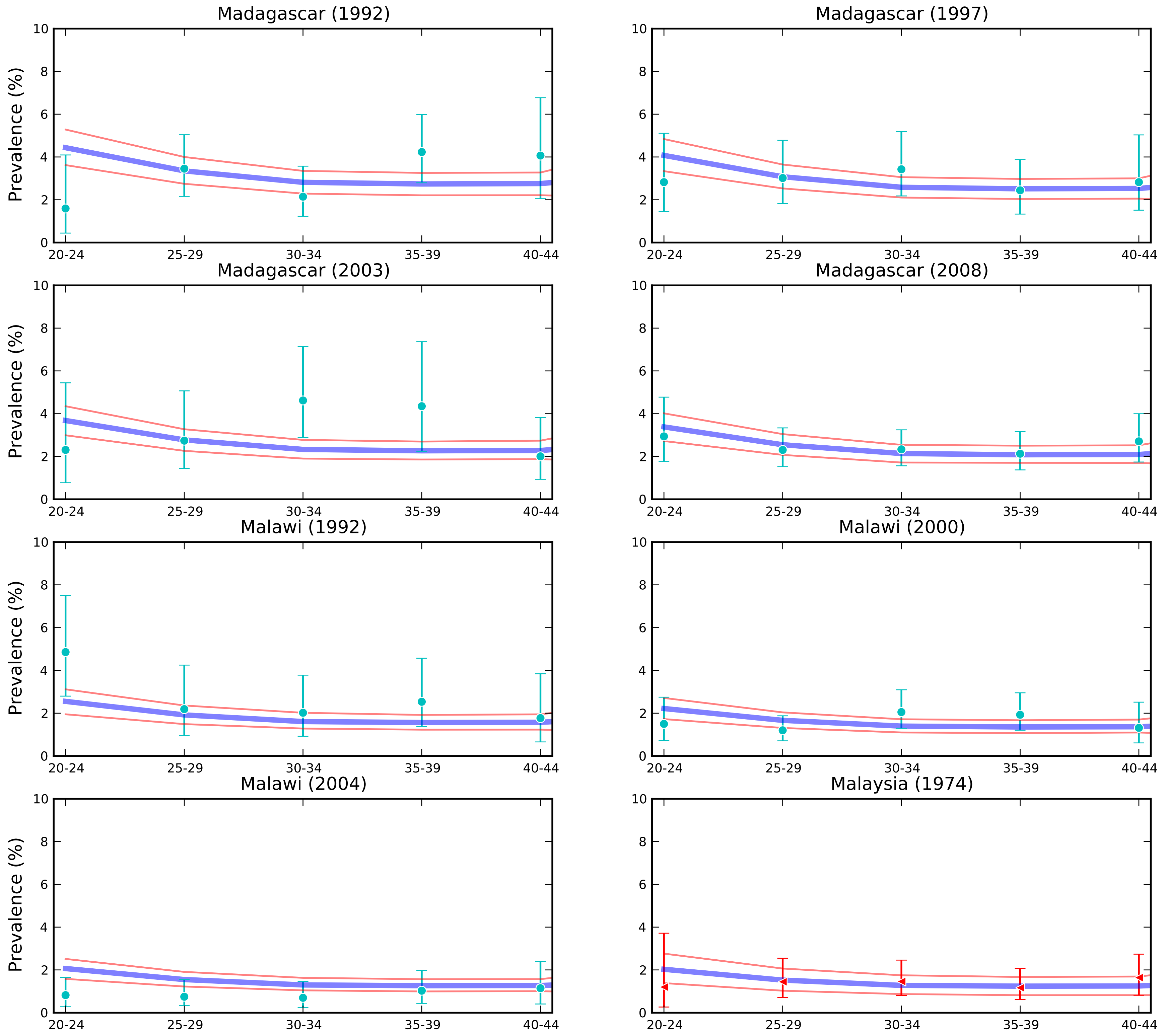

Madagascar (1992)
Madagascar (1997)
Madagascar (2003)
Madagascar (2008)
Malawi (1992)
Malawi (2000)
Malawi (2004)
Malaysia (1974)
Prevalence (%)
0
2
4
6
8
10
20-24
25-29
30-34
35-39
40-44

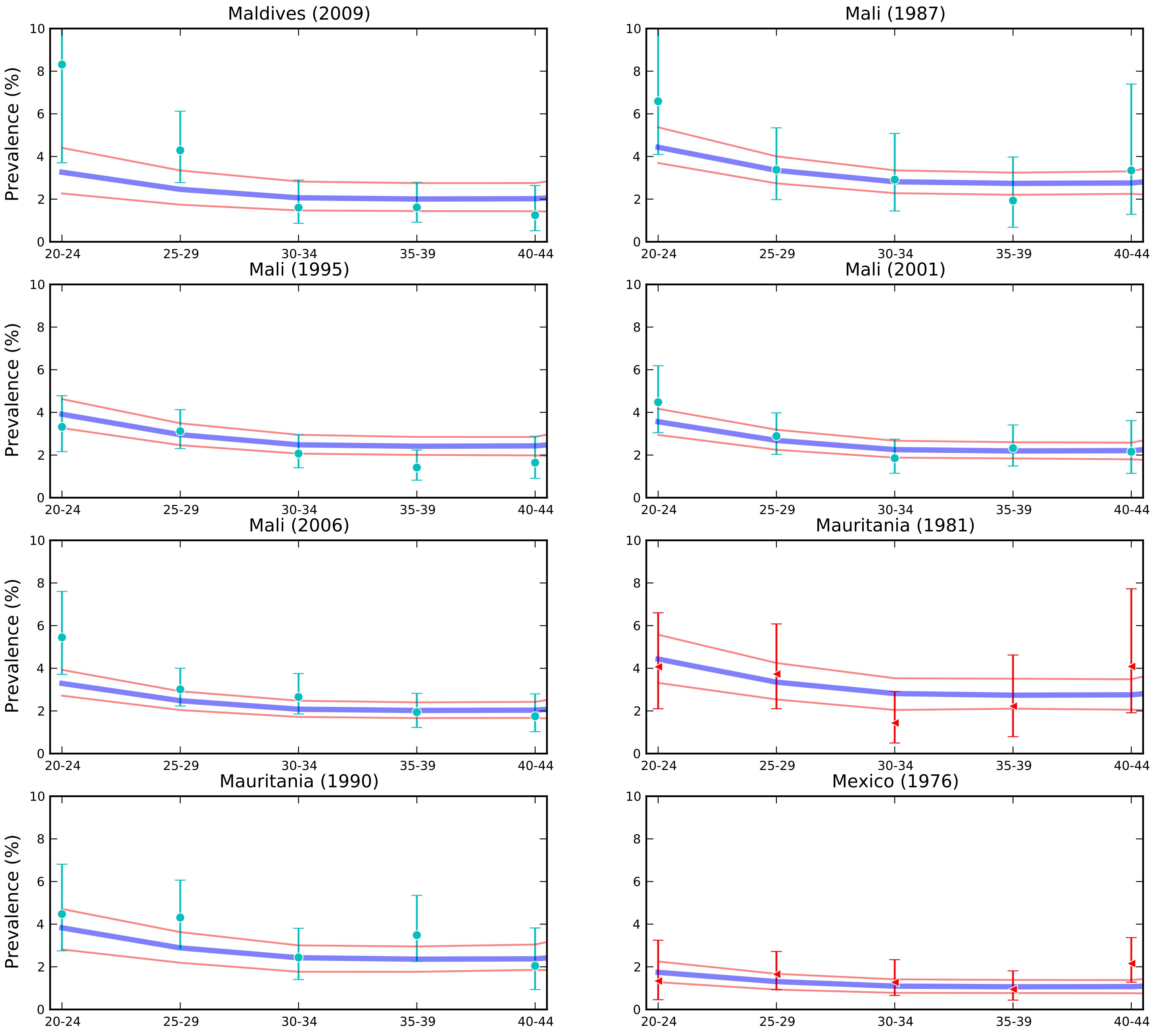
Maldives (2009)
Mali (1987)
Mali (1995)
Mali (2001)
Mali (2006)
Mauritania (1981)
Mauritania (1990)
Mexico (1976)
Prevalence (%)
0
2
4
6
8
10
20-24
25-29
30-34
35-39
40-44

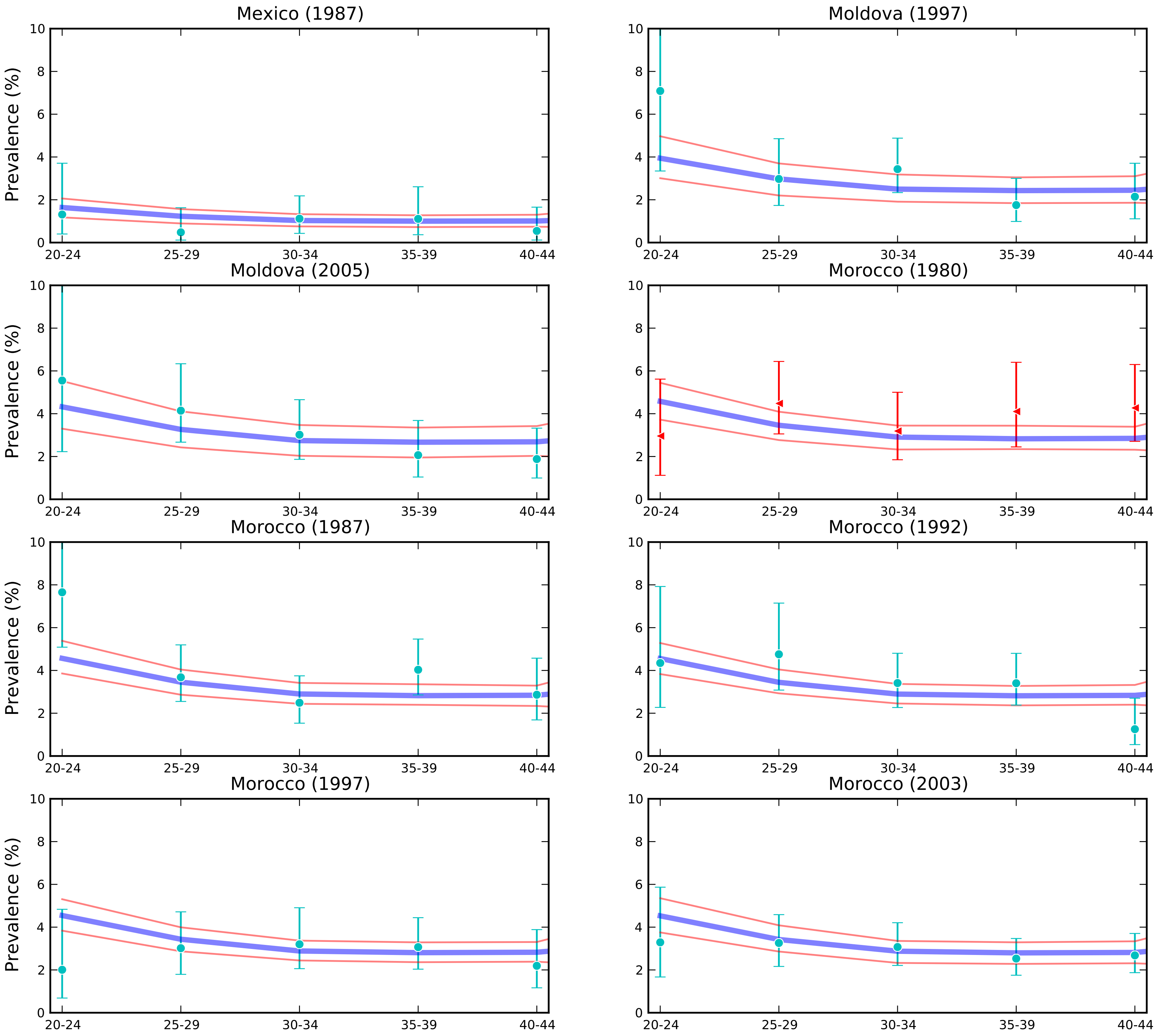

Mexico (1987)
Moldova (1997)
Moldova (2005)
Morocco (1980)
Morocco (1987)
Morocco (1992)
Morocco (1997)
Morocco (2003)
Prevalence (%)
0
2
4
6
8
10
20-24
25-29
30-34
35-39
40-44

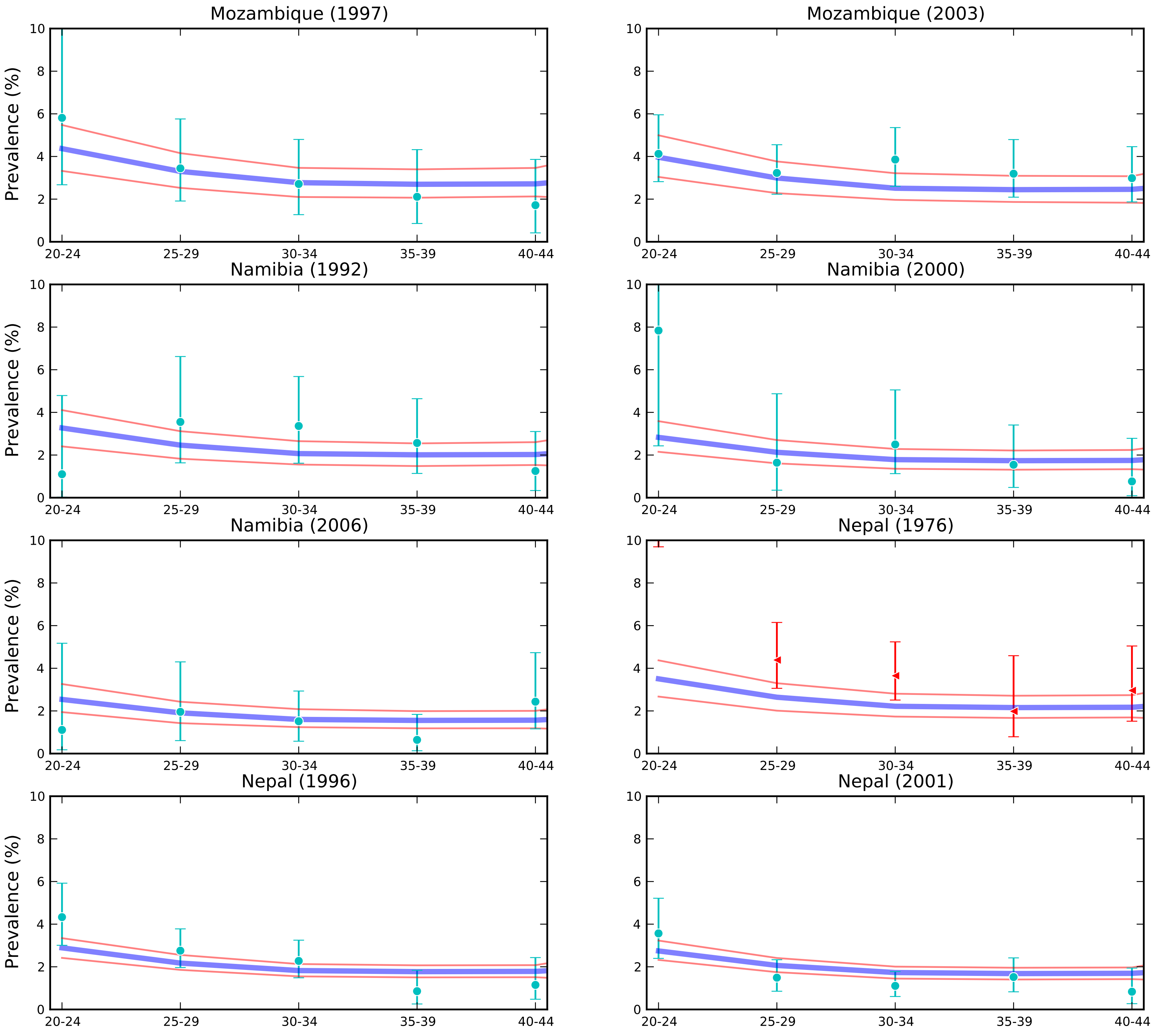

Mozambique (1997)
Mozambique (2003)
Namibia (1992)
Namibia (2000)
Namibia (2006)
Nepal (1976)
Nepal (1996)
Nepal (2001)
Prevalence (%)
0
2
4
6
8
10
20-24
25-29
30-34
35-39
40-44

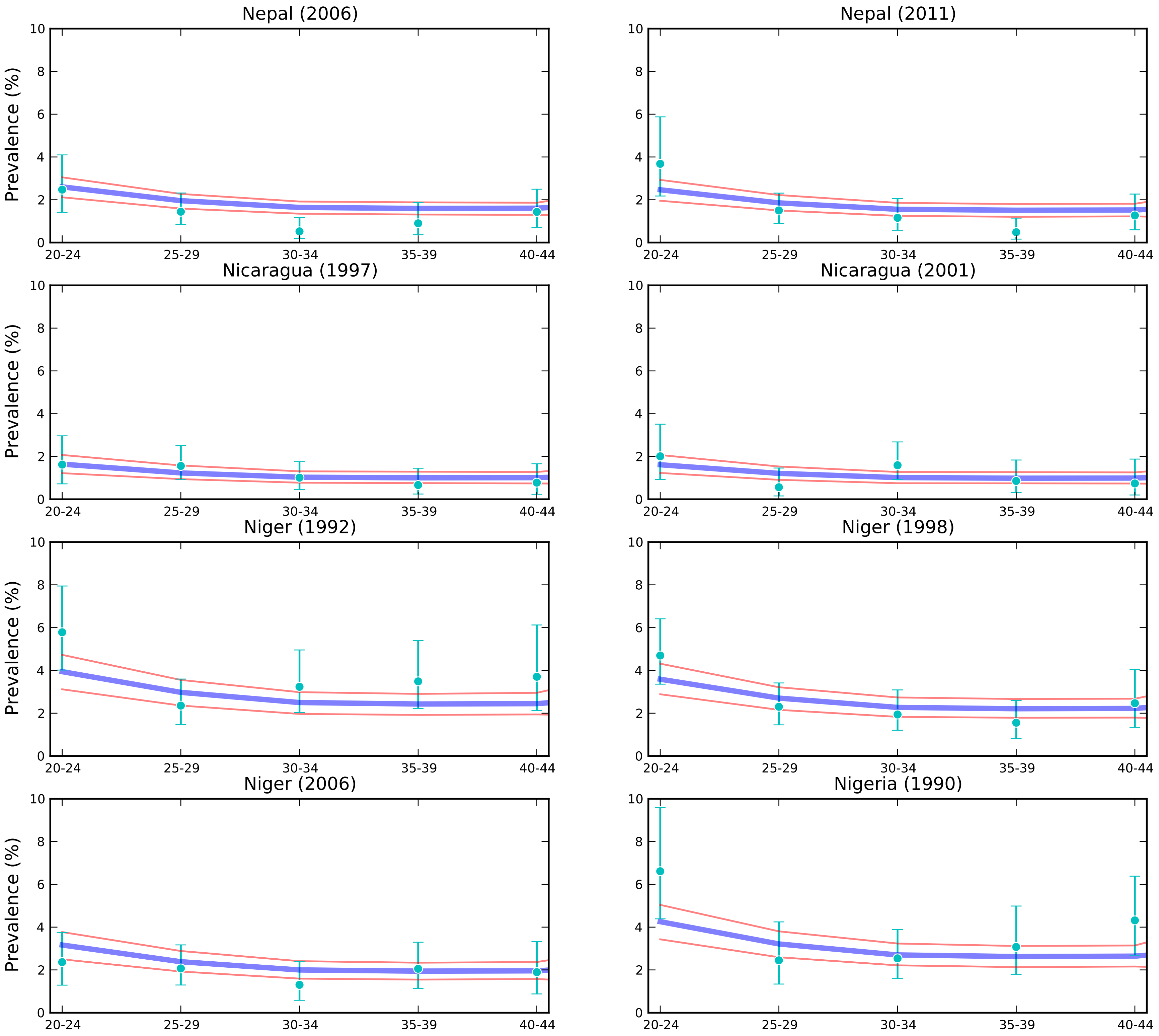

Nepal (2006)
Nepal (2011)
Nicaragua (1997)
Nicaragua (2001)
Niger (1992)
Niger (1998)
Niger (2006)
Nigeria (1990)
Prevalence (%)
0
2
4
6
8
10
20-24
25-29
30-34
35-39
40-44

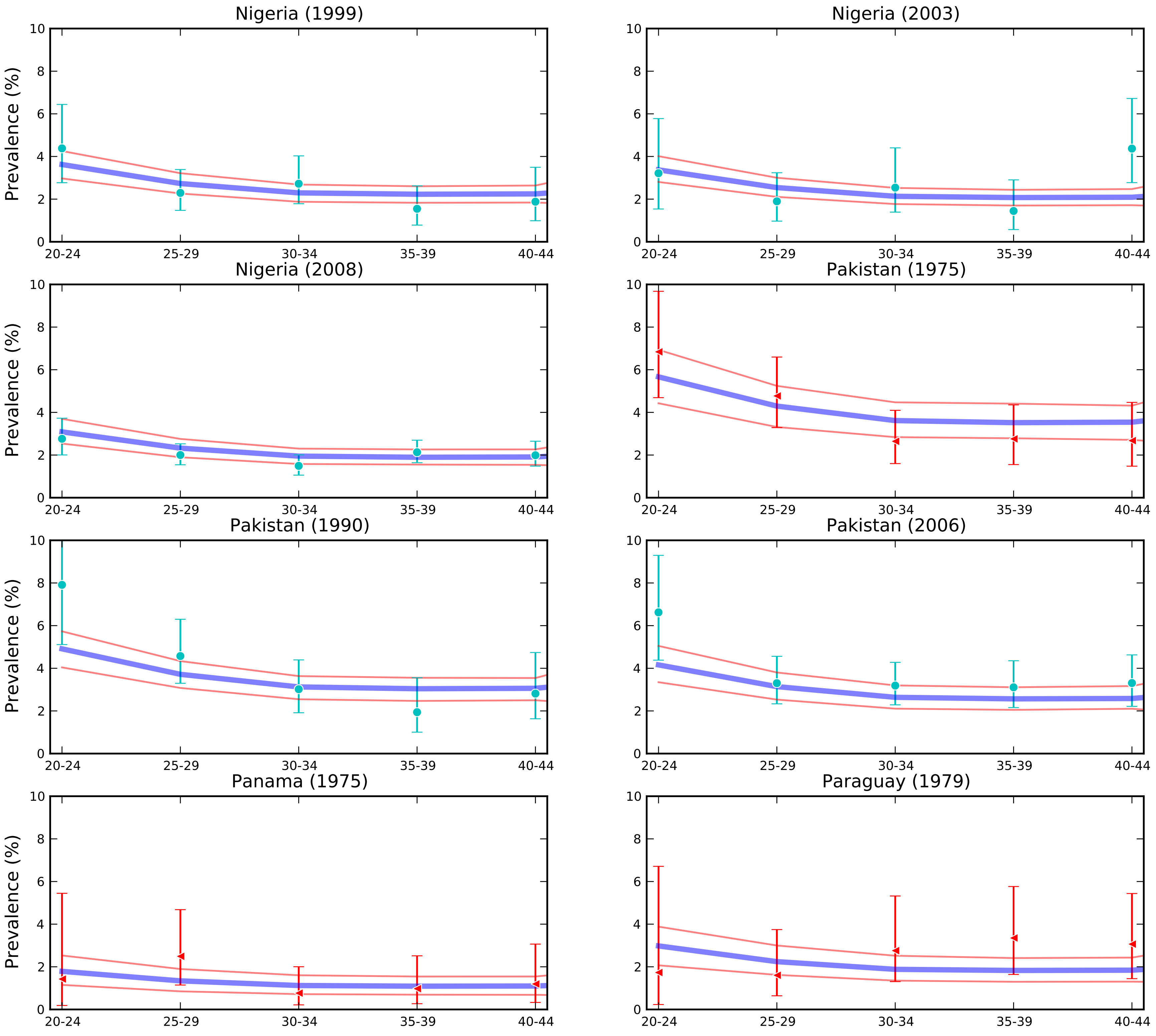
Nigeria (1999)
Nigeria (2003)
Nigeria (2008)
Pakistan (1975)
Pakistan (1990)
Pakistan (2006)
Panama (1975)
Paraguay (1979)
Prevalence (%)
0
2
4
6
8
10
20-24
25-29
30-34
35-39
40-44

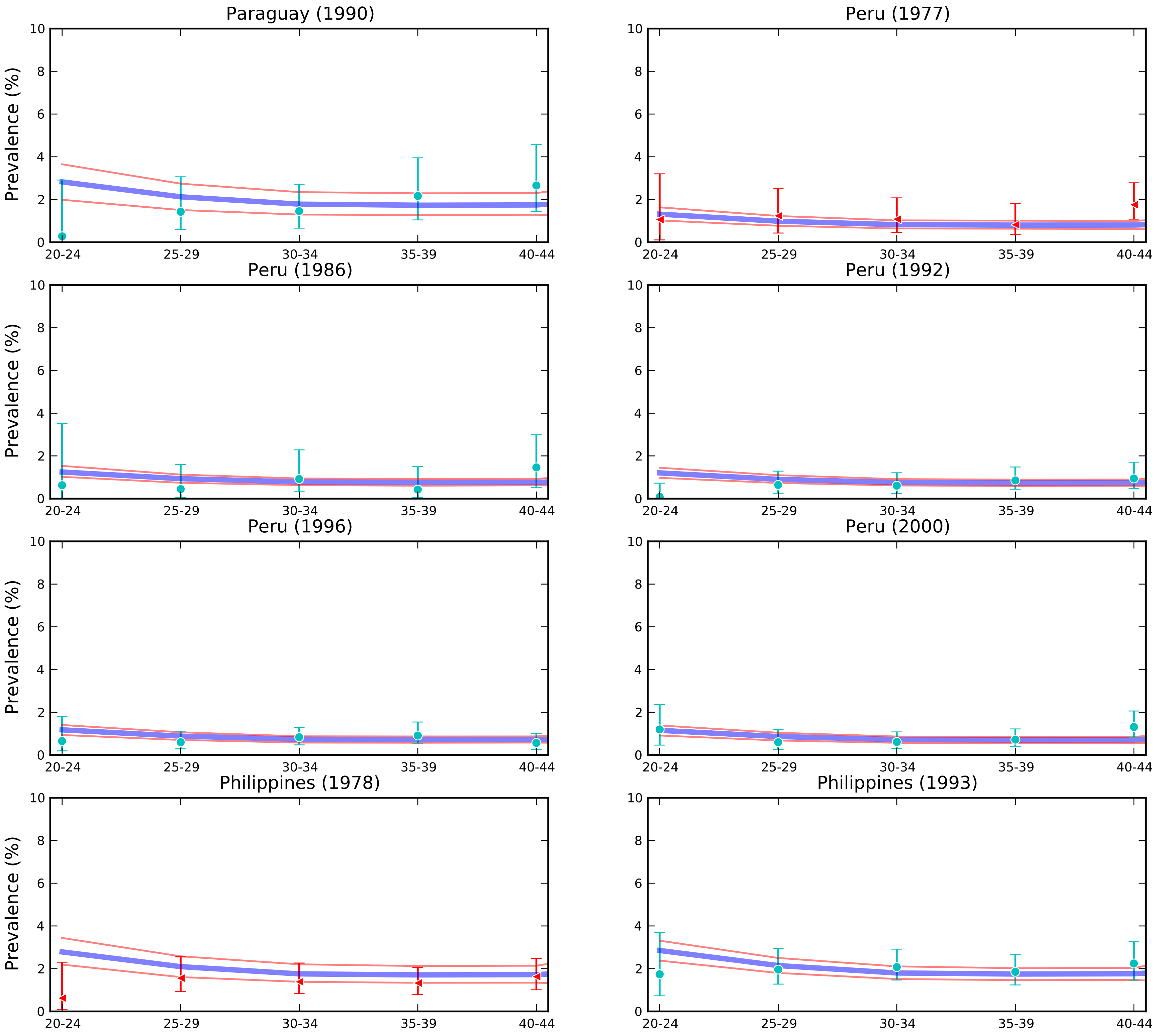

Paraguay (1990)
Peru (1977)
Peru (1986)
Peru (1992)
Peru (1996)
Peru (2000)
Philippines (1978)
Philippines (1993)
Prevalence (%)
0
2
4
6
8
10
20-24
25-29
30-34
35-39
40-44

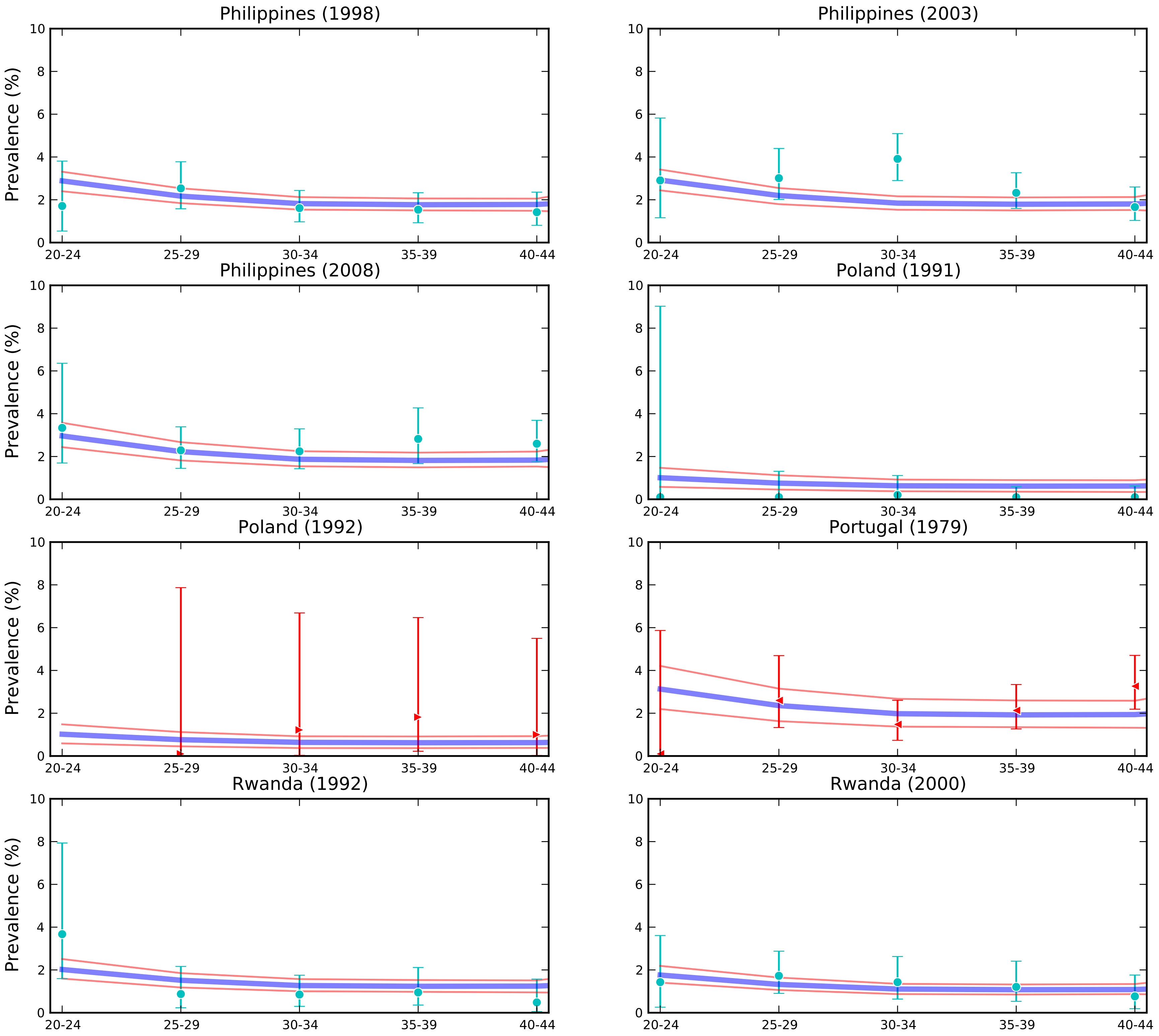

Philippines (1998)
Philippines (2003)
Philippines (2008)
Poland (1991)
Poland (1992)
Portugal (1979)
Rwanda (1992)
Rwanda (2000)
Prevalence (%)
0
2
4
6
8
10
20-24
25-29
30-34
35-39
40-44

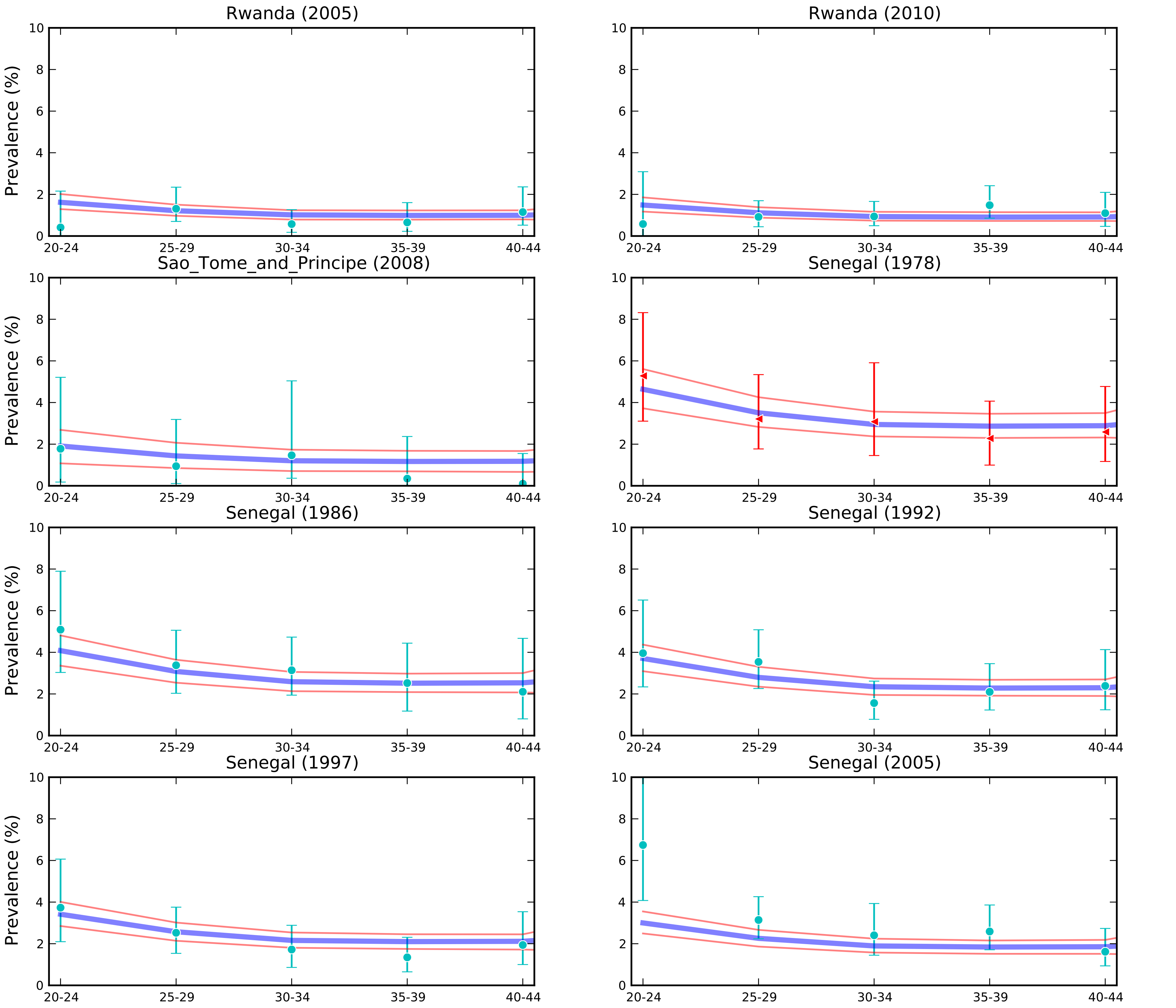

Rwanda (2005)
Rwanda (2010)
Sao_Tome_and_Principe (2008)
Senegal (1978)
Senegal (1986)
Senegal (1992)
Senegal (1997)
Senegal (2005)
Prevalence (%)
0
2
4
6
8
10
20-24
25-29
30-34
35-39
40-44

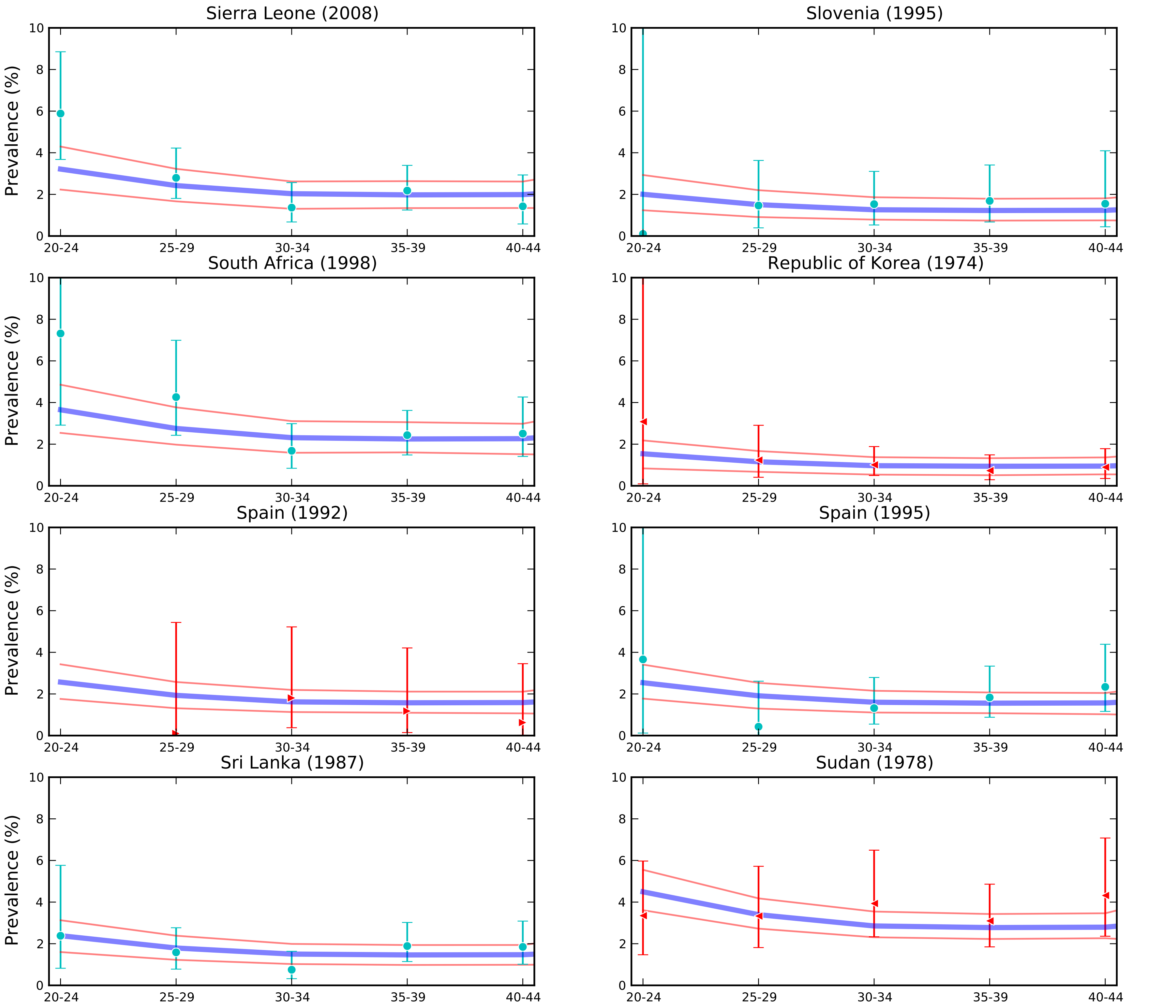

Sierra Leone (2008)
Slovenia (1995)
South Africa (1998)
Republic of Korea (1974)
Spain (1992)
Spain (1995)
Sri Lanka (1987)
Sudan (1978)
Prevalence (%)
0
2
4
6
8
10
20-24
25-29
30-34
35-39
40-44

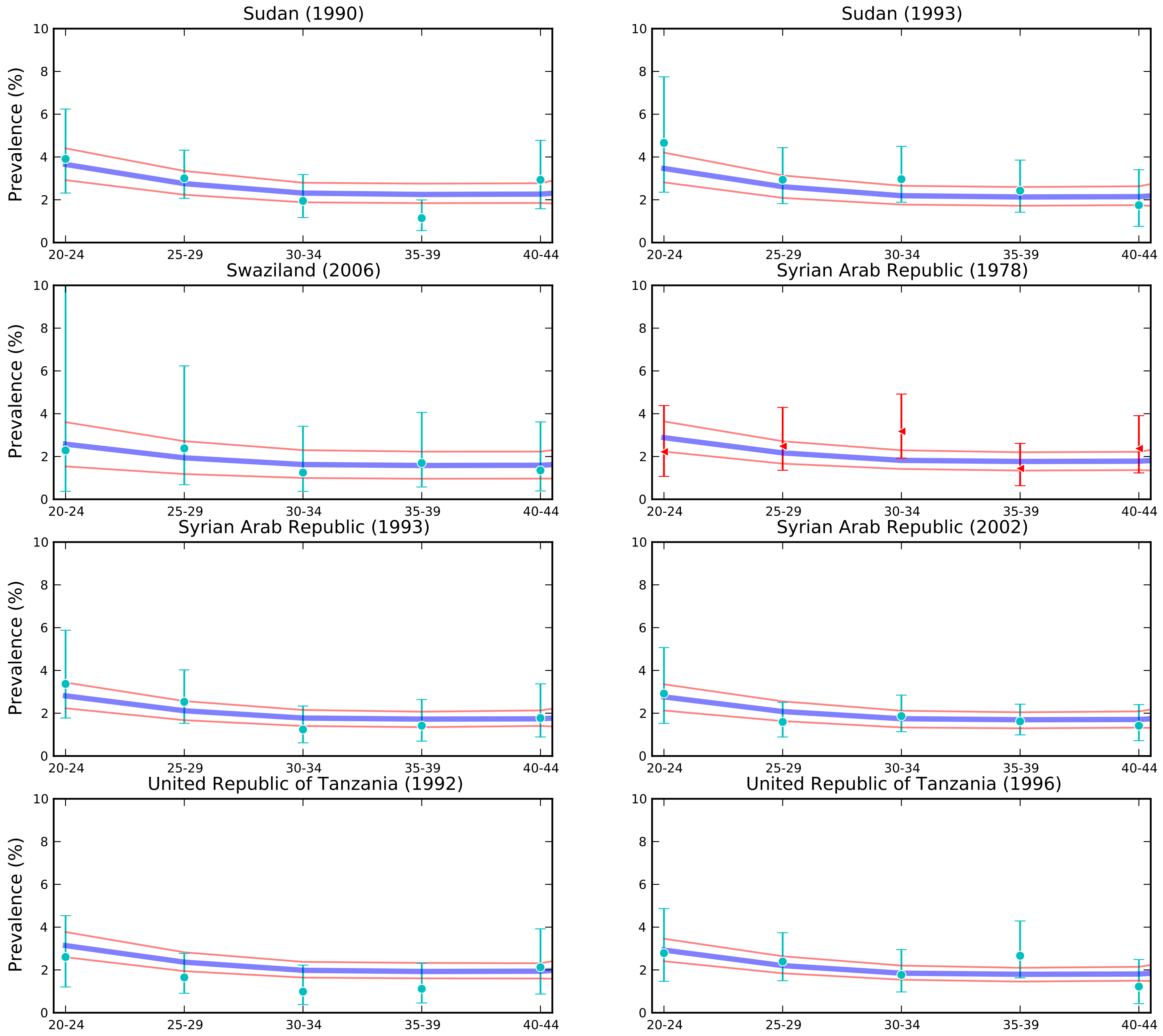
Sudan (1990)
Sudan (1993)
Swaziland (2006)
Syrian Arab Republic (1978)
Syrian Arab Republic (1993)
Syrian Arab Republic (2002)
United Republic of Tanzania (1992)
United Republic of Tanzania (1996)
Prevalence (%)
0
2
4
6
8
10
20-24
25-29
30-34
35-39
40-44

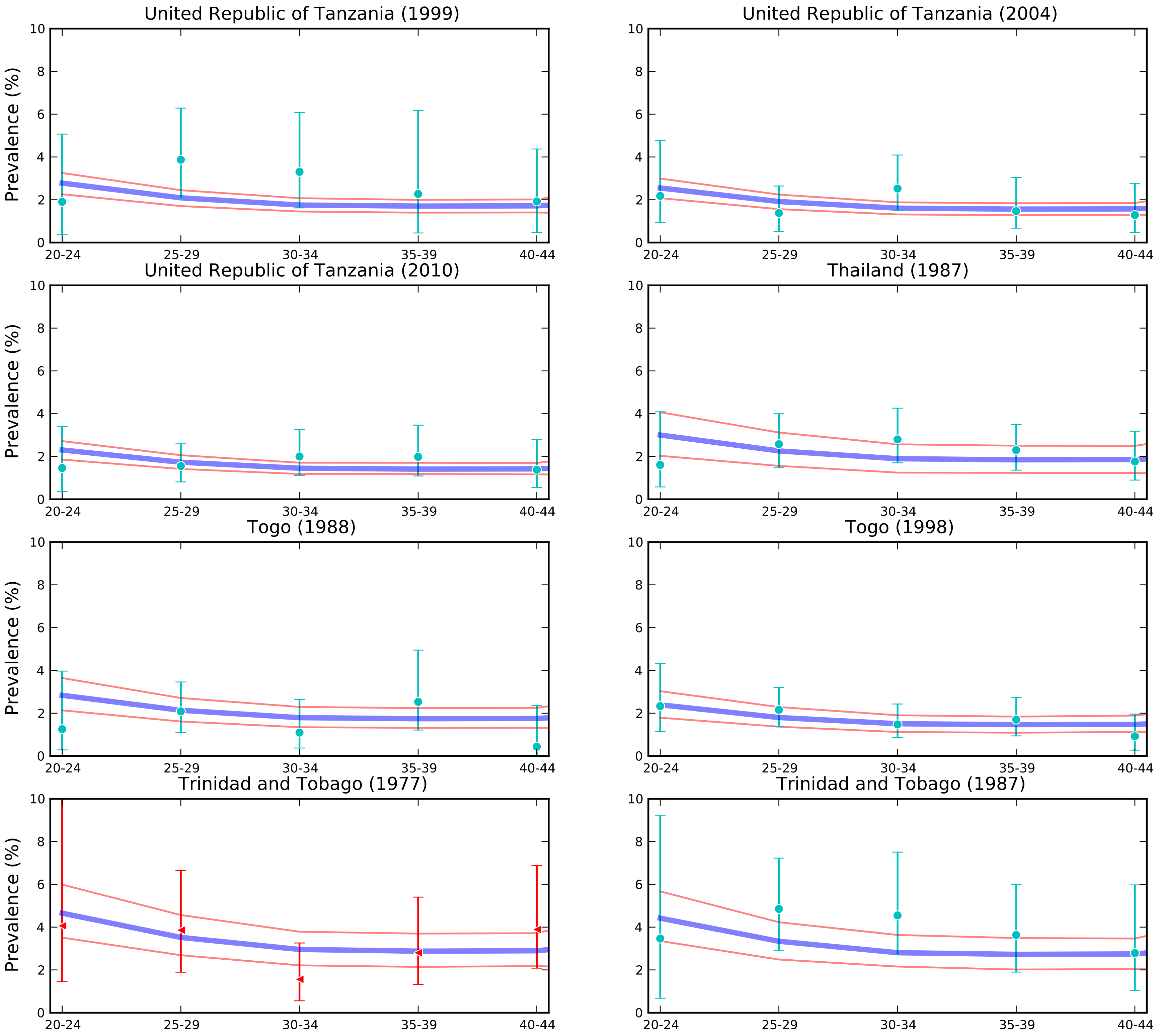
United Republic of Tanzania (1999)
United Republic of Tanzania (2004)
United Republic of Tanzania (2010)
Thailand (1987)
Togo (1988)
Togo (1998)
Trinidad and Tobago (1977)
Trinidad and Tobago (1987)
Prevalence (%)
0
2
4
6
8
10
20-24
25-29
30-34
35-39
40-44

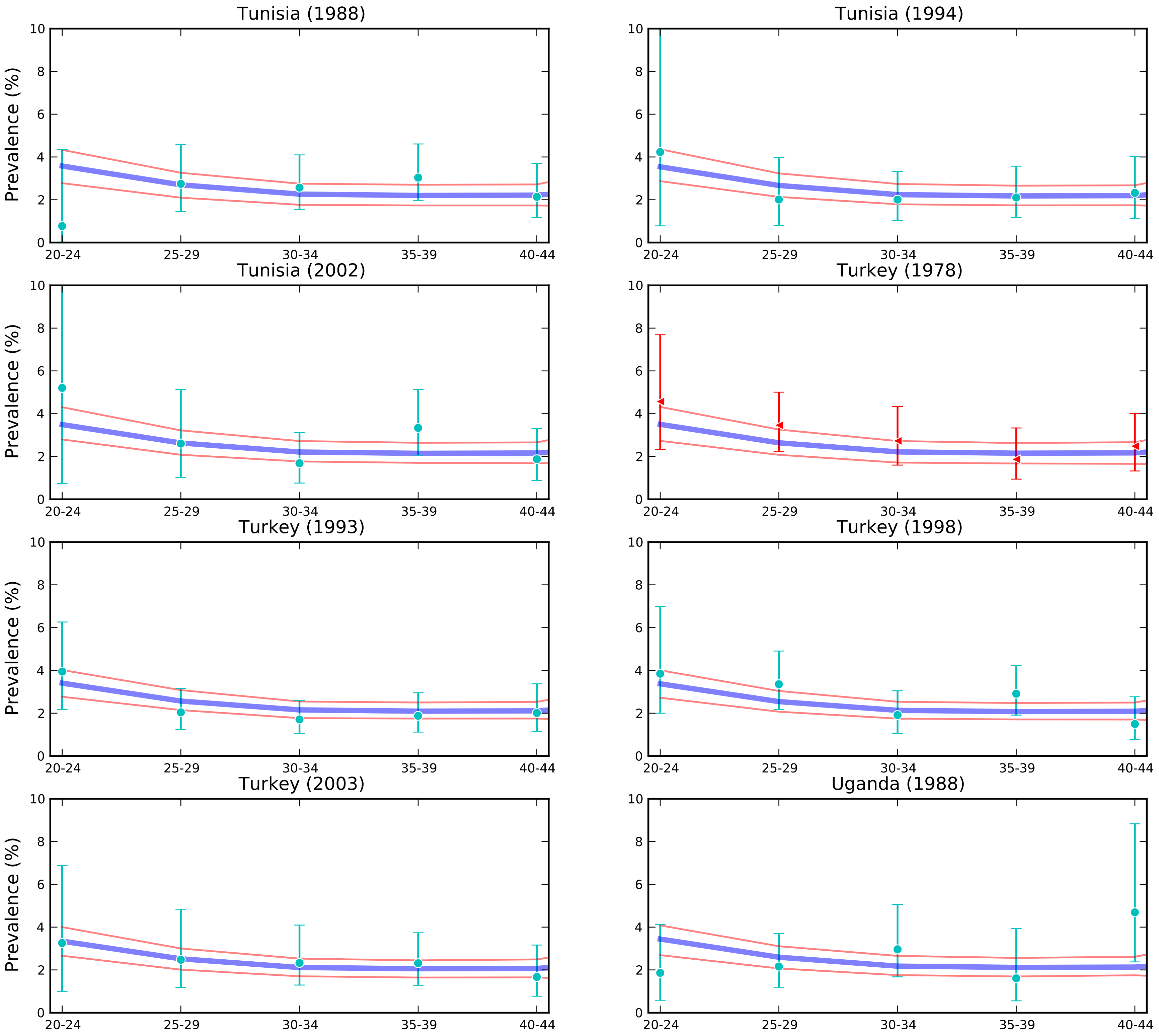
Tunisia (1988)
Tunisia (1994)
Tunisia (2002)
Turkey (1978)
Turkey (1993)
Turkey (1998)
Turkey (2003)
Uganda (1988)
Prevalence (%)
0
2
4
6
8
10
20-24
25-29
30-34
35-39
40-44

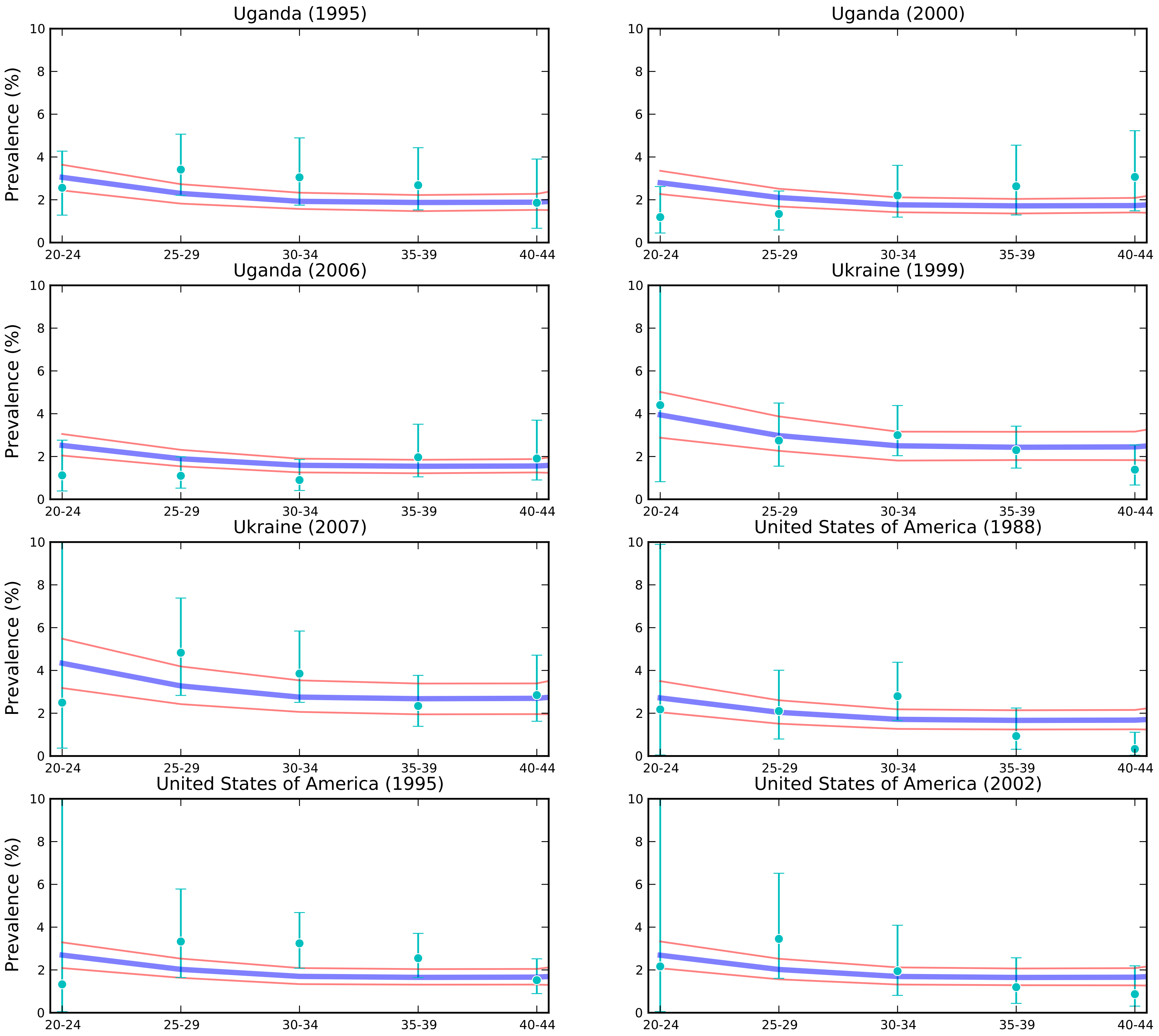
Uganda (1995)
Uganda (2000)
Uganda (2006)
Ukraine (1999)
Ukraine (2007)
United States of America (1988)
United States of America (1995)
United States of America (2002)
Prevalence (%)
0
2
4
6
8
10
20-24
25-29
30-34
35-39
40-44

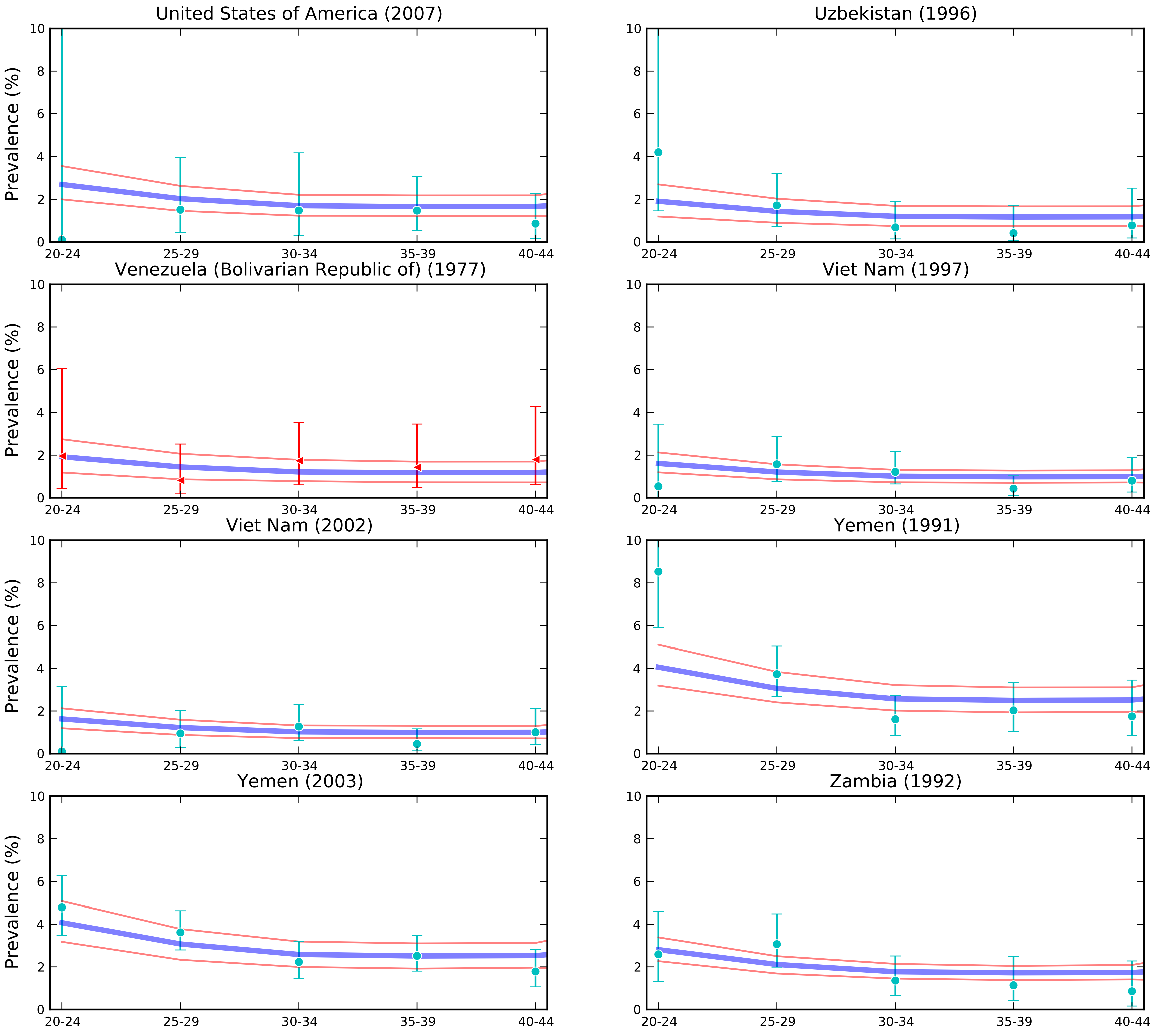
United States of America (2007)
Uzbekistan (1996)
Venezuela (Bolivarian Republic of) (1977)
Viet Nam (1997)
Viet Nam (2002)
Yemen (1991)
Yemen (2003)
Zambia (1992)
Prevalence (%)
0
2
4
6
8
10
20-24
25-29
30-34
35-39
40-44

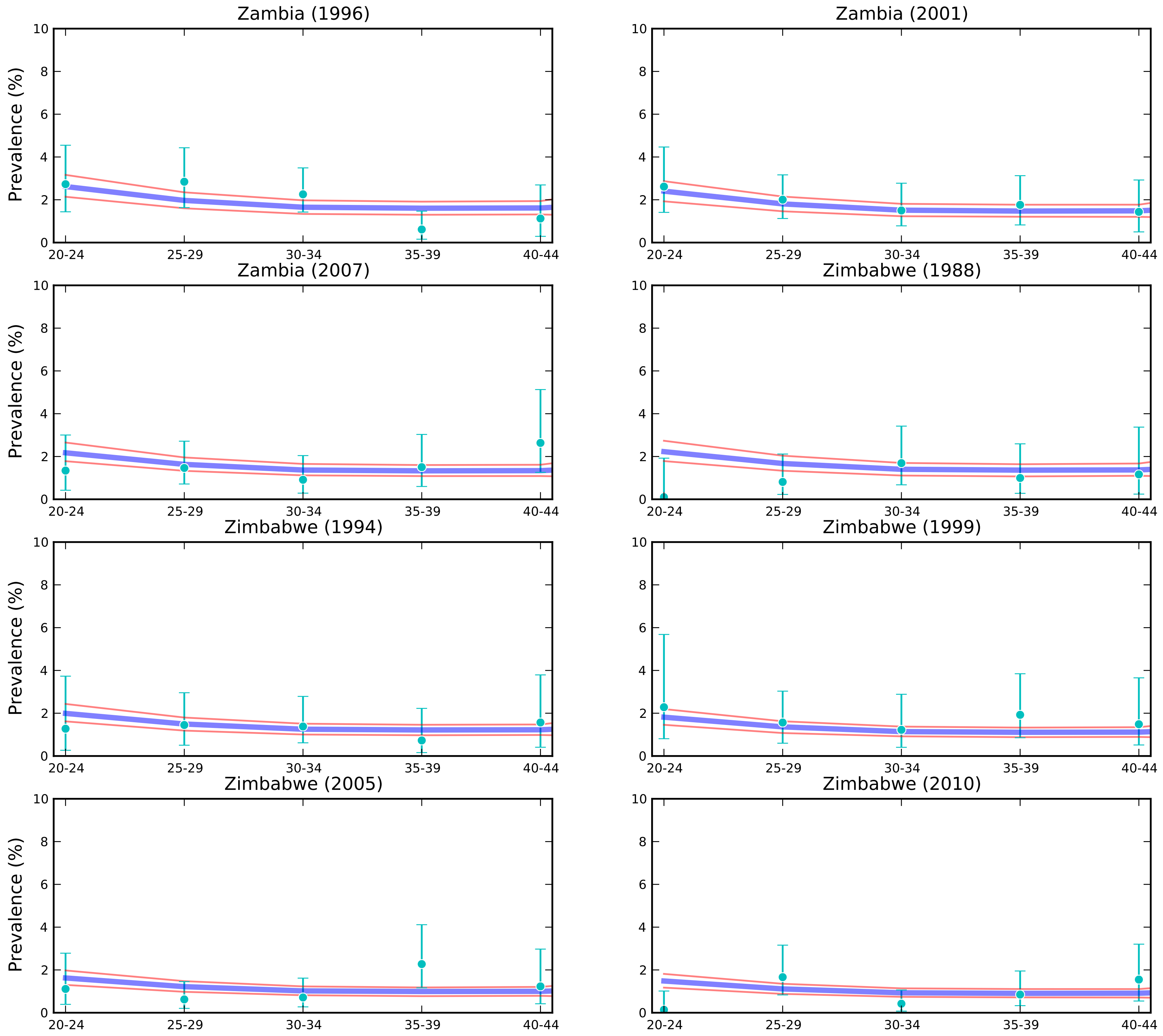

Zambia (1996)
Zambia (2001)
Zambia (2007)
Zimbabwe (1988)
Zimbabwe (1994)
Zimbabwe (1999)
Zimbabwe (2005)
Zimbabwe (2010)
Prevalence (%)
0
2
4
6
8
10
20-24
25-29
30-34
35-39
40-44

Figure E: Prevalence of secondary infertility by age group, shown for each country-year of data on following pages. Legend for plots is given below.

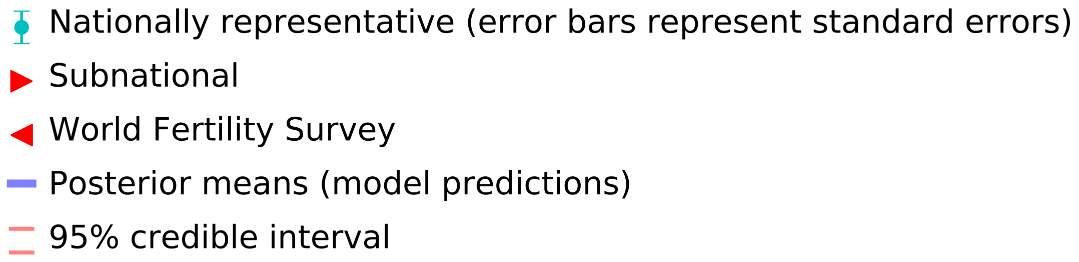

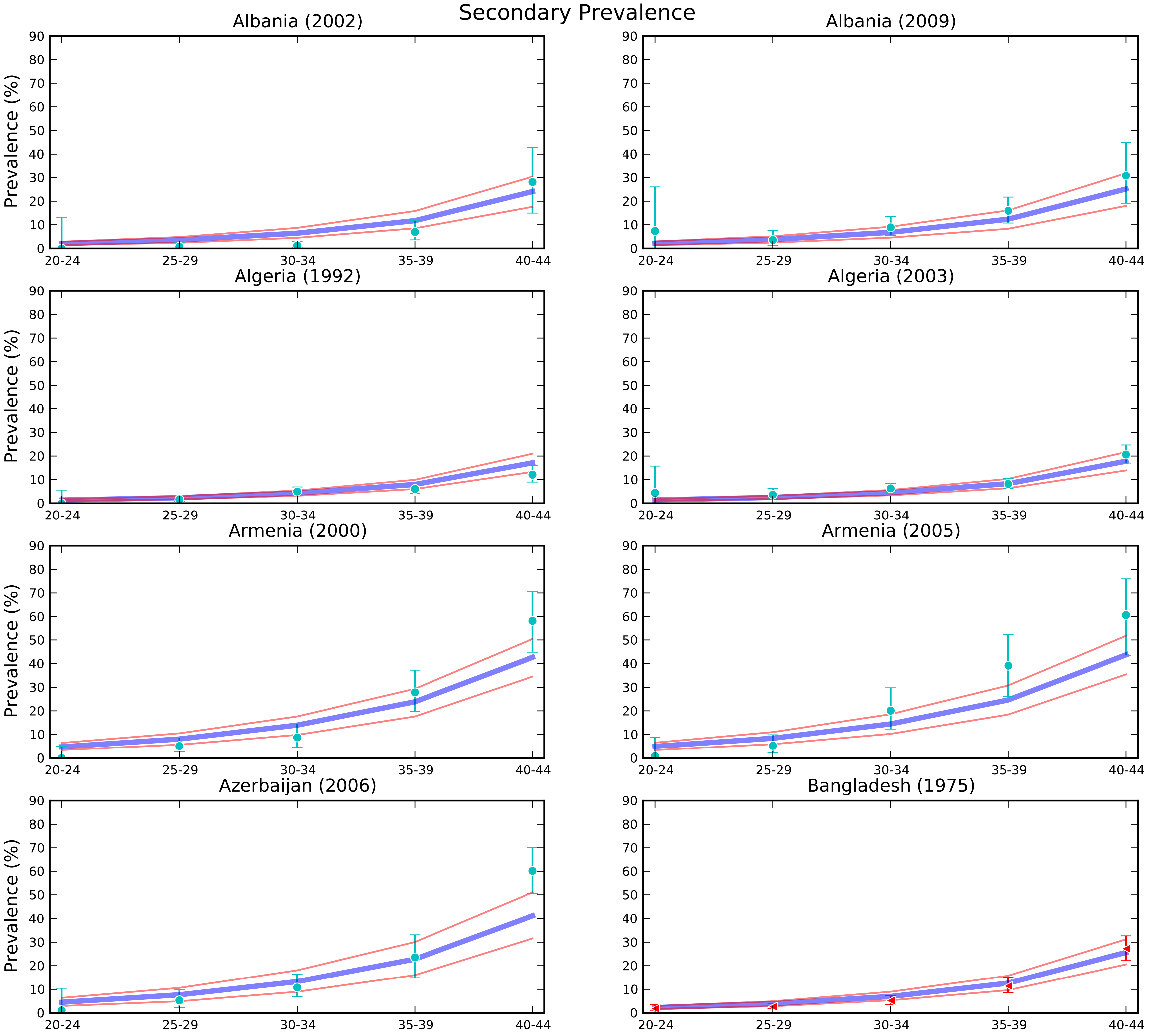
Secondary Prevalence
Albania (2002)
Albania (2009)
Algeria (1992)
Algeria (2003)
Armenia (2000)
Armenia (2005)
Azerbaijan (2006)
Bangladesh (1975)
Prevalence (%)
0
10
20
30
40
50
60
70
80
90
20-24
25-29
30-34
35-39
40-44

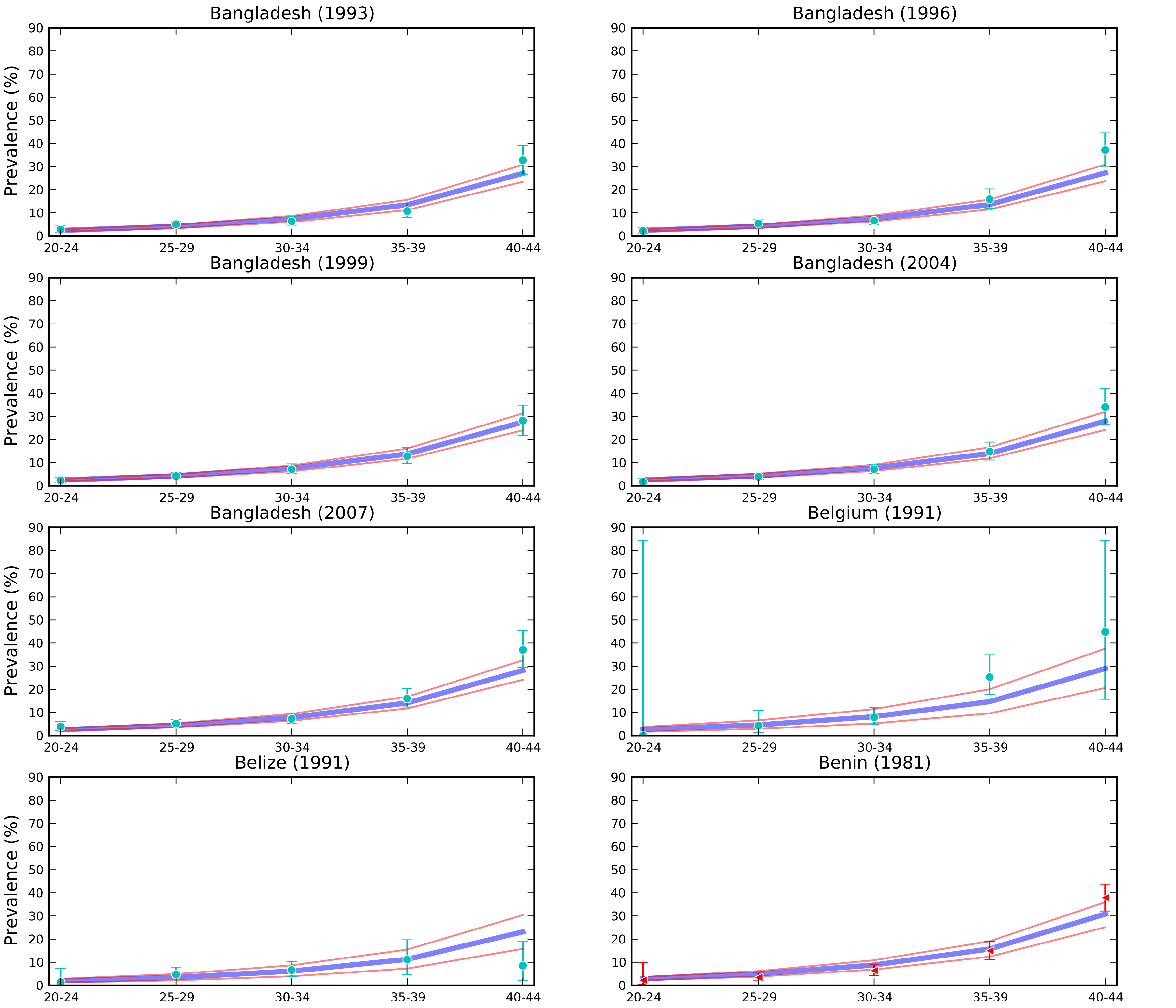
Bangladesh (1993)
Bangladesh (1996)
Bangladesh (1999)
Bangladesh (2004)
Bangladesh (2007)
Belgium (1991)
Belize (1991)
Benin (1981)
Prevalence (%)
0
10
20
30
40
50
60
70
80
90
20-24
25-29
30-34
35-39
40-44

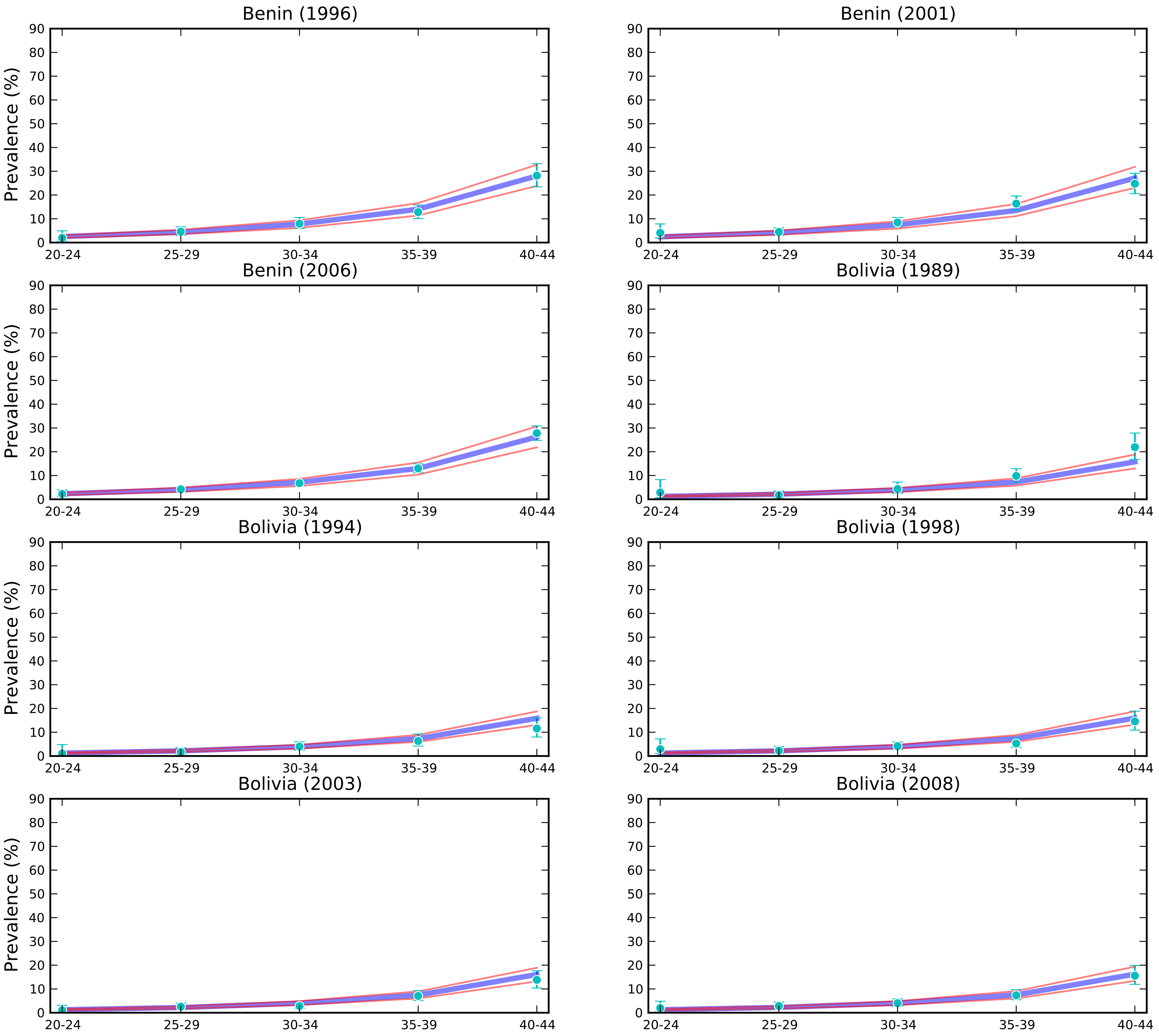
Benin (1996)
Benin (2001)
Benin (2006)
Bolivia (1989)
Bolivia (1994)
Bolivia (1998)
Bolivia (2003)
Bolivia (2008)
Prevalence (%)
0
10
20
30
40
50
60
70
80
90
20-24
25-29
30-34
35-39
40-44

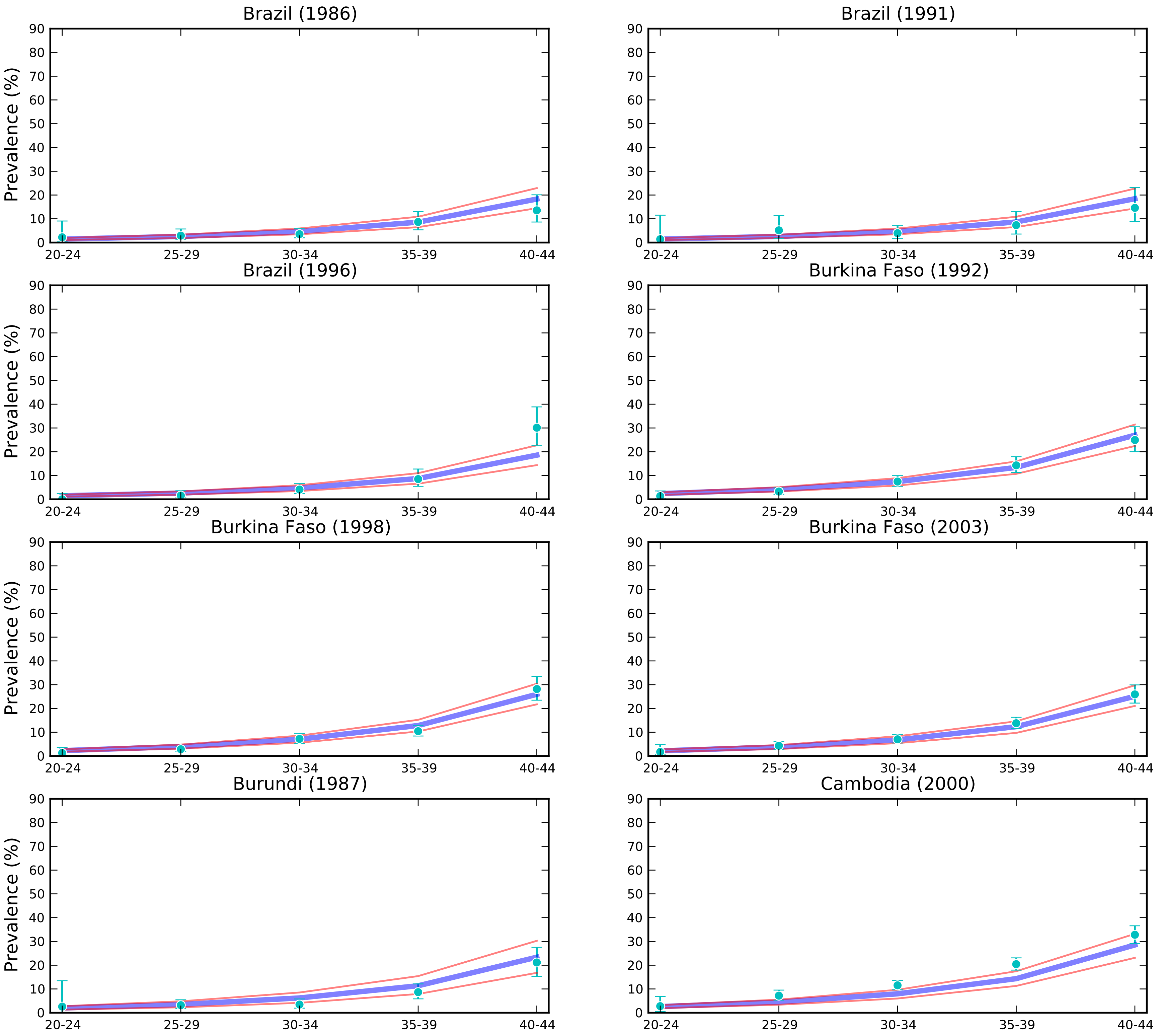
Brazil (1986)
Brazil (1991)
Brazil (1996)
Burkina Faso (1992)
Burkina Faso (1998)
Burkina Faso (2003)
Burundi (1987)
Cambodia (2000)
Prevalence (%)
0
10
20
30
40
50
60
70
80
90
20-24
25-29
30-34
35-39
40-44

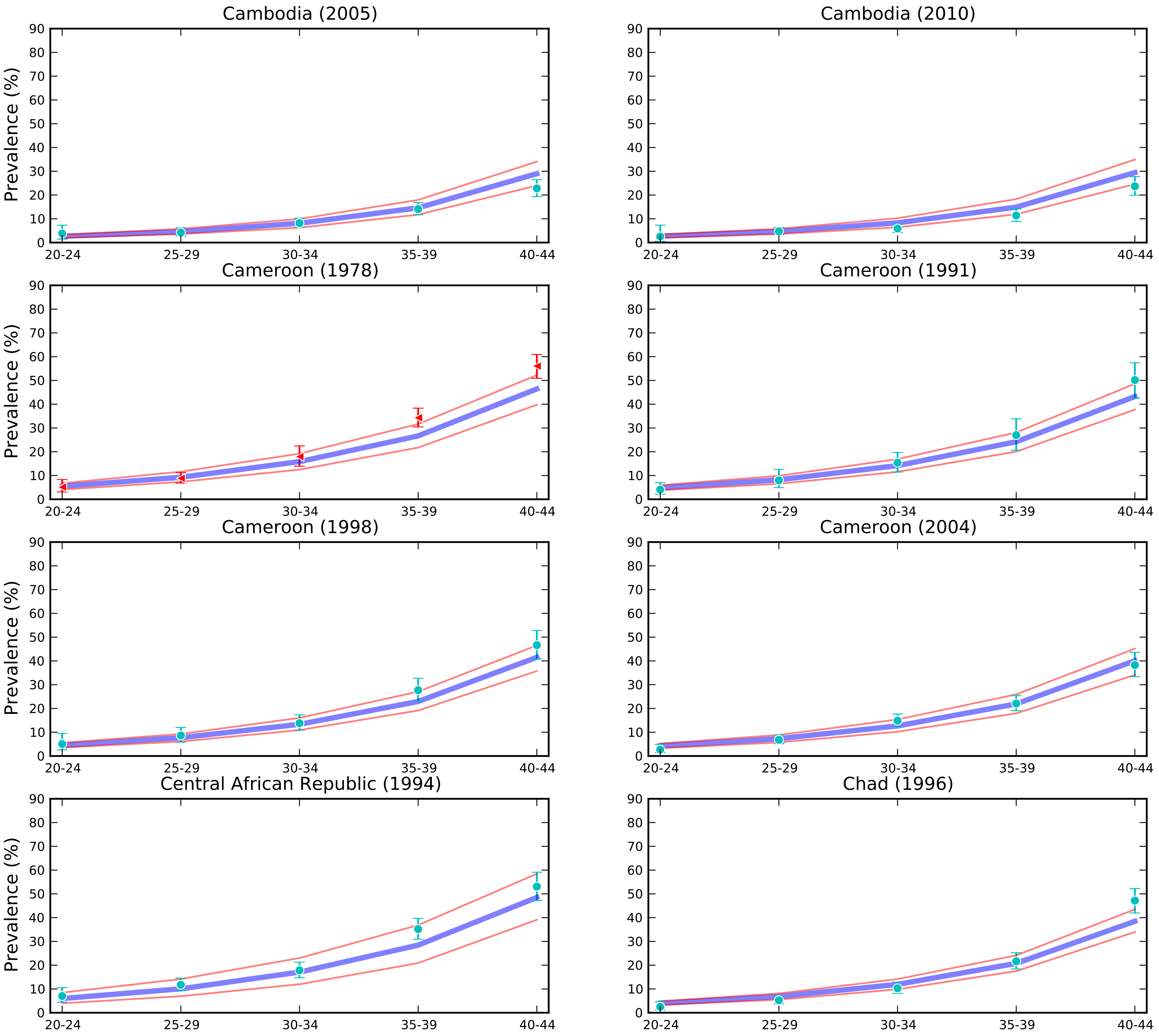
Cambodia (2005)
Cambodia (2010)
Cameroon (1978)
Cameroon (1991)
Cameroon (1998)
Cameroon (2004)
Central African Republic (1994)
Chad (1996)
Prevalence (%)
0
10
20
30
40
50
60
70
80
90
20-24
25-29
30-34
35-39
40-44

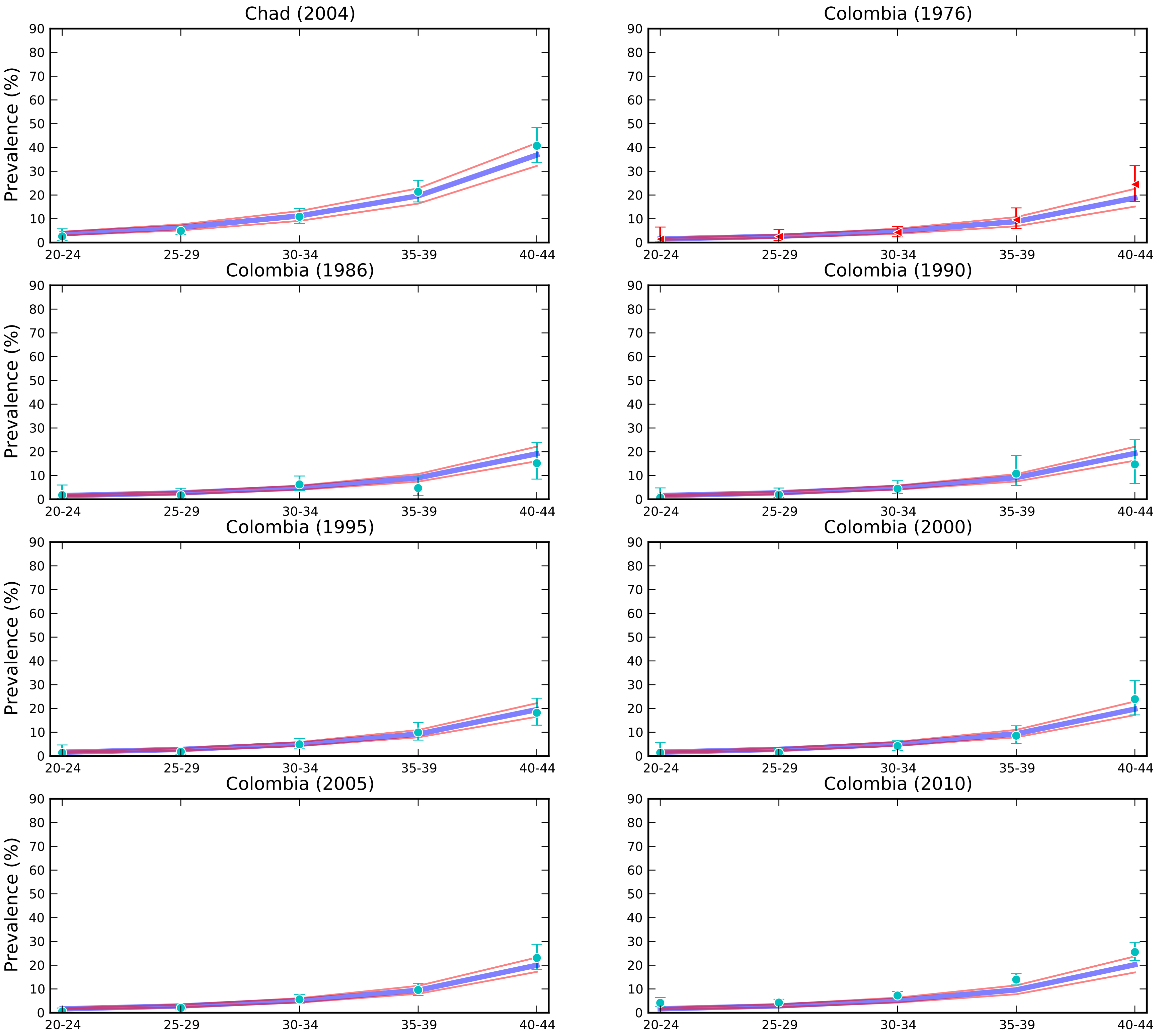

Chad (2004)
Colombia (1976)
Colombia (1986)
Colombia (1990)
Colombia (1995)
Colombia (2000)
Colombia (2005)
Colombia (2010)
Prevalence (%)
0
10
20
30
40
50
60
70
80
90
20-24
25-29
30-34
35-39
40-44

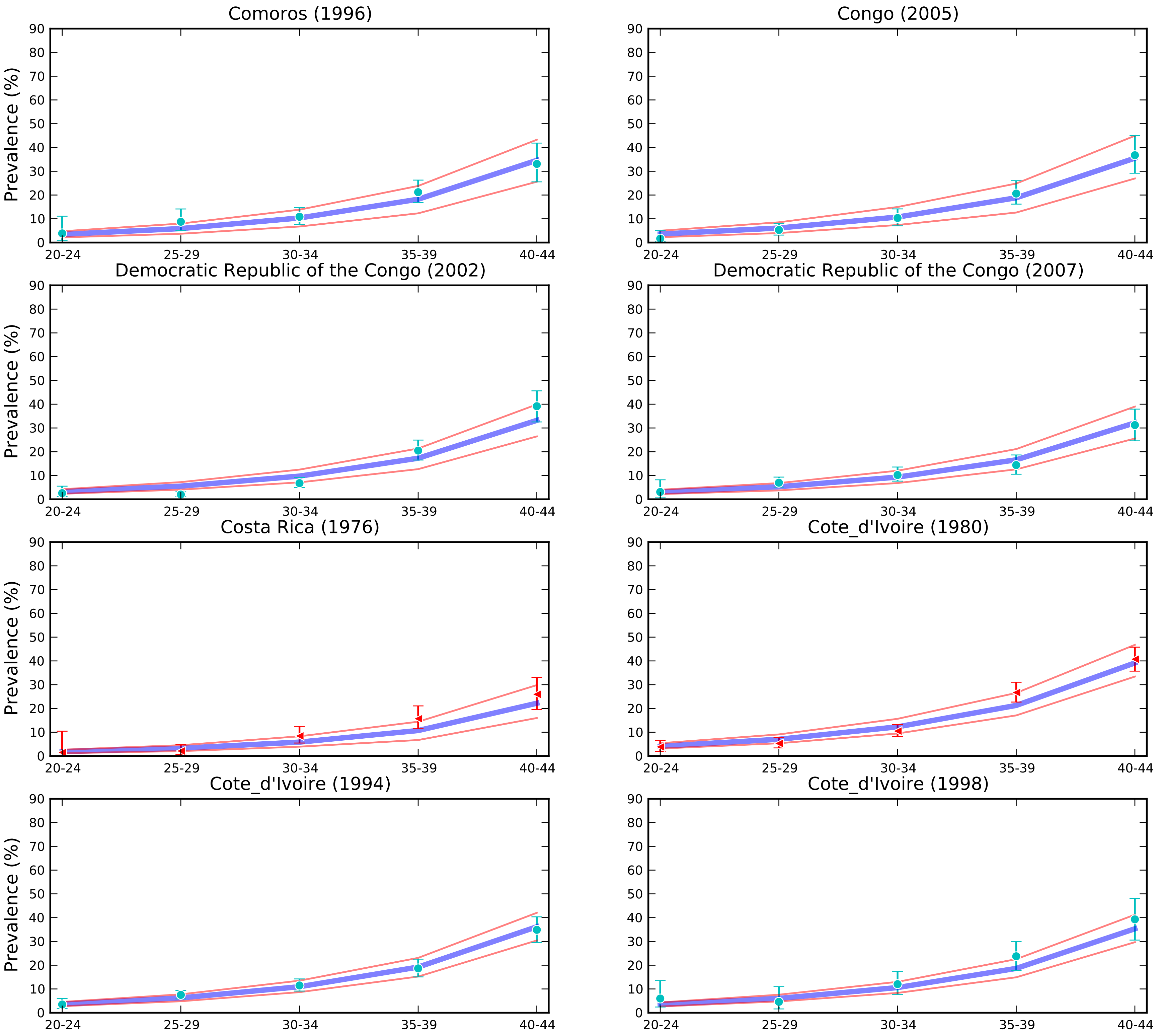

Comoros (1996)
Congo (2005)
Democratic Republic of the Congo (2002)
Democratic Republic of the Congo (2007)
Costa Rica (1976)
Cote_d'Ivoire (1980)
Cote_d'Ivoire (1994)
Cote_d'Ivoire (1998)
Prevalence (%)
0 10 20 30 40 50 60 70 80 90
20-24 25-29 30-34 35-39 40-44

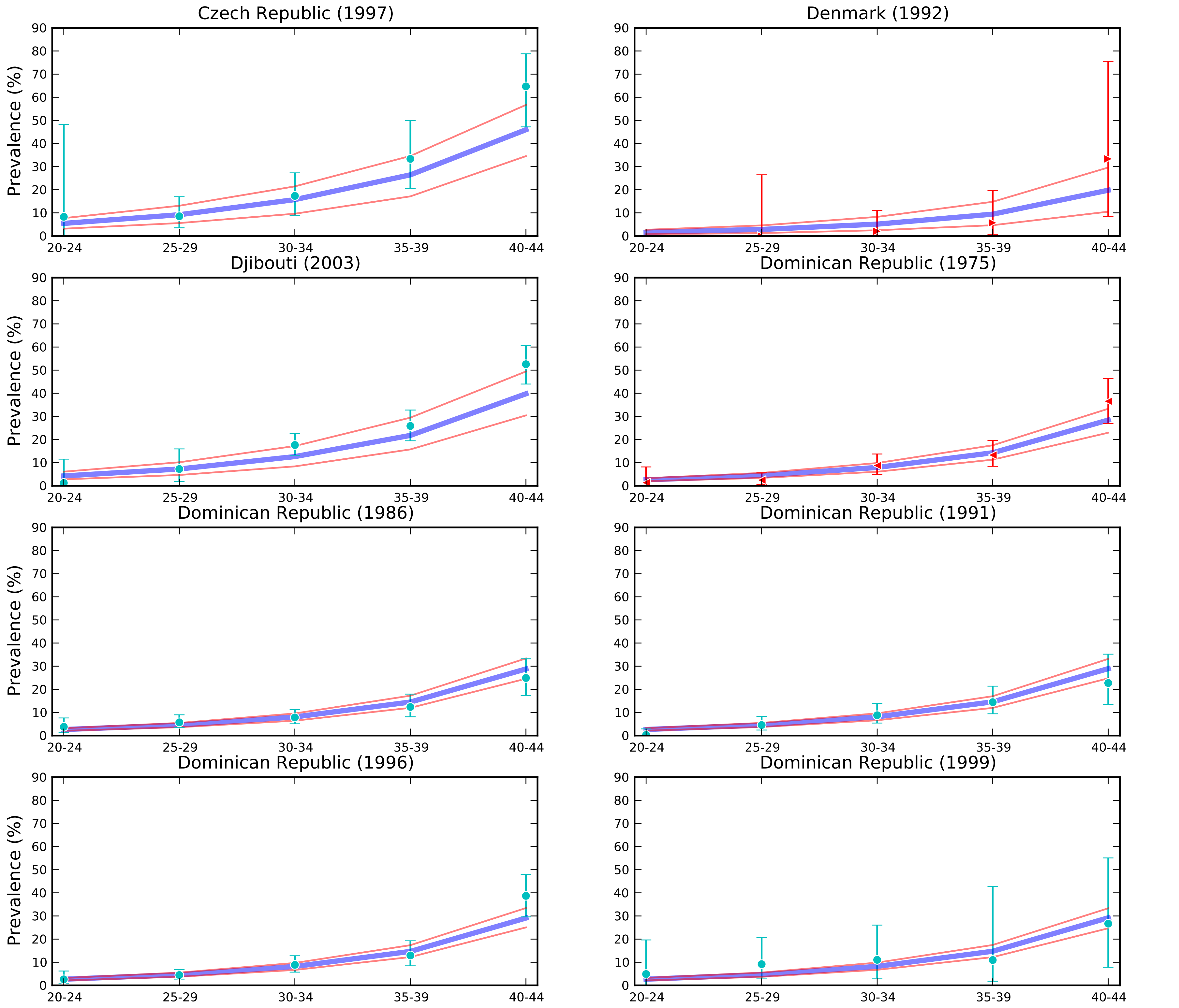
Czech Republic (1997)
Denmark (1992)
Djibouti (2003)
Dominican Republic (1975)
Dominican Republic (1986)
Dominican Republic (1991)
Dominican Republic (1996)
Dominican Republic (1999)
Prevalence (%)
0
10
20
30
40
50
60
70
80
90
20-24
25-29
30-34
35-39
40-44

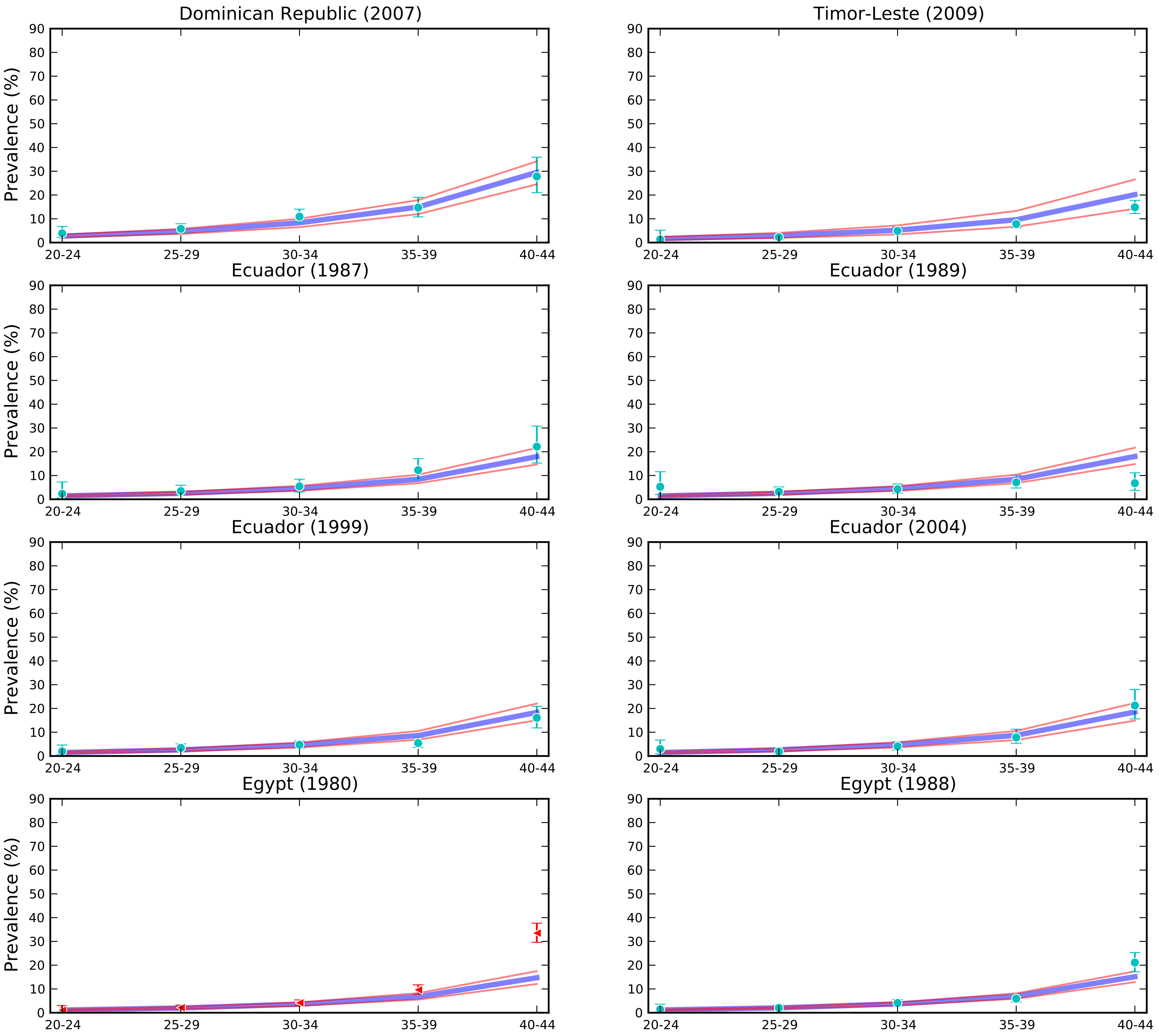
Dominican Republic (2007)
Timor-Leste (2009)
Ecuador (1987)
Ecuador (1989)
Ecuador (1999)
Ecuador (2004)
Egypt (1980)
Egypt (1988)
Prevalence (%)
0
10
20
30
40
50
60
70
80
90
20-24
25-29
30-34
35-39
40-44

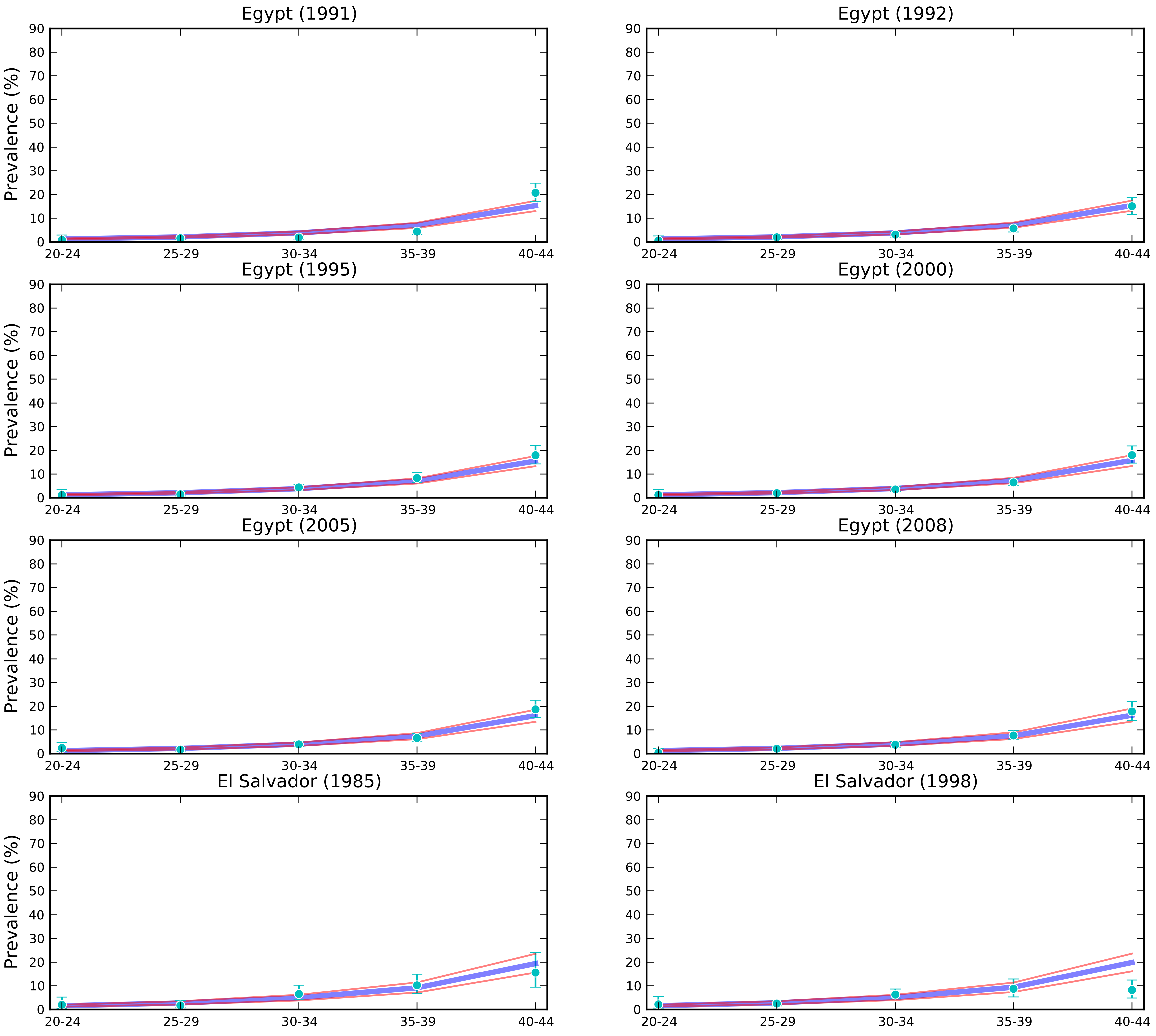

Egypt (1991)
Egypt (1992)
Egypt (1995)
Egypt (2000)
Egypt (2005)
Egypt (2008)
El Salvador (1985)
El Salvador (1998)
Prevalence (%)
0
10
20
30
40
50
60
70
80
90
20-24
25-29
30-34
35-39
40-44

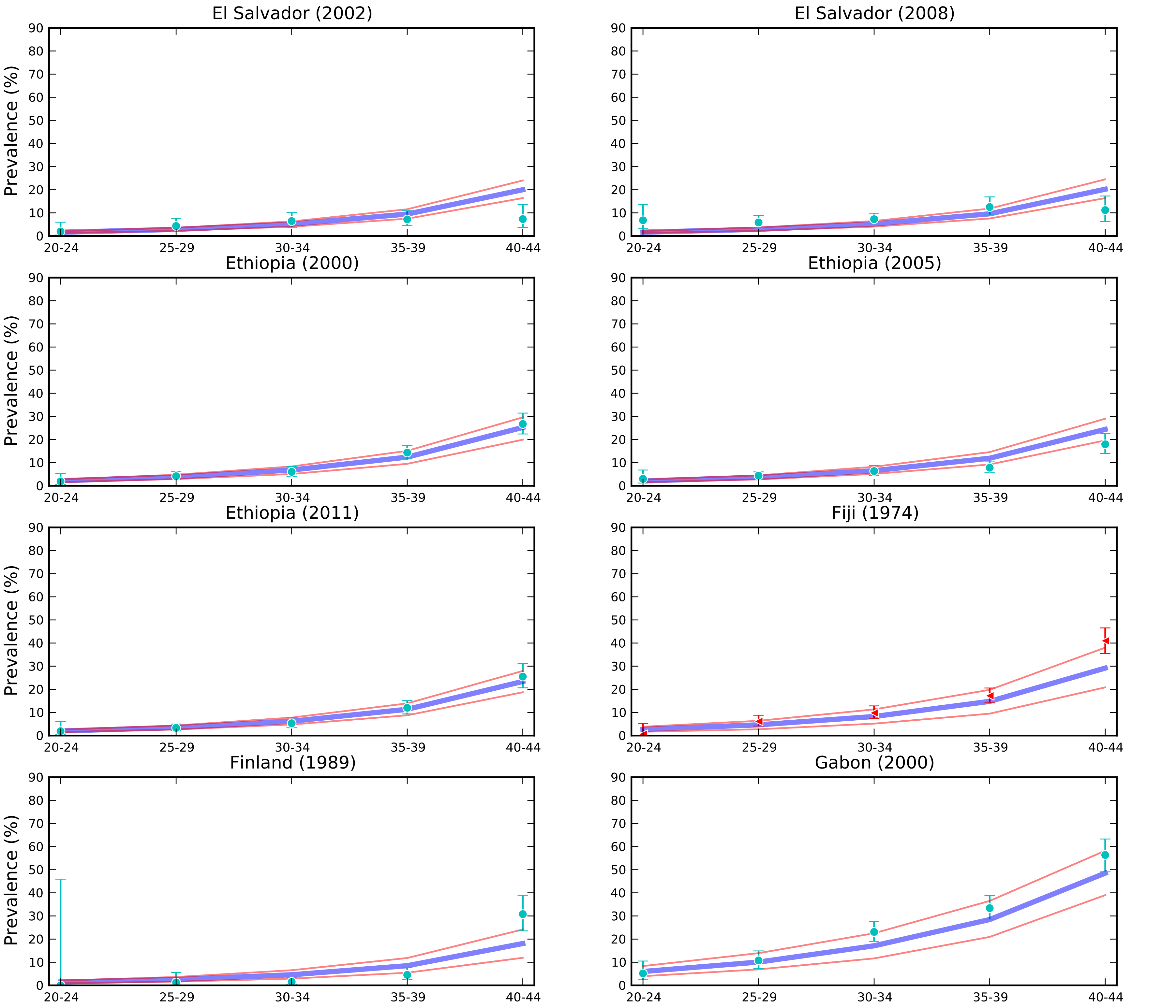
El Salvador (2002)
El Salvador (2008)
Ethiopia (2000)
Ethiopia (2005)
Ethiopia (2011)
Fiji (1974)
Finland (1989)
Gabon (2000)
Prevalence (%)
0
10
20
30
40
50
60
70
80
90
20-24
25-29
30-34
35-39
40-44

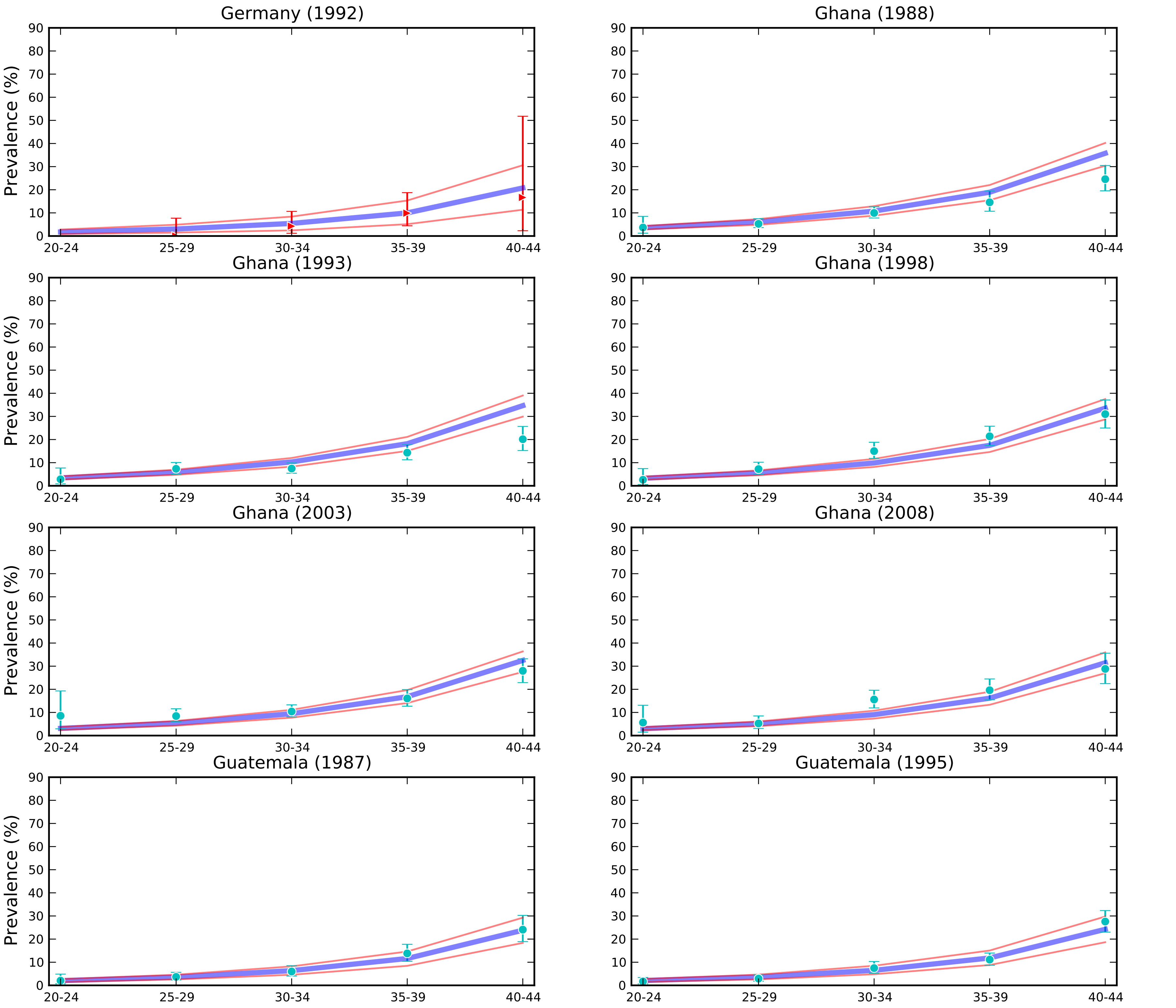
Germany (1992)
Ghana (1988)
Ghana (1993)
Ghana (1998)
Ghana (2003)
Ghana (2008)
Guatemala (1987)
Guatemala (1995)
Prevalence (%)
0 10 20 30 40 50 60 70 80 90
20-24 25-29 30-34 35-39 40-44

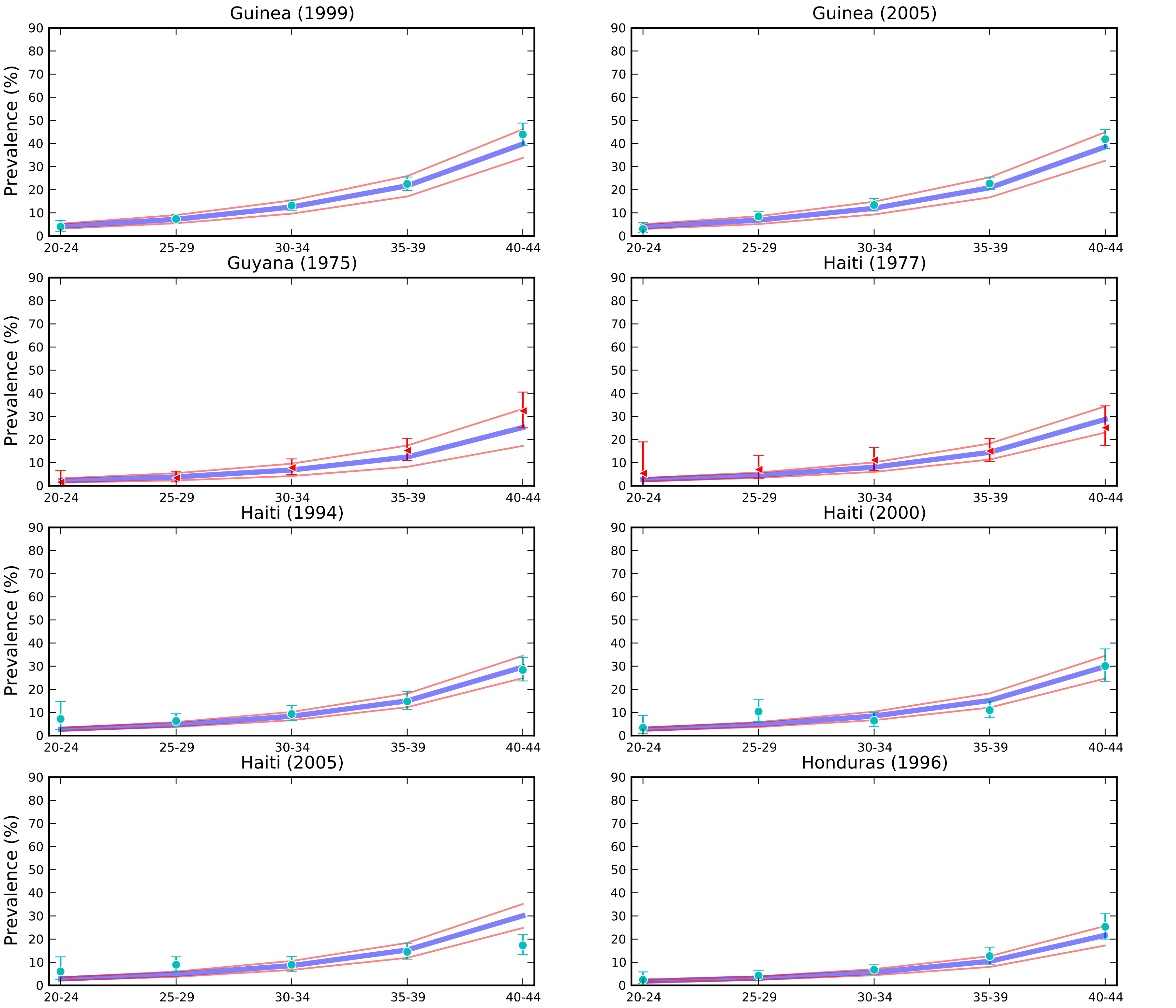
Guinea (1999)
Guinea (2005)
Guyana (1975)
Haiti (1977)
Haiti (1994)
Haiti (2000)
Haiti (2005)
Honduras (1996)
Prevalence (%)
20-24
25-29
30-34
35-39
40-44

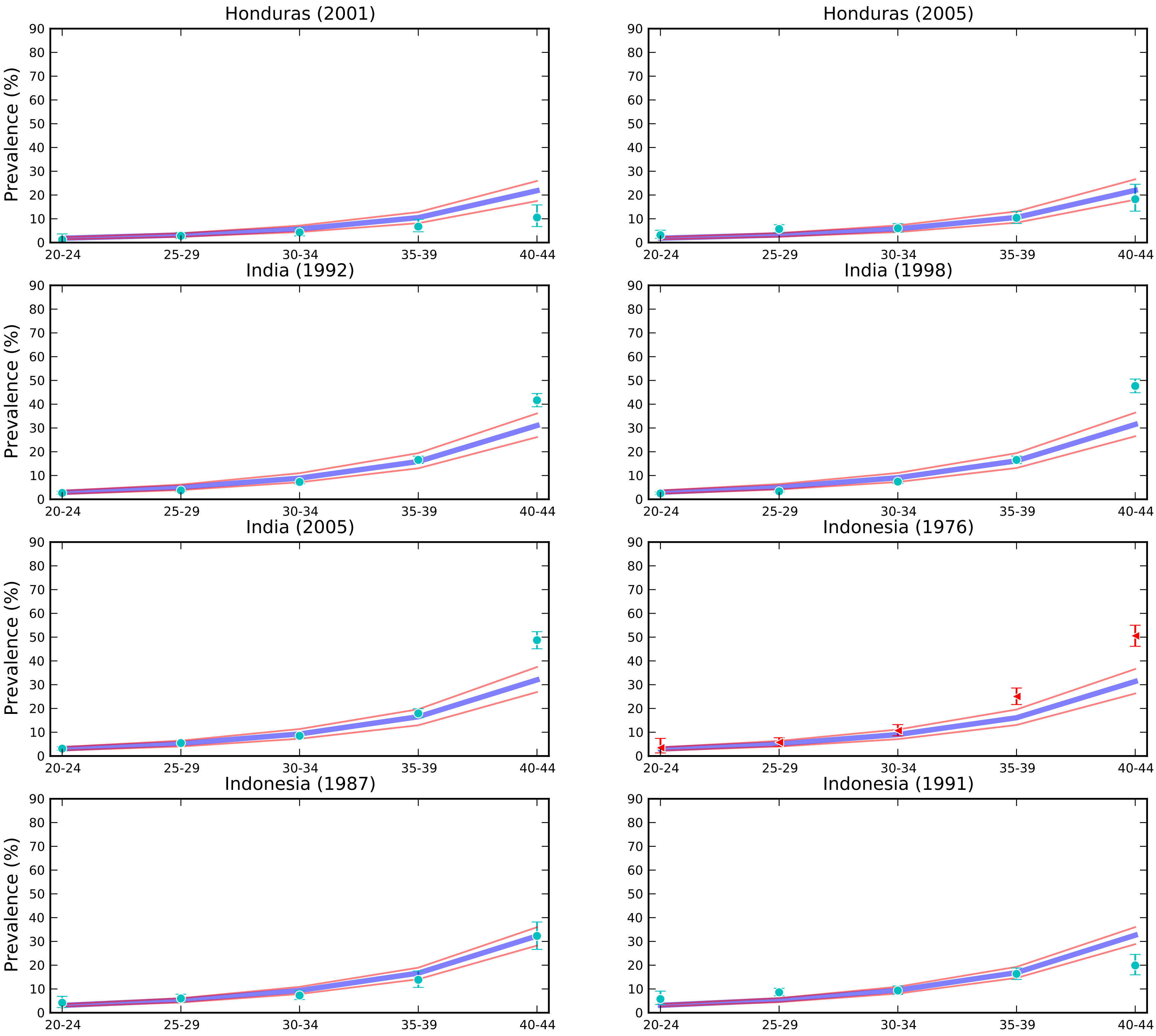
Honduras (2001)
Honduras (2005)
India (1992)
India (1998)
India (2005)
Indonesia (1976)
Indonesia (1987)
Indonesia (1991)
Prevalence (%)
0 10 20 30 40 50 60 70 80 90
20-24
25-29
30-34
35-39
40-44

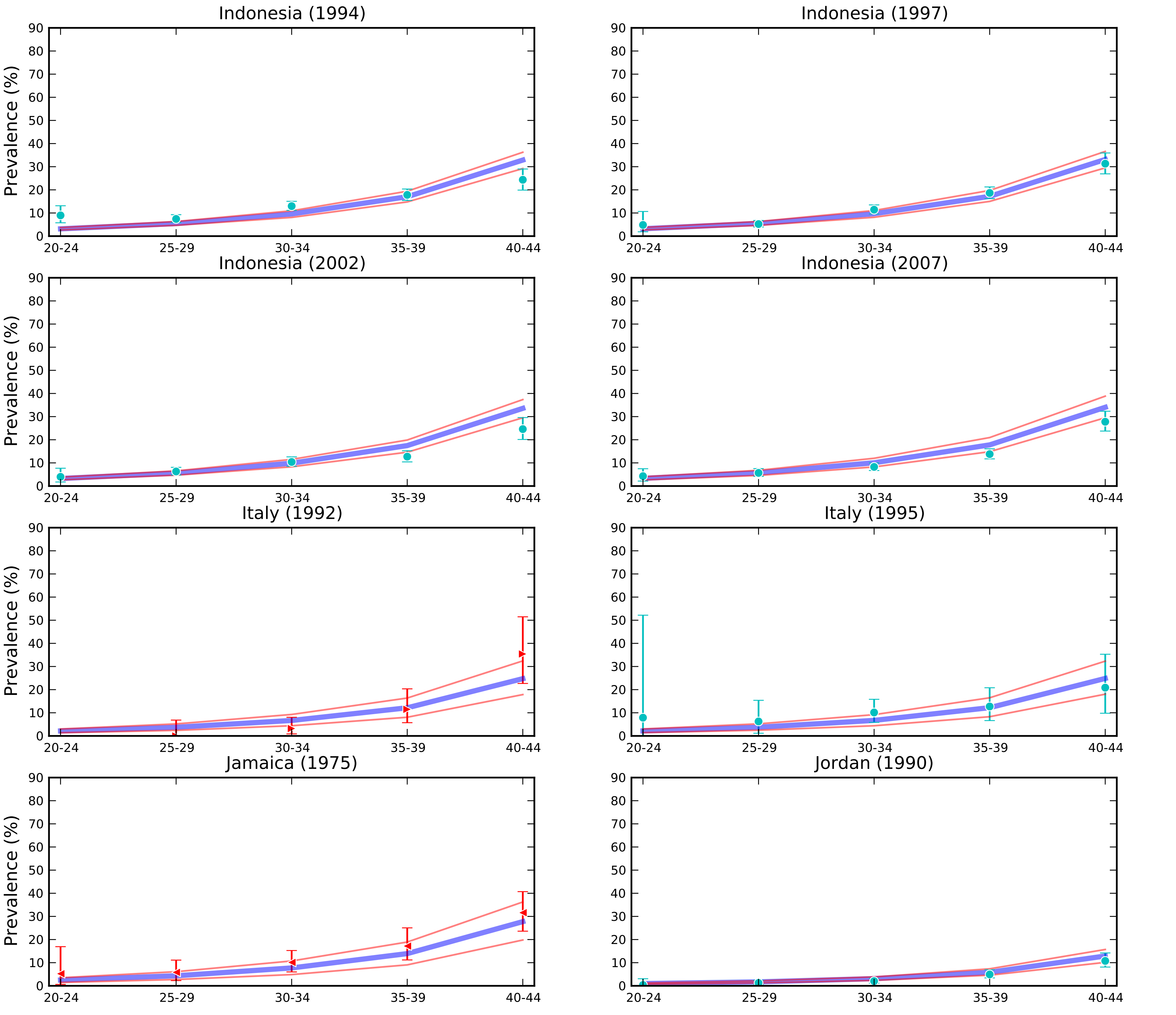
Indonesia (1994)
Indonesia (1997)
Indonesia (2002)
Indonesia (2007)
Italy (1992)
Italy (1995)
Jamaica (1975)
Jordan (1990)
Prevalence (%)
0
10
20
30
40
50
60
70
80
90
20-24
25-29
30-34
35-39
40-44

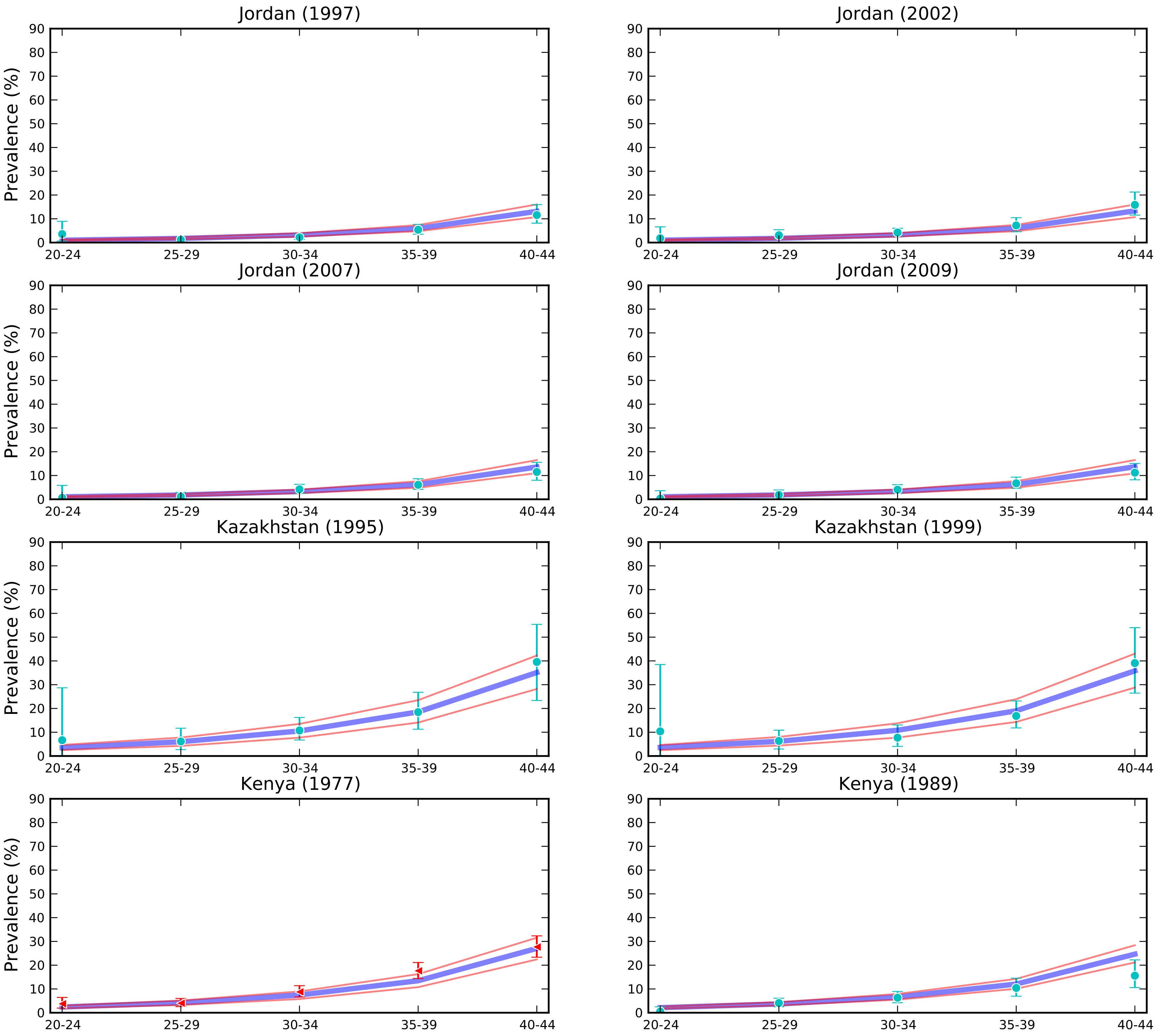
Jordan (1997)
Jordan (2002)
Jordan (2007)
Jordan (2009)
Kazakhstan (1995)
Kazakhstan (1999)
Kenya (1977)
Kenya (1989)
Prevalence (%)
0
10
20
30
40
50
60
70
80
90
20-24
25-29
30-34
35-39
40-44

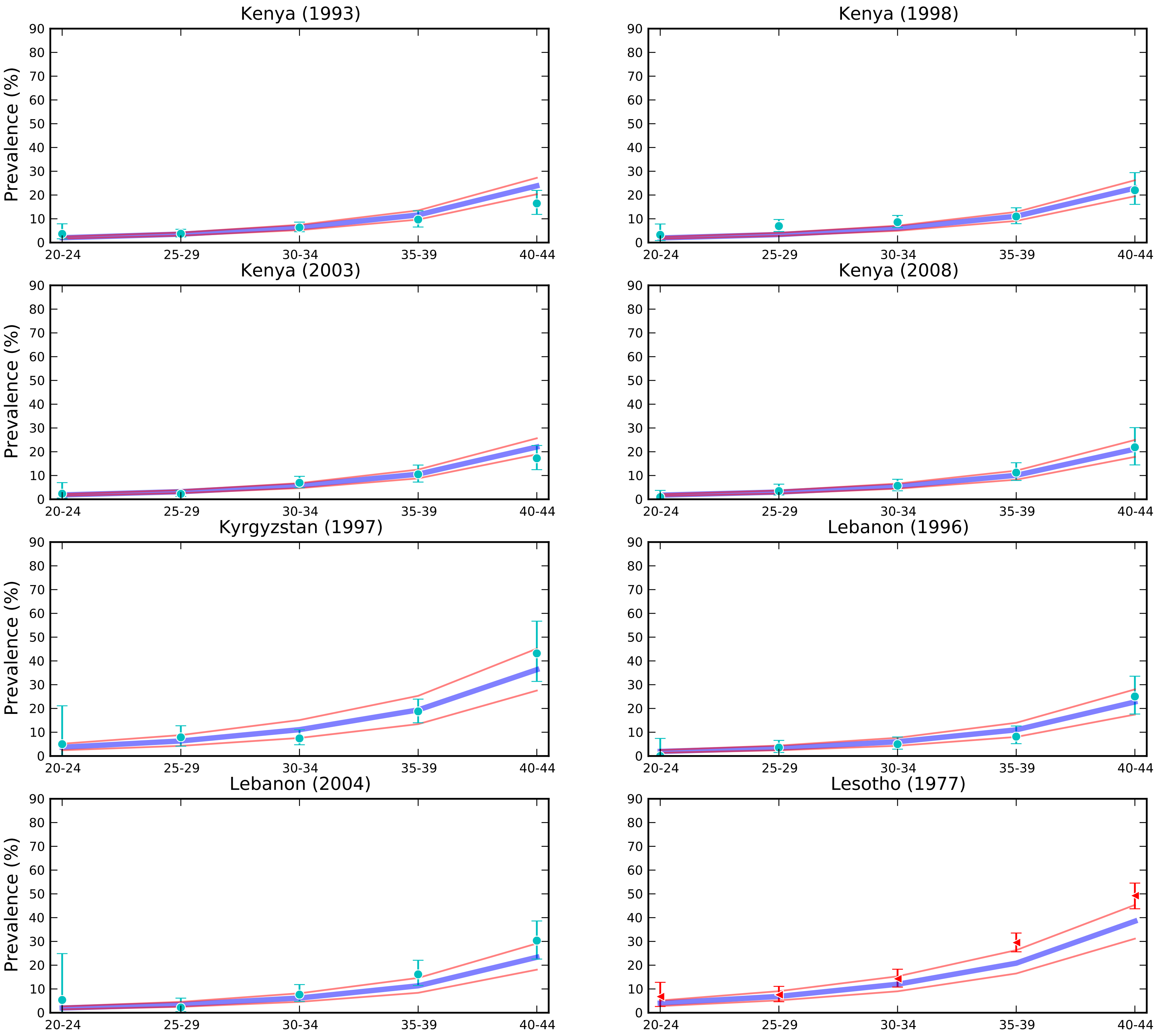
Kenya (1993)
Kenya (1998)
Kenya (2003)
Kenya (2008)
Kyrgyzstan (1997)
Lebanon (1996)
Lebanon (2004)
Lesotho (1977)
Prevalence (%)
0 10 20 30 40 50 60 70 80 90
20-24 25-29 30-34 35-39 40-44

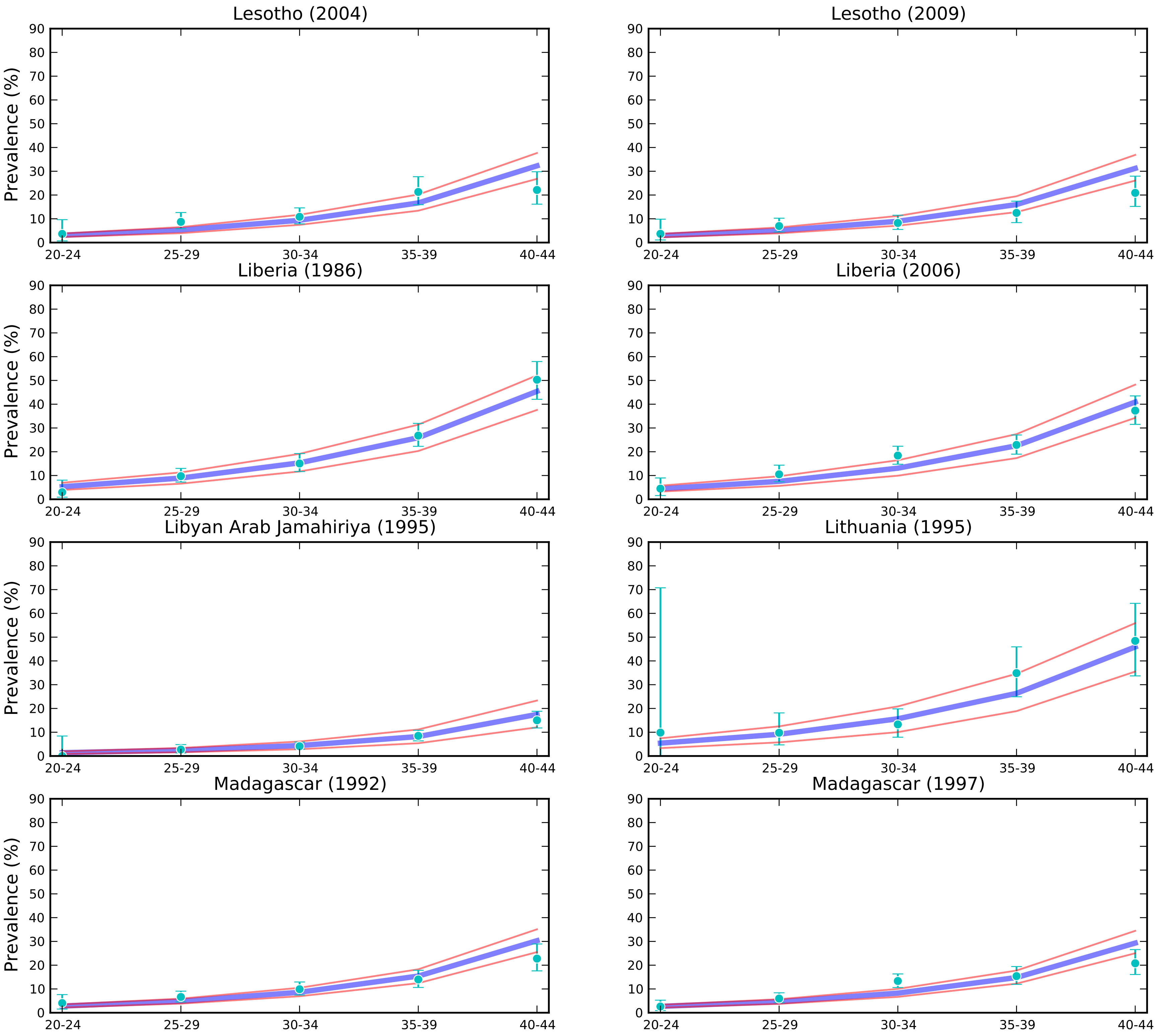

Lesotho (2004)
Lesotho (2009)
Liberia (1986)
Liberia (2006)
Libyan Arab Jamahiriya (1995)
Lithuania (1995)
Madagascar (1992)
Madagascar (1997)
Prevalence (%)
0
10
20
30
40
50
60
70
80
90
20-24
25-29
30-34
35-39
40-44

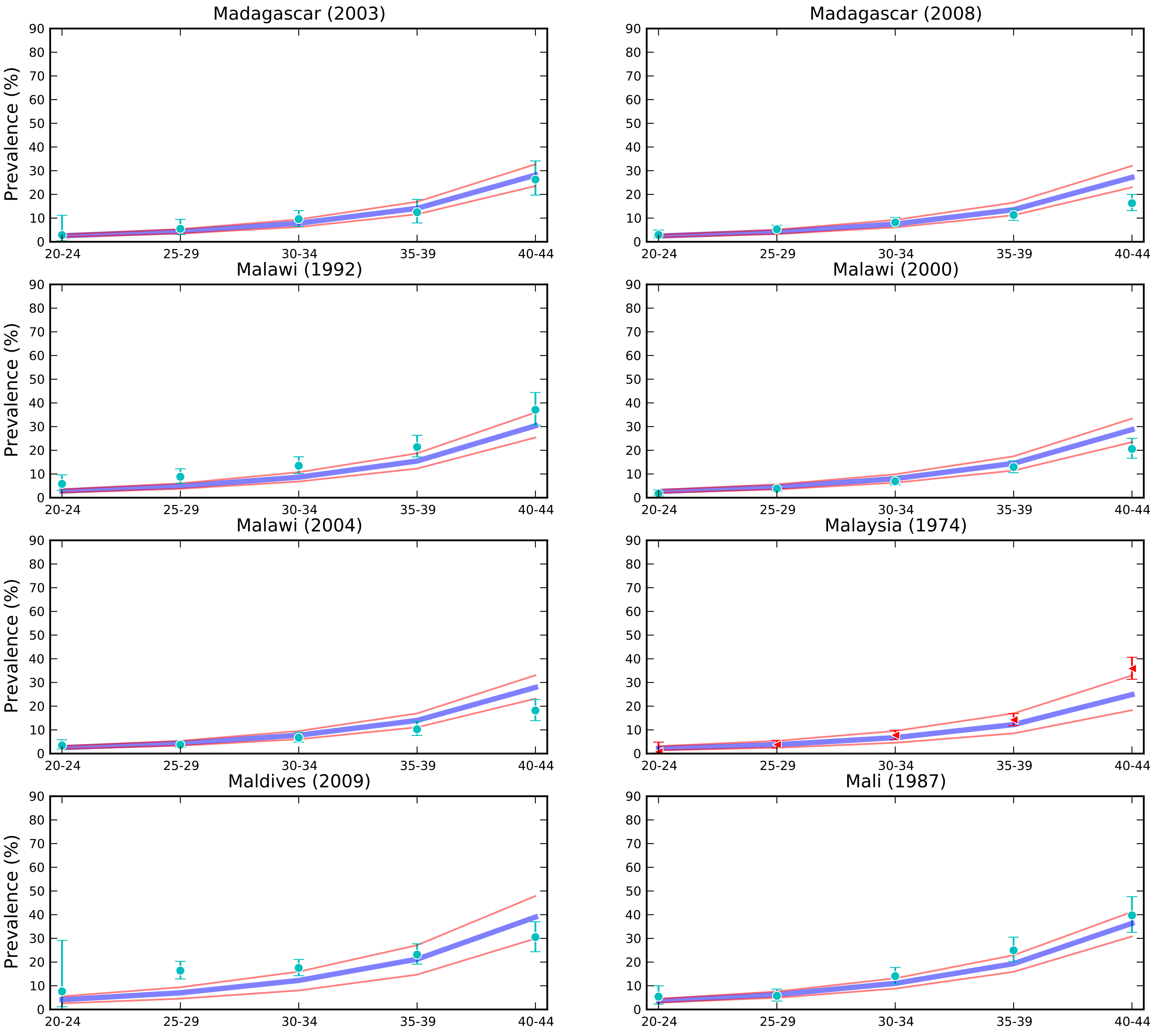
Madagascar (2003)
Madagascar (2008)
Malawi (1992)
Malawi (2000)
Malawi (2004)
Malaysia (1974)
Maldives (2009)
Mali (1987)
Prevalence (%)
0
10
20
30
40
50
60
70
80
90
20-24
25-29
30-34
35-39
40-44

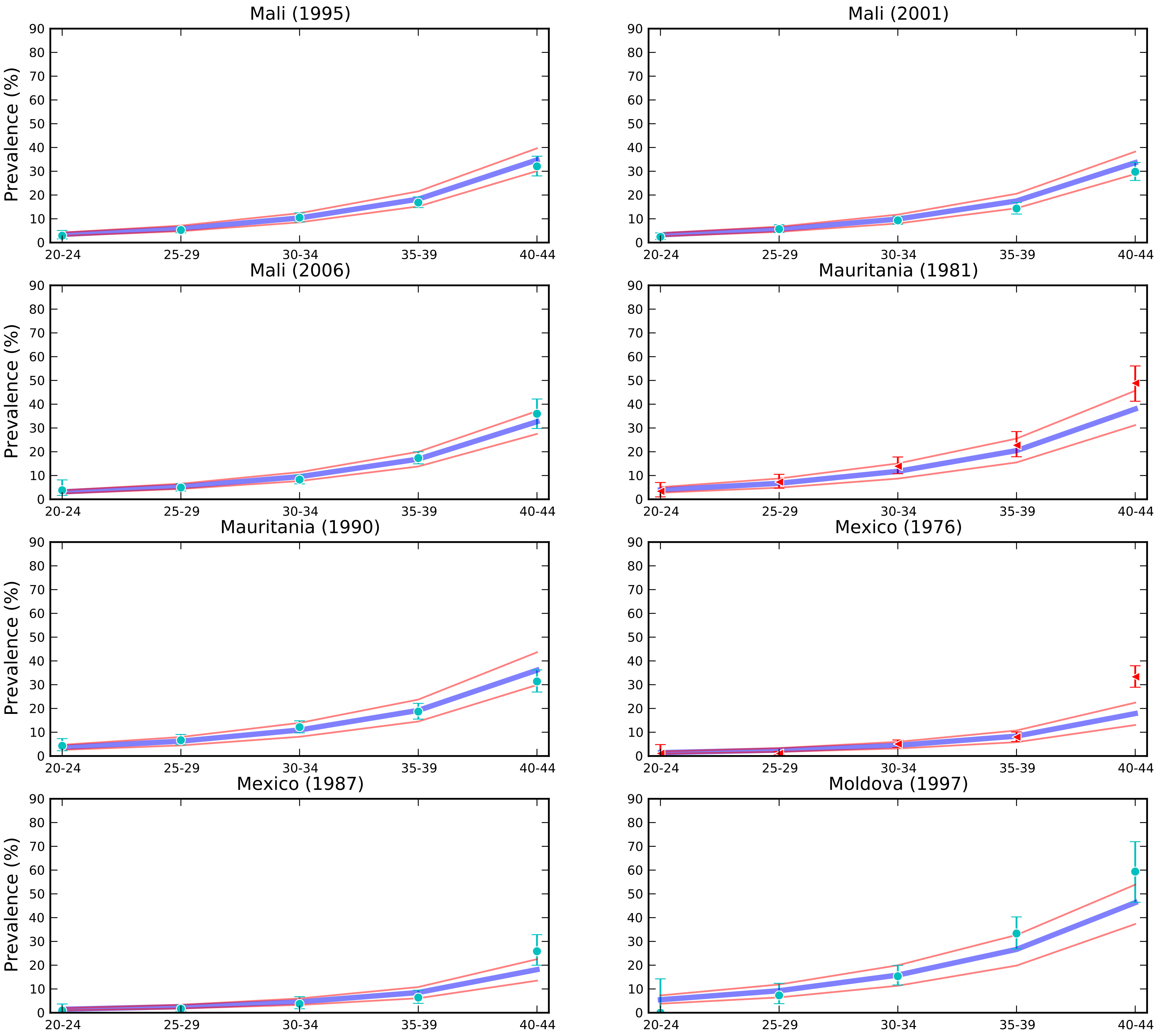
Mali (1995)
Mali (2001)
Mali (2006)
Mauritania (1981)
Mauritania (1990)
Mexico (1976)
Mexico (1987)
Moldova (1997)
Prevalence (%)
0
10
20
30
40
50
60
70
80
90
20-24
25-29
30-34
35-39
40-44

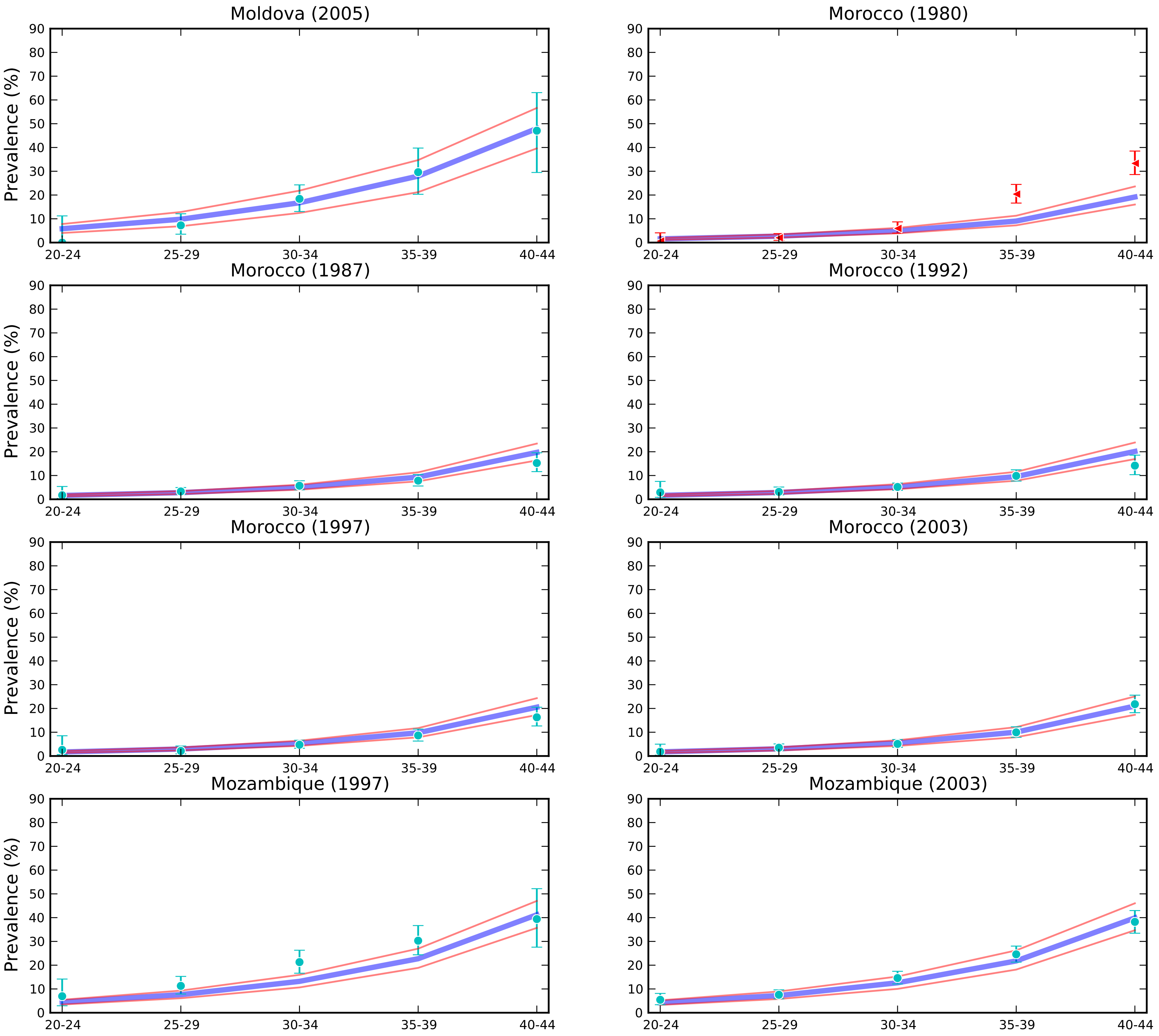

Moldova (2005)
Morocco (1980)
Morocco (1987)
Morocco (1992)
Morocco (1997)
Morocco (2003)
Mozambique (1997)
Mozambique (2003)
Prevalence (%)
0
10
20
30
40
50
60
70
80
90
20-24
25-29
30-34
35-39
40-44

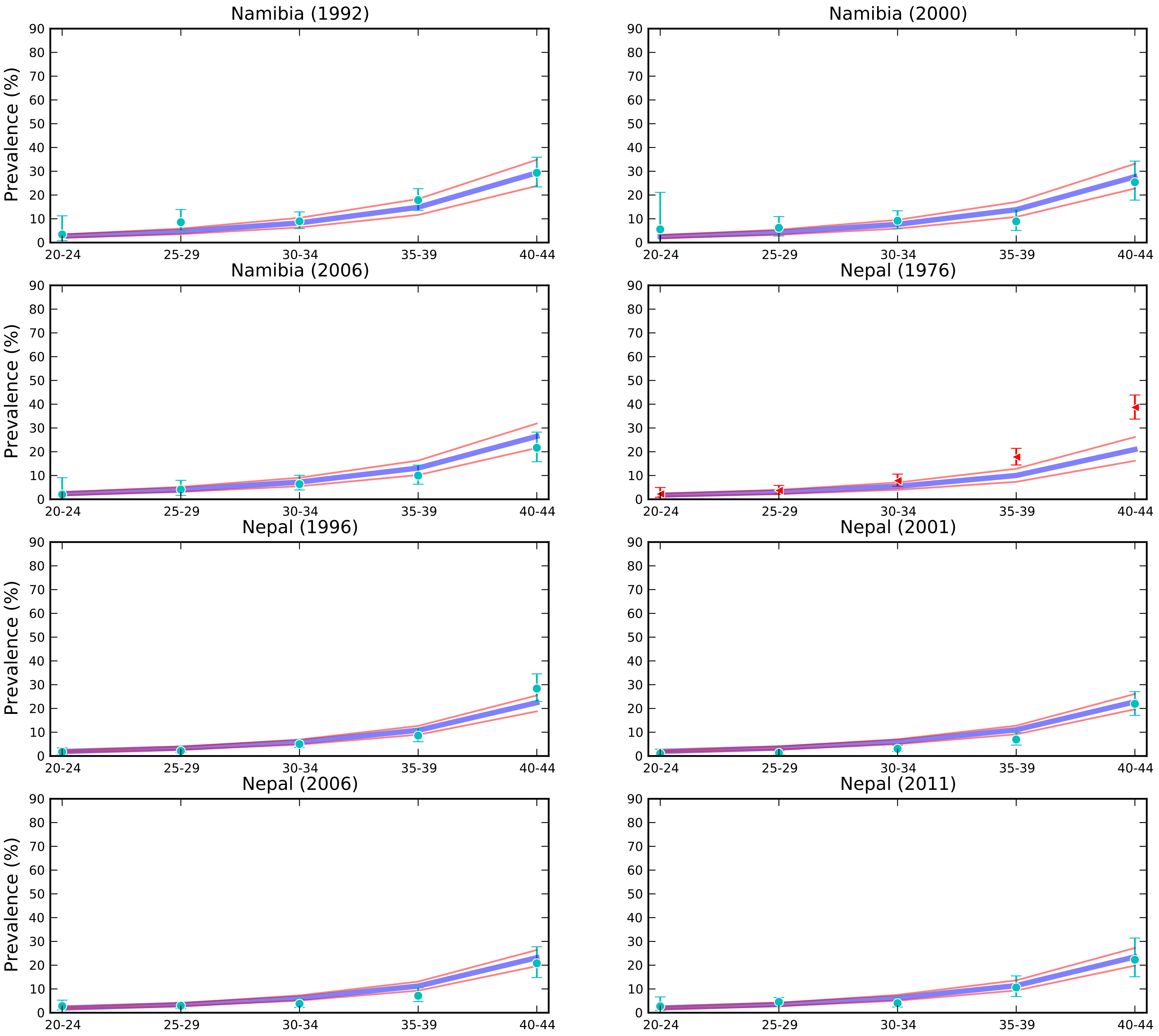
Namibia (1992)
Namibia (2000)
Namibia (2006)
Nepal (1976)
Nepal (1996)
Nepal (2001)
Nepal (2006)
Nepal (2011)
Prevalence (%)
0
10
20
30
40
50
60
70
80
90
20-24
25-29
30-34
35-39
40-44

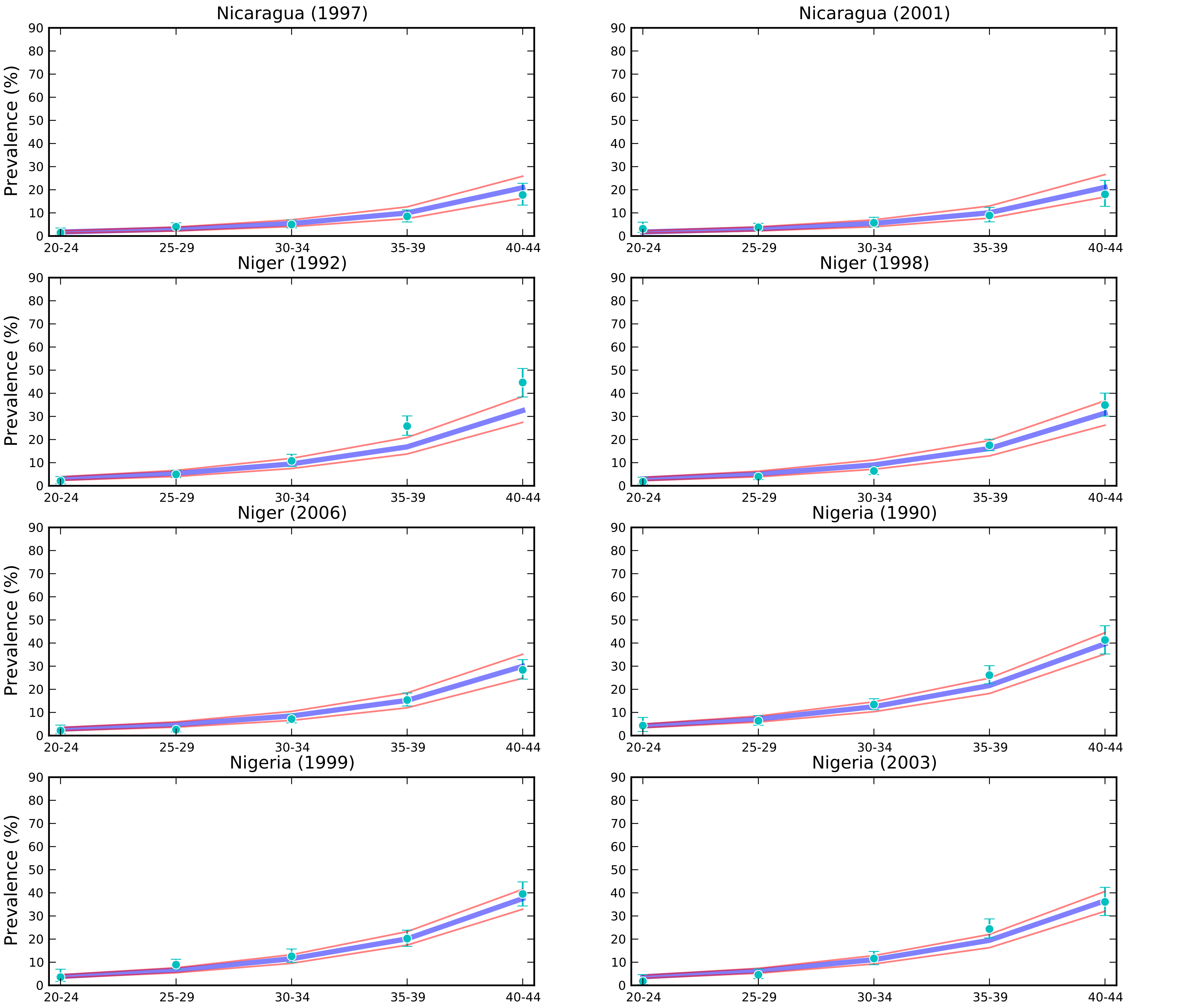

Nicaragua (1997)
Nicaragua (2001)
Niger (1992)
Niger (1998)
Niger (2006)
Nigeria (1990)
Nigeria (1999)
Nigeria (2003)
Prevalence (%)
0
10
20
30
40
50
60
70
80
90
20-24
25-29
30-34
35-39
40-44

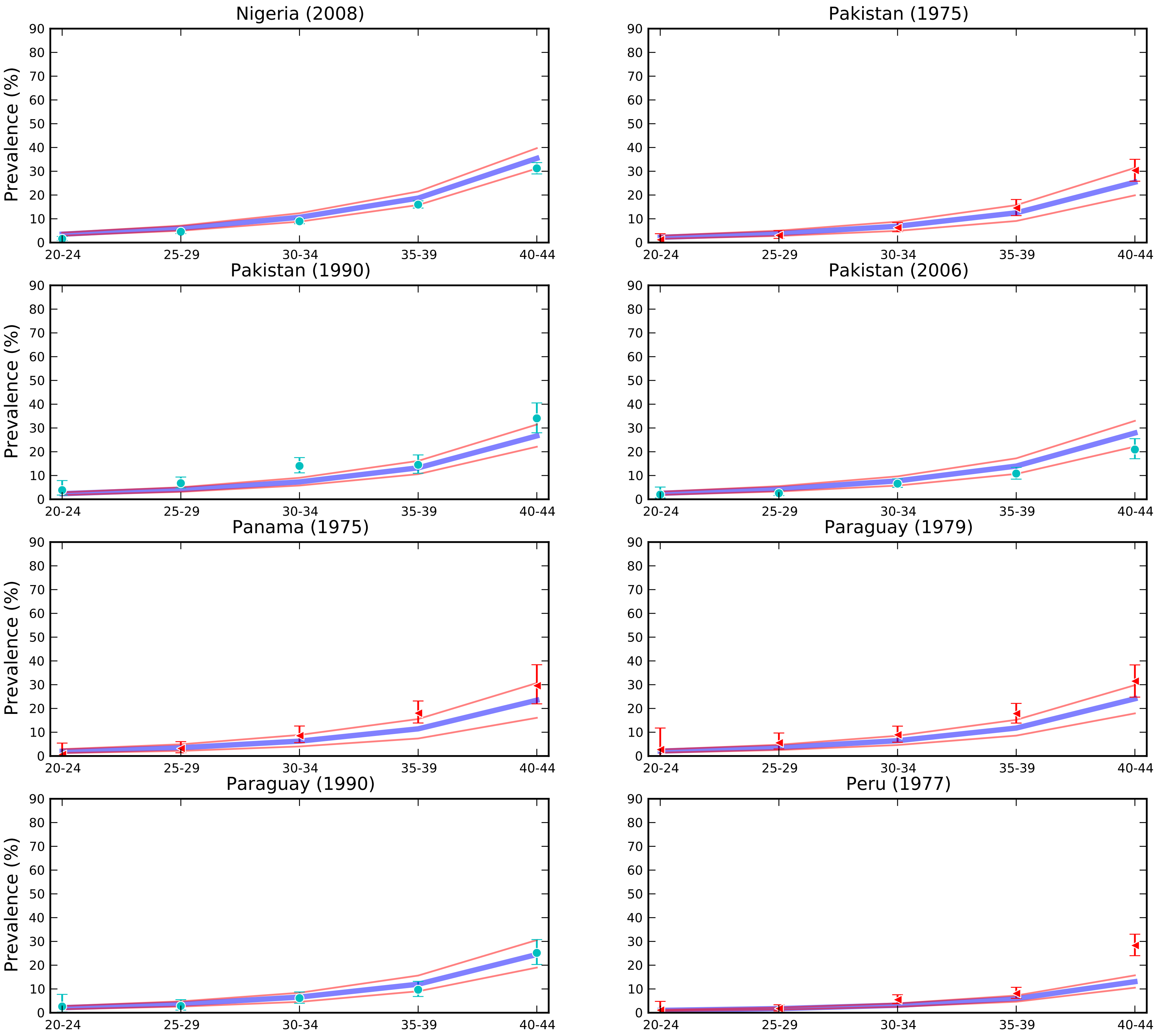

Nigeria (2008)
Pakistan (1975)
Pakistan (1990)
Pakistan (2006)
Panama (1975)
Paraguay (1979)
Paraguay (1990)
Peru (1977)
Prevalence (%)
0
10
20
30
40
50
60
70
80
90
20-24
25-29
30-34
35-39
40-44

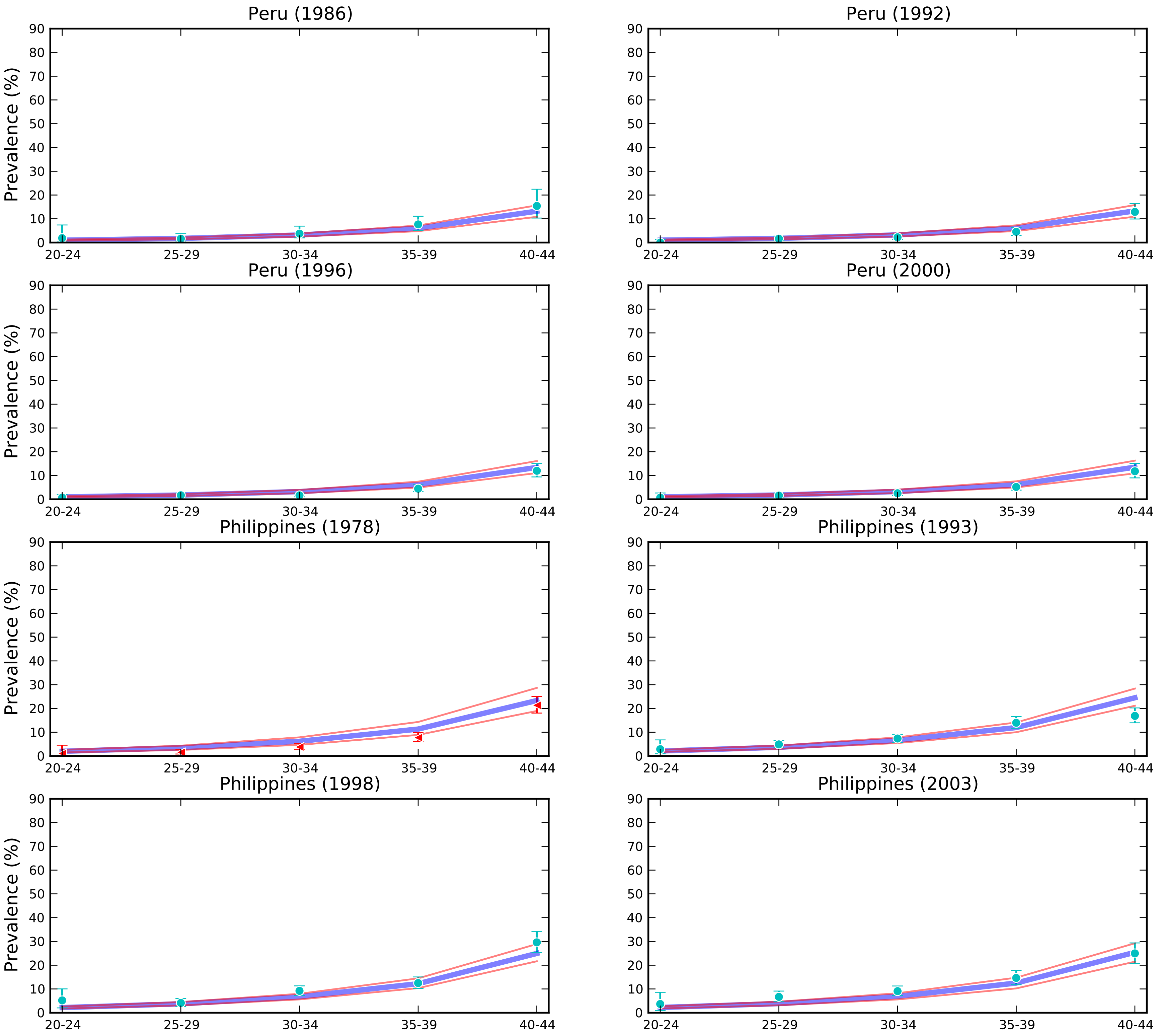
Peru (1986)
Peru (1992)
Peru (1996)
Peru (2000)
Philippines (1978)
Philippines (1993)
Philippines (1998)
Philippines (2003)
Prevalence (%)
0
10
20
30
40
50
60
70
80
90
20-24
25-29
30-34
35-39
40-44

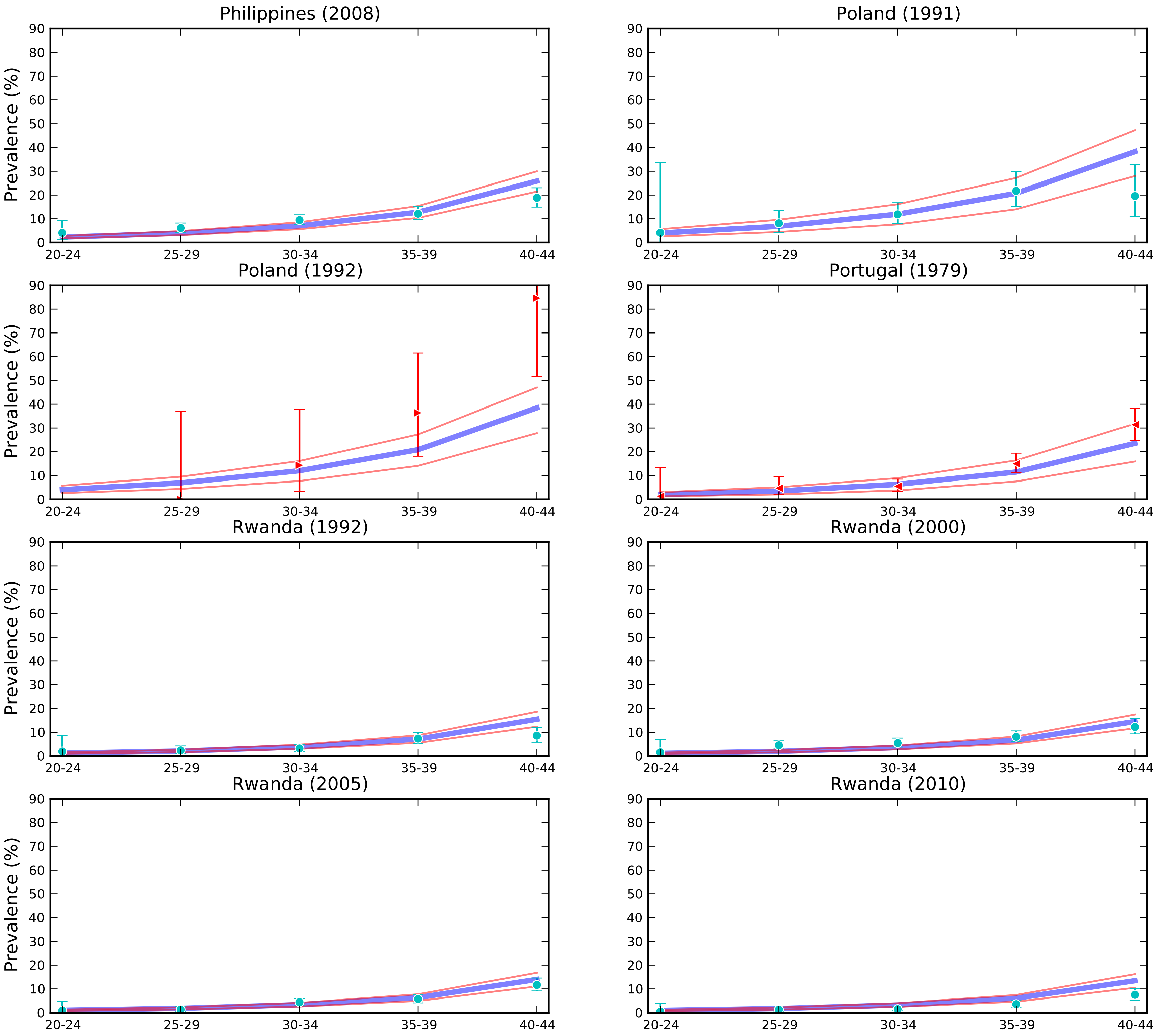
Philippines (2008)
Poland (1991)
Poland (1992)
Portugal (1979)
Rwanda (1992)
Rwanda (2000)
Rwanda (2005)
Rwanda (2010)
Prevalence (%)
0
10
20
30
40
50
60
70
80
90
20-24
25-29
30-34
35-39
40-44

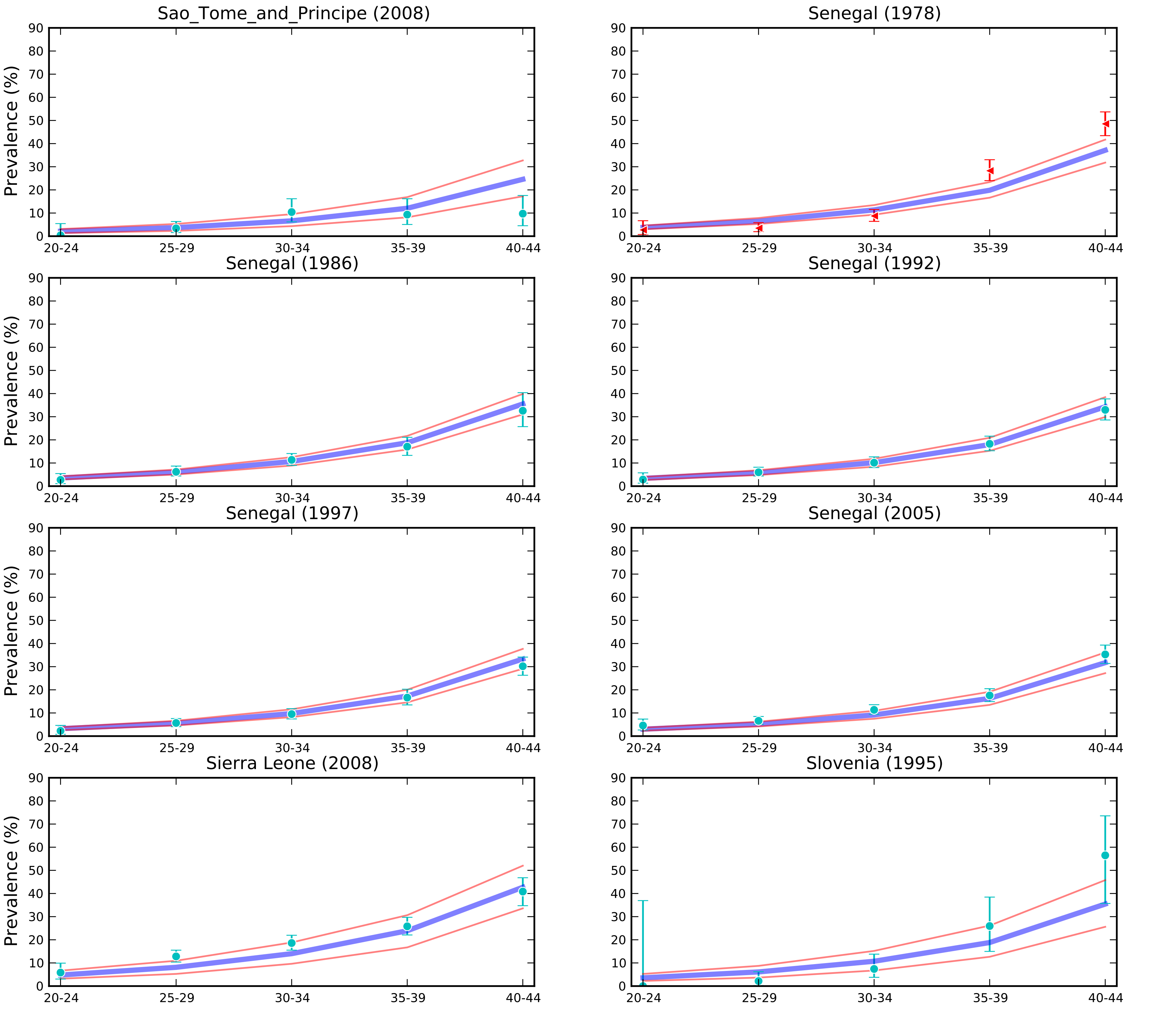

Sao_Tome_and_Principe (2008)
Senegal (1978)
Senegal (1986)
Senegal (1992)
Senegal (1997)
Senegal (2005)
Sierra Leone (2008)
Slovenia (1995)
Prevalence (%)
0
10
20
30
40
50
60
70
80
90
20-24
25-29
30-34
35-39
40-44

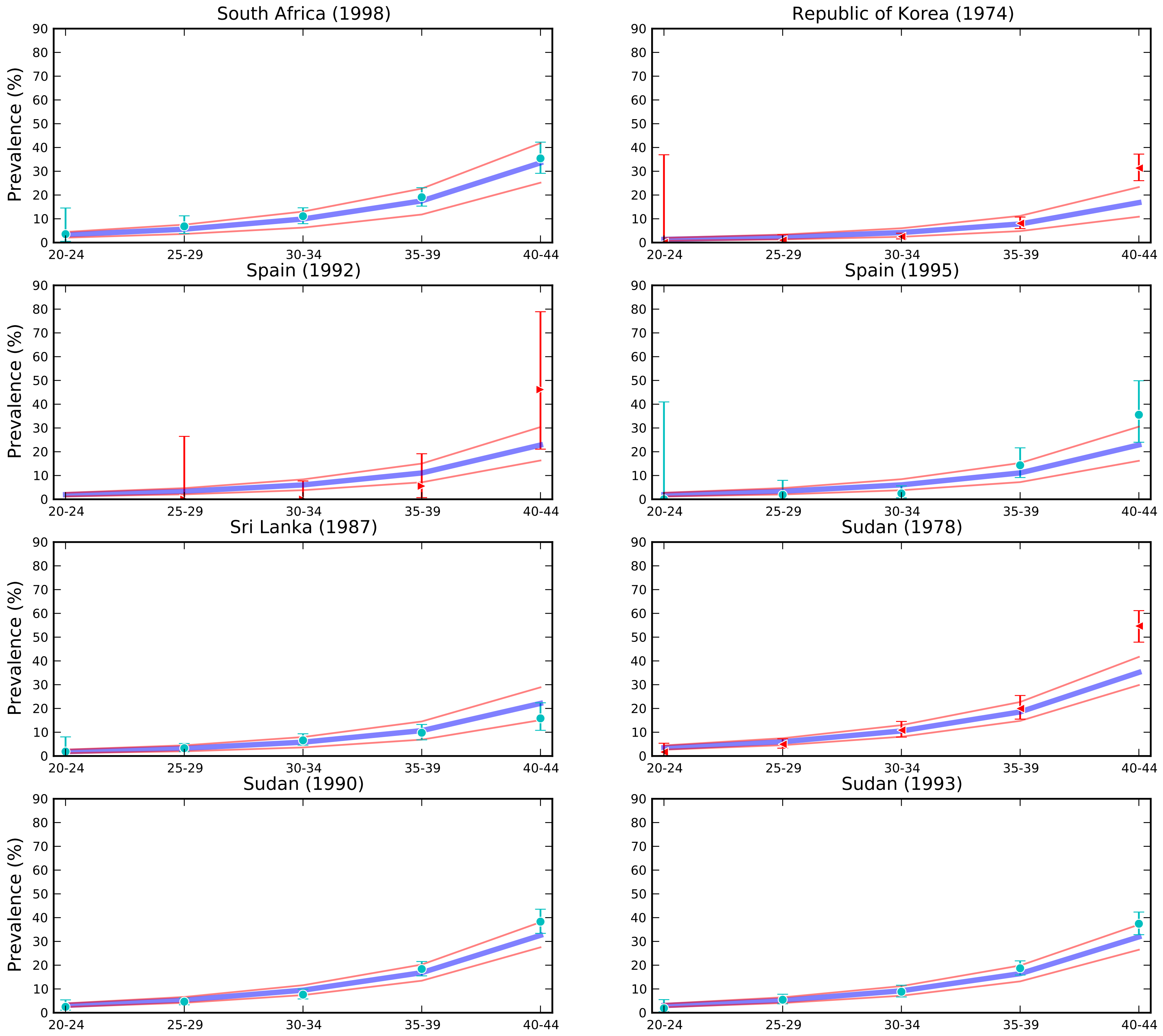

South Africa (1998)
Republic of Korea (1974)
Spain (1992)
Spain (1995)
Sri Lanka (1987)
Sudan (1978)
Sudan (1990)
Sudan (1993)
Prevalence (%)
0
10
20
30
40
50
60
70
80
90
20-24
25-29
30-34
35-39
40-44

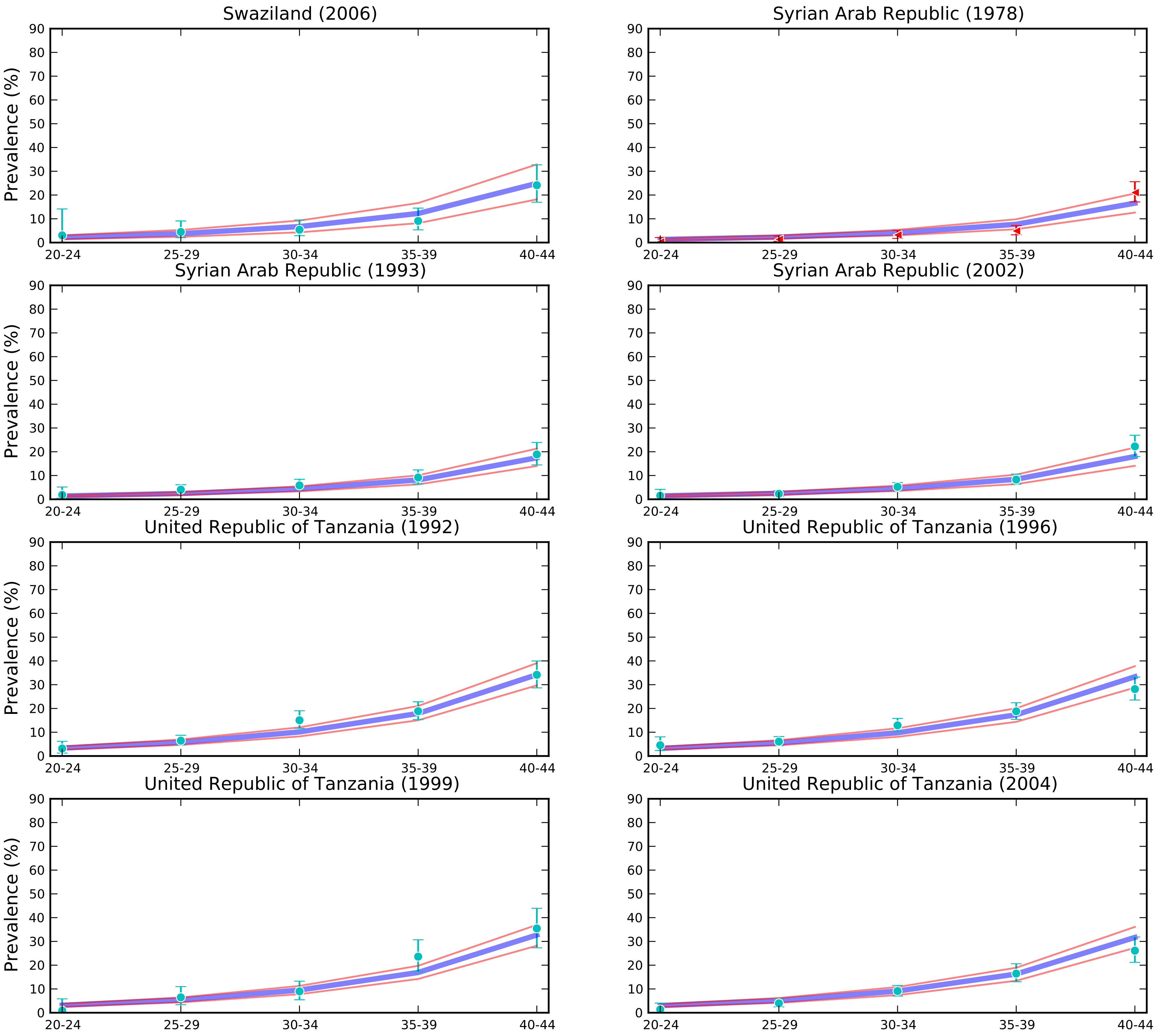
Swaziland (2006)
Syrian Arab Republic (1978)
Syrian Arab Republic (1993)
Syrian Arab Republic (2002)
United Republic of Tanzania (1992)
United Republic of Tanzania (1996)
United Republic of Tanzania (1999)
United Republic of Tanzania (2004)
Prevalence (%)
0 10 20 30 40 50 60 70 80 90
20-24 25-29 30-34 35-39 40-44

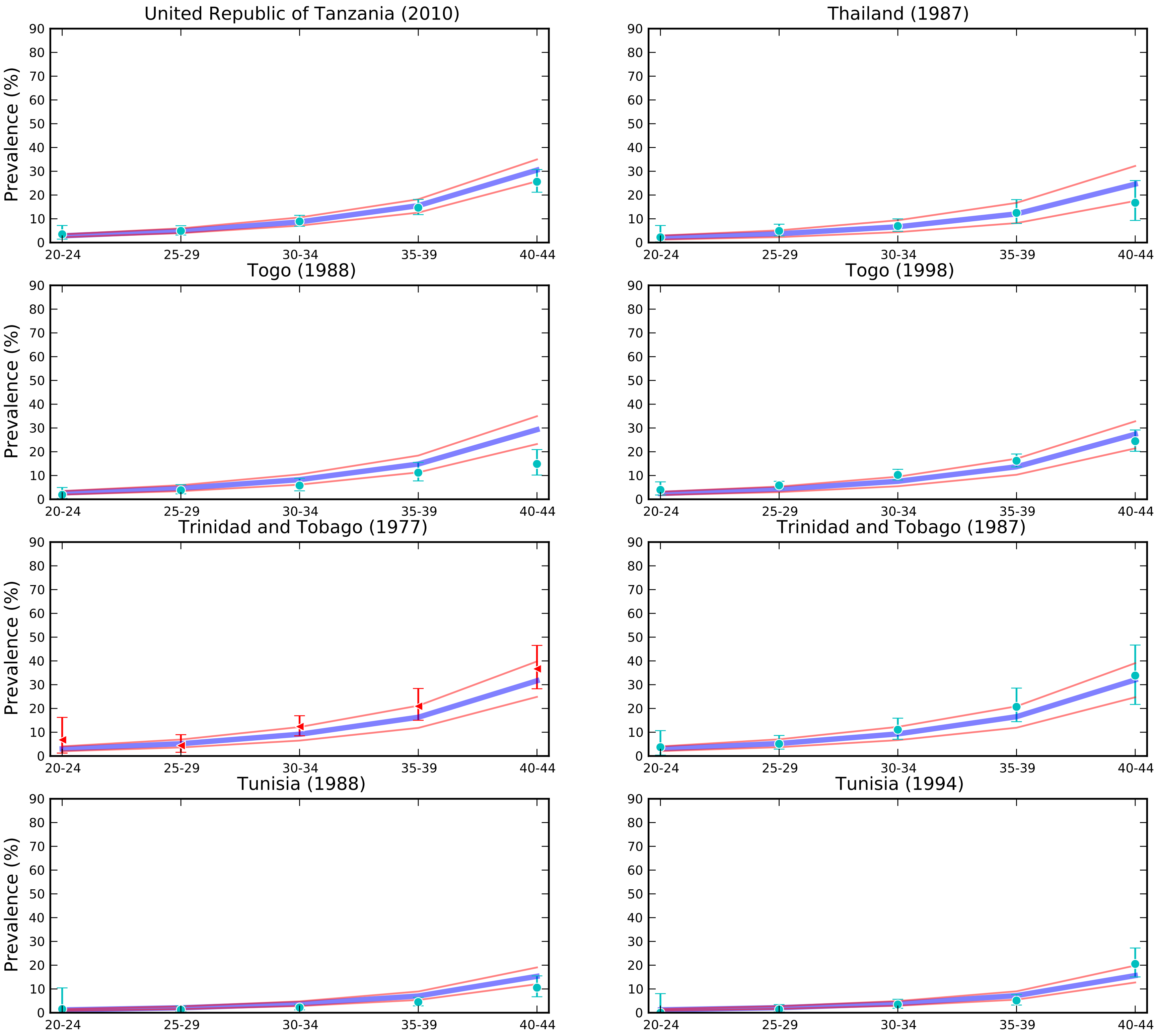
United Republic of Tanzania (2010)
Thailand (1987)
Togo (1988)
Togo (1998)
Trinidad and Tobago (1977)
Trinidad and Tobago (1987)
Tunisia (1988)
Tunisia (1994)
Prevalence (%)
0
10
20
30
40
50
60
70
80
90
20-24
25-29
30-34
35-39
40-44

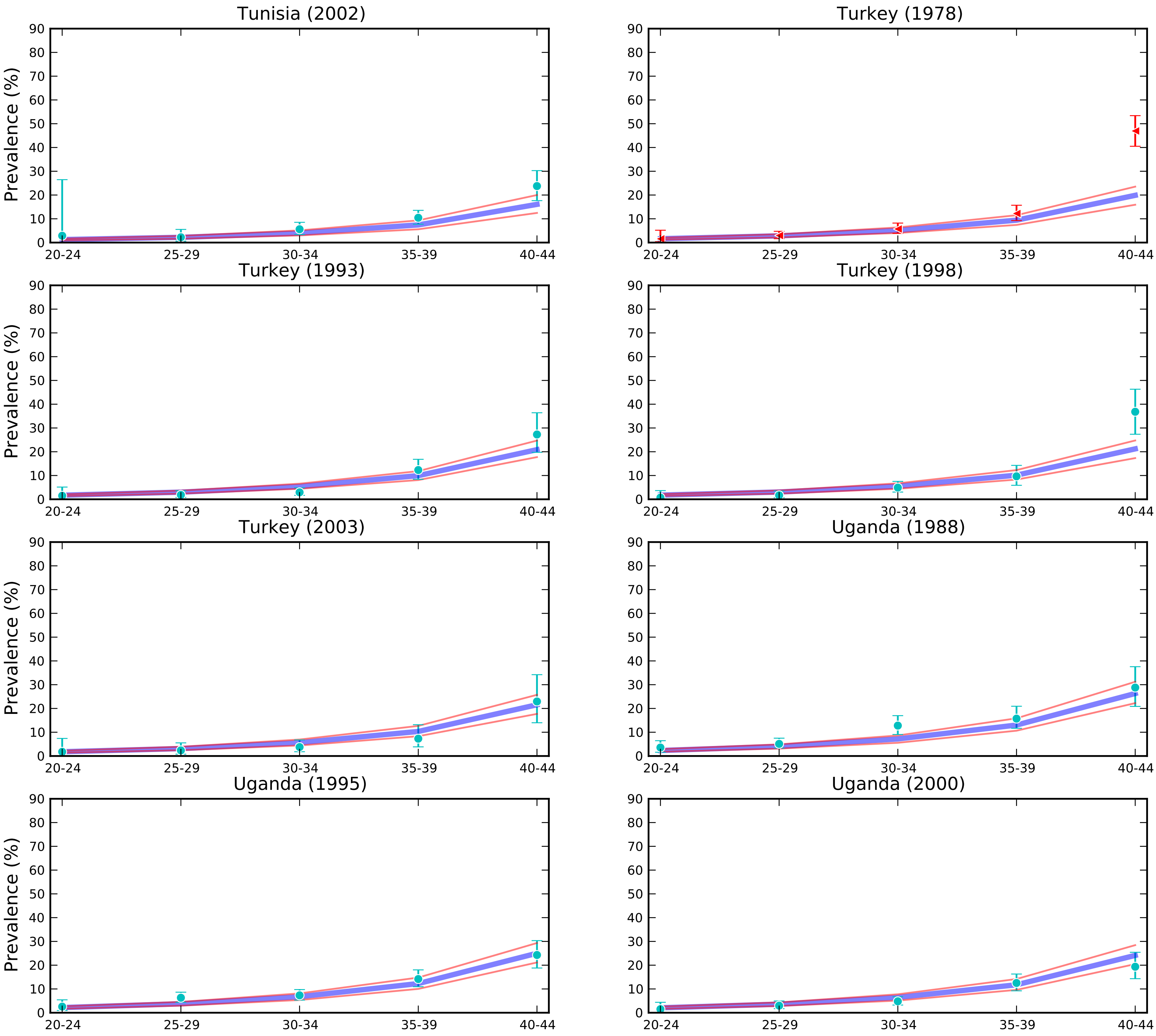
Tunisia (2002)
Turkey (1978)
Turkey (1993)
Turkey (1998)
Turkey (2003)
Uganda (1988)
Uganda (1995)
Uganda (2000)
Prevalence (%)
0
10
20
30
40
50
60
70
80
90
20-24
25-29
30-34
35-39
40-44

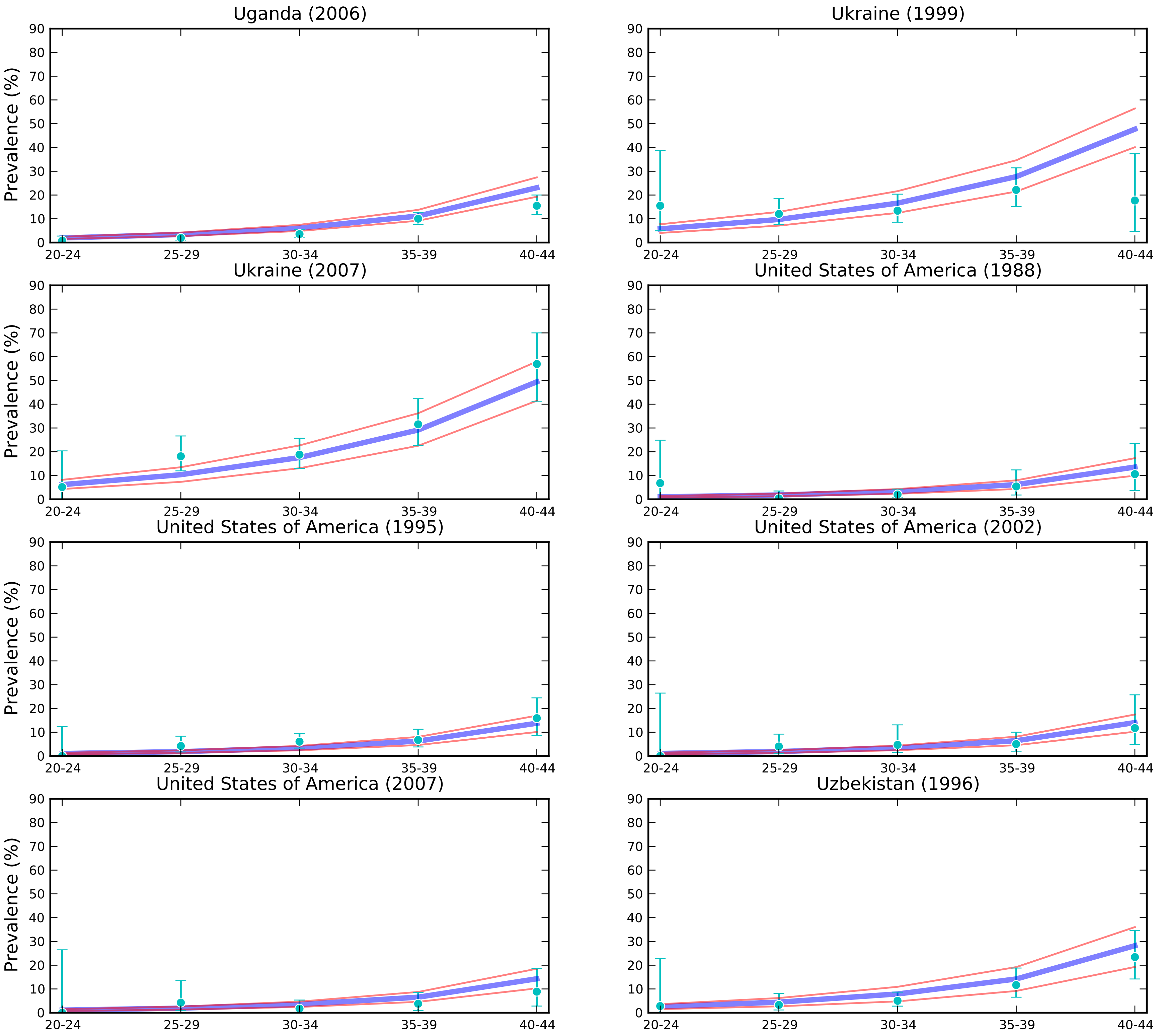
Uganda (2006)
Ukraine (1999)
Ukraine (2007)
United States of America (1988)
United States of America (1995)
United States of America (2002)
United States of America (2007)
Uzbekistan (1996)
Prevalence (%)
0
10
20
30
40
50
60
70
80
90
20-24
25-29
30-34
35-39
40-44

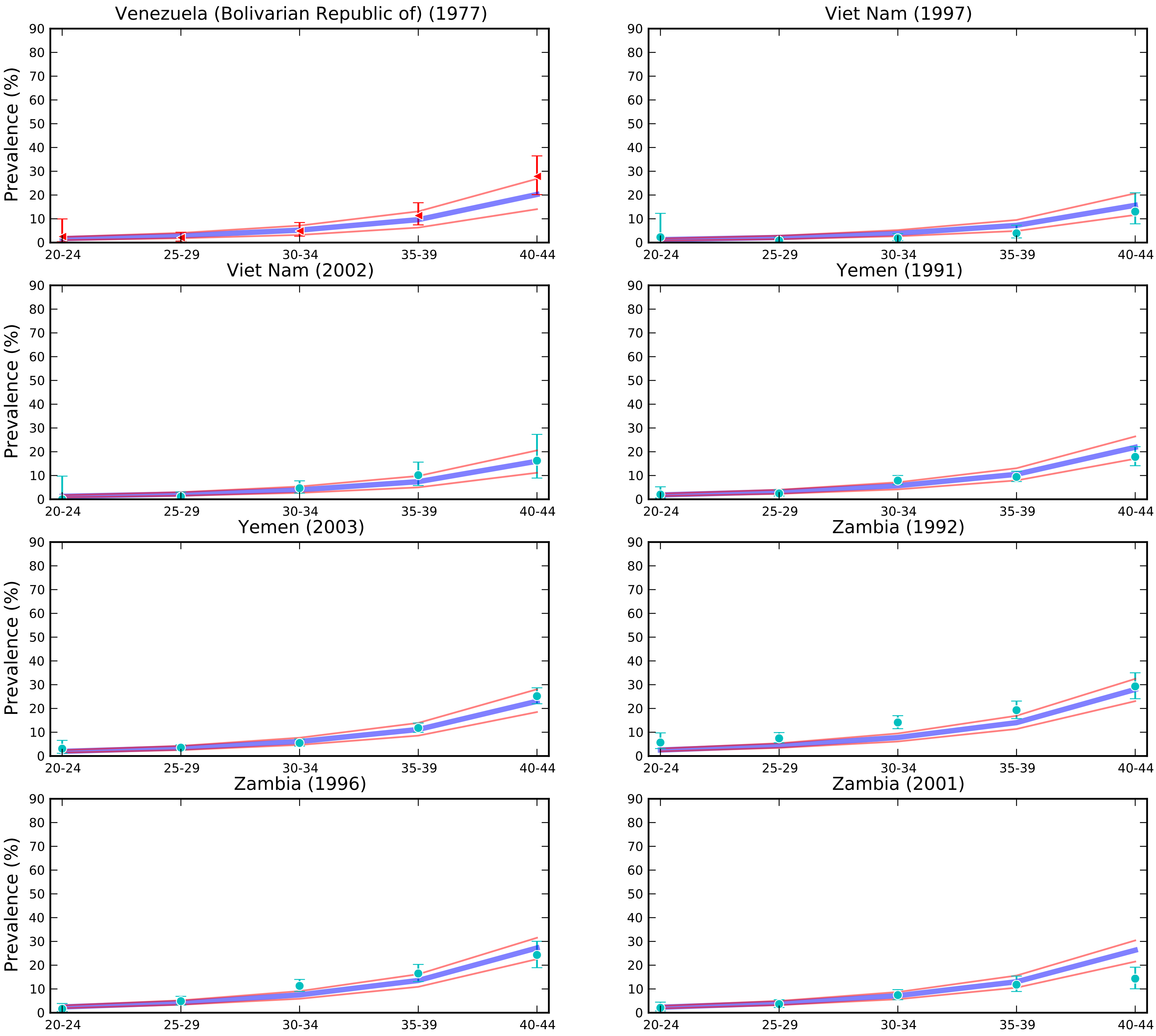

Venezuela (Bolivarian Republic of) (1977)
Viet Nam (1997)
Viet Nam (2002)
Yemen (1991)
Yemen (2003)
Zambia (1992)
Zambia (1996)
Zambia (2001)
Prevalence (%)
0
10
20
30
40
50
60
70
80
90
20-24
25-29
30-34
35-39
40-44

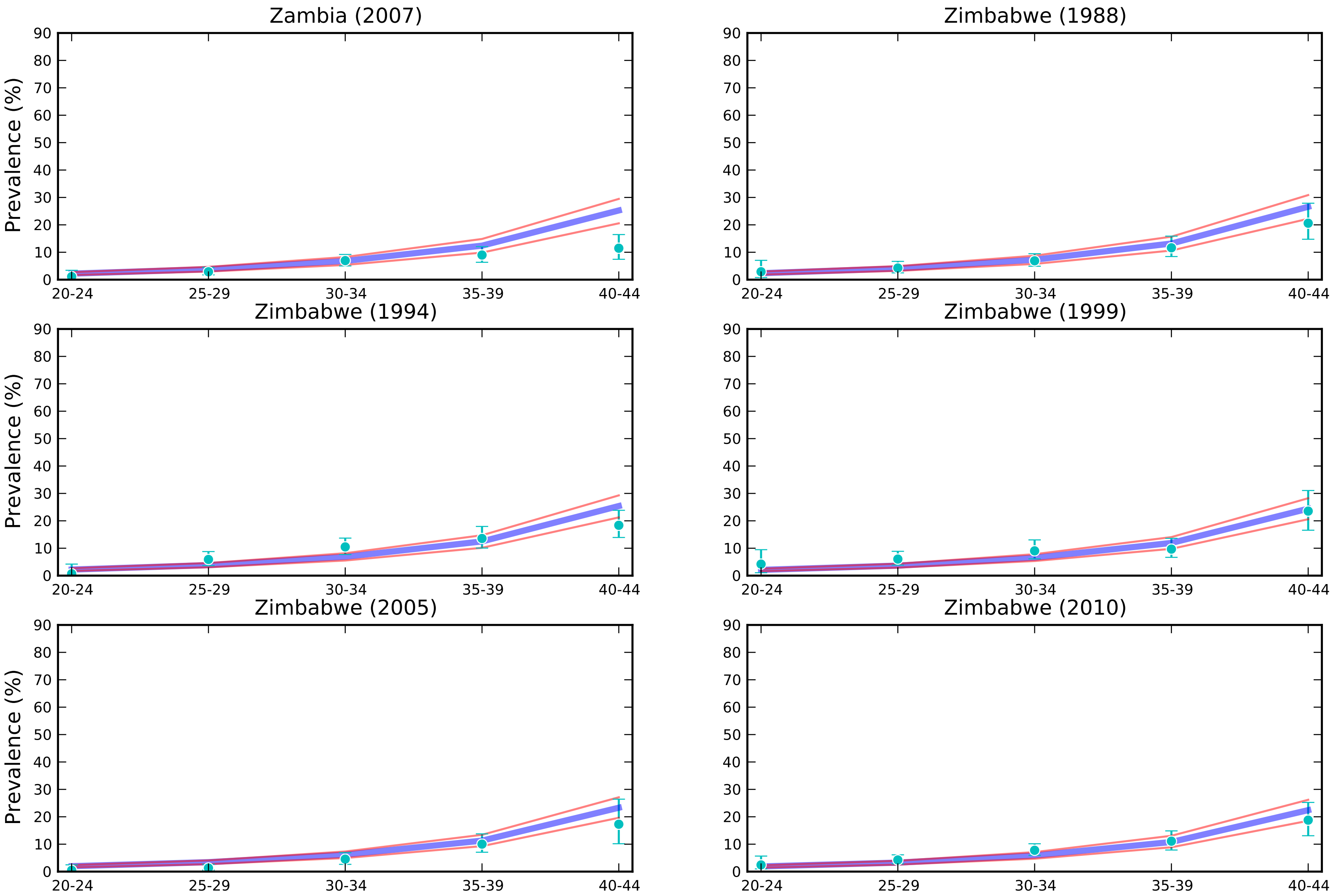
Zambia (2007)
Zimbabwe (1988)
Zimbabwe (1994)
Zimbabwe (1999)
Zimbabwe (2005)
Zimbabwe (2010)
Prevalence (%)
0 10 20 30 40 50 60 70 80 90
20-24 25-29 30-34 35-39 40-44

Figure F: Trends in prevalence of primary infertility by year, shown for each country with data on following pages. Infertility is indexed on the female partner; age-standardized prevalence among women aged 20-44 years is shown here. Legend for plots is given below.

- Nationally representative (error bars represent standard errors)
- Nationally representative, data do not cover the entire 20-44 year age range
- Subnational
- Subnational, data do not cover the entire 20-44 year age range
- World Fertility Survey
- World Fertility Survey, data do not cover the entire 20-44 year age range
- Posterior means (model predictions)
- 95% credible interval

Primary Prevalence

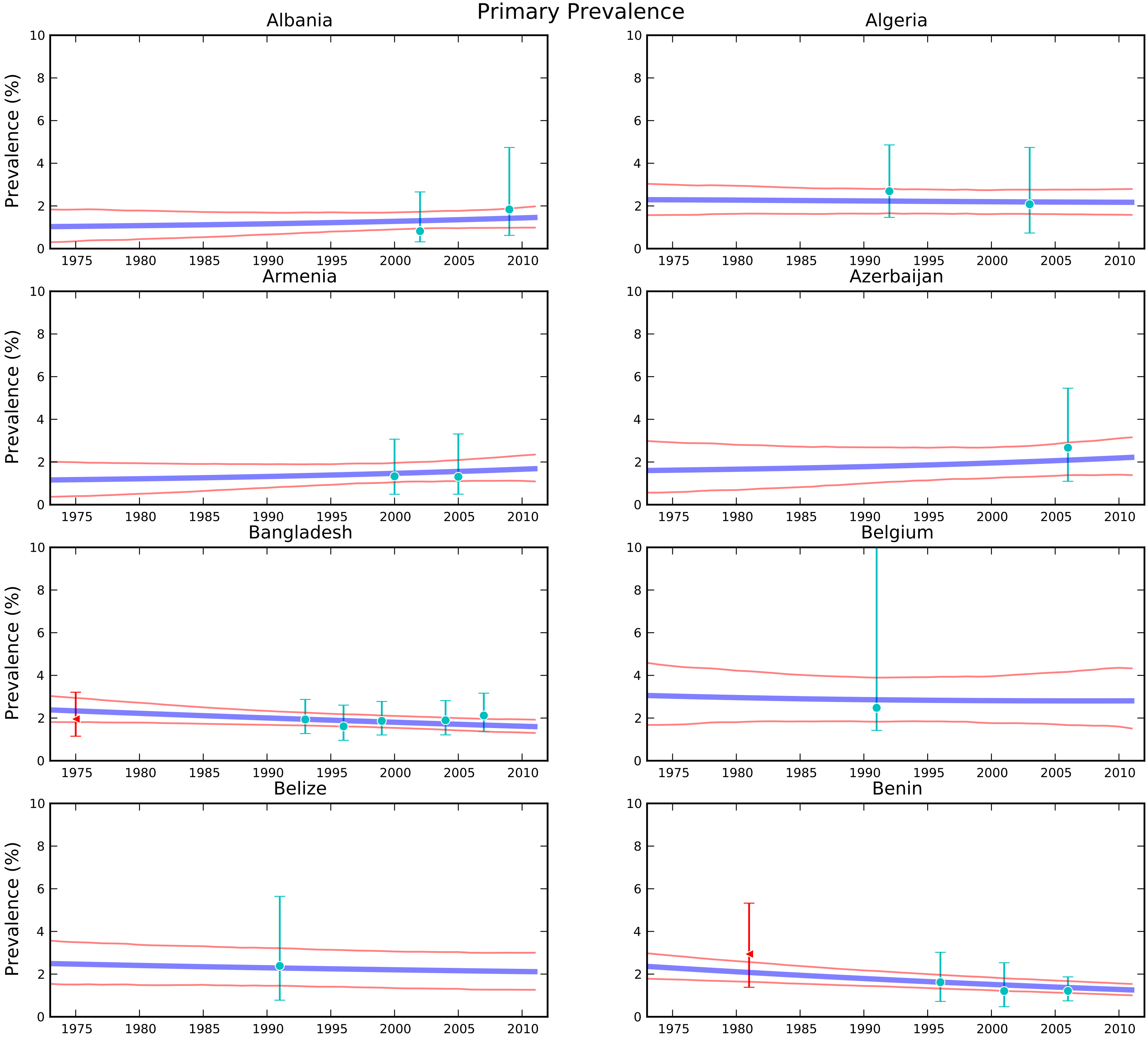

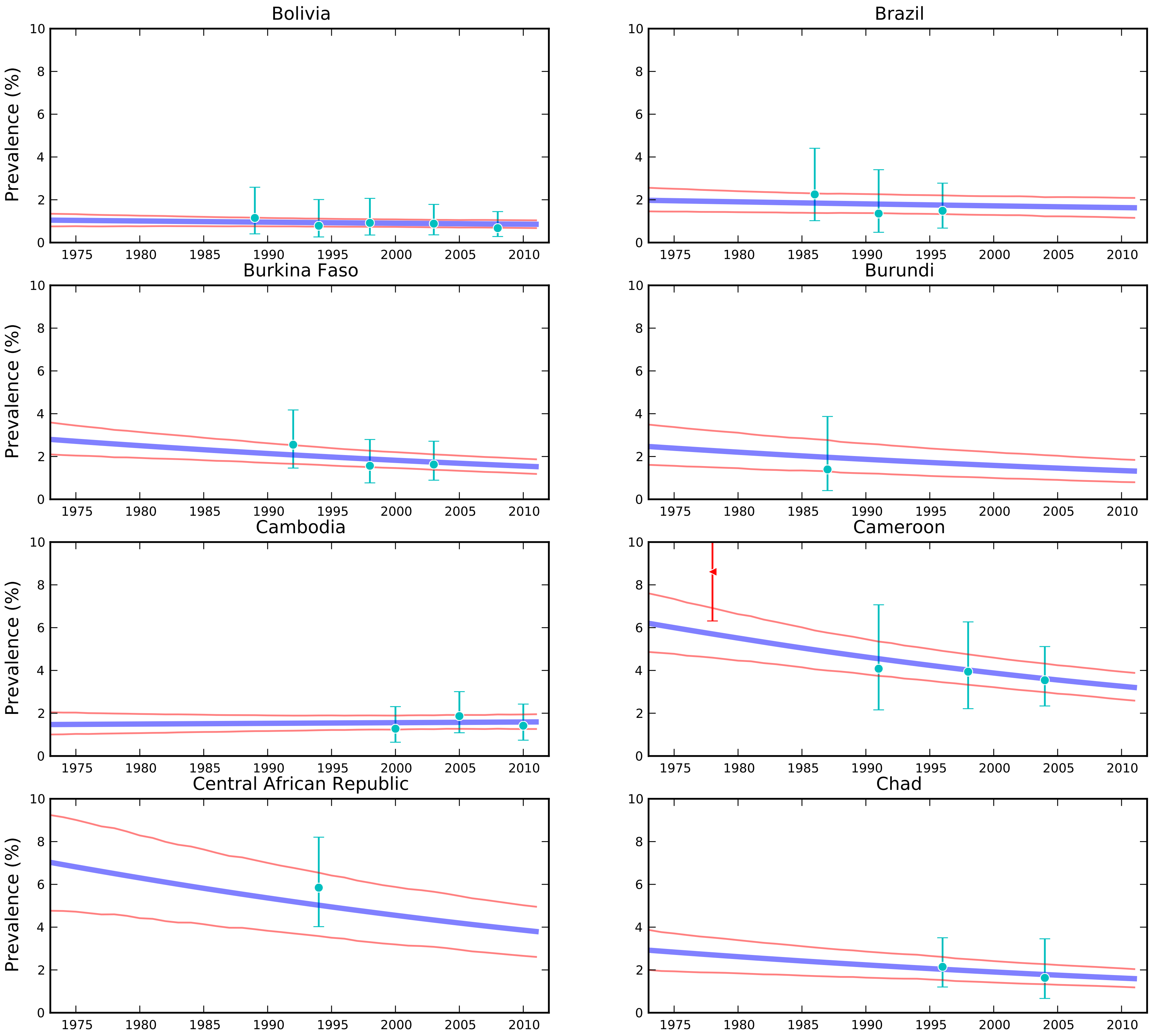
Bolivia
Brazil
Burkina Faso
Burundi
Cambodia
Cameroon
Central African Republic
Chad
Prevalence (%)
0
2
4
6
8
10
1975
1980
1985
1990
1995
2000
2005
2010

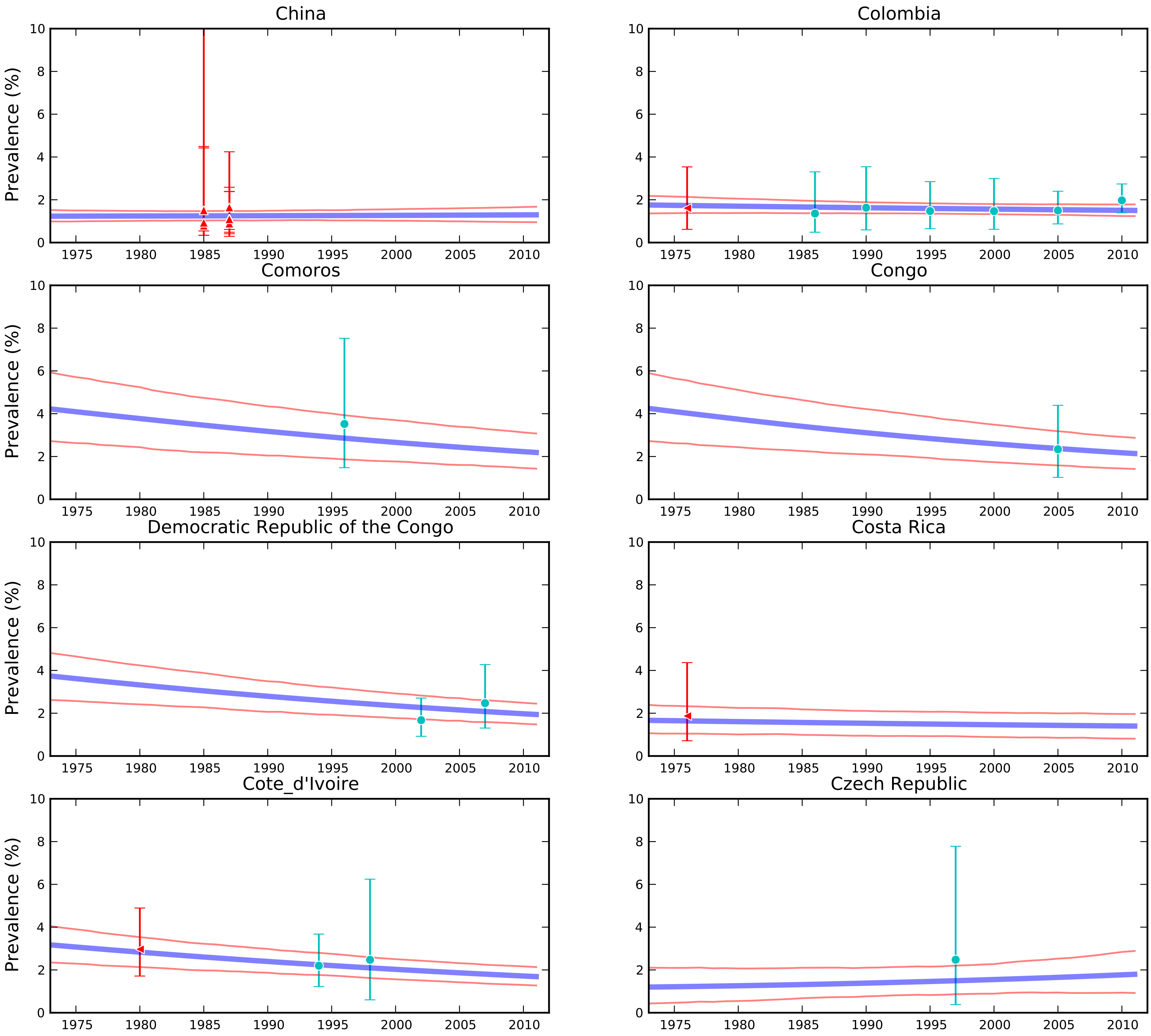
China
Colombia
Comoros
Congo
Democratic Republic of the Congo
Costa Rica
Cote_d'Ivoire
Czech Republic
Prevalence (%)
0
2
4
6
8
10
1975
1980
1985
1990
1995
2000
2005
2010

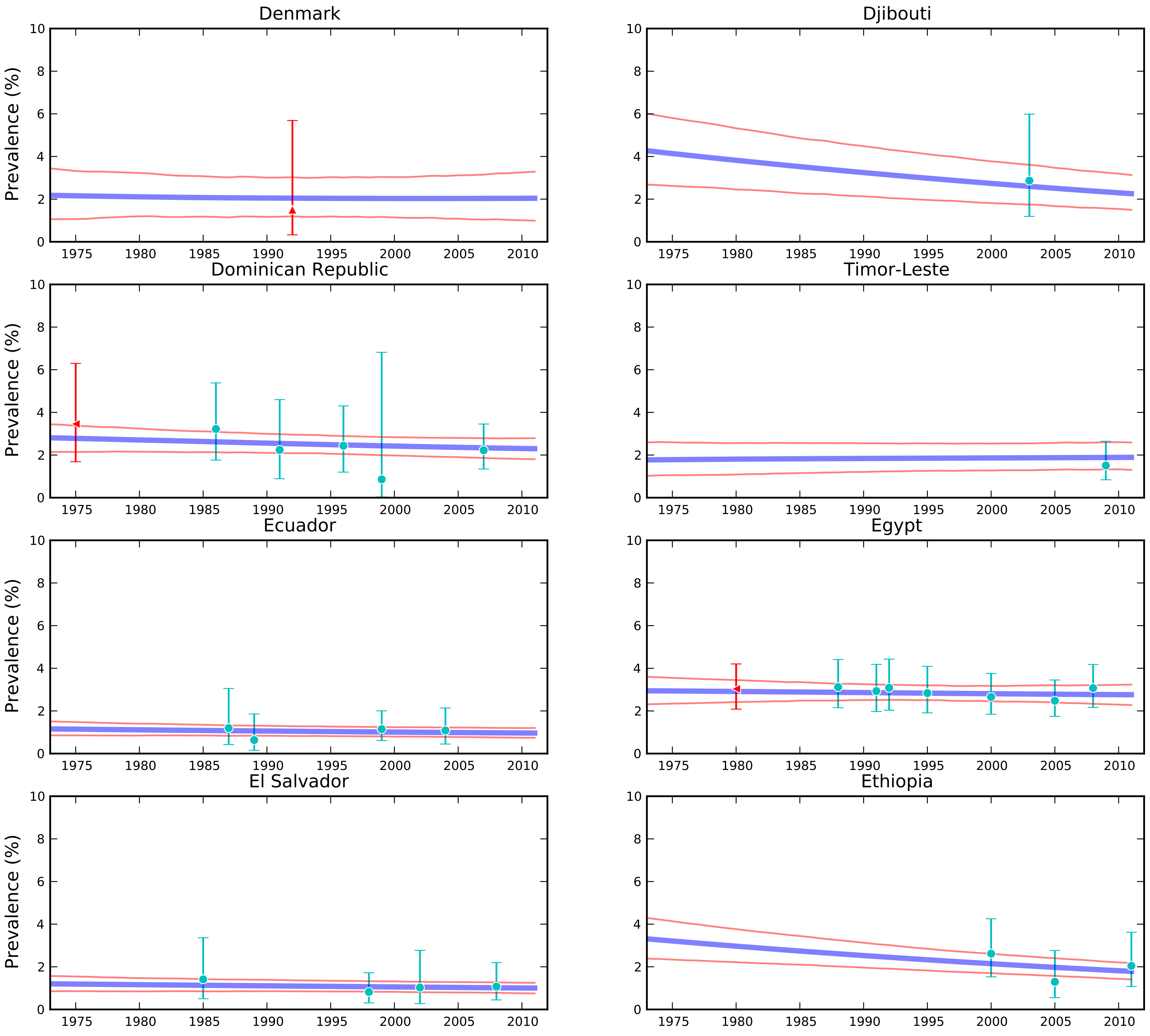
Denmark
Djibouti
Dominican Republic
Timor-Leste
Ecuador
Egypt
El Salvador
Ethiopia
Prevalence (%)
0
2
4
6
8
10
1975
1980
1985
1990
1995
2000
2005
2010

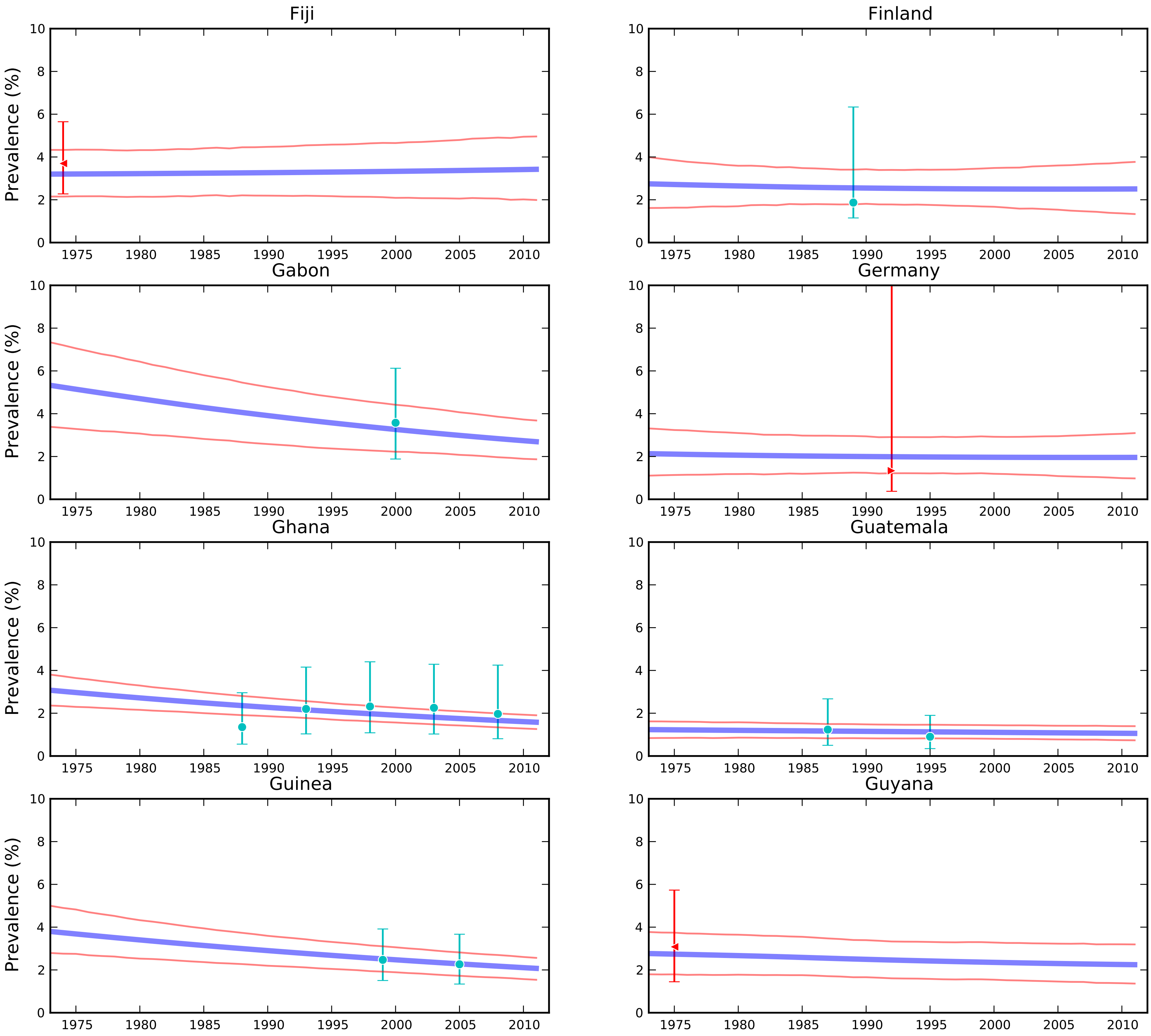
Fiji
Finland
Gabon
Germany
Ghana
Guatemala
Guinea
Guyana
Prevalence (%)
0
2
4
6
8
10
1975
1980
1985
1990
1995
2000
2005
2010

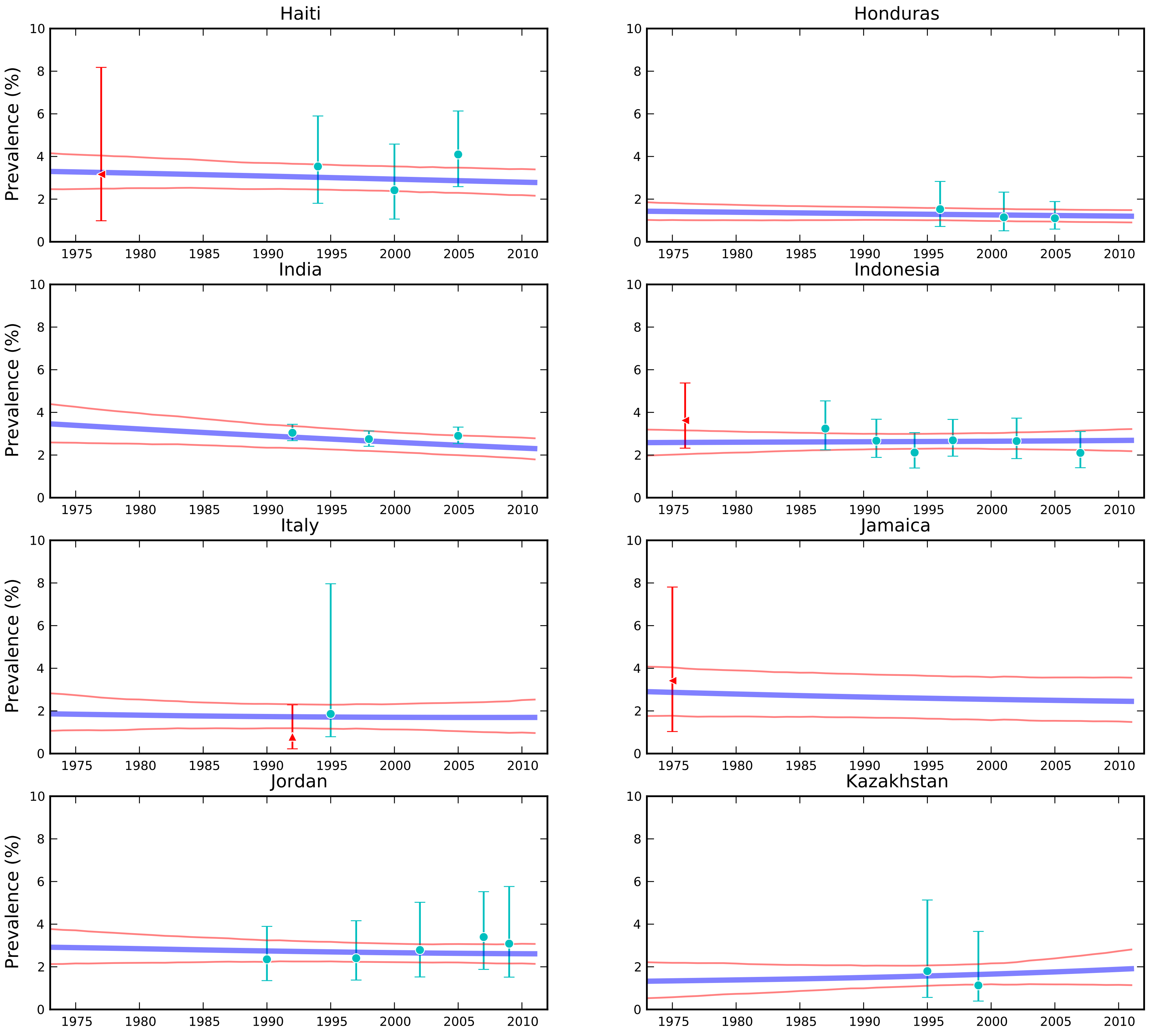
Haiti
Honduras
India
Indonesia
Italy
Jamaica
Jordan
Kazakhstan
Prevalence (%)
0
2
4
6
8
10
1975
1980
1985
1990
1995
2000
2005
2010

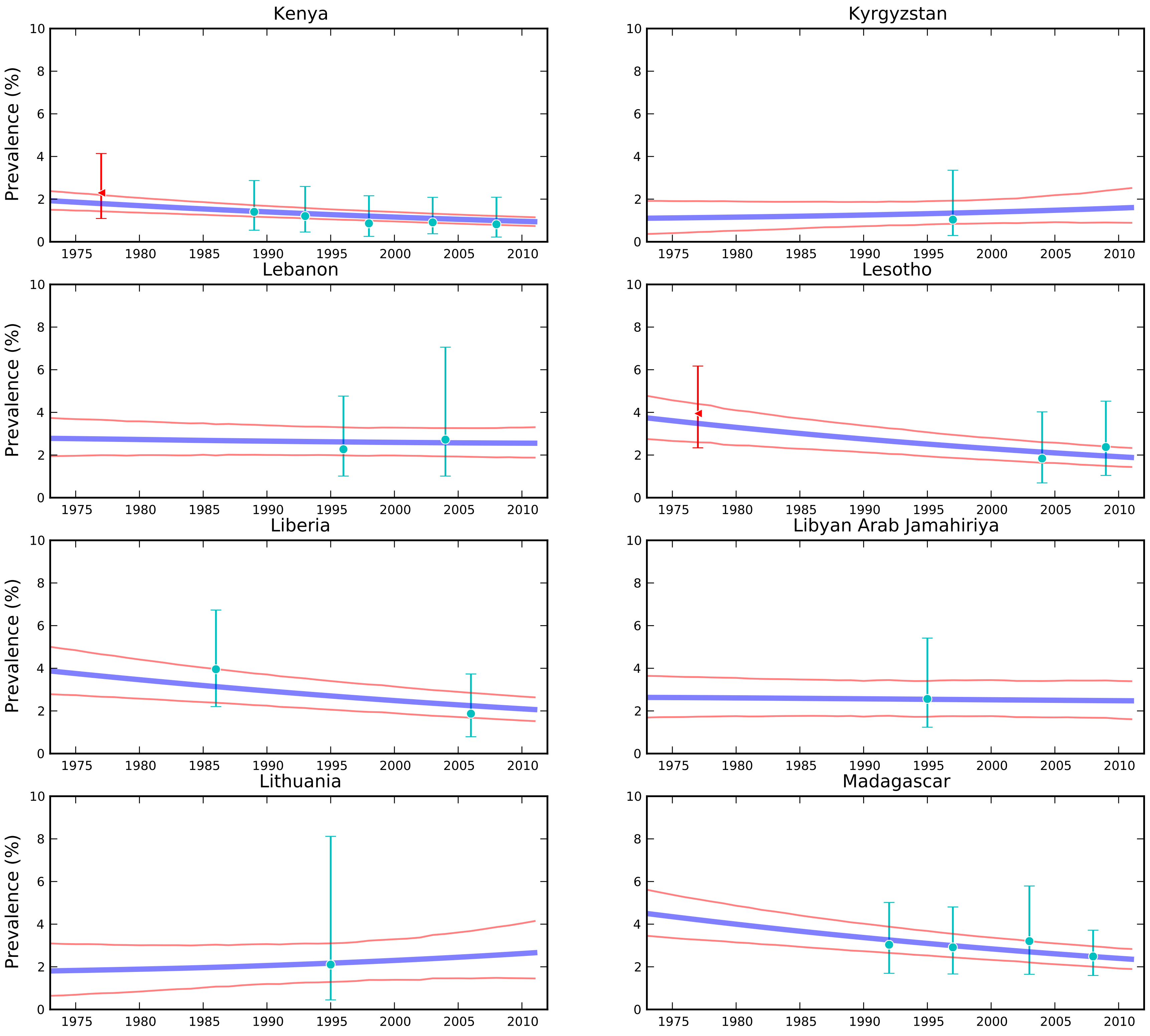

Kenya
Kyrgyzstan
Lebanon
Lesotho
Liberia
Libyan Arab Jamahiriya
Lithuania
Madagascar
Prevalence (%)
0
2
4
6
8
10
1975
1980
1985
1990
1995
2000
2005
2010

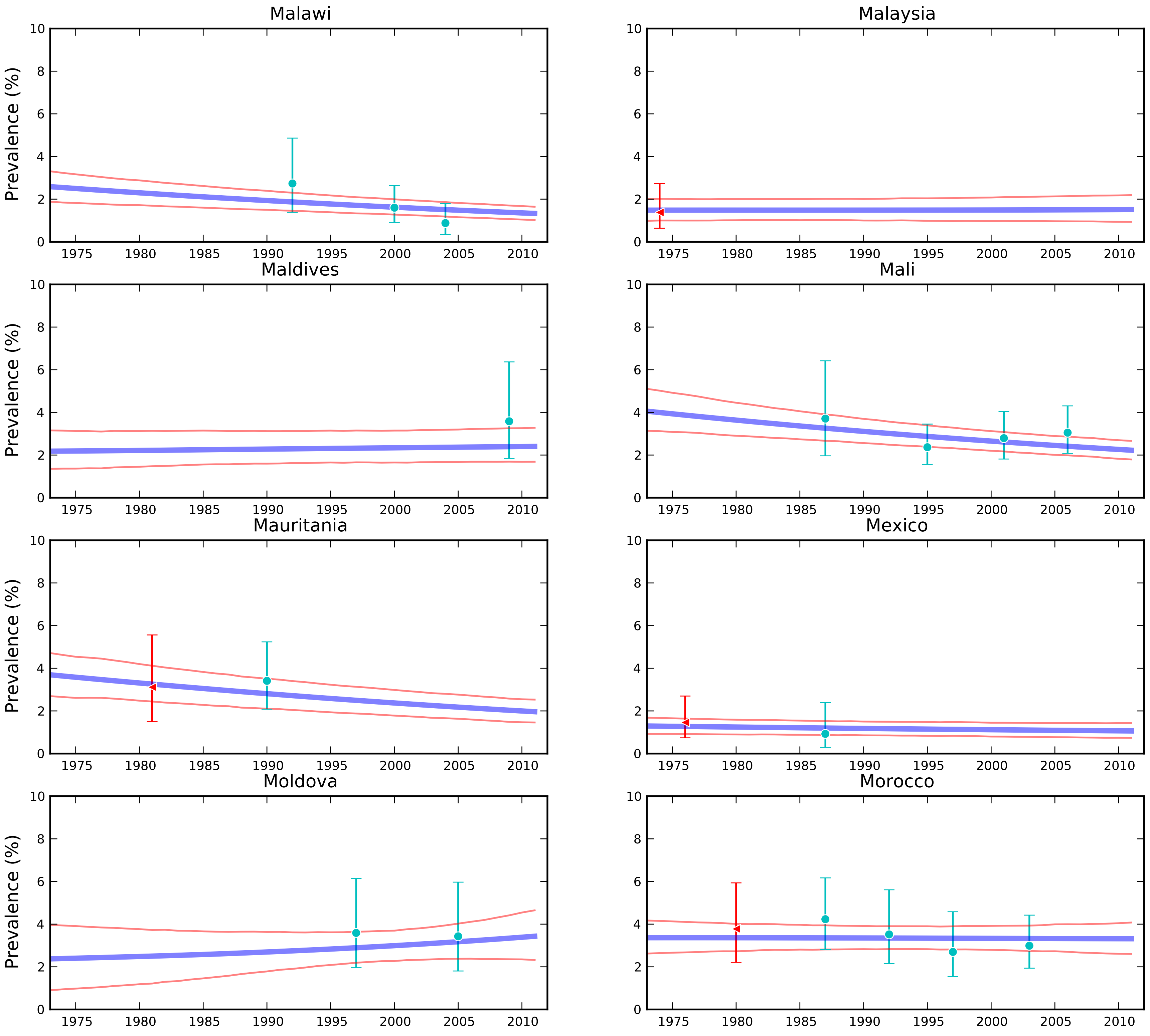
Malawi
Malaysia
Maldives
Mali
Mauritania
Mexico
Moldova
Morocco
Prevalence (%)
0
2
4
6
8
10
1975
1980
1985
1990
1995
2000
2005
2010

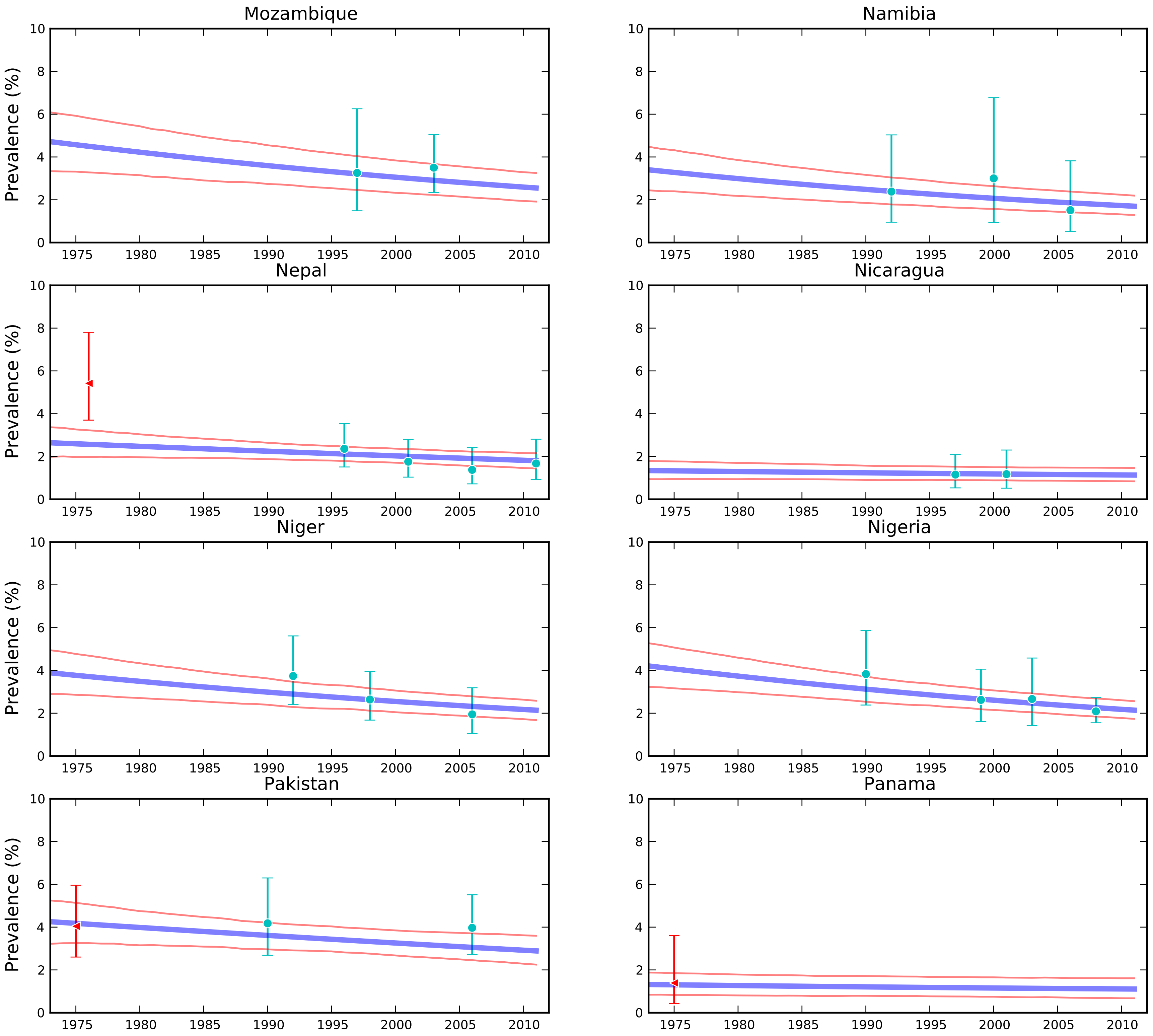
Mozambique
Namibia
Nepal
Nicaragua
Niger
Nigeria
Pakistan
Panama
Prevalence (%)
0
2
4
6
8
10
1975
1980
1985
1990
1995
2000
2005
2010

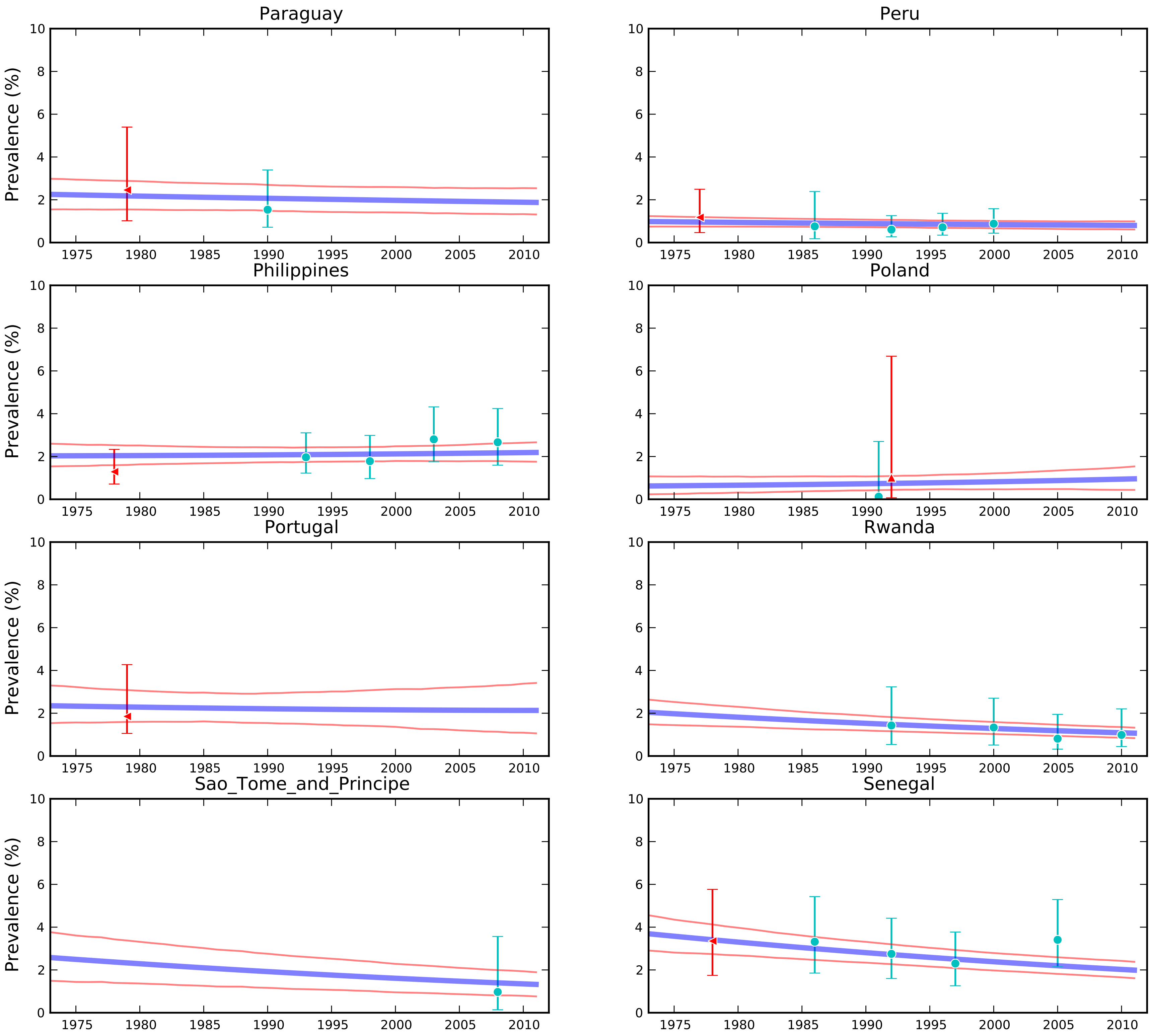

Paraguay
Peru
Philippines
Poland
Portugal
Rwanda
Sao_Tome_and_Principe
Senegal
Prevalence (%)
0
2
4
6
8
10
1975
1980
1985
1990
1995
2000
2005
2010

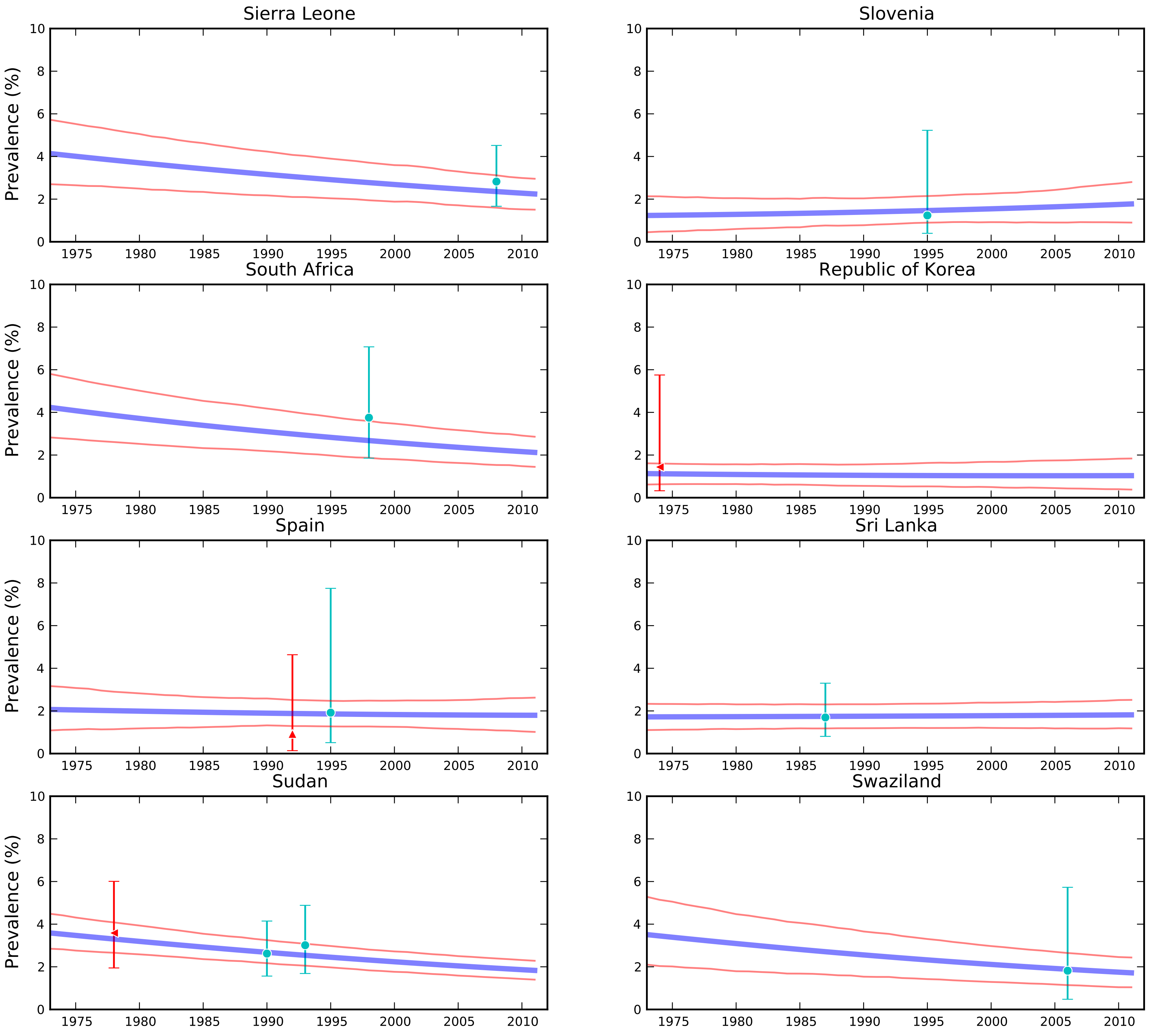
Sierra Leone
Slovenia
South Africa
Republic of Korea
Spain
Sri Lanka
Sudan
Swaziland
Prevalence (%)
0 2 4 6 8 10
1975 1980 1985 1990 1995 2000 2005 2010

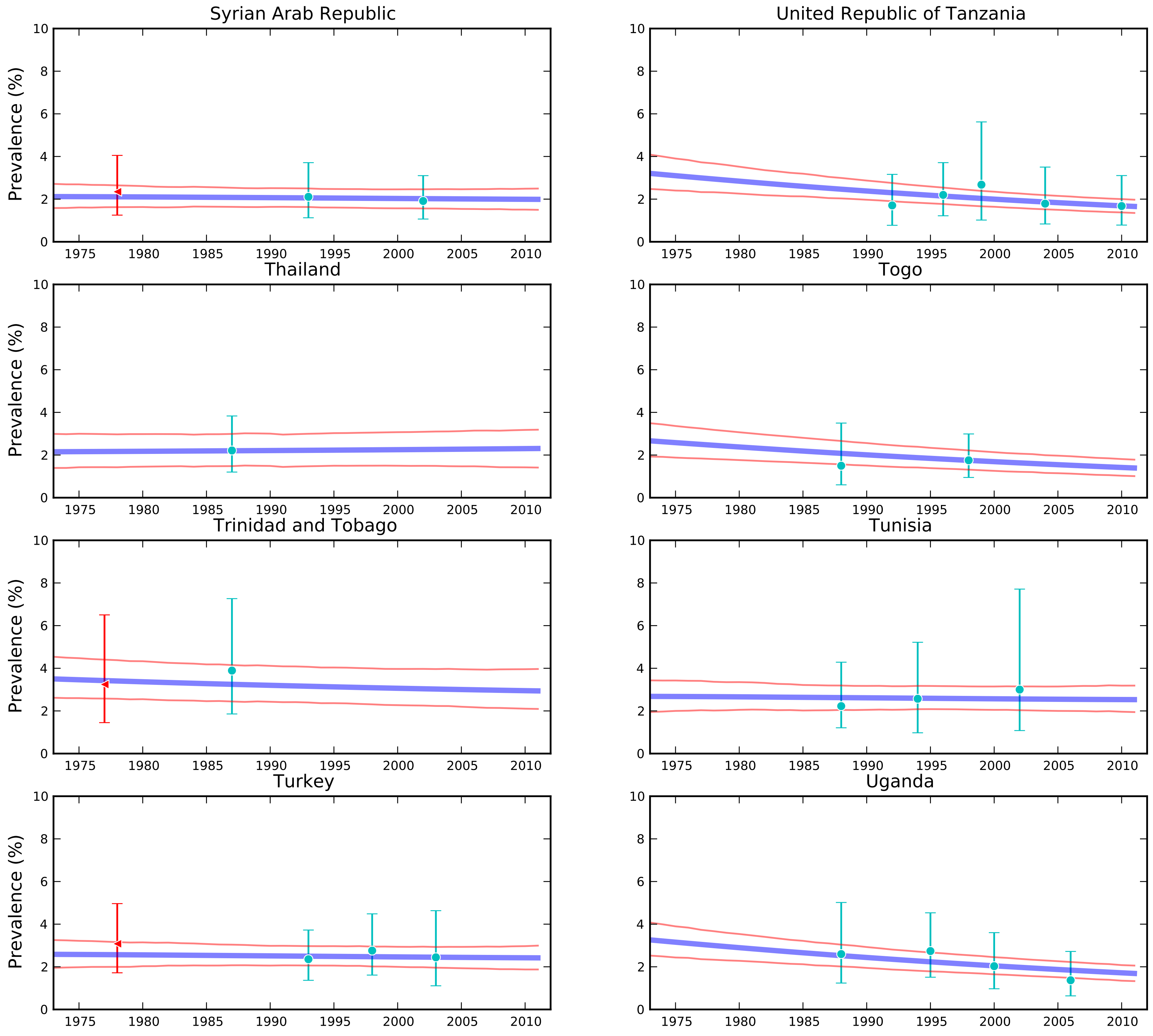
Syrian Arab Republic
United Republic of Tanzania
Thailand
Togo
Trinidad and Tobago
Tunisia
Turkey
Uganda
Prevalence (%)
0
2
4
6
8
10
1975
1980
1985
1990
1995
2000
2005
2010

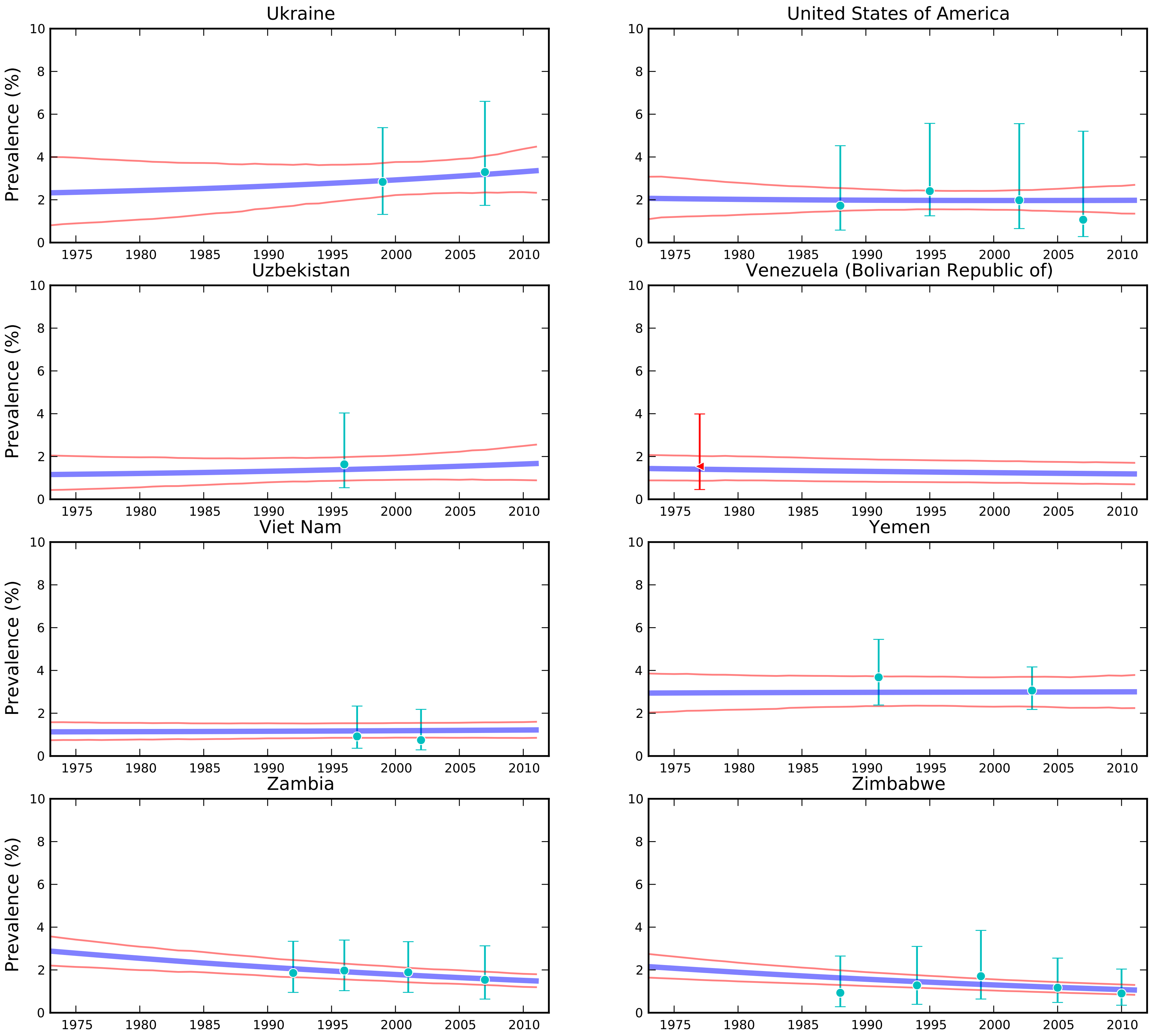
Ukraine
United States of America
Uzbekistan
Venezuela (Bolivarian Republic of)
Viet Nam
Yemen
Zambia
Zimbabwe
Prevalence (%)
0
2
4
6
8
10
1975
1980
1985
1990
1995
2000
2005
2010

Figure G: Trends in prevalence of secondary infertility by year, shown for each country with data on following pages. Infertility is indexed on the female partner; age-standardized prevalence among women aged 20-44 years is shown here. Legend for plots is given below.

- Nationally representative (error bars represent standard errors)
- Nationally representative, data do not cover the entire 20-44 year age range
- Subnational
- Subnational, data do not cover the entire 20-44 year age range
- World Fertility Survey
- World Fertility Survey, data do not cover the entire 20-44 year age range
- Posterior means (model predictions)
- 95% credible interval

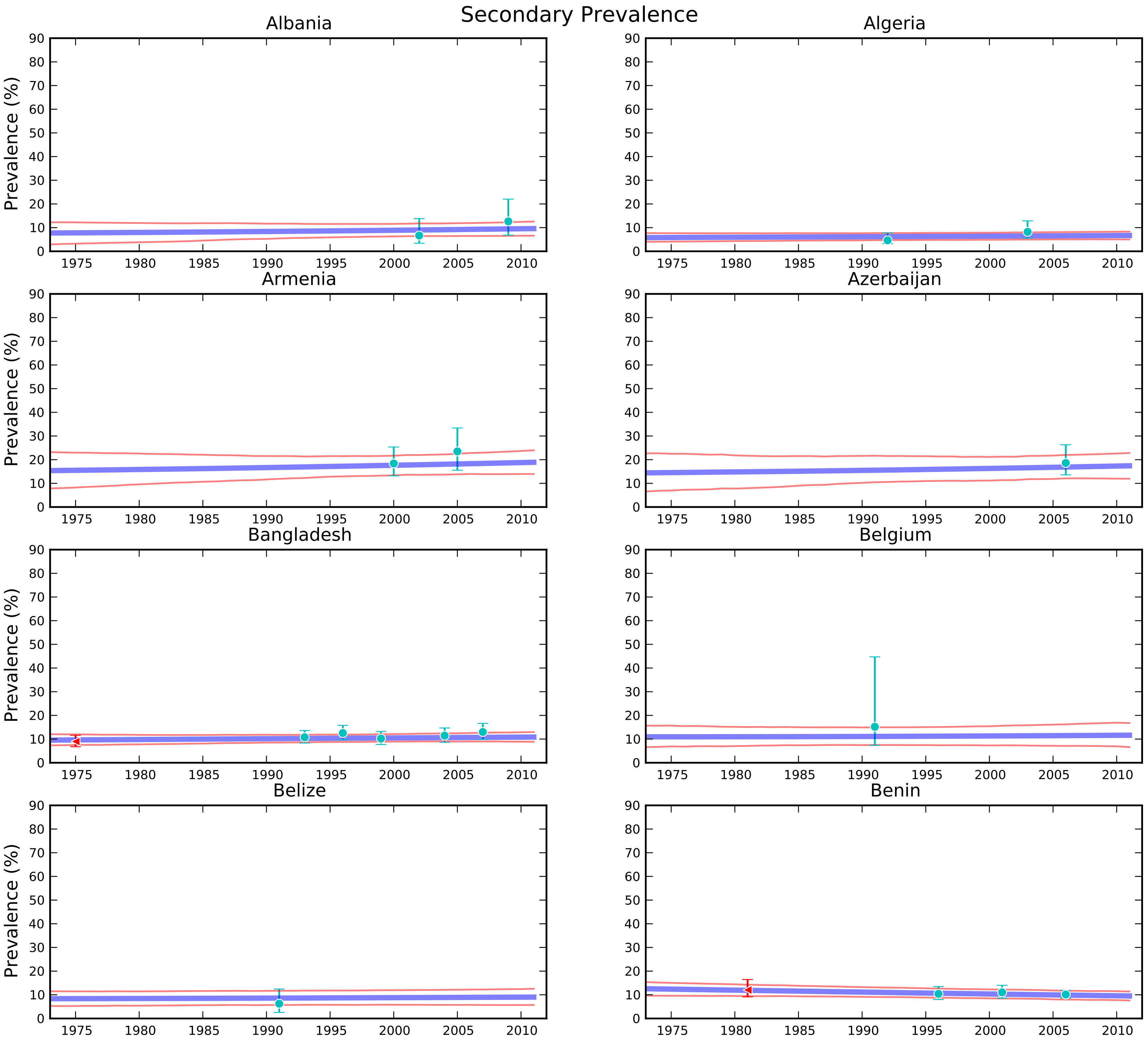
Secondary Prevalence
Albania
Algeria
Armenia
Azerbaijan
Bangladesh
Belgium
Belize
Benin
Prevalence (%)
0
10
20
30
40
50
60
70
80
90
1975
1980
1985
1990
1995
2000
2005
2010

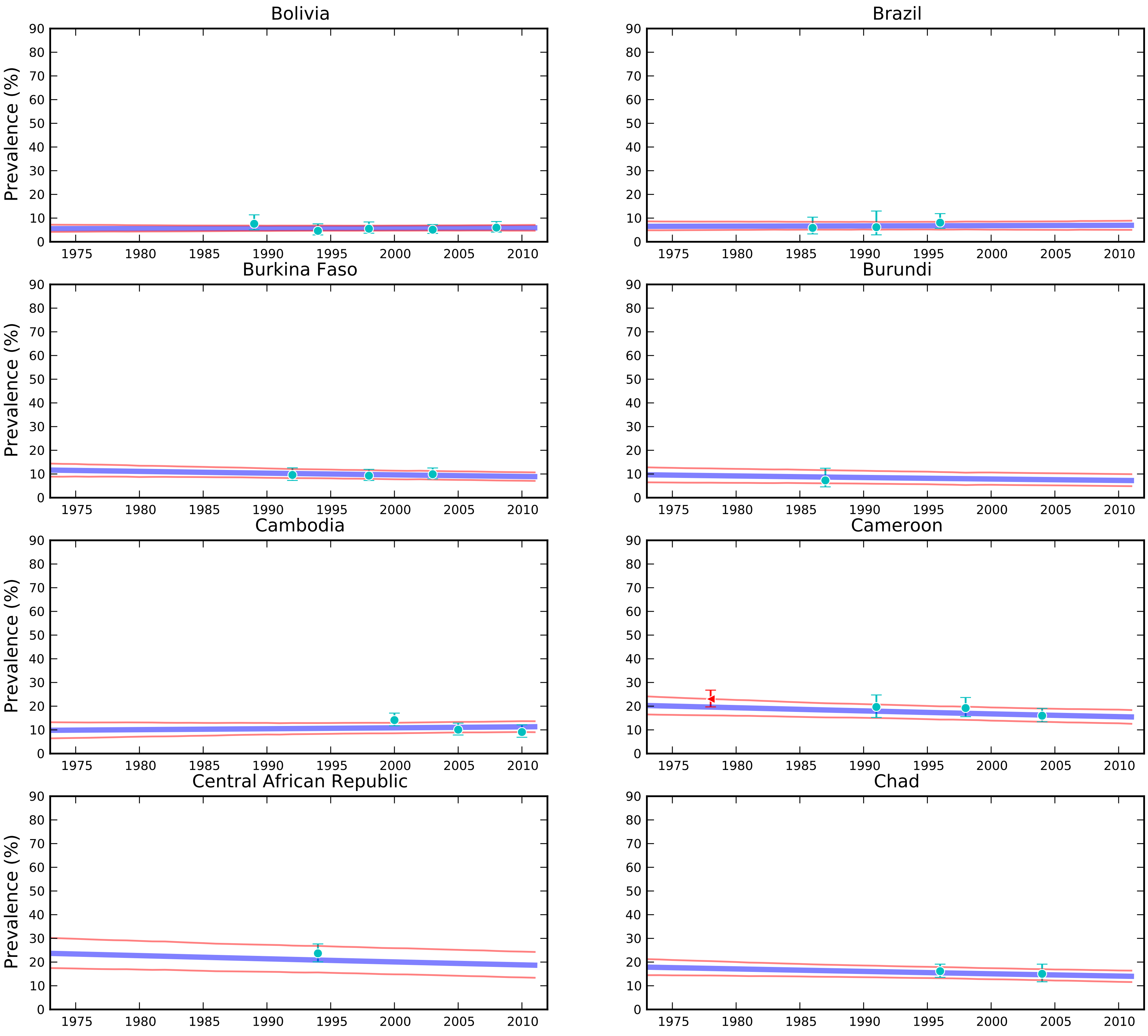
Bolivia
Brazil
Burkina Faso
Burundi
Cambodia
Cameroon
Central African Republic
Chad
Prevalence (%)
0 10 20 30 40 50 60 70 80 90
1975 1980 1985 1990 1995 2000 2005 2010

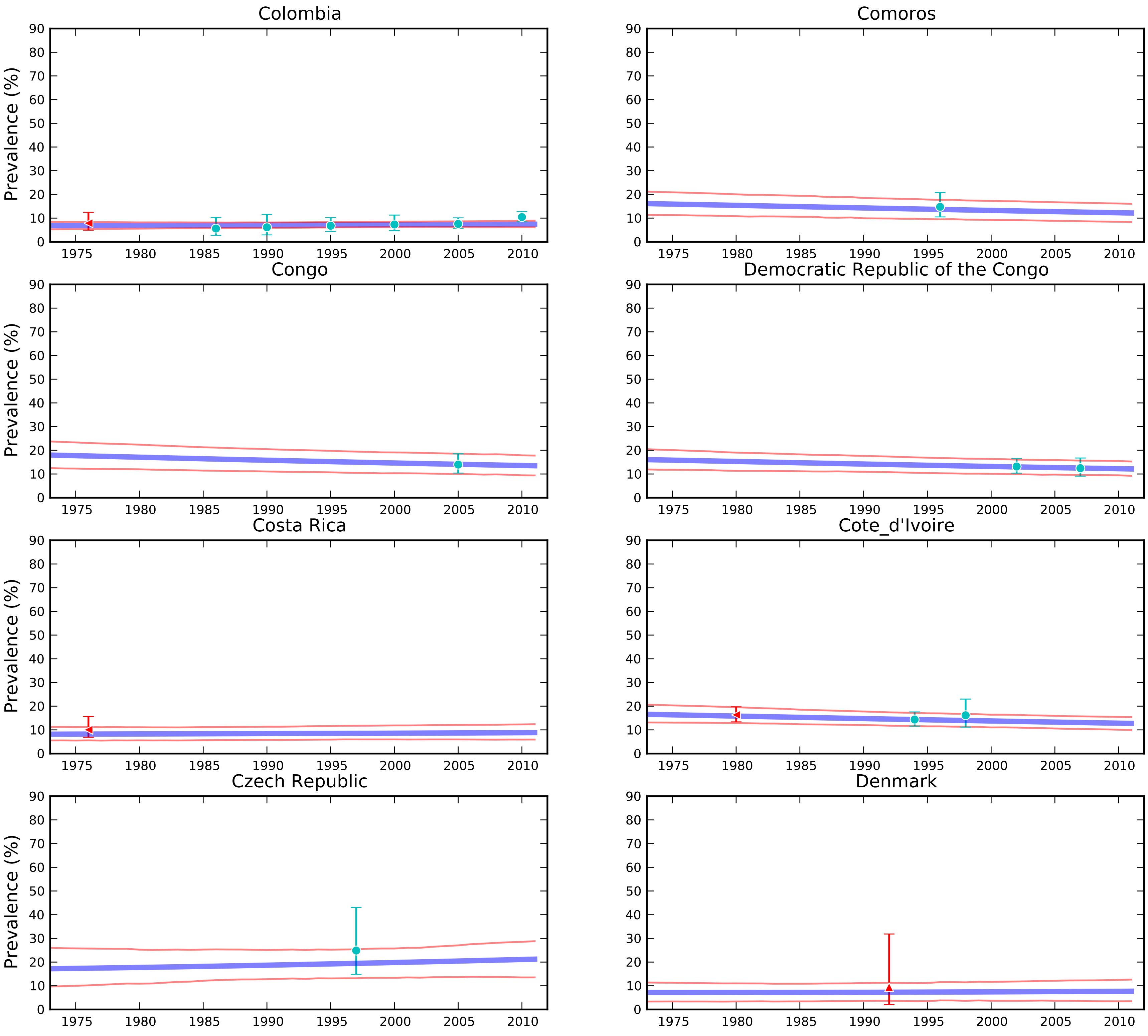
Colombia
Comoros
Congo
Democratic Republic of the Congo
Costa Rica
Cote_d'Ivoire
Czech Republic
Denmark
Prevalence (%)
0
10
20
30
40
50
60
70
80
90
1975
1980
1985
1990
1995
2000
2005
2010

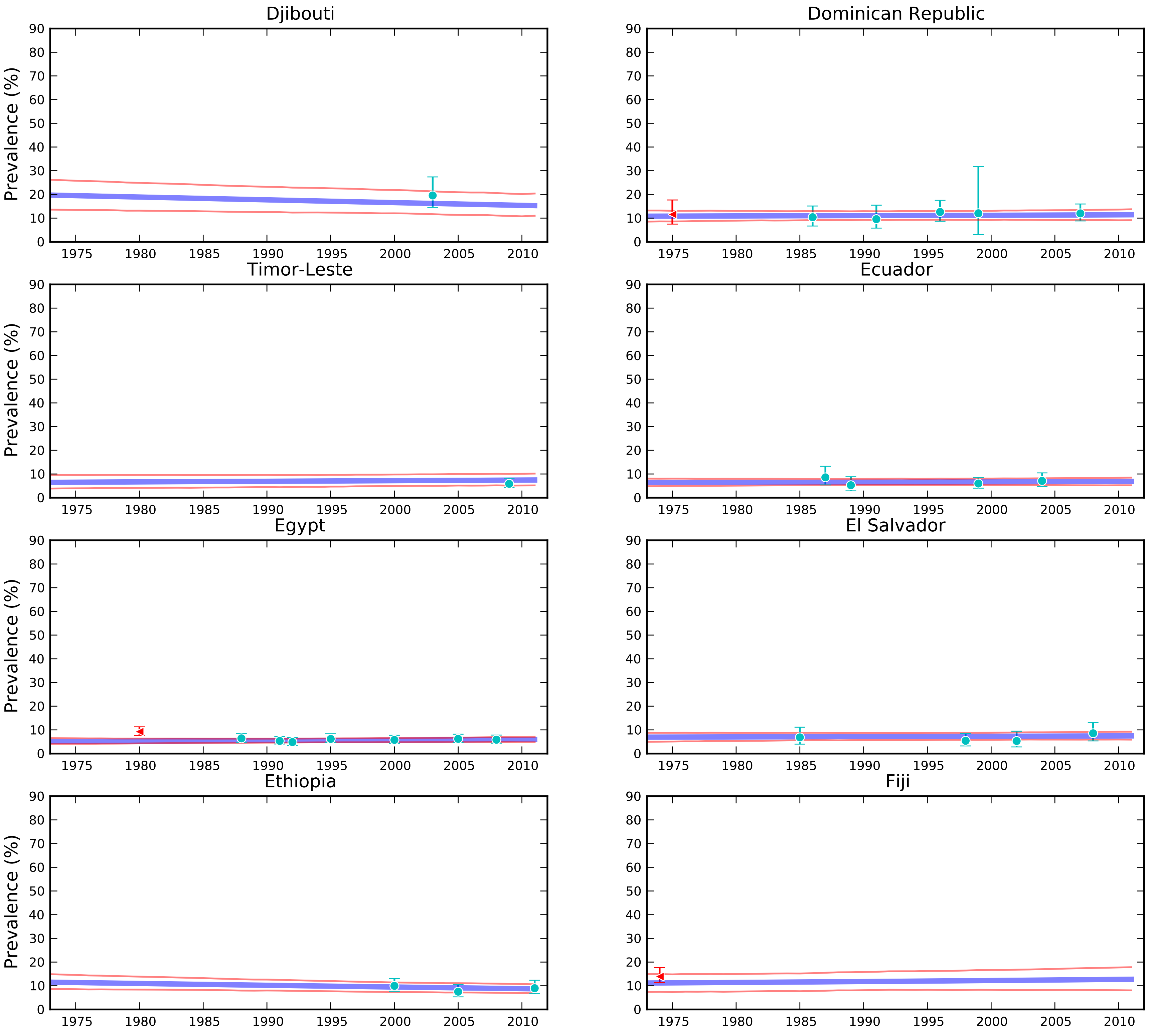
Djibouti
Dominican Republic
Timor-Leste
Ecuador
Egypt
El Salvador
Ethiopia
Fiji
Prevalence (%)
0 10 20 30 40 50 60 70 80 90
1975 1980 1985 1990 1995 2000 2005 2010

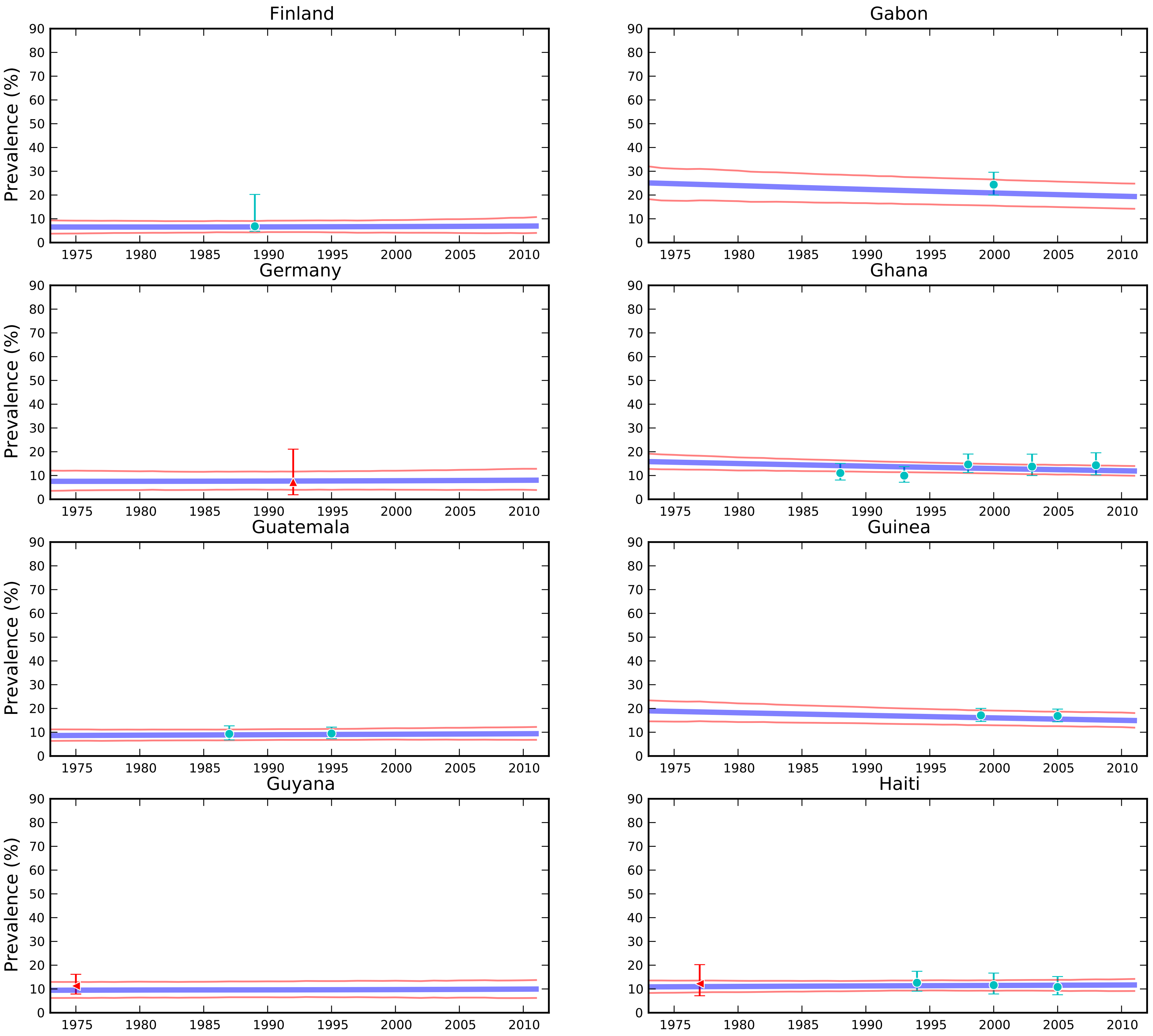

Finland
Gabon
Germany
Ghana
Guatemala
Guinea
Guyana
Haiti
Prevalence (%)
0
10
20
30
40
50
60
70
80
90
1975
1980
1985
1990
1995
2000
2005
2010

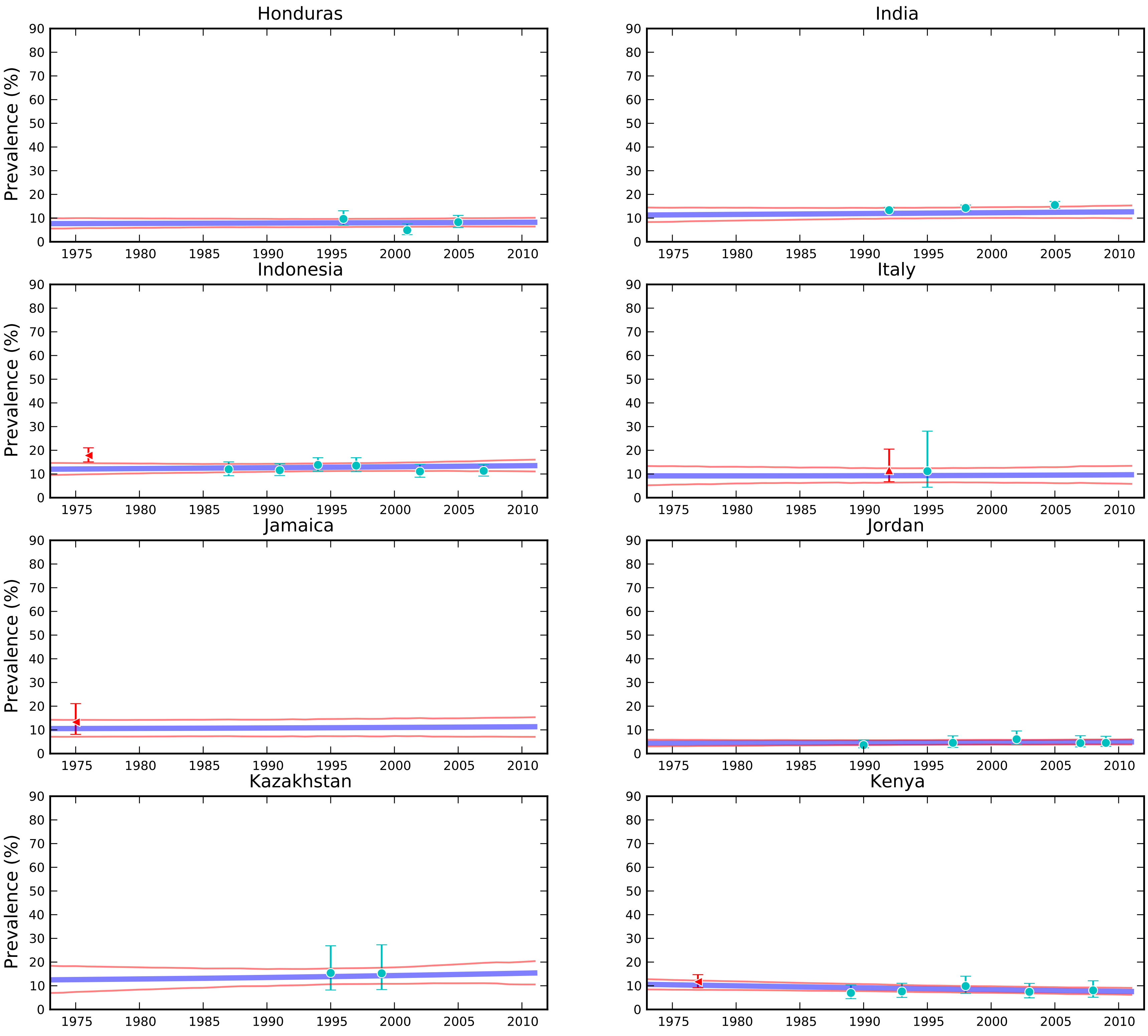
Honduras
India
Indonesia
Italy
Jamaica
Jordan
Kazakhstan
Kenya
Prevalence (%)
0
10
20
30
40
50
60
70
80
90
1975
1980
1985
1990
1995
2000
2005
2010

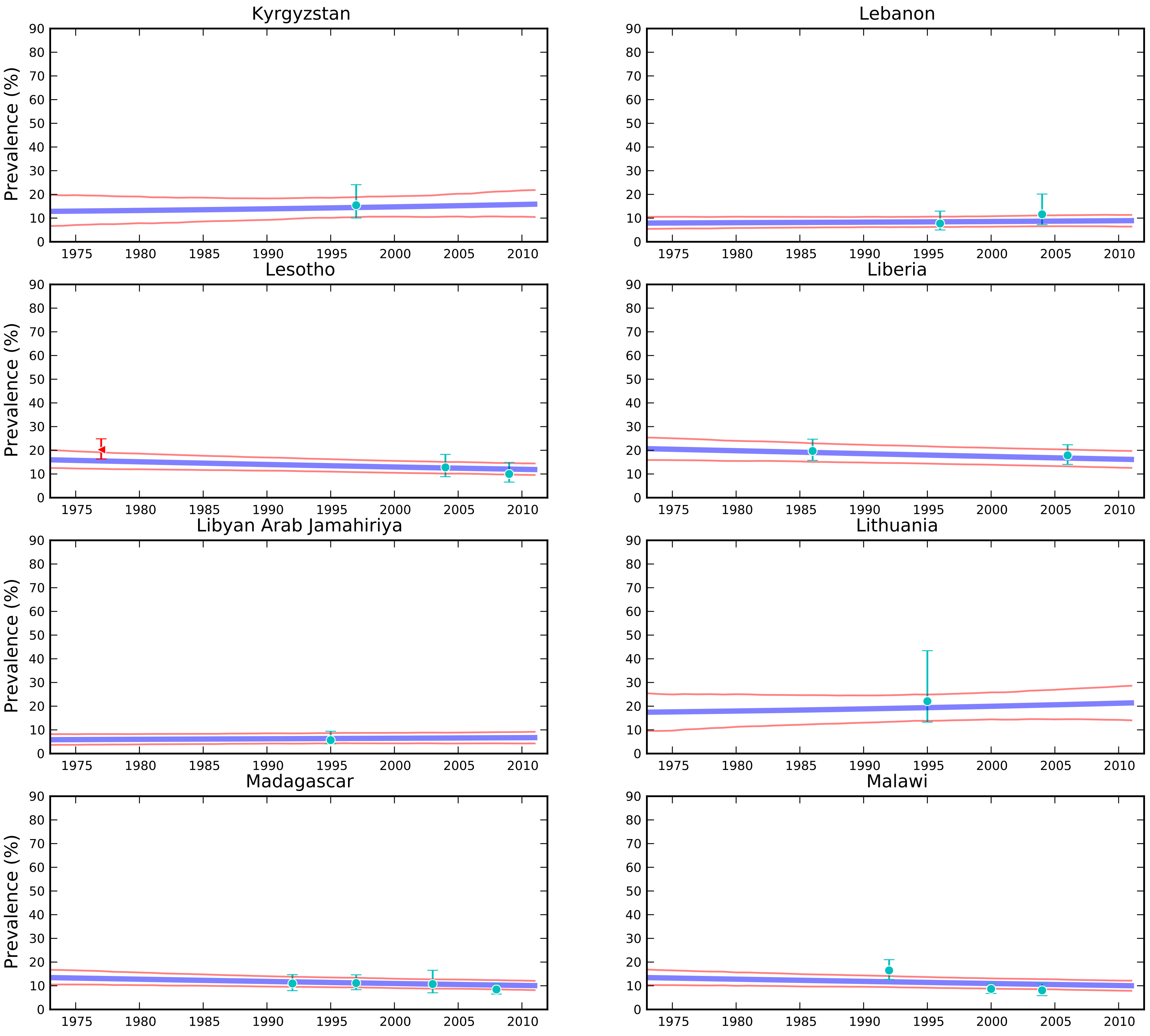
Kyrgyzstan
Lebanon
Lesotho
Liberia
Libyan Arab Jamahiriya
Lithuania
Madagascar
Malawi
Prevalence (%)
0
10
20
30
40
50
60
70
80
90
1975
1980
1985
1990
1995
2000
2005
2010

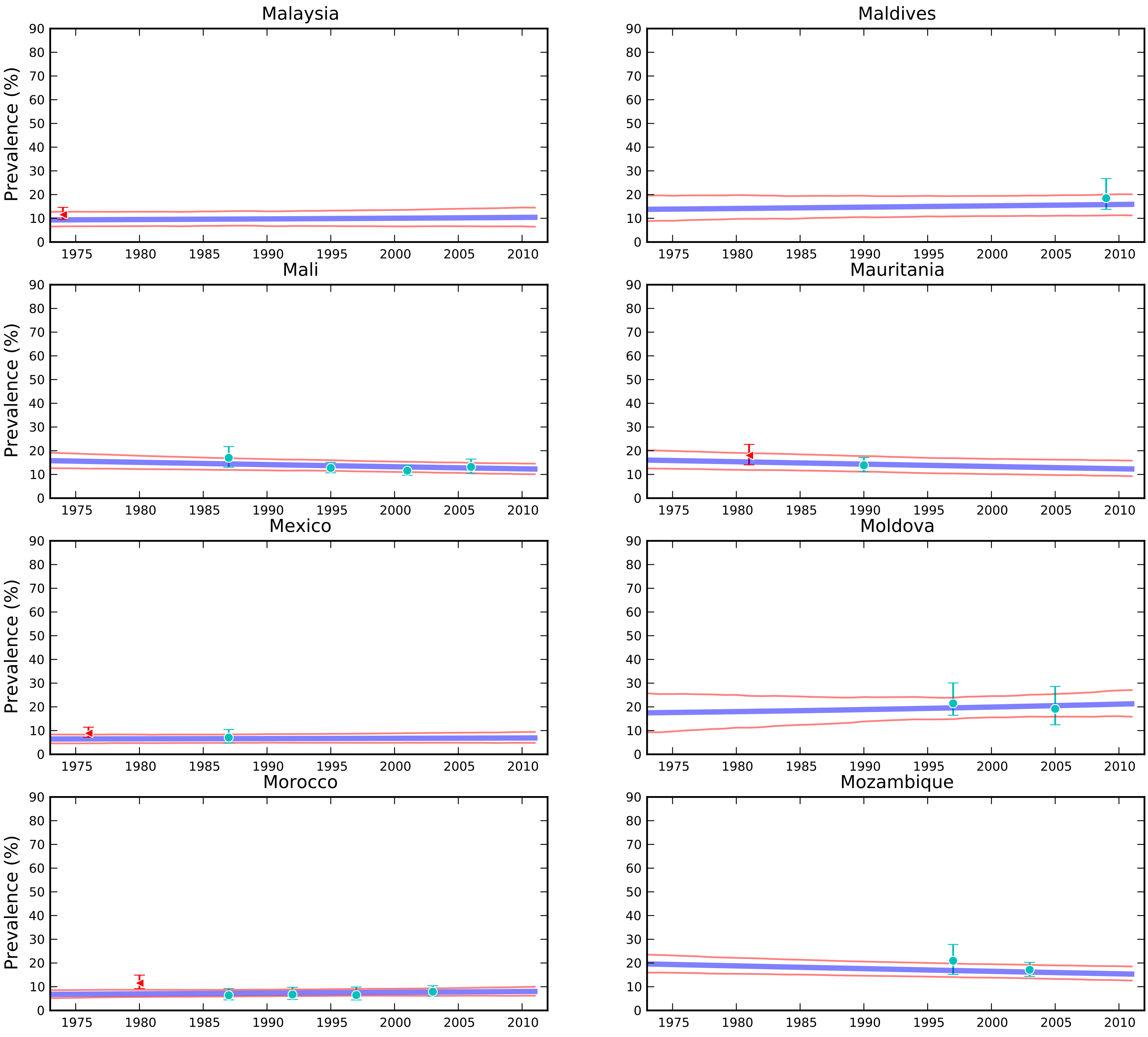
Malaysia
Maldives
Mali
Mauritania
Mexico
Moldova
Morocco
Mozambique
Prevalence (%)
0 10 20 30 40 50 60 70 80 90
1975 1980 1985 1990 1995 2000 2005 2010

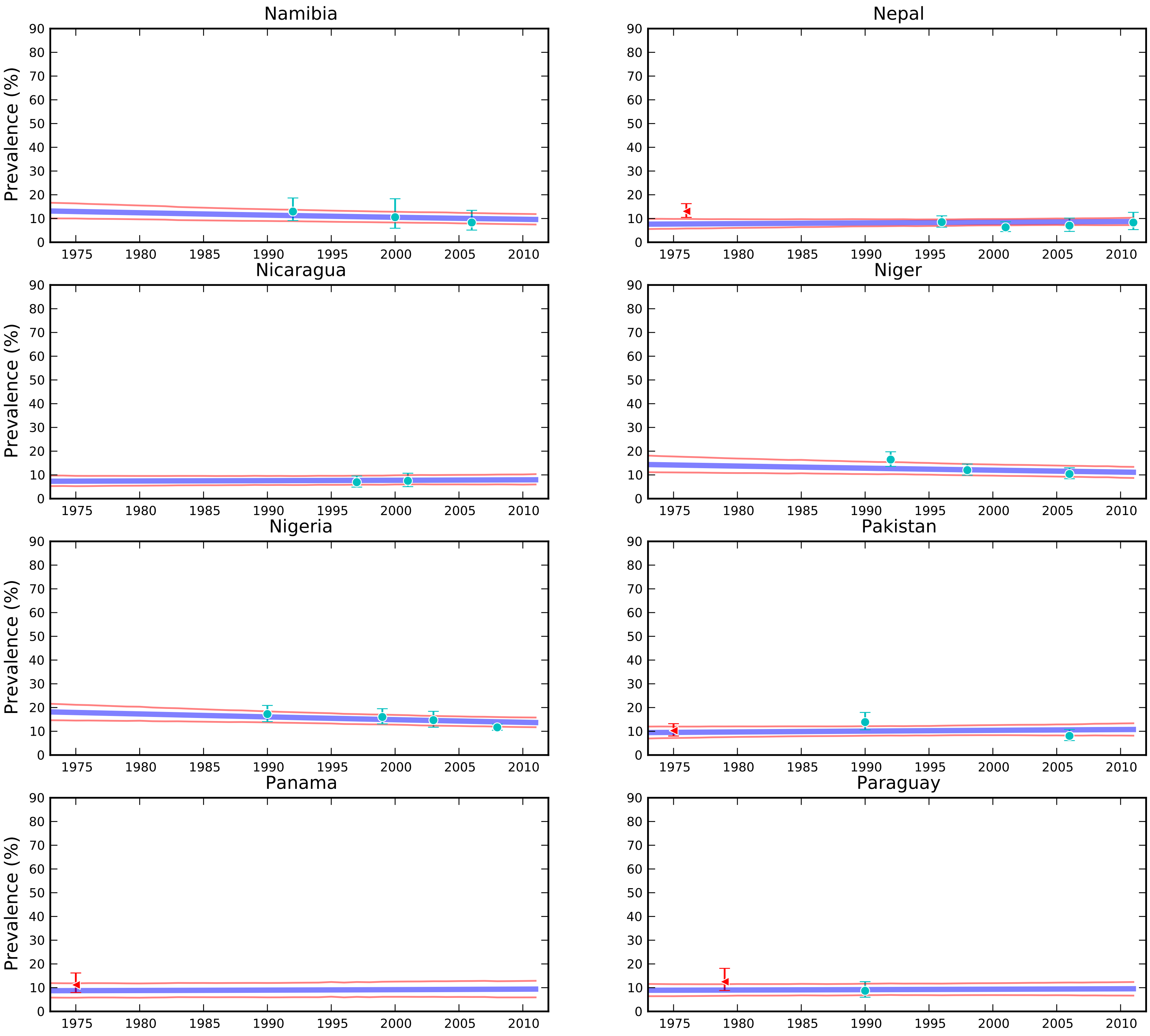
Namibia
Nepal
Nicaragua
Niger
Nigeria
Pakistan
Panama
Paraguay
Prevalence (%)
0 10 20 30 40 50 60 70 80 90
1975 1980 1985 1990 1995 2000 2005 2010

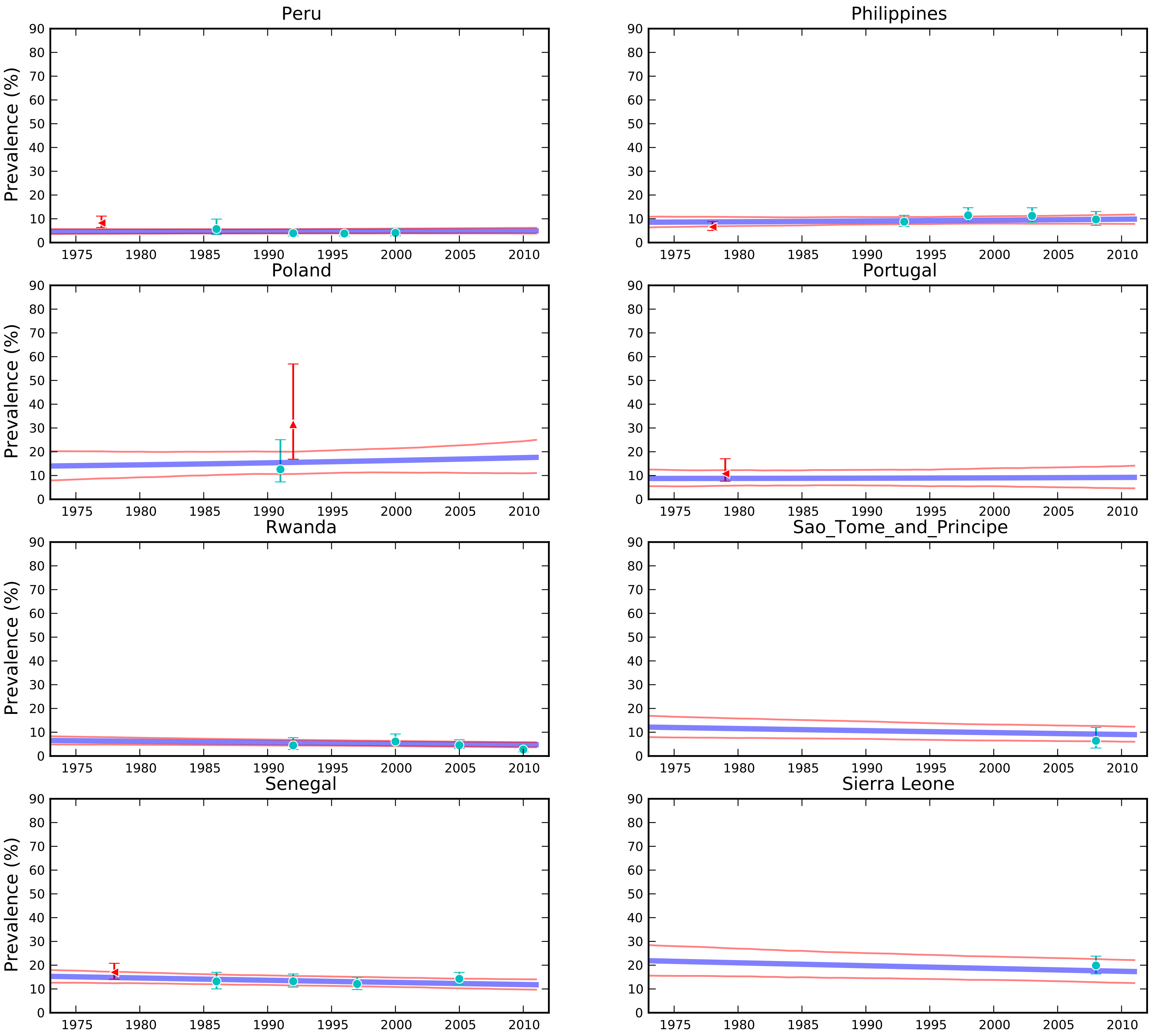
Peru
Philippines
Poland
Portugal
Rwanda
Sao_Tome_and_Principe
Senegal
Sierra Leone
Prevalence (%)
0 10 20 30 40 50 60 70 80 90
1975 1980 1985 1990 1995 2000 2005 2010

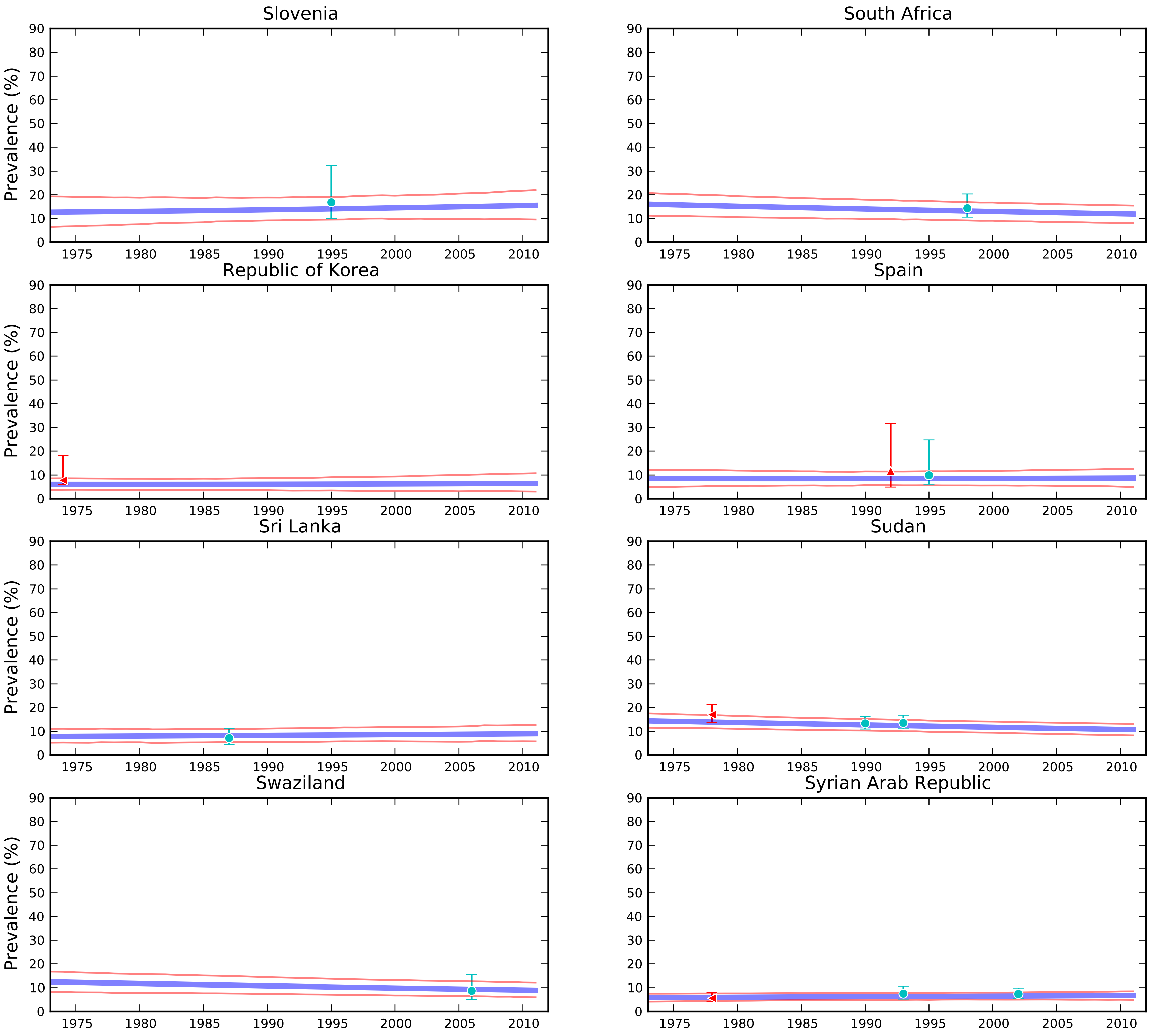
Slovenia
South Africa
Republic of Korea
Spain
Sri Lanka
Sudan
Swaziland
Syrian Arab Republic
Prevalence (%)
0
10
20
30
40
50
60
70
80
90
1975
1980
1985
1990
1995
2000
2005
2010

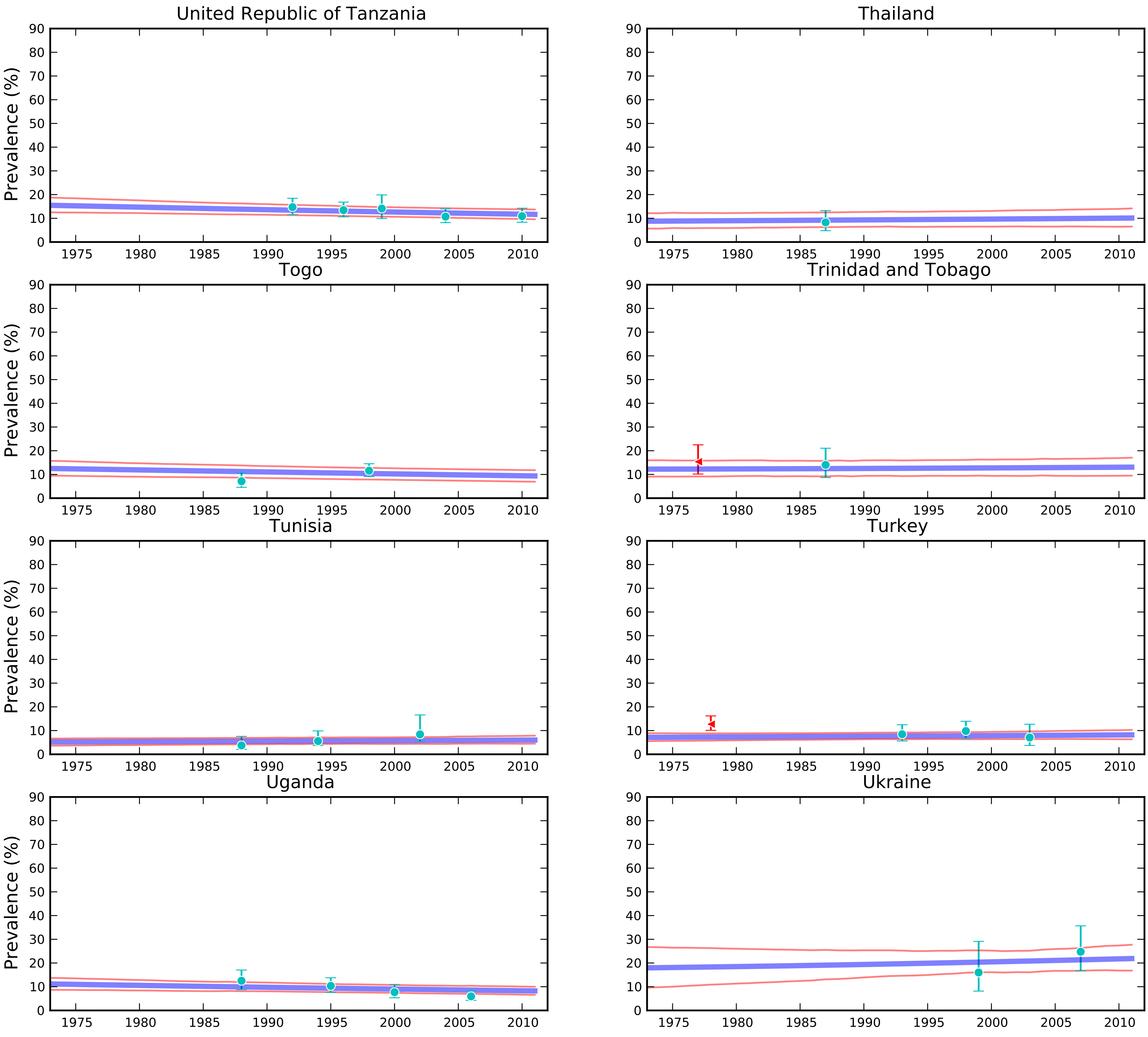
United Republic of Tanzania
Thailand
Togo
Trinidad and Tobago
Tunisia
Turkey
Uganda
Ukraine
Prevalence (%)
0 10 20 30 40 50 60 70 80 90
1975 1980 1985 1990 1995 2000 2005 2010

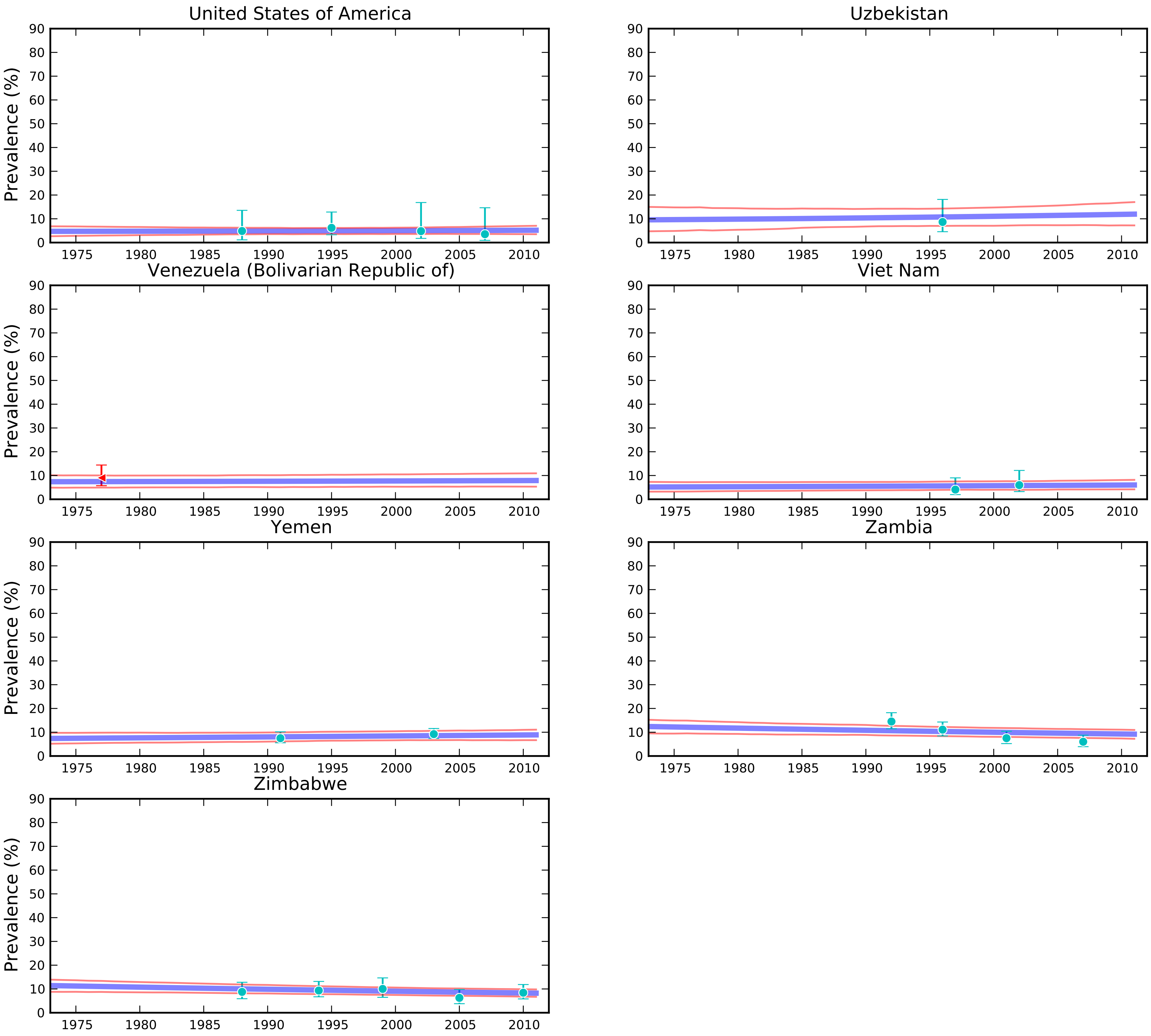
United States of America
Uzbekistan
Venezuela (Bolivarian Republic of)
Viet Nam
Yemen
Zambia
Zimbabwe
Prevalence (%)
0
10
20
30
40
50
60
70
80
90
1975
1980
1985
1990
1995
2000
2005
2010

Figure H: Trends in exposure to primary infertility by year, shown for each country with data on following pages. Infertility is indexed on the female partner; age-standardized prevalence among women aged 20-44 years is shown here. Legend for plots is given below.

- Nationally representative (error bars represent standard errors)
- ■ Nationally representative, data do not cover the entire 20-44 year age range
- ▶ Subnational
- ▲ Subnational, data do not cover the entire 20-44 year age range
- ◀ World Fertility Survey
- ▼ World Fertility Survey, data do not cover the entire 20-44 year age range
- Posterior means (model predictions)
- 95% credible interval

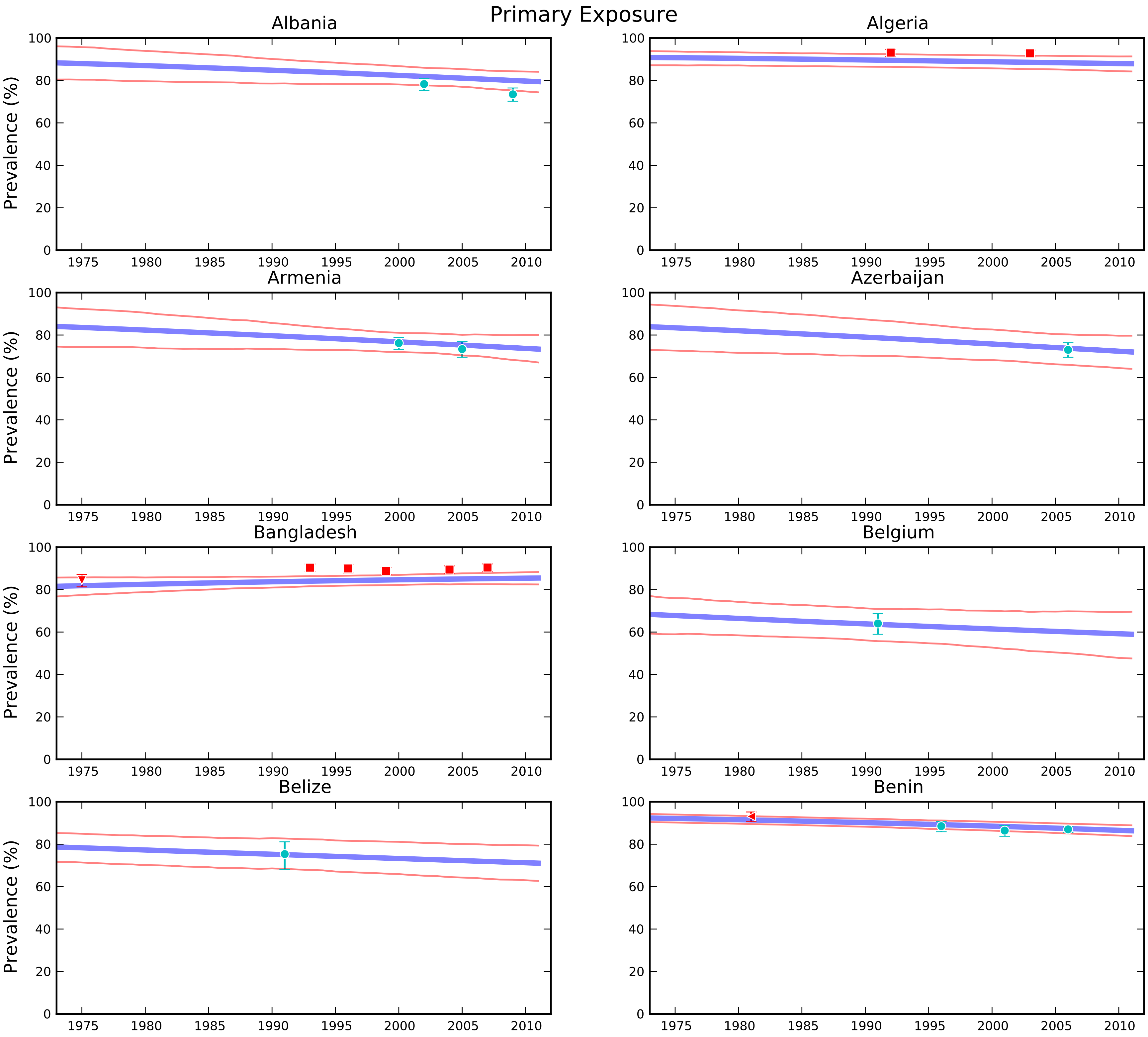
Primary Exposure
Albania
Algeria
Armenia
Azerbaijan
Bangladesh
Belgium
Belize
Benin
Prevalence (%)
0
20
40
60
80
100
1975
1980
1985
1990
1995
2000
2005
2010

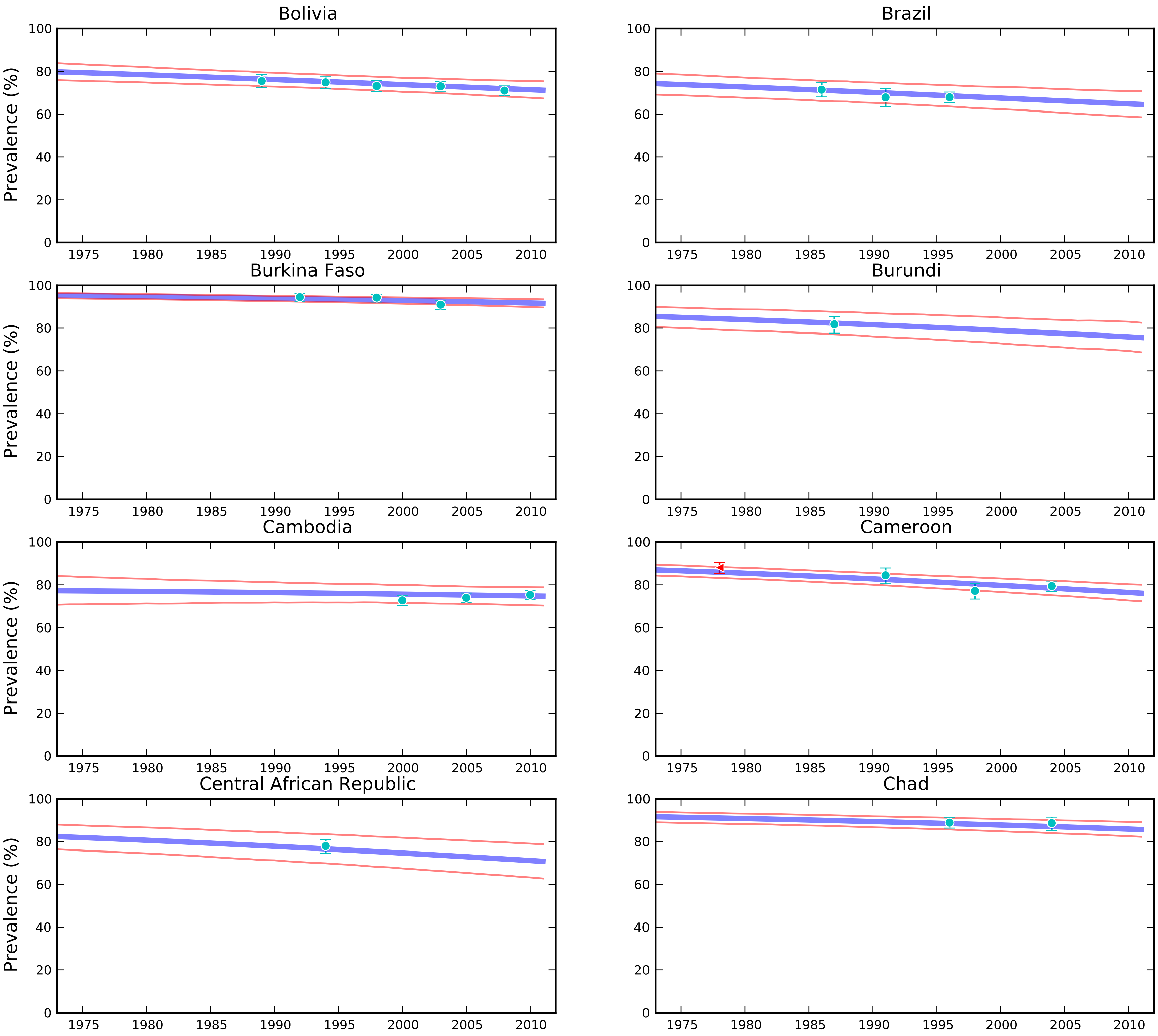
Bolivia
Brazil
Burkina Faso
Burundi
Cambodia
Cameroon
Central African Republic
Chad
Prevalence (%)
0
20
40
60
80
100
1975
1980
1985
1990
1995
2000
2005
2010

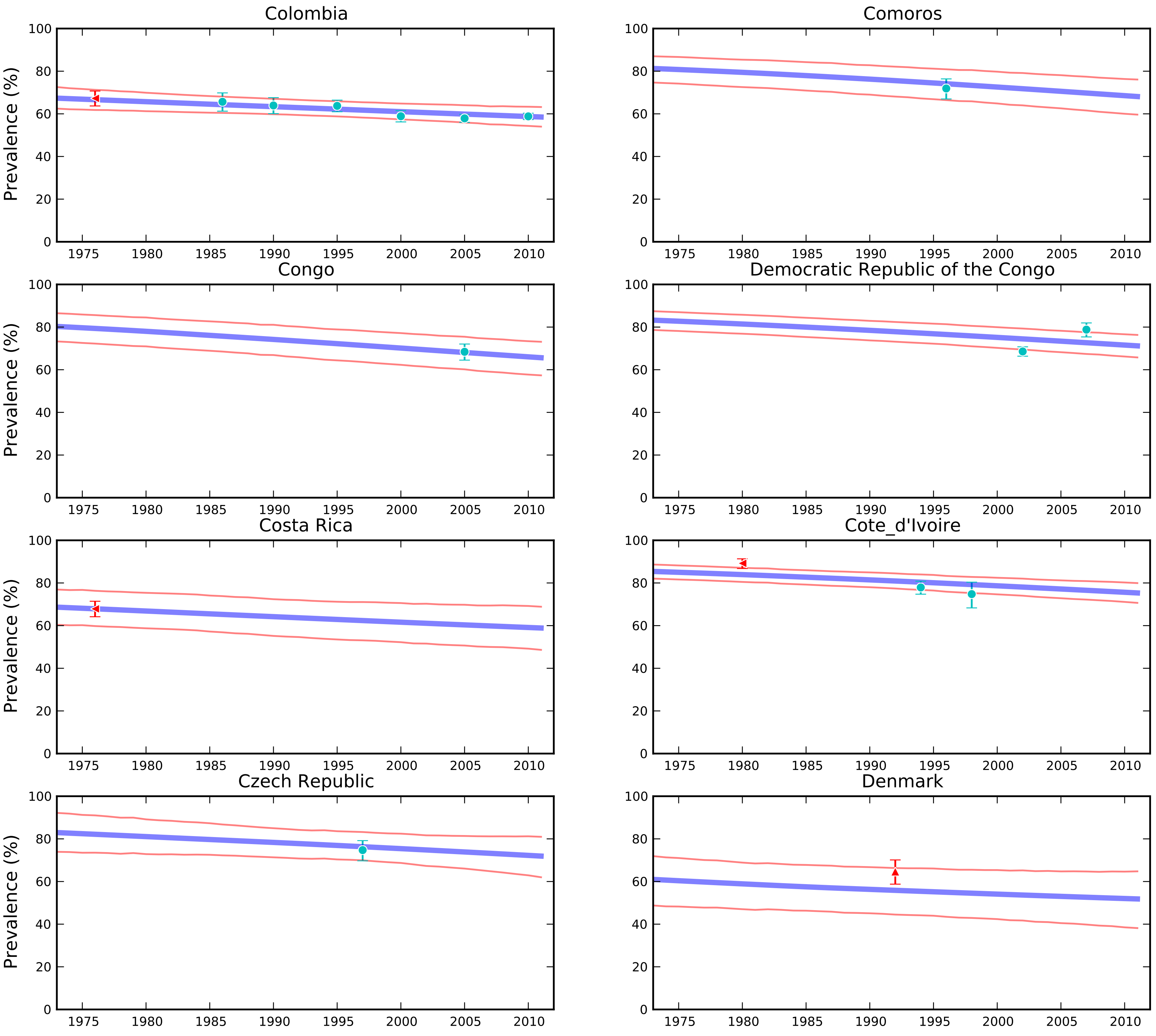
Colombia
Comoros
Congo
Democratic Republic of the Congo
Costa Rica
Cote_d'Ivoire
Czech Republic
Denmark
Prevalence (%)
0
20
40
60
80
100
1975
1980
1985
1990
1995
2000
2005
2010

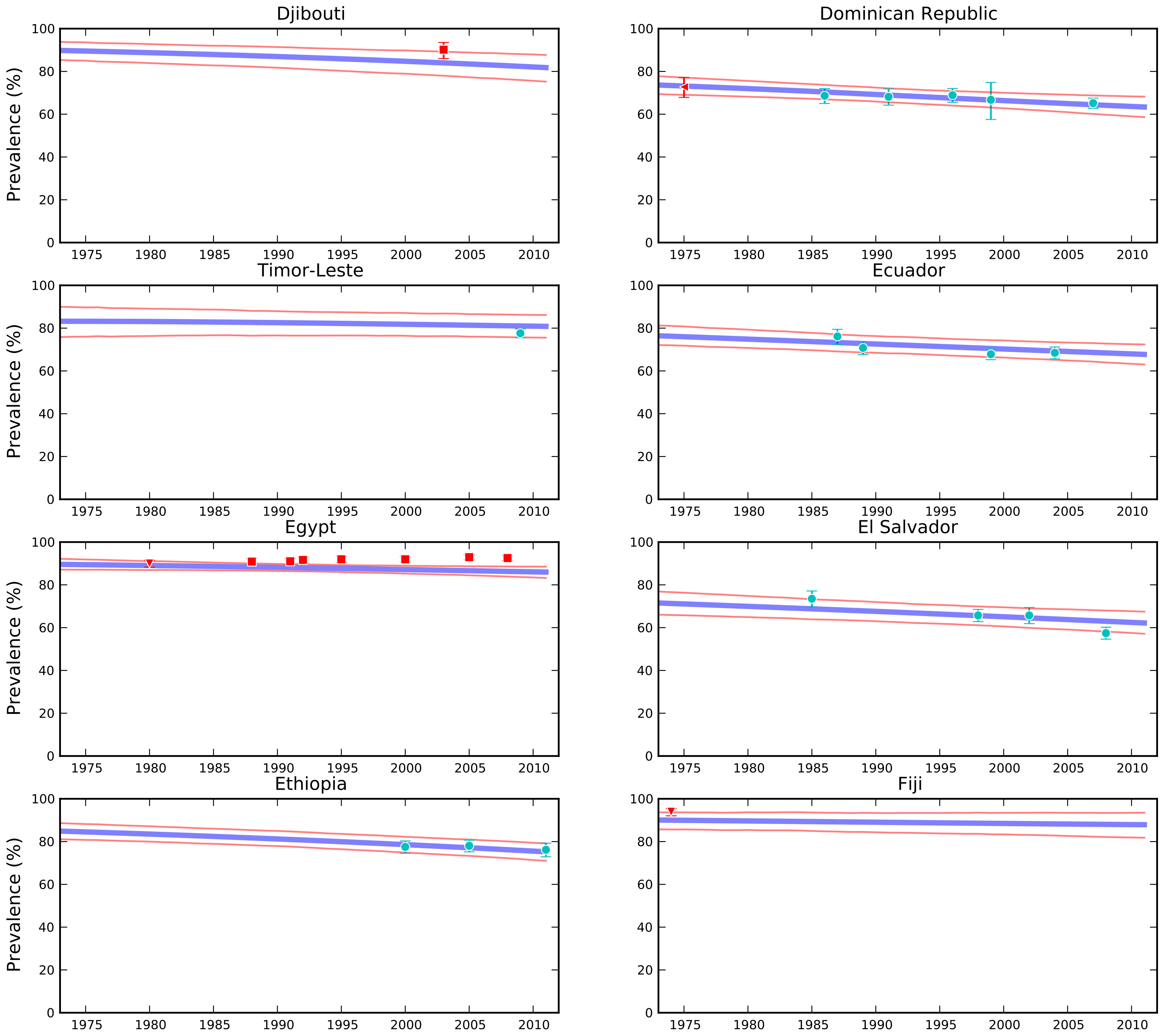
Djibouti
Dominican Republic
Timor-Leste
Ecuador
Egypt
El Salvador
Ethiopia
Fiji
Prevalence (%)
0
20
40
60
80
100
1975
1980
1985
1990
1995
2000
2005
2010

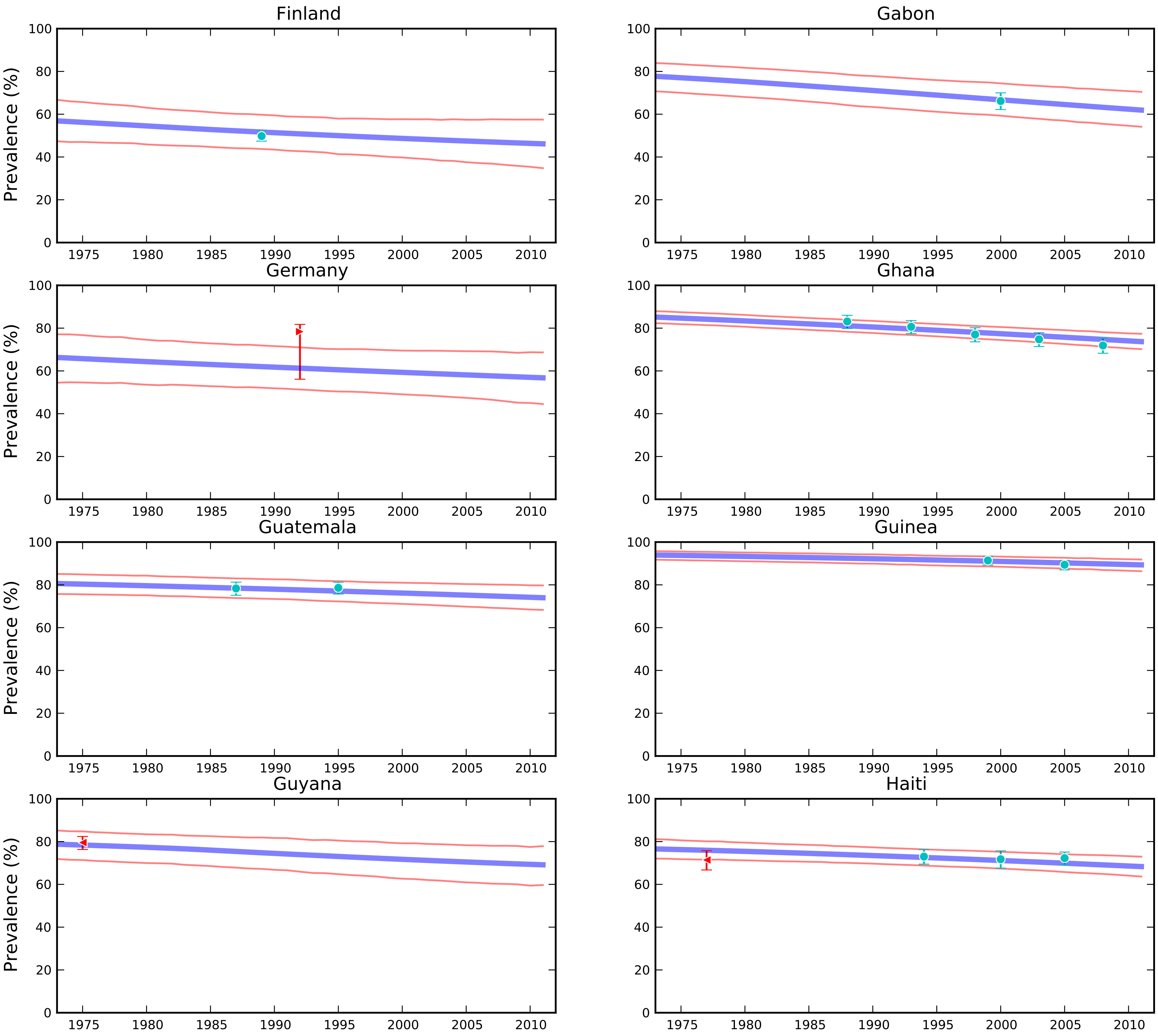
Finland
Gabon
Germany
Ghana
Guatemala
Guinea
Guyana
Haiti
Prevalence (%)
0
20
40
60
80
100
1975
1980
1985
1990
1995
2000
2005
2010

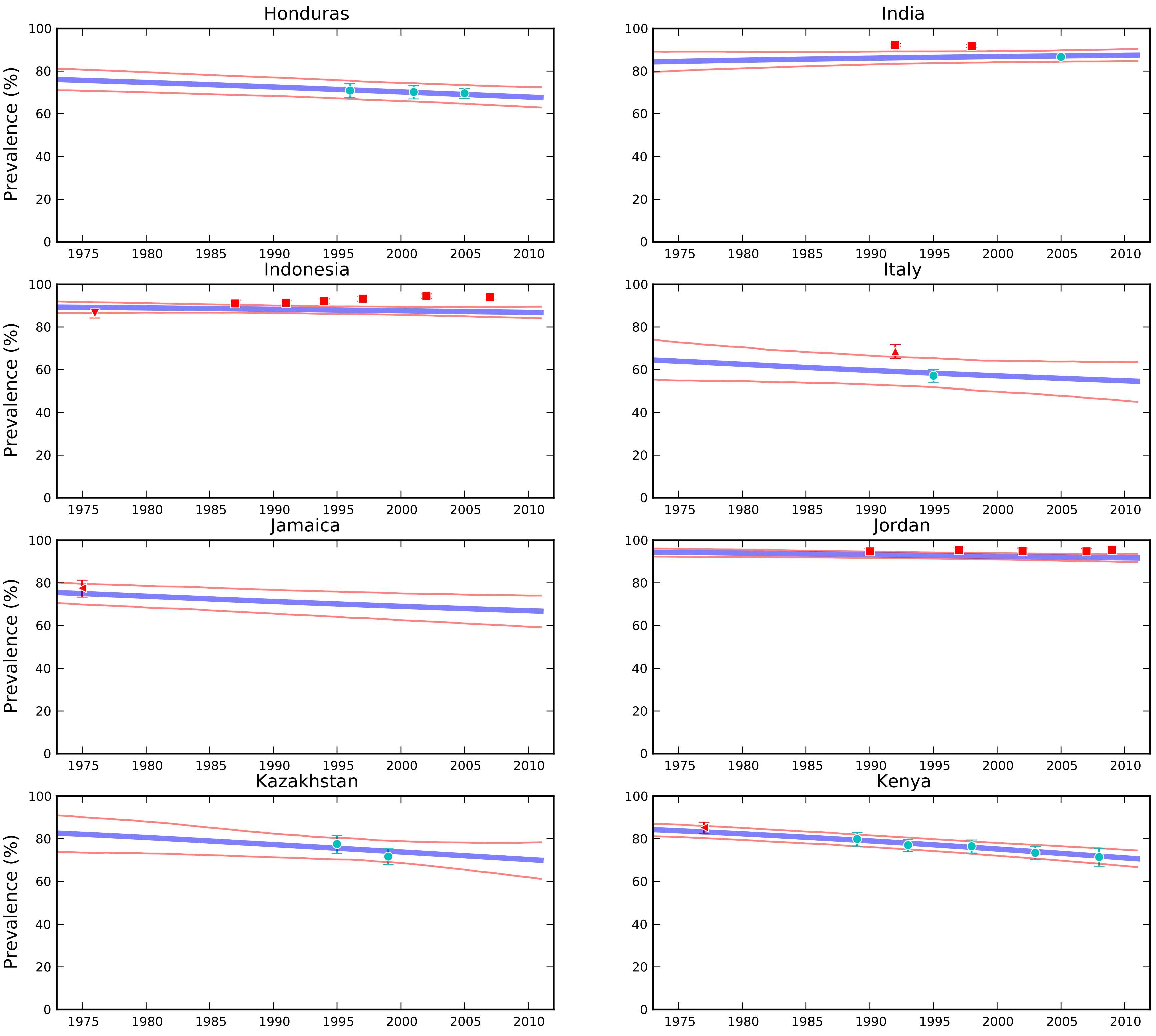
Honduras
India
Indonesia
Italy
Jamaica
Jordan
Kazakhstan
Kenya
Prevalence (%)
0
20
40
60
80
100
1975
1980
1985
1990
1995
2000
2005
2010

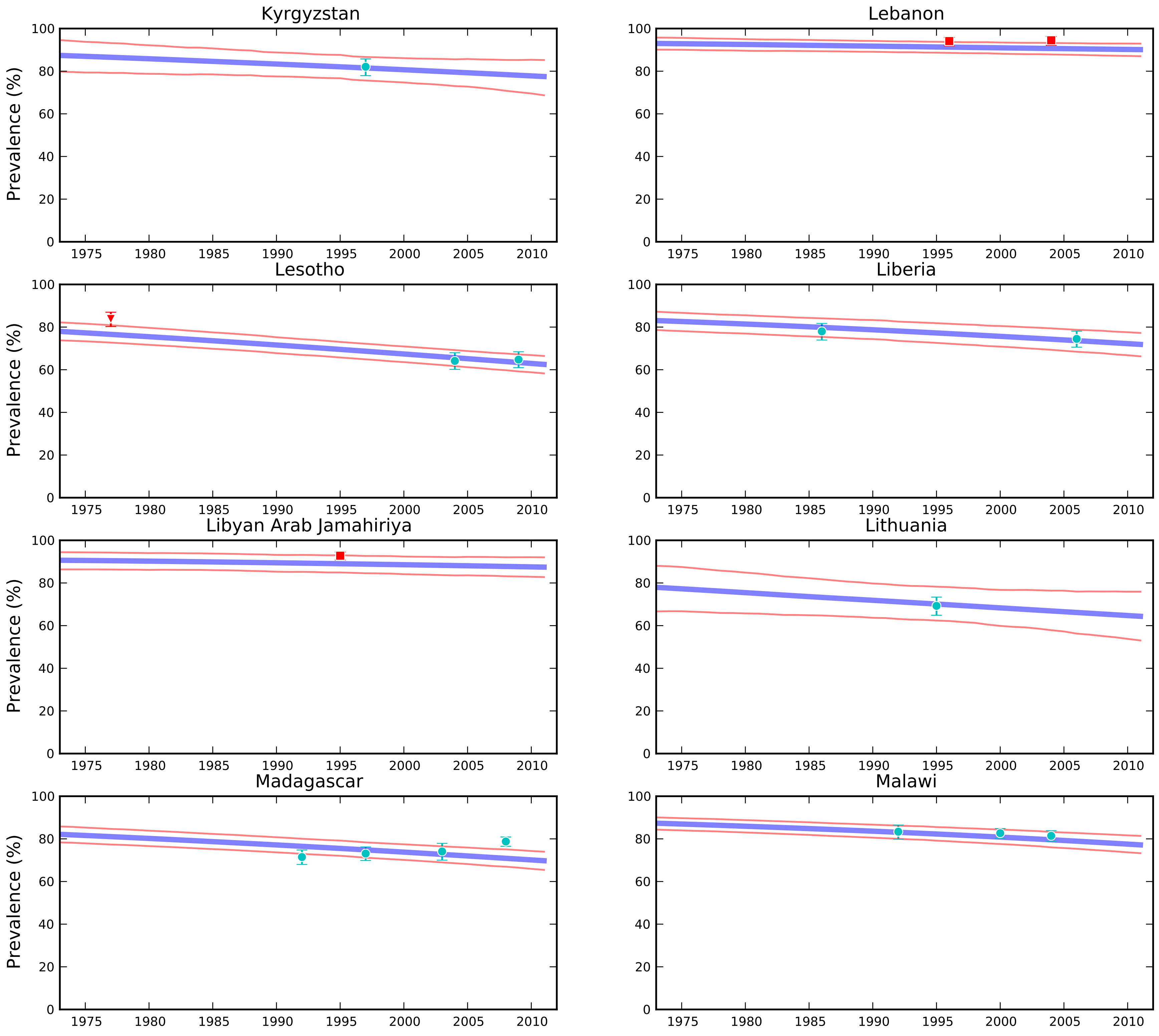
Kyrgyzstan
Lebanon
Lesotho
Liberia
Libyan Arab Jamahiriya
Lithuania
Madagascar
Malawi
Prevalence (%)
0
20
40
60
80
100
1975
1980
1985
1990
1995
2000
2005
2010

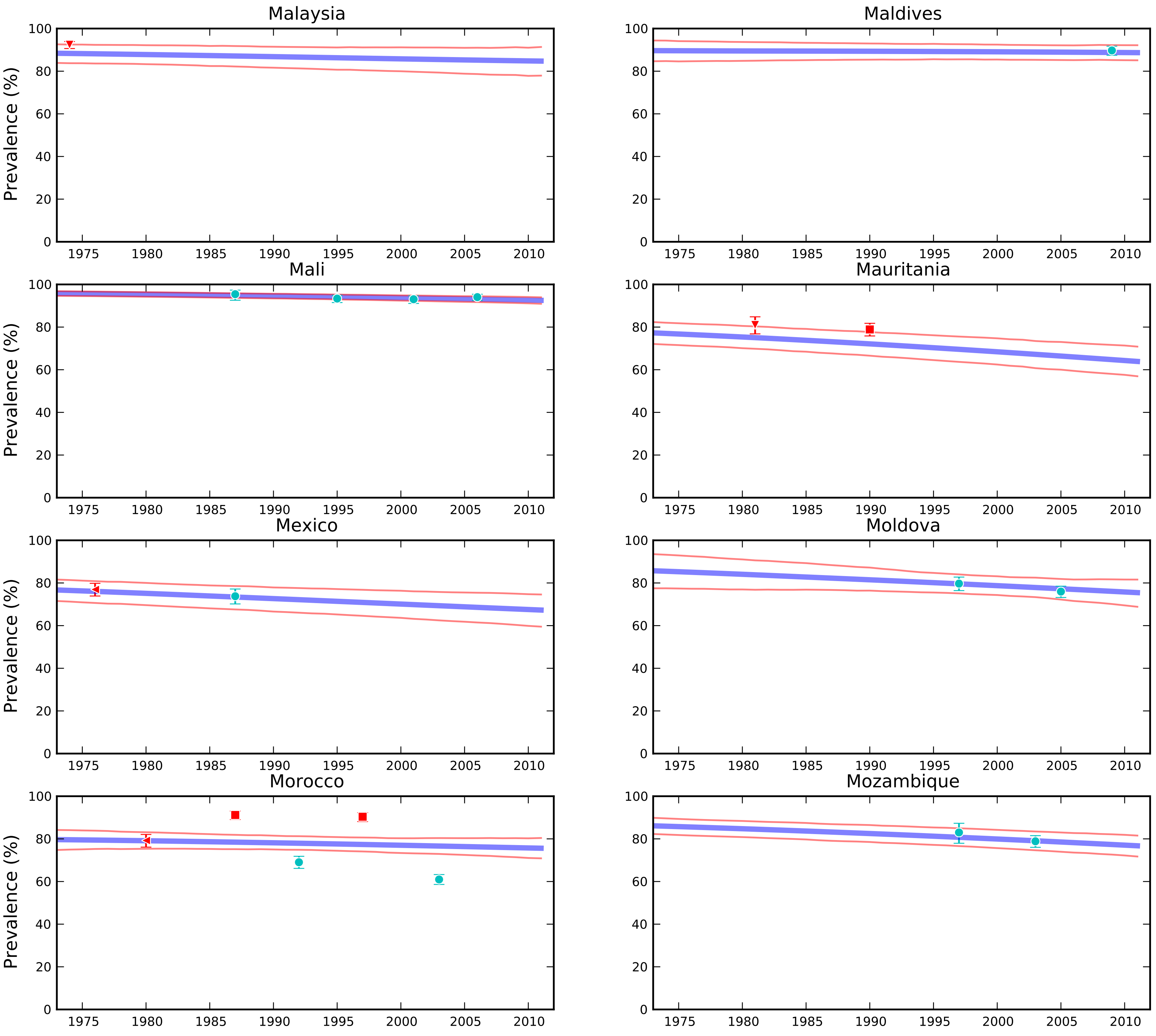
Malaysia
Maldives
Mali
Mauritania
Mexico
Moldova
Morocco
Mozambique
Prevalence (%)
0
20
40
60
80
100
1975
1980
1985
1990
1995
2000
2005
2010

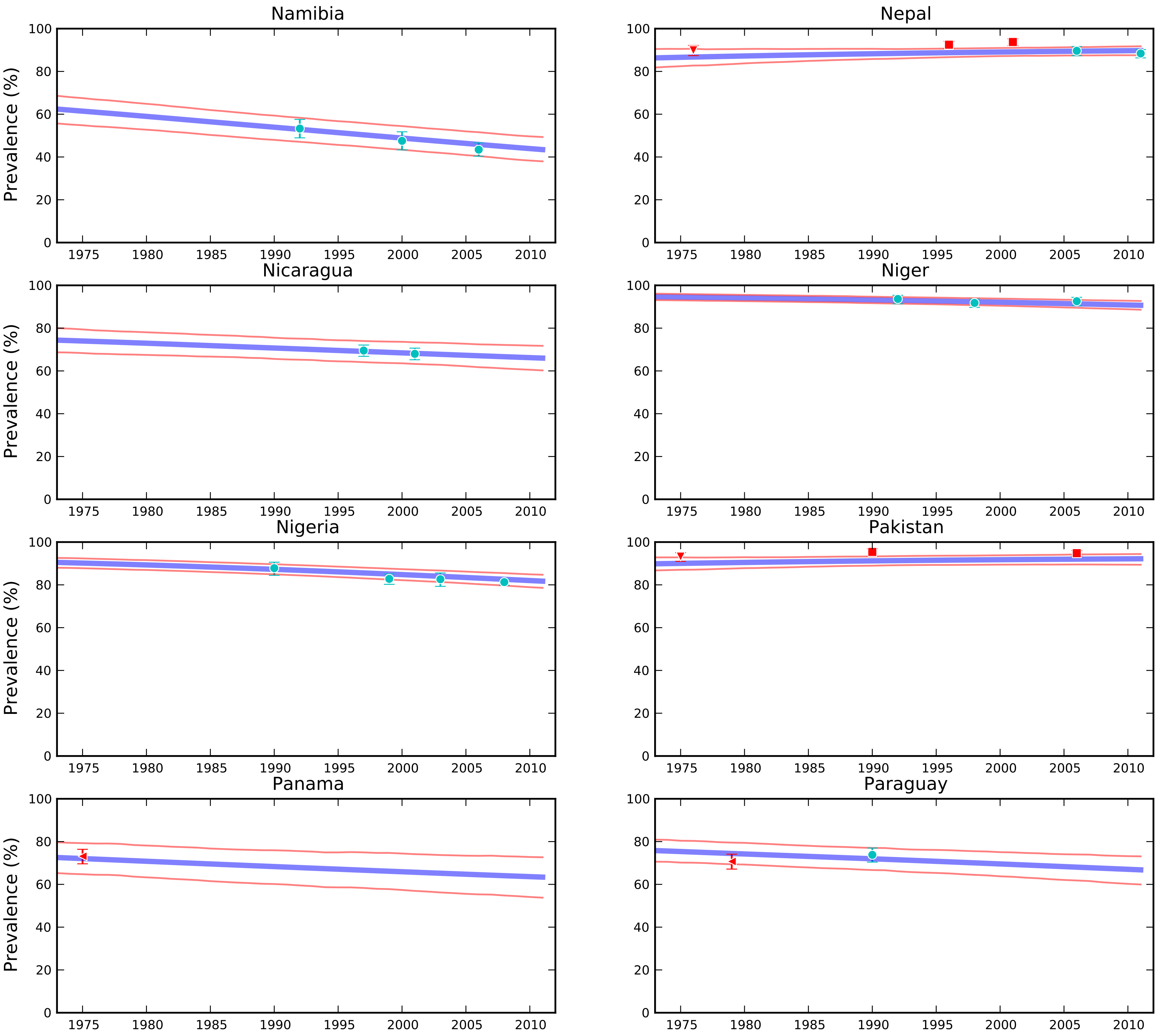
Namibia
Nepal
Nicaragua
Niger
Nigeria
Pakistan
Panama
Paraguay
Prevalence (%)
0
20
40
60
80
100
1975
1980
1985
1990
1995
2000
2005
2010

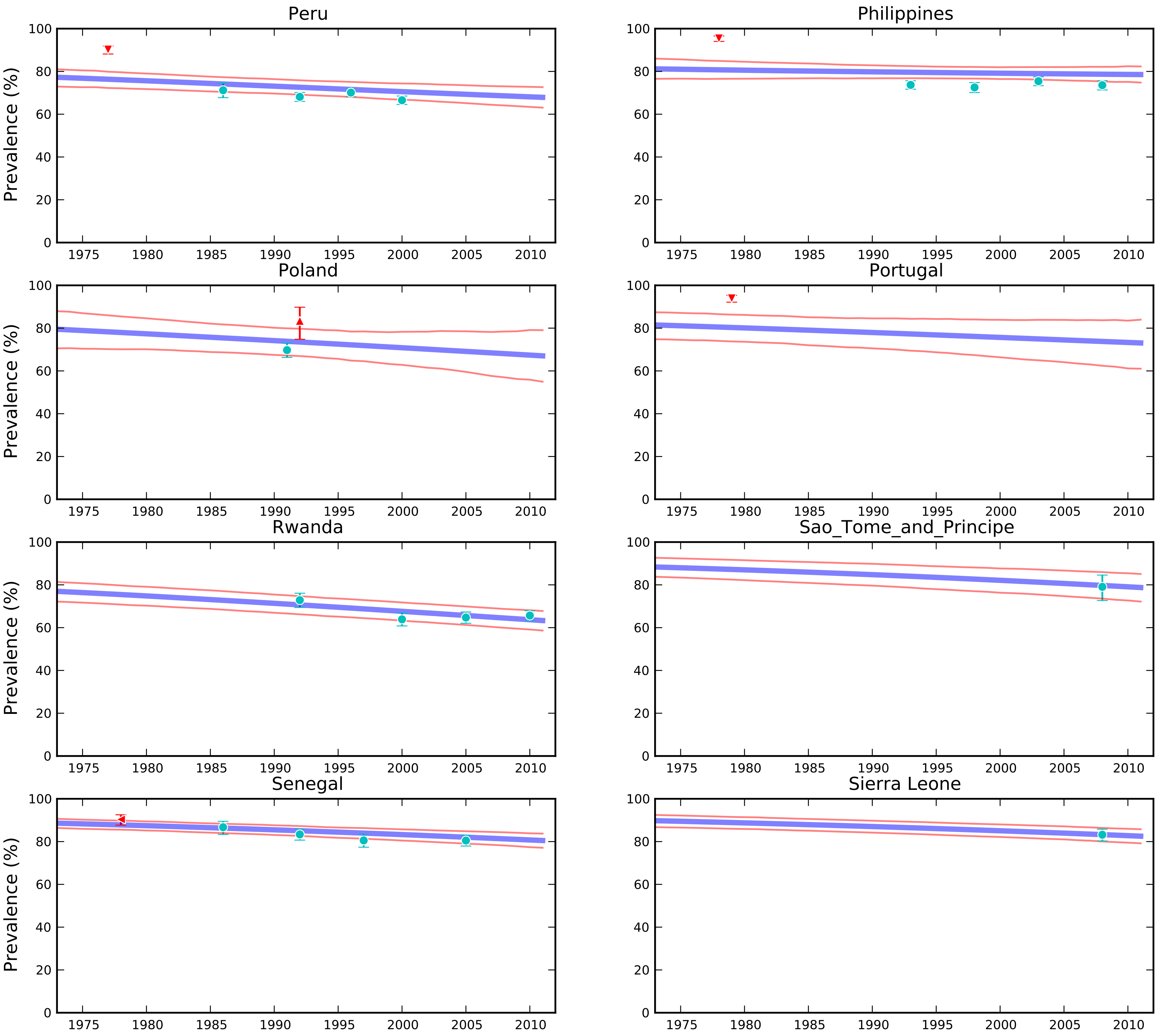

Peru
Philippines
Poland
Portugal
Rwanda
Sao_Tome_and_Principe
Senegal
Sierra Leone
Prevalence (%)
0
20
40
60
80
100
1975
1980
1985
1990
1995
2000
2005
2010

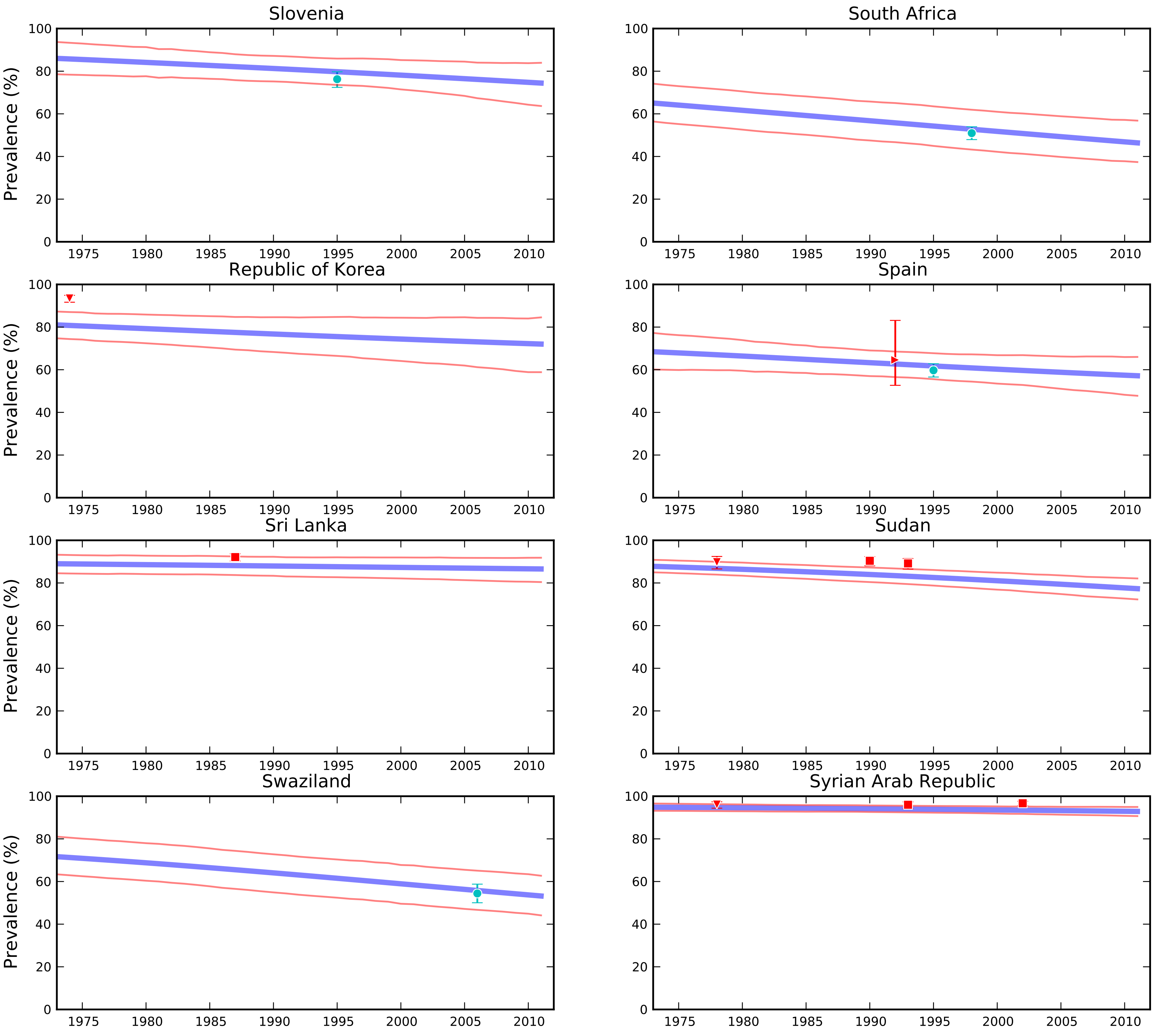
Slovenia
South Africa
Republic of Korea
Spain
Sri Lanka
Sudan
Swaziland
Syrian Arab Republic
Prevalence (%)
0
20
40
60
80
100
1975
1980
1985
1990
1995
2000
2005
2010

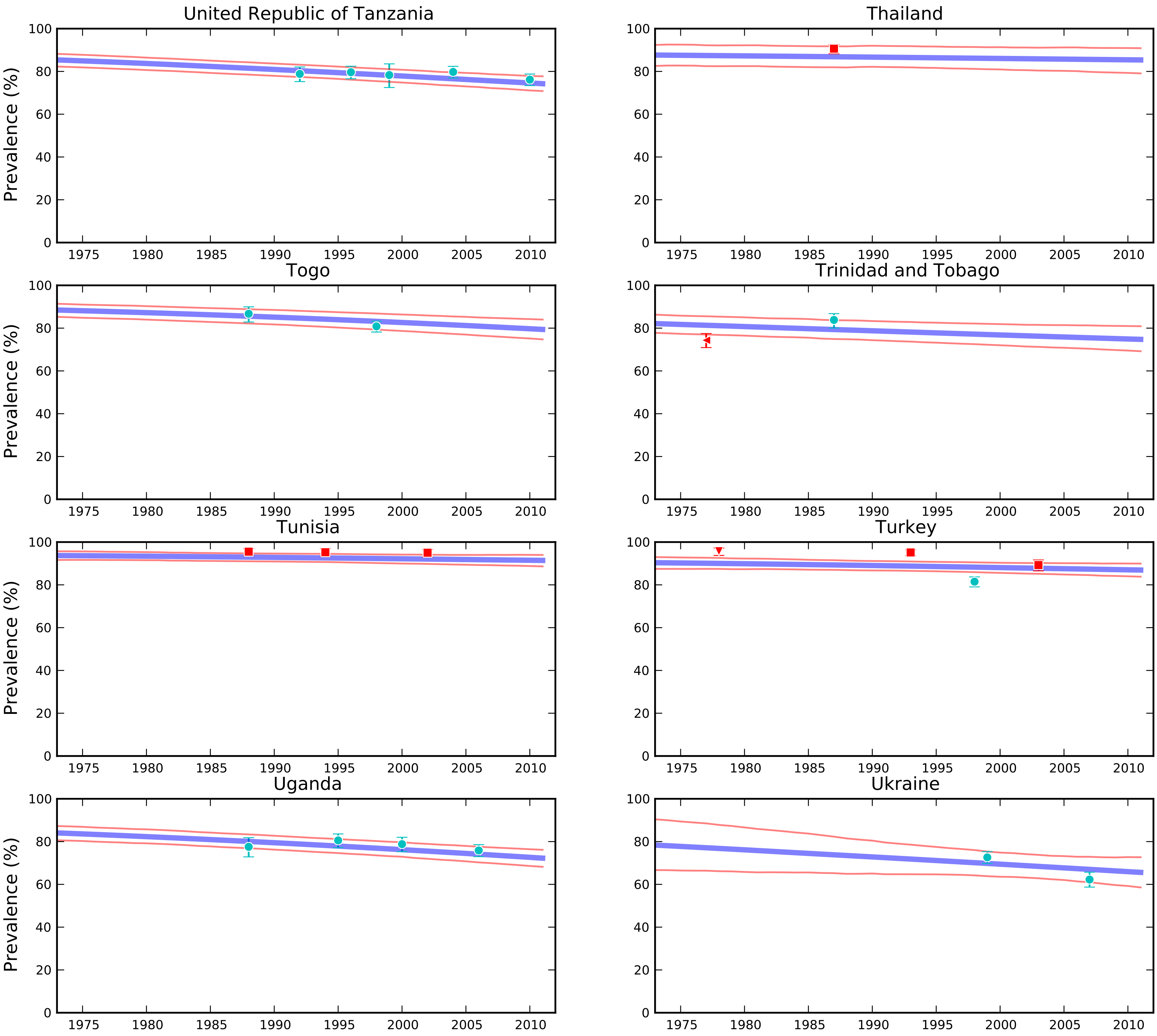
United Republic of Tanzania
Thailand
Togo
Trinidad and Tobago
Tunisia
Turkey
Uganda
Ukraine
Prevalence (%)
0
20
40
60
80
100
1975
1980
1985
1990
1995
2000
2005
2010

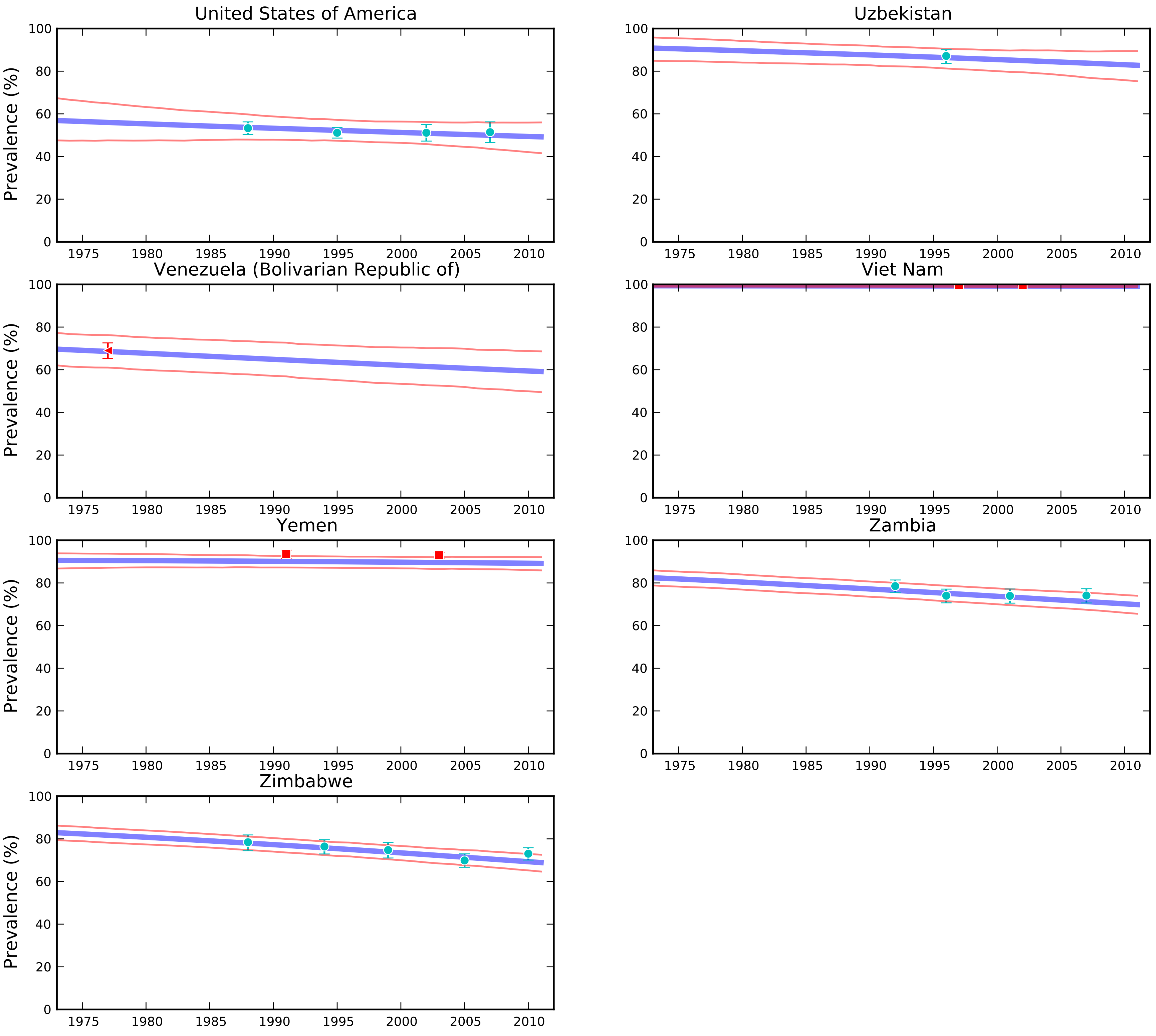
United States of America
Uzbekistan
Venezuela (Bolivarian Republic of)
Viet Nam
Yemen
Zambia
Zimbabwe
Prevalence (%)
0
20
40
60
80
100
1975
1980
1985
1990
1995
2000
2005
2010

Figure I: Trends in exposure to secondary infertility by year, shown for each country with data on following pages. Infertility is indexed on the female partner; age-standardized prevalence among women aged 20-44 years is shown here. Legend for plots is given below.

- Nationally representative (error bars represent standard errors)
- Nationally representative, data do not cover the entire 20-44 year age range
- Subnational
- Subnational, data do not cover the entire 20-44 year age range
- World Fertility Survey
- World Fertility Survey, data do not cover the entire 20-44 year age range
- Posterior means (model predictions)
- 95% credible interval

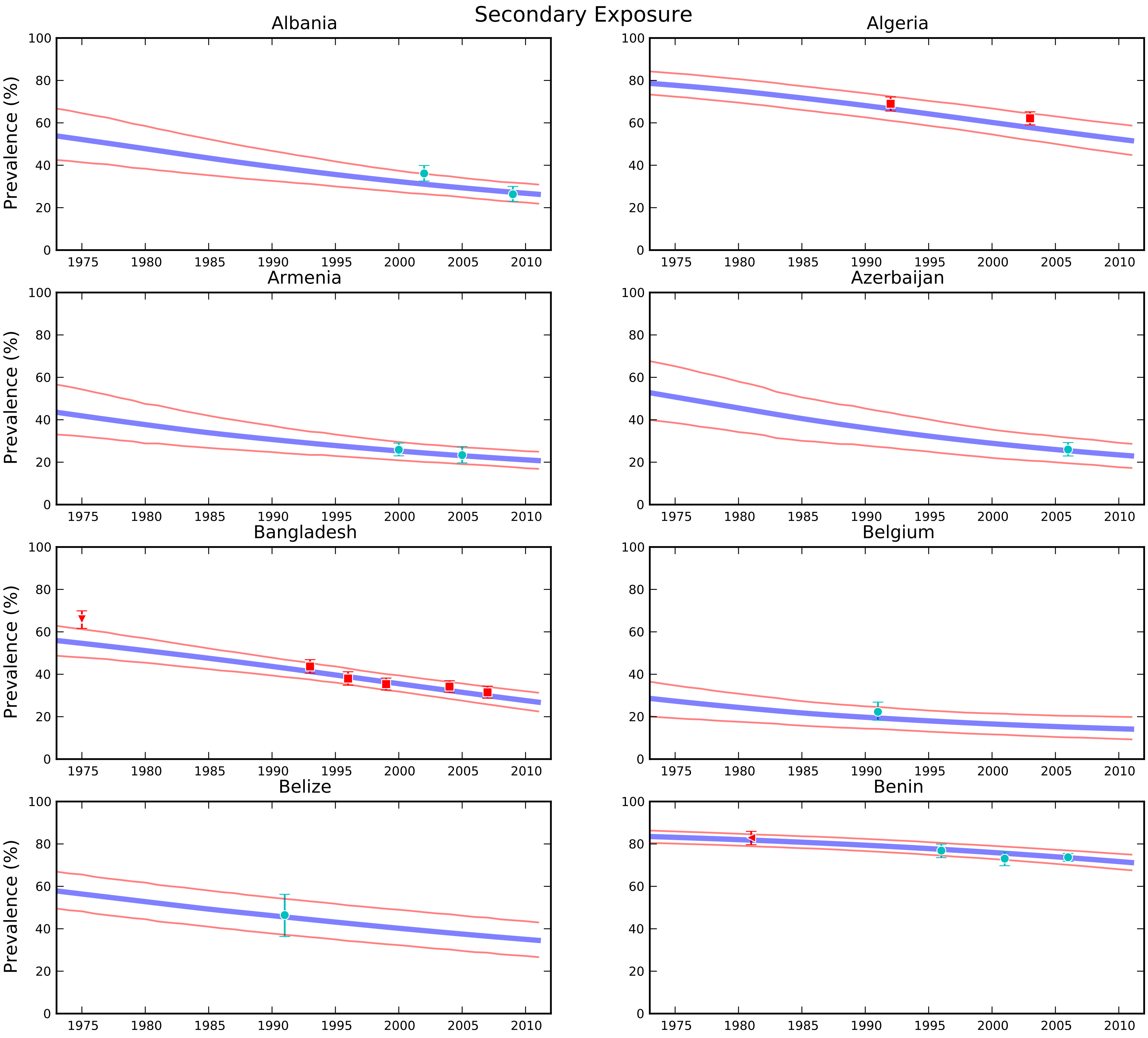

Secondary Exposure
Albania
Algeria
Armenia
Azerbaijan
Bangladesh
Belgium
Belize
Benin
Prevalence (%)
0
20
40
60
80
100
1975
1980
1985
1990
1995
2000
2005
2010

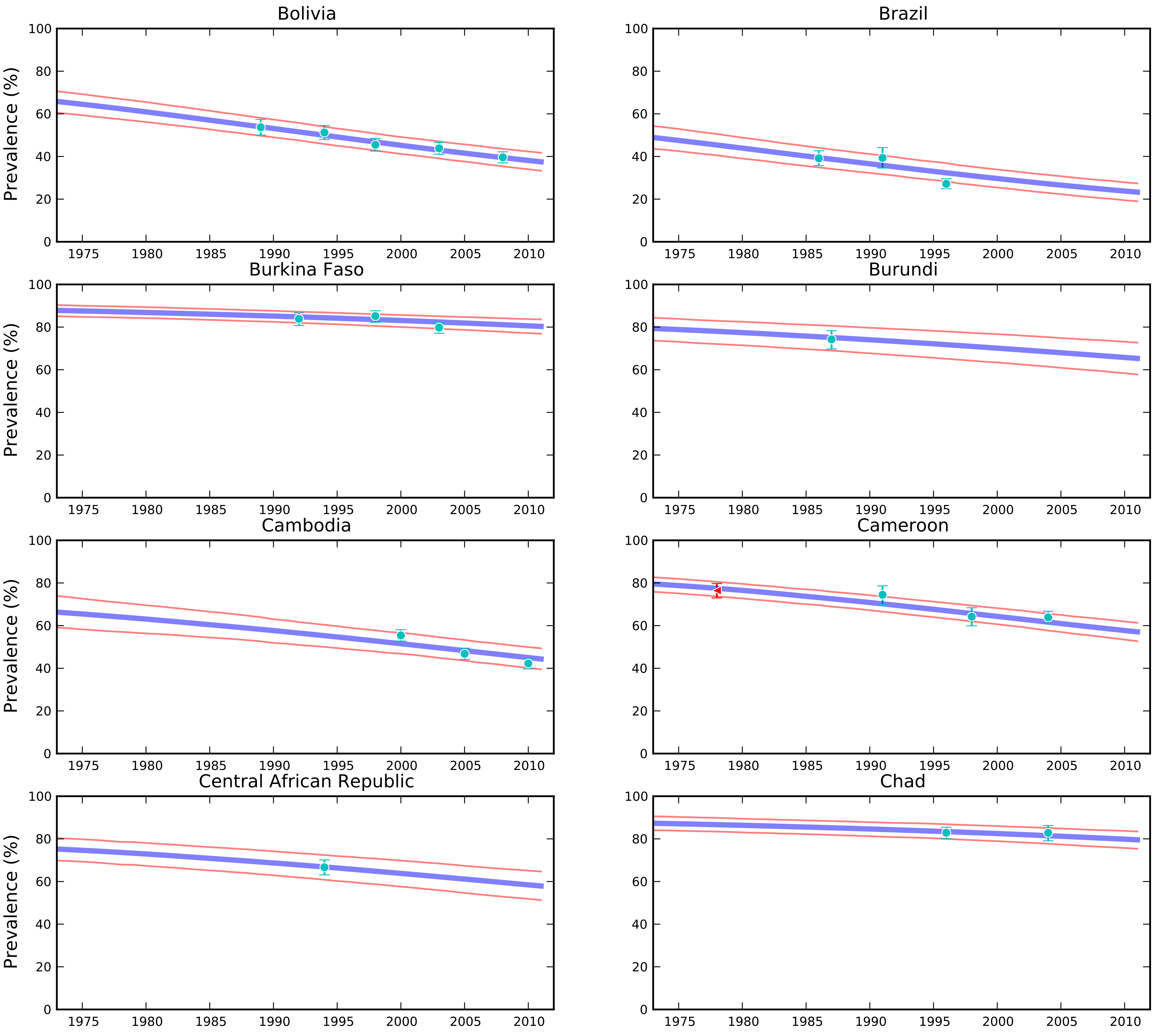
Bolivia
Brazil
Burkina Faso
Burundi
Cambodia
Cameroon
Central African Republic
Chad
Prevalence (%)
0
20
40
60
80
100
1975
1980
1985
1990
1995
2000
2005
2010

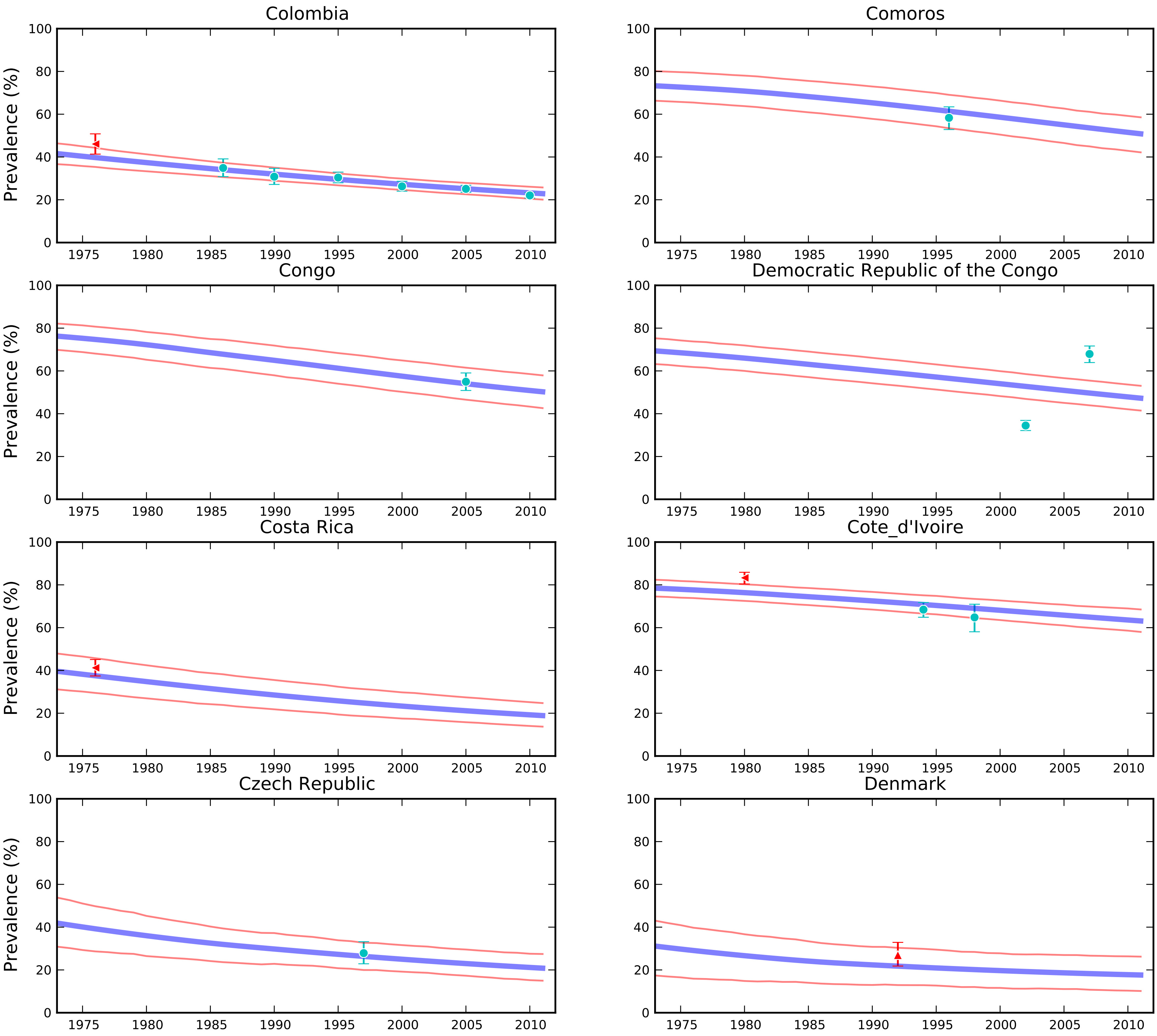
Colombia
Comoros
Congo
Democratic Republic of the Congo
Costa Rica
Cote_d'Ivoire
Czech Republic
Denmark
Prevalence (%)
0
20
40
60
80
100
1975
1980
1985
1990
1995
2000
2005
2010

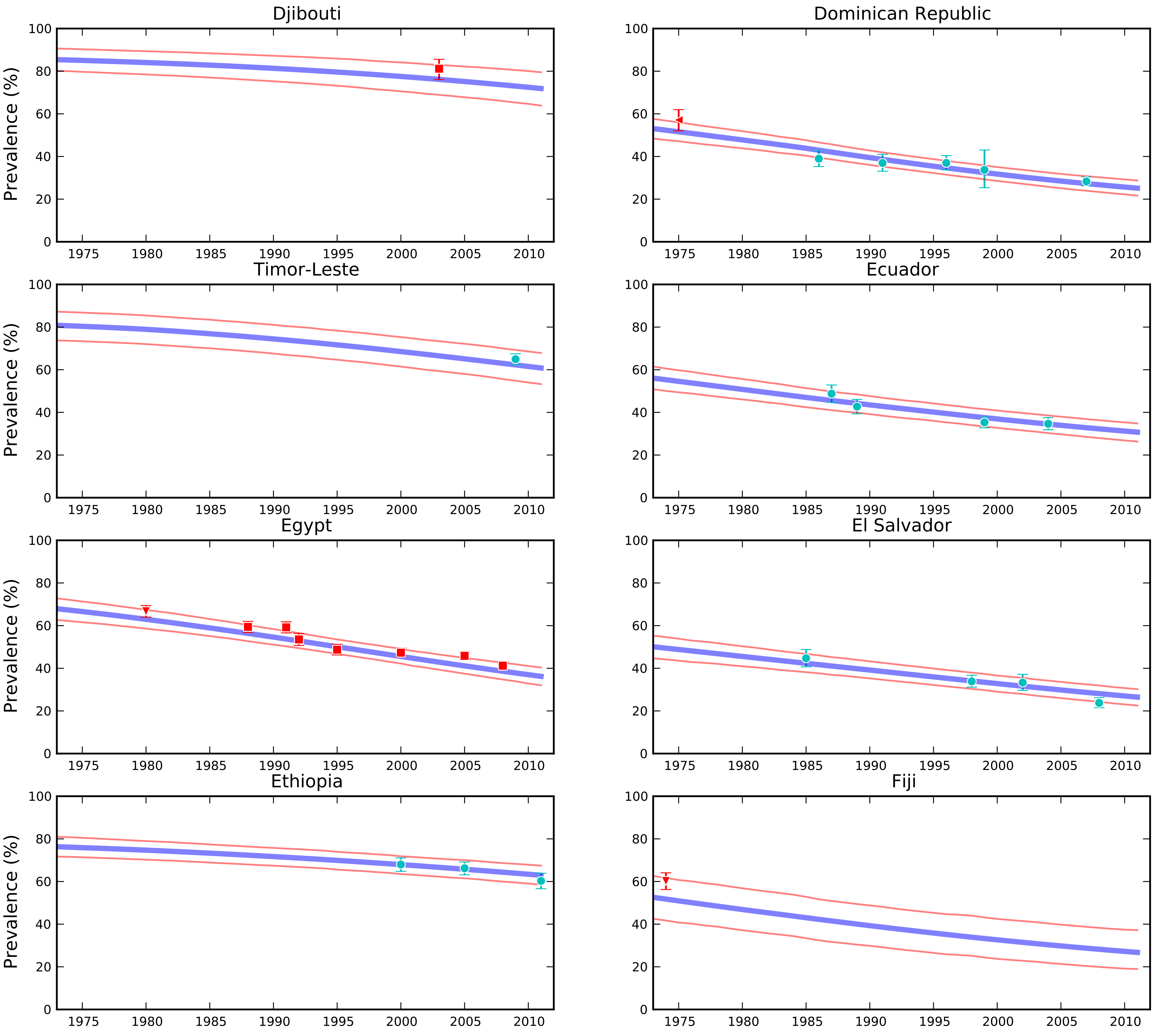
Djibouti
Dominican Republic
Timor-Leste
Ecuador
Egypt
El Salvador
Ethiopia
Fiji
Prevalence (%)
0
20
40
60
80
100
1975
1980
1985
1990
1995
2000
2005
2010

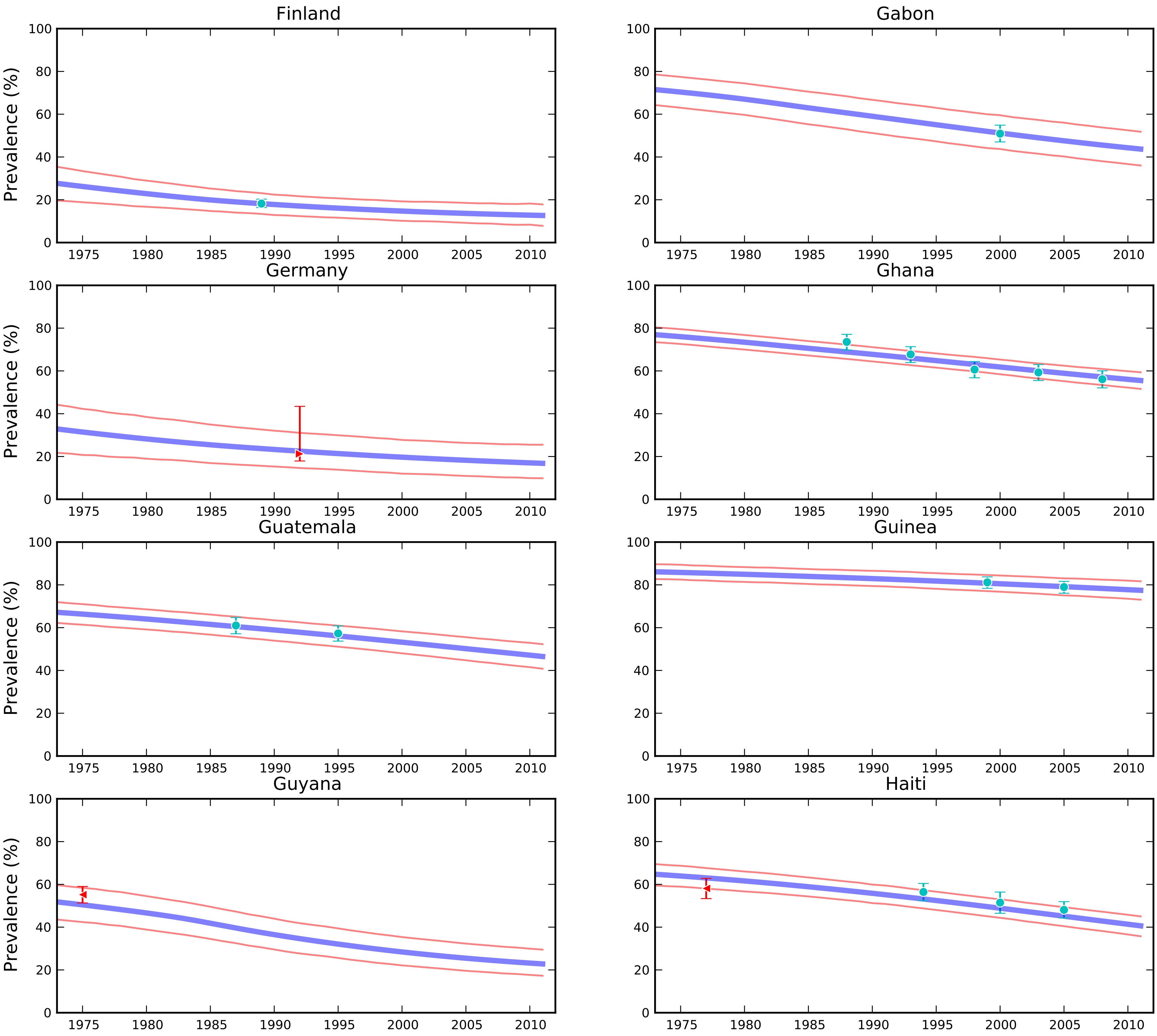

Finland
Gabon
Germany
Ghana
Guatemala
Guinea
Guyana
Haiti
Prevalence (%)
0
20
40
60
80
100
1975
1980
1985
1990
1995
2000
2005
2010

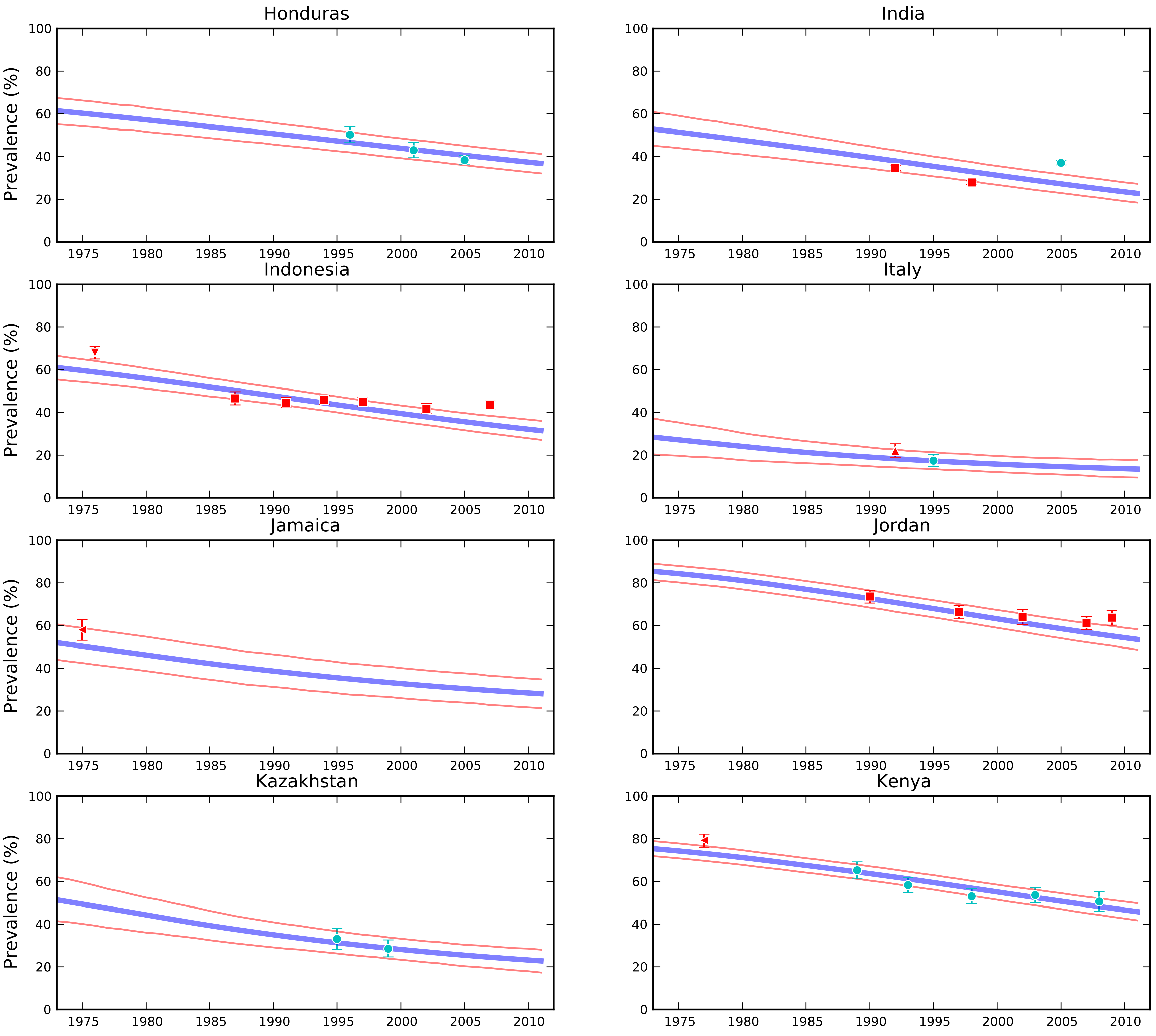

Honduras
India
Indonesia
Italy
Jamaica
Jordan
Kazakhstan
Kenya
Prevalence (%)
0
20
40
60
80
100
1975
1980
1985
1990
1995
2000
2005
2010

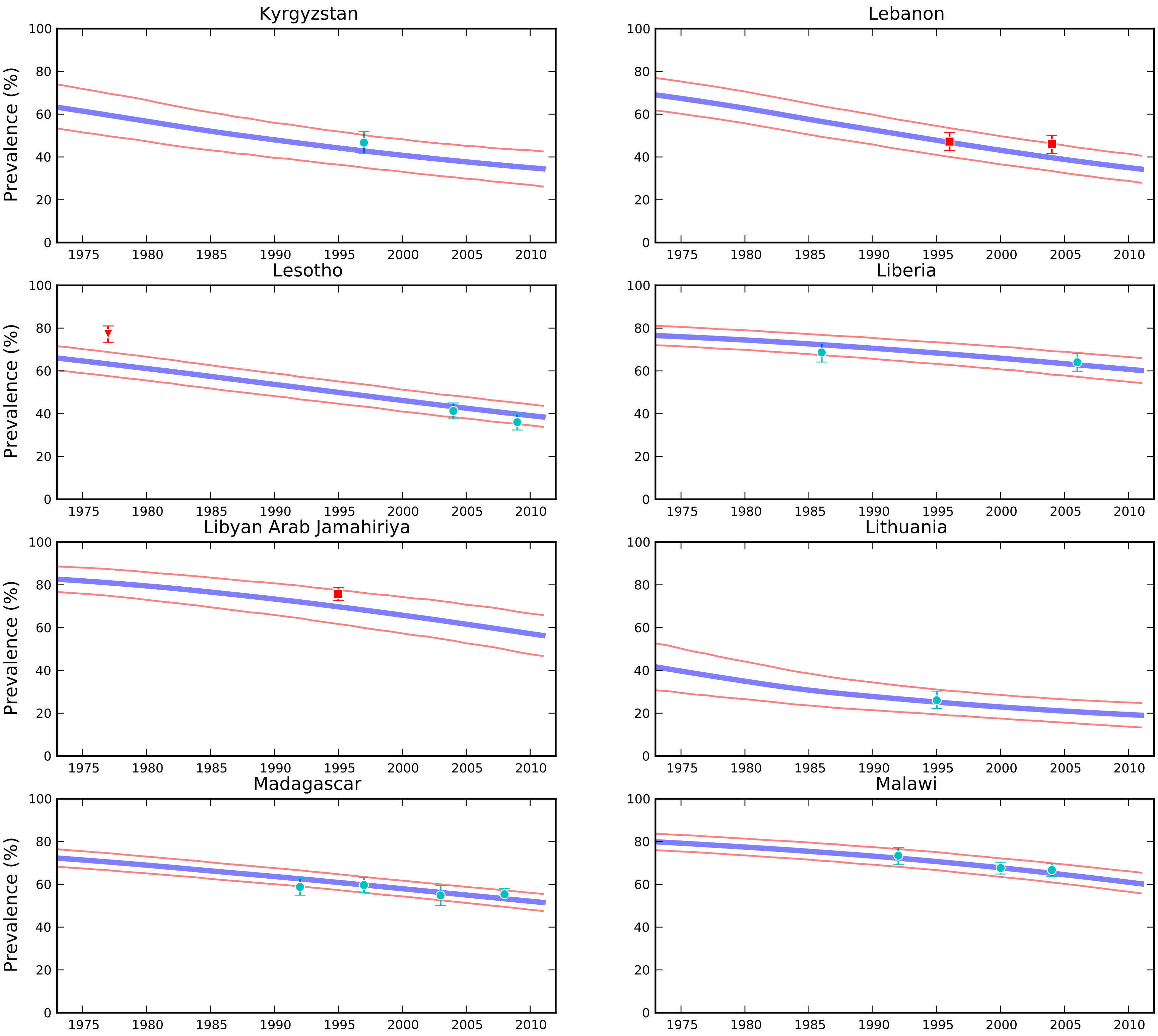
Kyrgyzstan
Lebanon
Lesotho
Liberia
Libyan Arab Jamahiriya
Lithuania
Madagascar
Malawi
Prevalence (%)
0
20
40
60
80
100
1975
1980
1985
1990
1995
2000
2005
2010

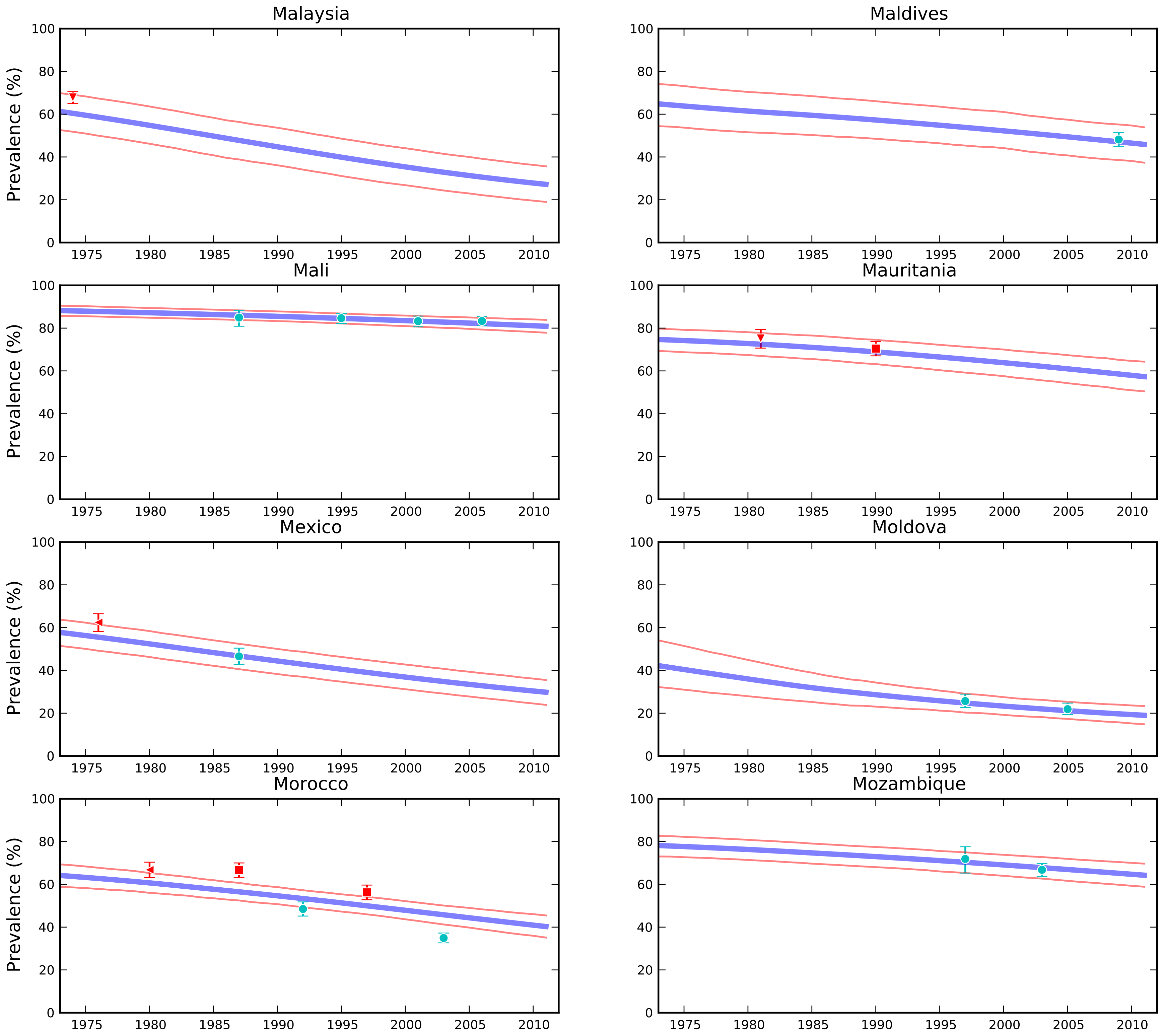
Malaysia
Maldives
Mali
Mauritania
Mexico
Moldova
Morocco
Mozambique
Prevalence (%)
0
20
40
60
80
100
1975
1980
1985
1990
1995
2000
2005
2010

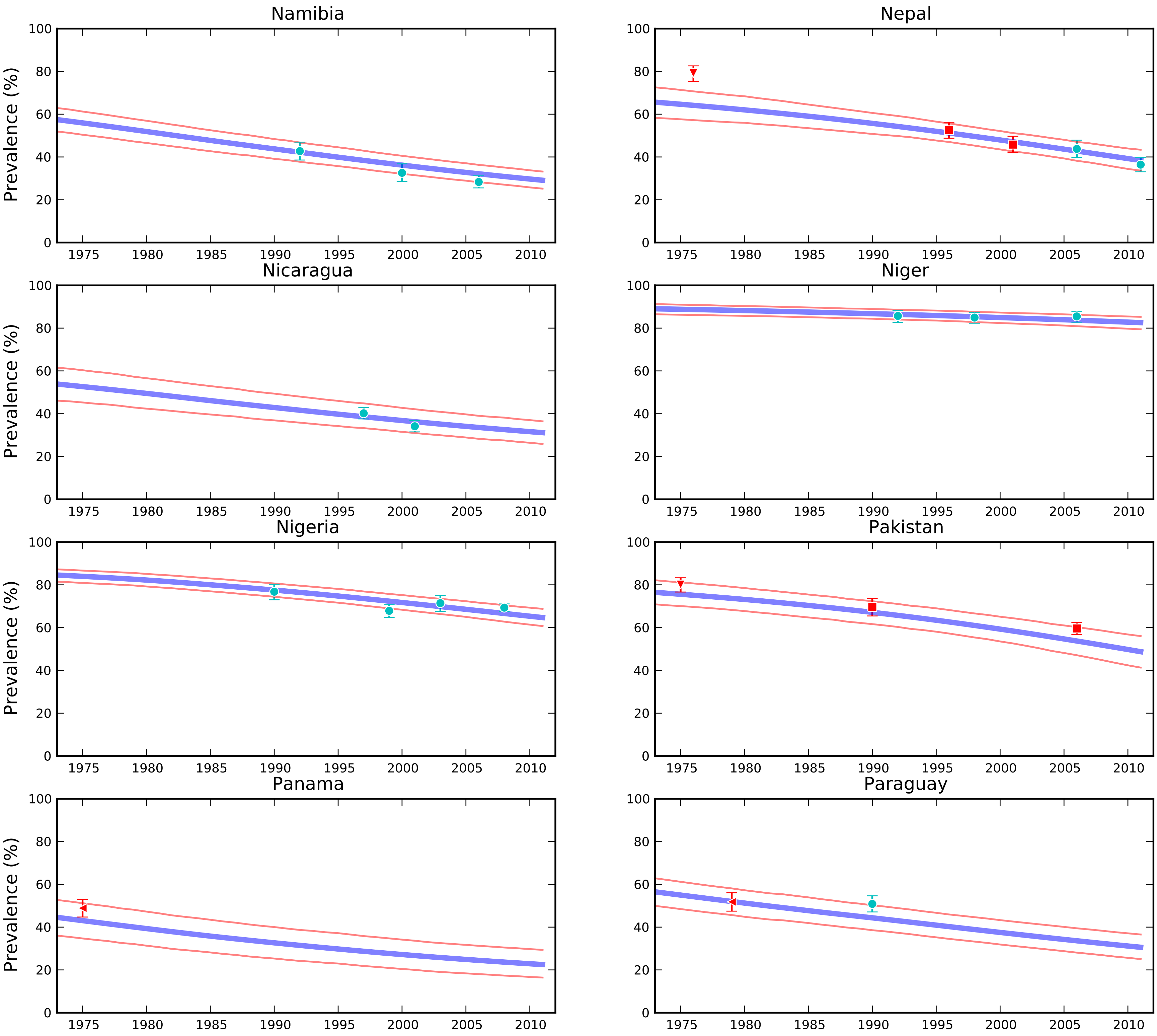

Namibia
Nepal
Nicaragua
Niger
Nigeria
Pakistan
Panama
Paraguay
Prevalence (%)
0
20
40
60
80
100
1975
1980
1985
1990
1995
2000
2005
2010

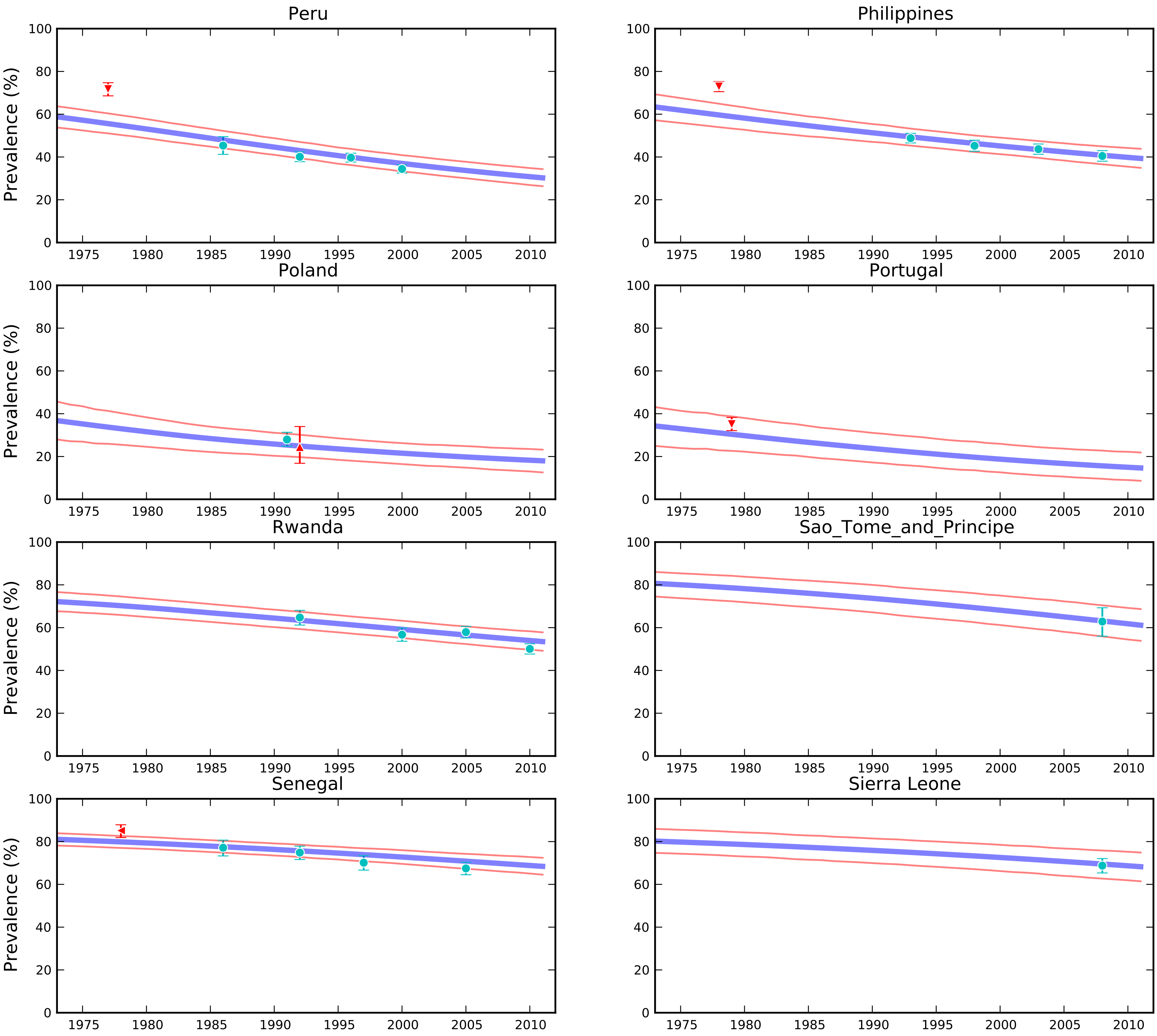
Peru
Philippines
Poland
Portugal
Rwanda
Sao_Tome_and_Principe
Senegal
Sierra Leone
Prevalence (%)
0
20
40
60
80
100
1975
1980
1985
1990
1995
2000
2005
2010

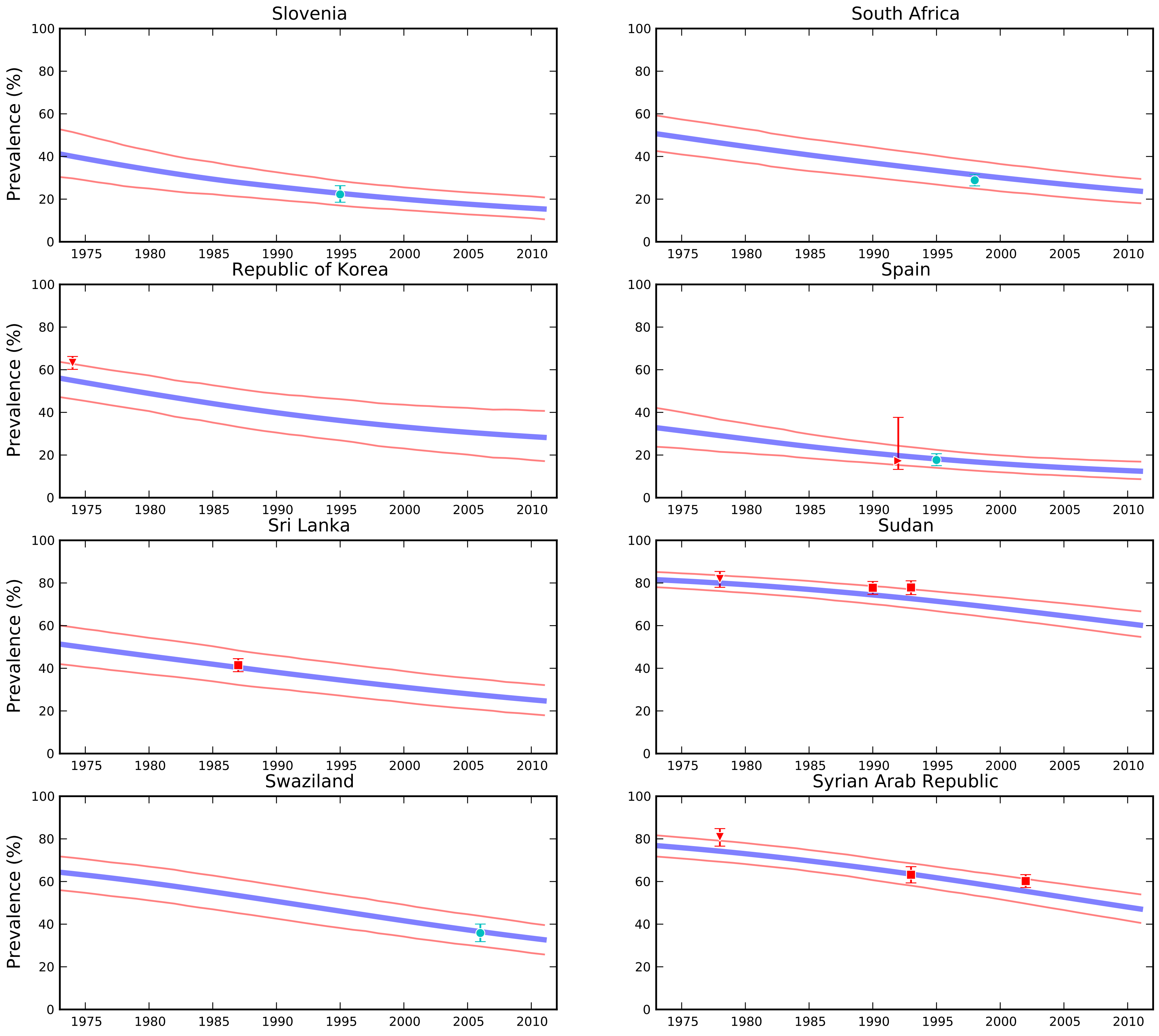
Slovenia
South Africa
Republic of Korea
Spain
Sri Lanka
Sudan
Swaziland
Syrian Arab Republic
Prevalence (%)
0
20
40
60
80
100
1975
1980
1985
1990
1995
2000
2005
2010

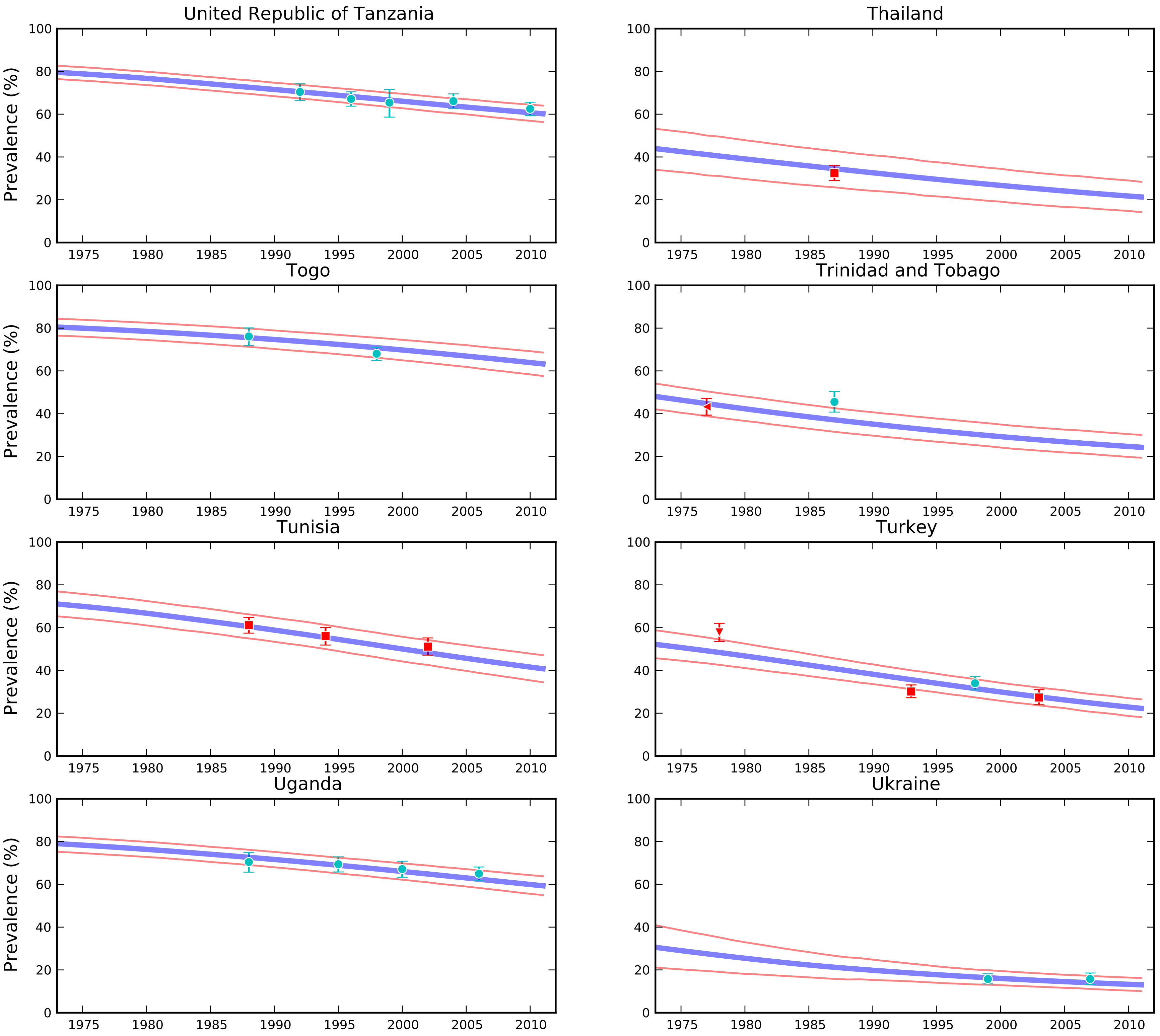
United Republic of Tanzania
Thailand
Togo
Trinidad and Tobago
Tunisia
Turkey
Uganda
Ukraine
Prevalence (%)
0
20
40
60
80
100
1975
1980
1985
1990
1995
2000
2005
2010

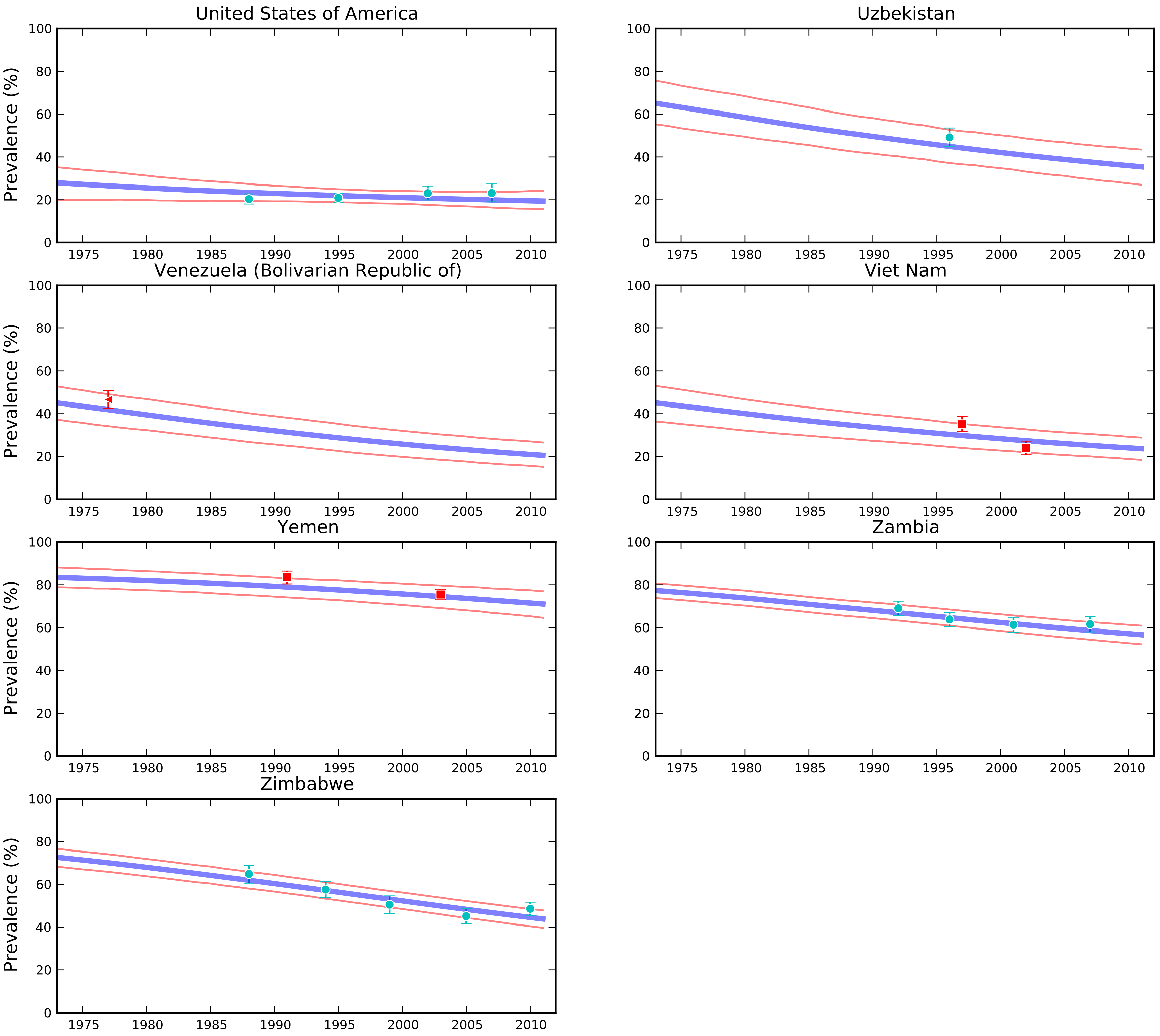
United States of America
Uzbekistan
Venezuela (Bolivarian Republic of)
Viet Nam
Yemen
Zambia
Zimbabwe
Prevalence (%)
0
20
40
60
80
100
1975
1980
1985
1990
1995
2000
2005
2010

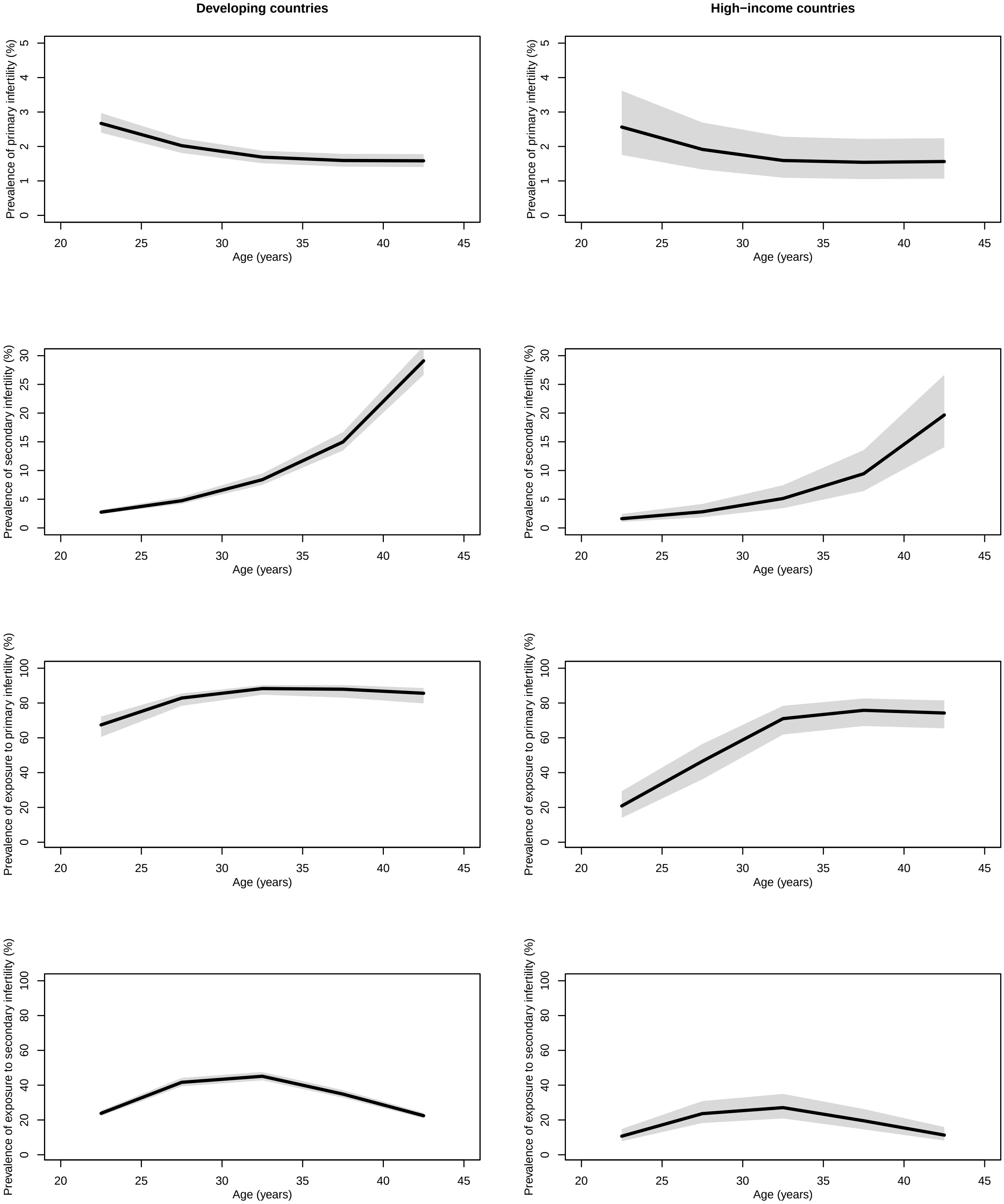

Figure J: Age pattern of infertility prevalence and exposure, by development status and type of infertility. All countries were modeled to have the same age pattern for primary infertility, secondary infertility, and exposure to secondary infertility; the age pattern for exposure to primary infertility varied by development status. See Text C for details.

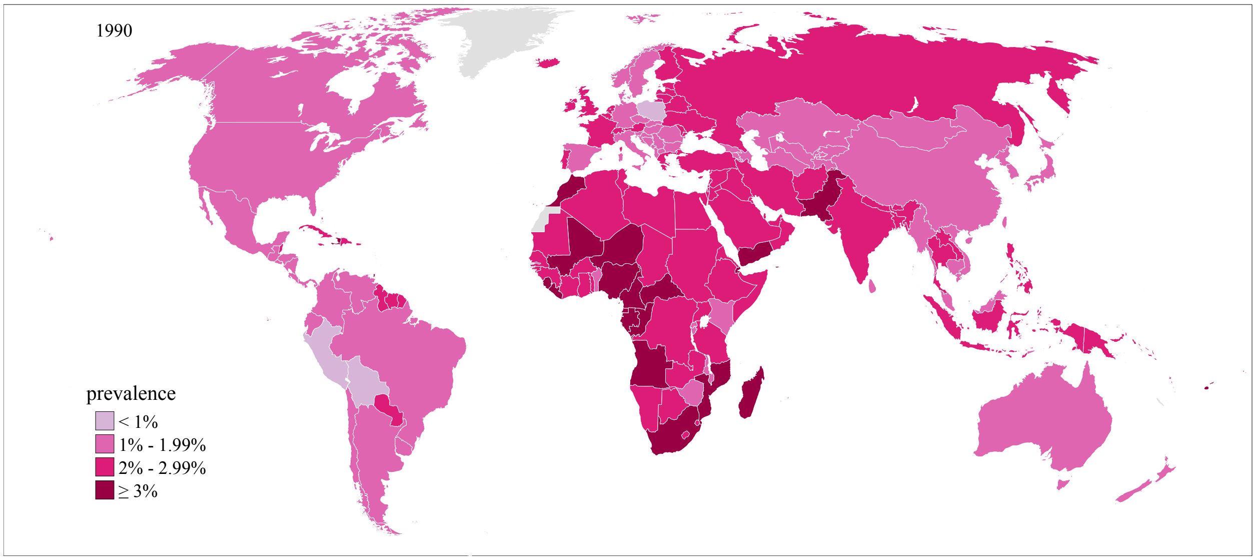

Figure K: Prevalence of primary infertility (out of women who seek a child) in 1990. (Map for 2010 is Figure 3 in the main text.) Infertility prevalence is indexed on the female partner; age-standardized prevalence among women aged 20-44 is shown here.

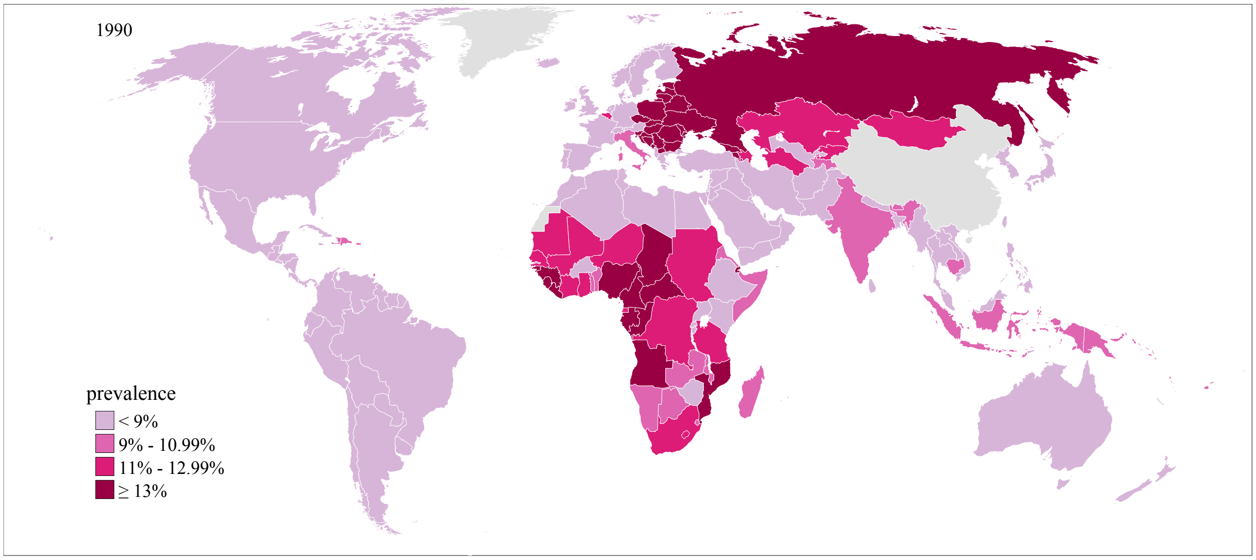

Figure L: Prevalence of secondary infertility (out of women who seek a child) in 1990. (Map for 2010 is Figure 4 in the main text.) Infertility prevalence is indexed on the female partner; age-standardized prevalence among women aged 20-44 is shown here.

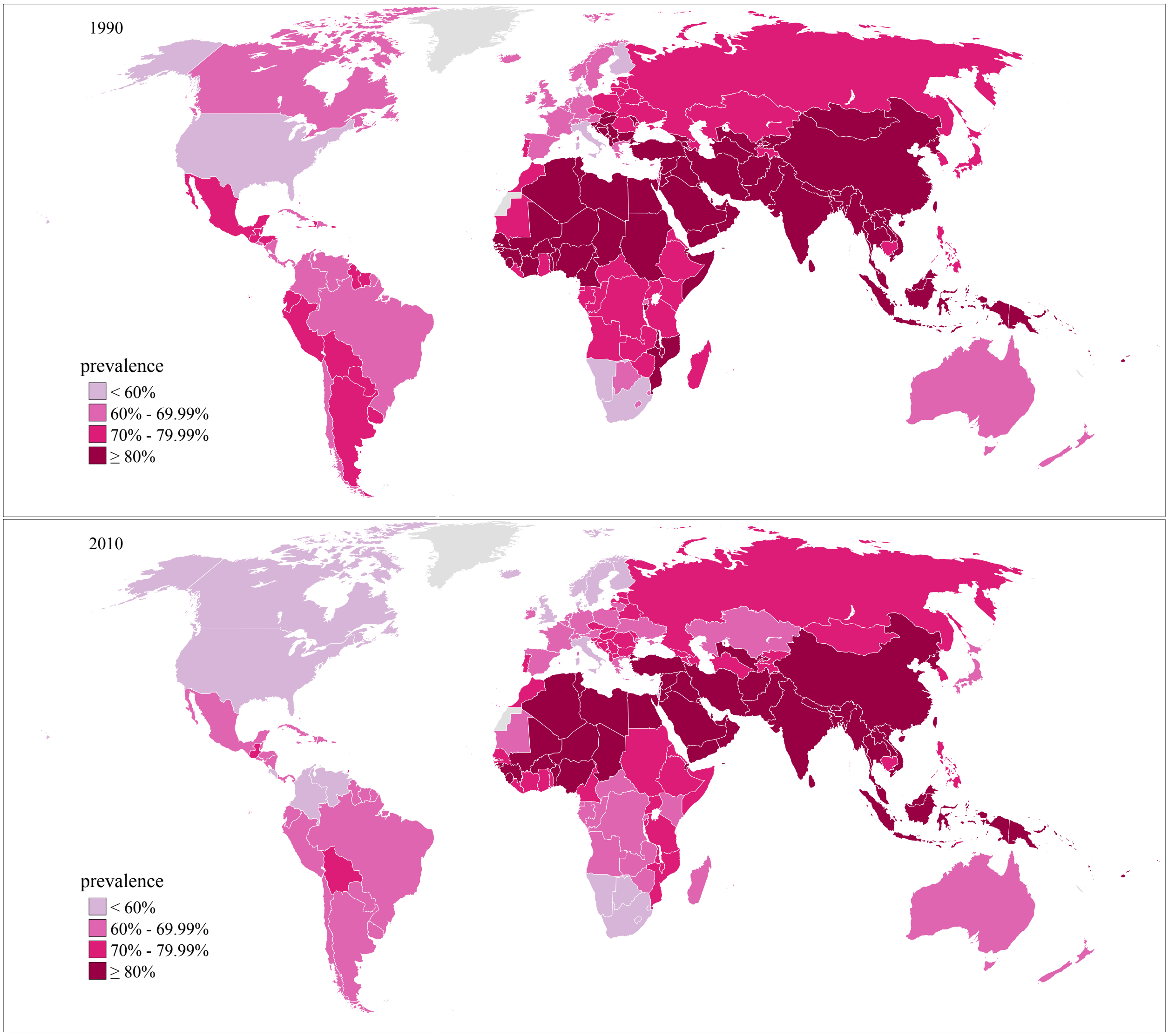

Figure M: Exposure to primary infertility (out of all women) in 1990 (top) and 2010 (bottom) Infertility exposure is indexed on the female partner; age-standardized prevalence among all women is shown here.

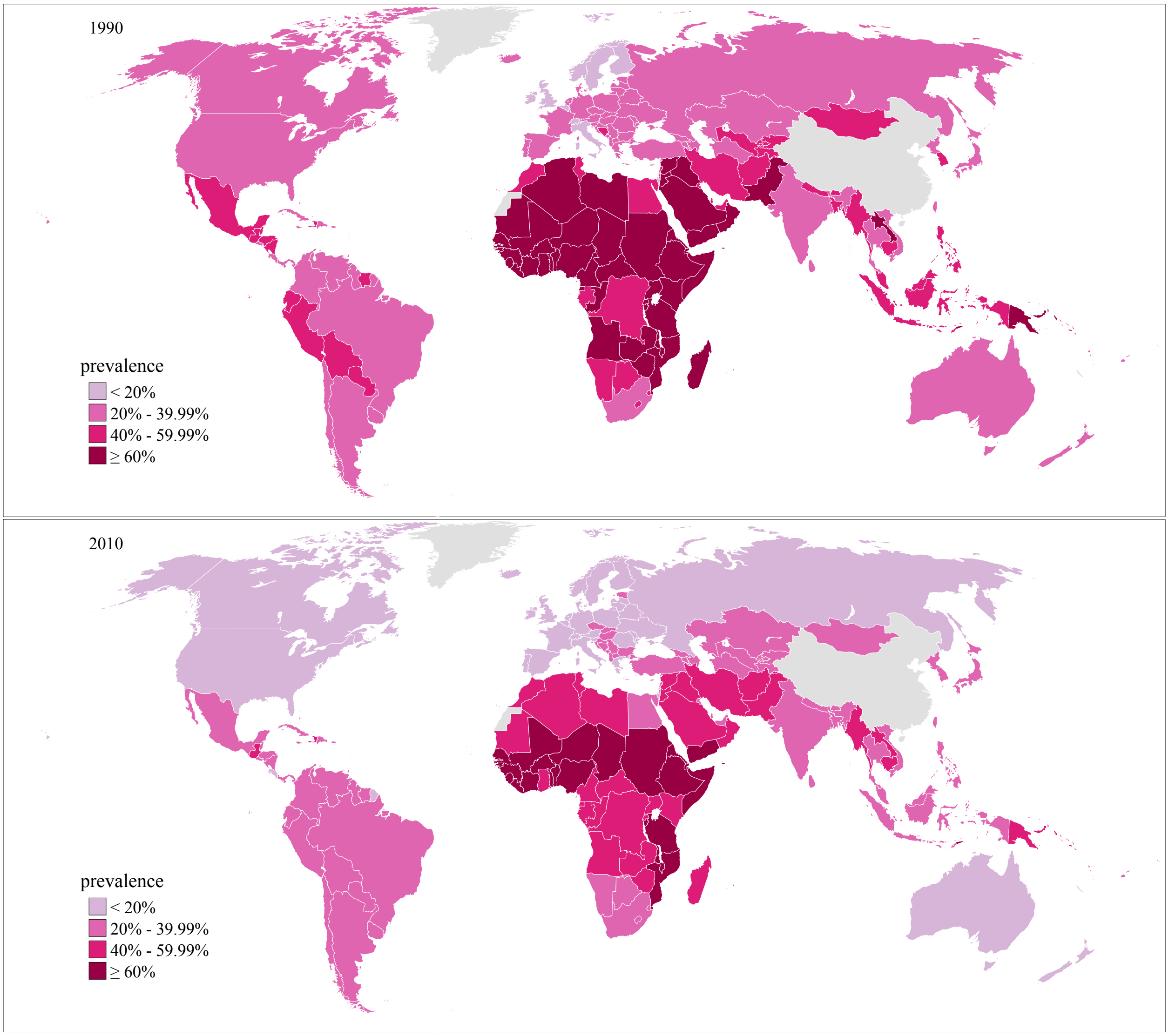

Figure N: Exposure to secondary infertility (out of all women) in 1990 (top) and 2010 (bottom). Infertility exposure is indexed on the female partner; age-standardized prevalence among all women is shown here.

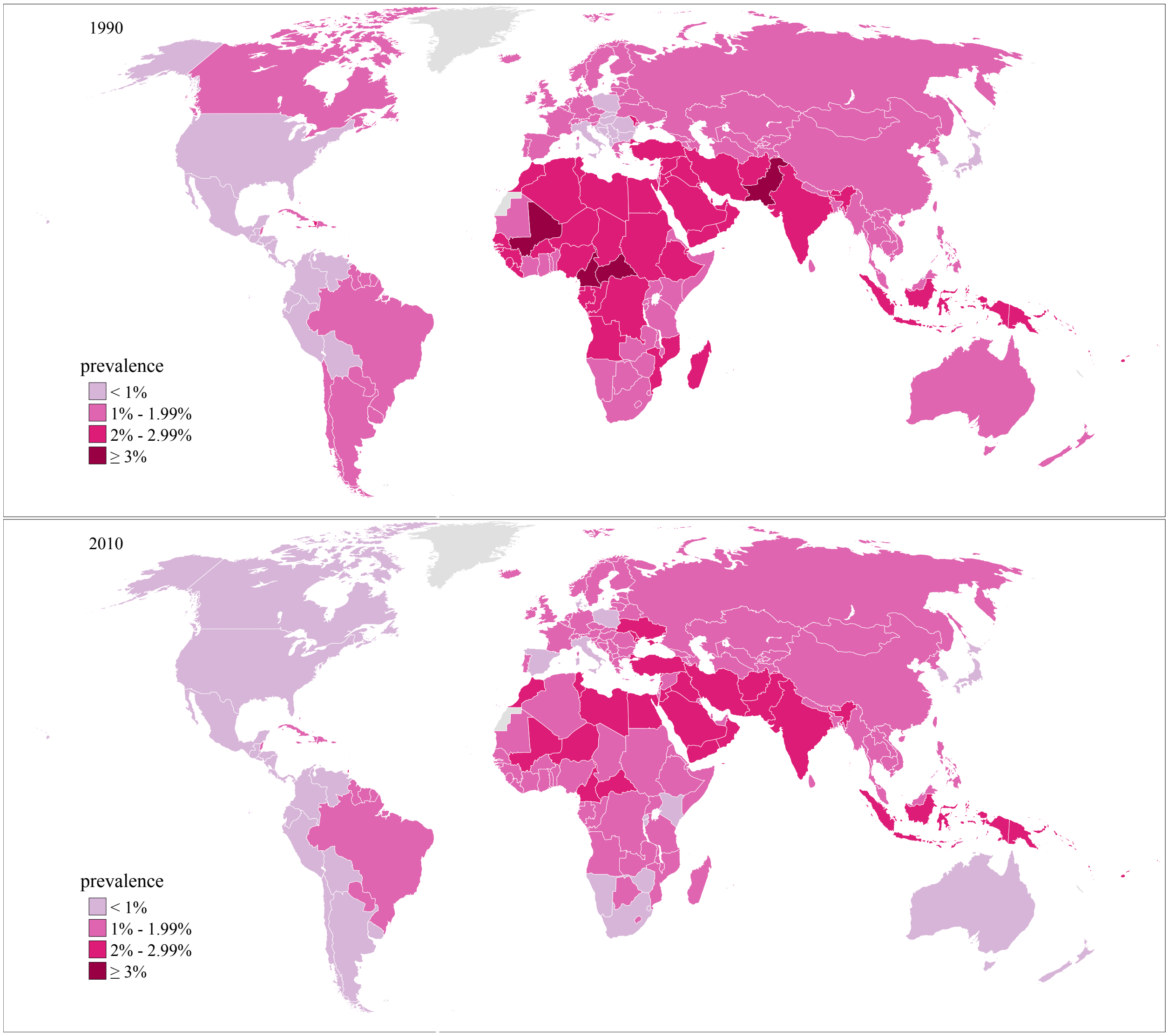

Figure O: Burden of primary infertility (out of all women) in 1990 (top) and 2010 (bottom). Infertility exposure is indexed on the female partner; age-standardized prevalence among women aged 20-44 is shown here.

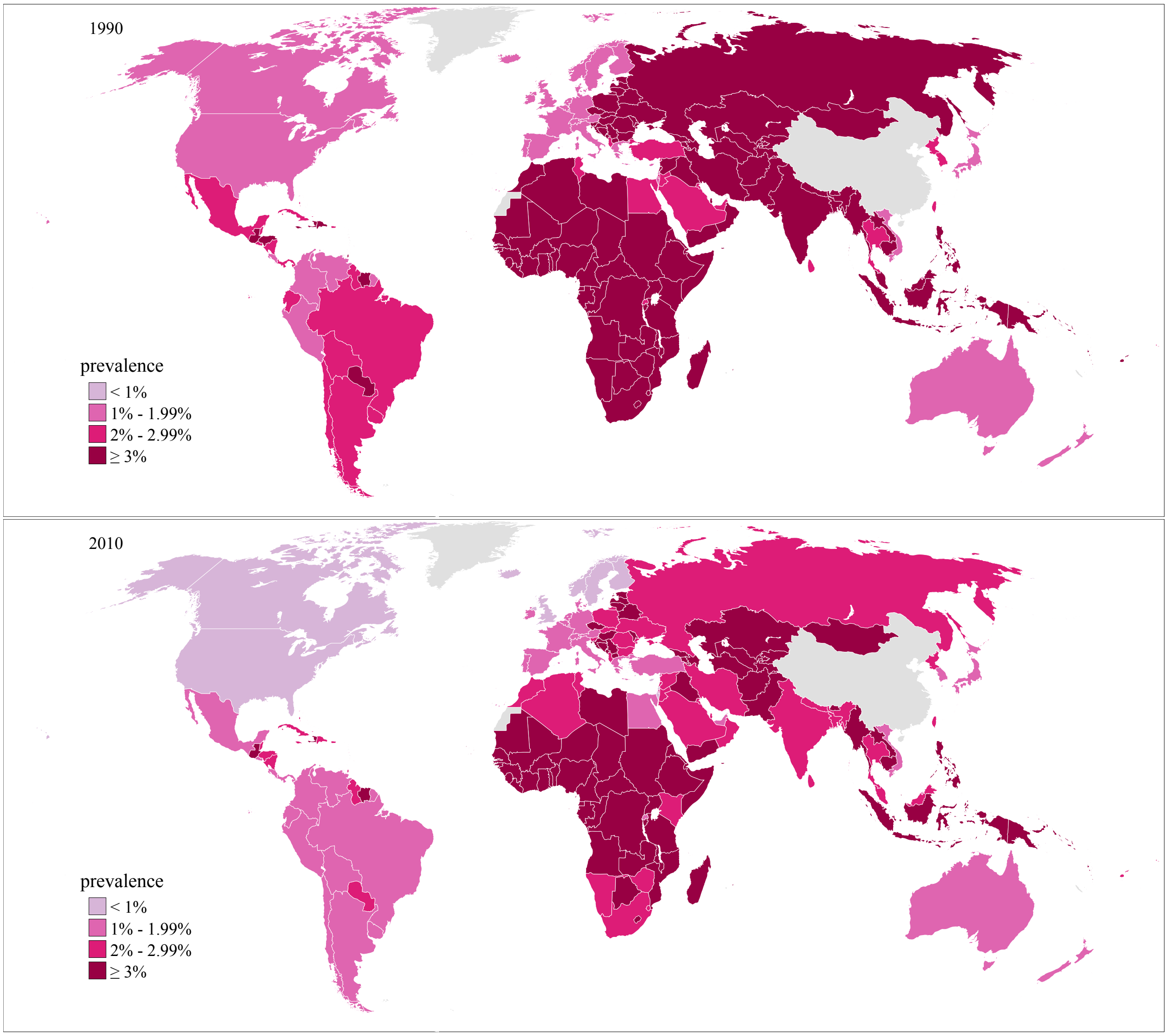

Figure P: Burden of secondary infertility (out of all women) in 1990 (top) and 2010 (bottom). Infertility exposure is indexed on the female partner; age-standardized prevalence among women aged 20-44 is shown here.

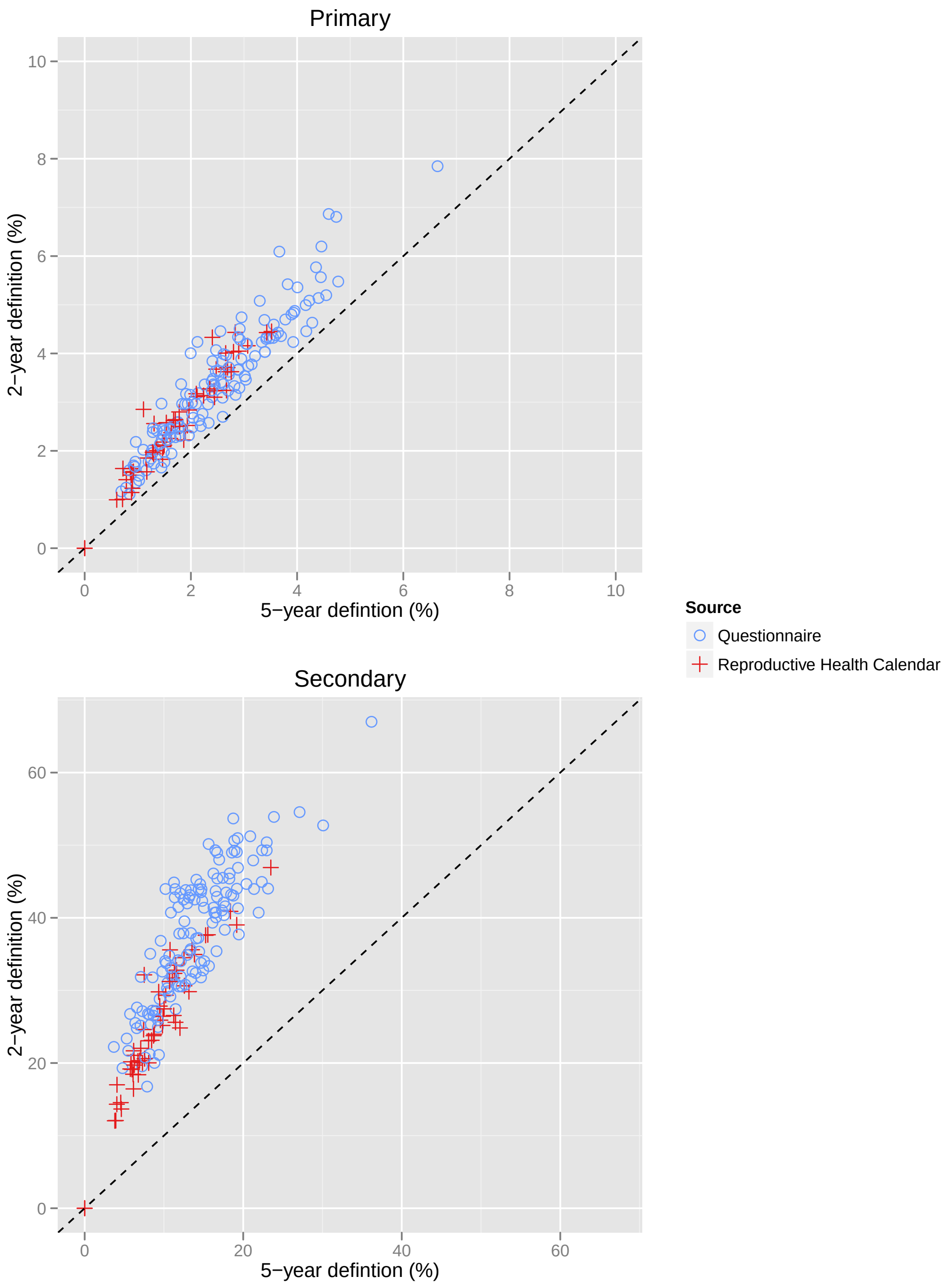

Figure Q: Results of a sensitivity analysis comparing a 2-year exposure period to a 5-year exposure period. Data from DHS surveys are shown; surveys using the reproductive health calendar collected information on past contraceptive use and union statues, whereas surveys using the questionnaire identify exposed unions using current contraceptive use and union status, without correction for incomplete information. Dotted lines indicate equal prevalence using the two exposure periods.
